# Cerebral Cortex Morphometry and Relaxometry in Male Children With Fragile X Syndrome and Autism

**DOI:** 10.1002/brb3.71375

**Published:** 2026-04-30

**Authors:** Jose M. Guerrero‐Gonzalez, Anna K. Lowe, Steven R. Kecskemeti, Brittany G. Travers, Andrew L. Alexander, Audra M. Sterling

**Affiliations:** ^1^ Waisman Center University of Wisconsin‐Madison Madison Wisconsin USA; ^2^ Department of Medical Physics University of Wisconsin‐Madison Madison Wisconsin USA; ^3^ Occupational Therapy Program in the Department of Kinesiology University of Wisconsin‐Madison Madison Wisconsin USA; ^4^ Department of Psychiatry University of Wisconsin‐Madison Madison Wisconsin USA; ^5^ Department of Communication Sciences and Disorders University of Wisconsin‐Madison Madison Wisconsin USA

**Keywords:** autism, fragile X syndrome, gray‐matter thickness, magnetic resonance imaging, myelin, sensory processing

## Abstract

**Purpose:**

An estimated 30%–50% of male individuals with fragile X syndrome (FXS) meet criteria for autism spectrum disorder (ASD), indicating phenotypic overlap but potentially distinct neurobiology. Here, we aimed to characterize shared and divergent cortical features between FXS and ASD.

**Method:**

High‐resolution, motion‐corrected quantitative MRI was used to compare cortical morphometry and relaxometry in 61 male participants (9–18 years) with FXS or ASD. To increase power, ASD participants were pooled from multiple MPnRAGE studies and harmonized across protocols using ComBat. Cortical thickness and R1 (longitudinal relaxation rate; proxy for myelination) were computed across the cerebral cortex.

**Finding:**

Relative to ASD, FXS exhibited greater cortical thickness predominantly in early sensory cortices implicated in low‐level visual and auditory processing spanning occipital, parietal, and temporal regions. No significant group differences in R1 were found.

**Conclusion:**

Thicker cortex in FXS within primary and early associative sensory areas suggests divergent early sensory processing mechanisms between FXS and ASD. Characterizing different neuroanatomical features between the two disorders provides a grounding to develop more disorder‐specific interventions despite similar behavioral difficulties. Future work should test developmental trajectories, include females and comorbidities, and link imaging markers to individual sensory/clinical profiles to inform and improve personalized therapies and interventions.

## Graphical abstract

In this study, regions of the cortex highlighted in shades of blue indicate children with fragile X syndrome have increased cortical thickness compared to children with autism spectrum disorder.

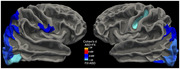

## 1 Introduction

Autism spectrum disorder (ASD) is defined behaviorally and represents a clinically heterogeneous syndrome rather than a single etiologic entity (American Psychiatric Association 2013). The term “idiopathic” (or “non‐syndromic”) ASD denotes cases without an identified genetic syndrome or known medical cause but does not imply a uniform underlying mechanism. The prevalence of autism is relatively high, with current estimates at 1 in every 31 children (Shaw et al. 2025). While fragile X syndrome (FXS) is an inherited disorder resulting from an expansion on the *FMR1* gene located on the X chromosome, it is also phenotypically heterogeneous, with substantial variability in cognitive functioning, autism‐related traits, and comorbid features. With an estimated prevalence of 1 in 3600–5000 males (Hunter et al. 2014; Verkerk et al. 1991), FXS is characterized by moderate to severe intellectual disability and communication impairments including global delays in expressive and receptive vocabulary, grammar, and reduced intelligibility (Crawford et al. 2001; Hagerman and Hagerman 2002), with males often being more severely affected than females due to the X‐linked nature of the disorder (Hagerman and Hagerman 2002).

Research to date suggests that ∼30%–50% of individuals with FXS meet full ASD diagnostic criteria, making FXS the most‐studied monogenetic model of ASD (Abbeduto et al. 2014; Ciaccio et al. 2017; Devitt et al. 2015; Kaufmann et al. 2017; Leigh and Hagerman 2020; McDuffie et al. 2010; Wilkinson and Nelson 2021). However, comparative behavioral studies also suggest marked distinctions between the two disorders across domains. For instance, while both conditions share core social‐communication impairments, FXS combines relatively preserved receptive vocabulary with greater gaze avoidance and social anxiety, whereas idiopathic ASD manifests broader pragmatic‐language dysfunction (Klusek et al. 2014; Maltman et al. 2023). Further, pragmatic‐language studies have reported overlapping discourse difficulties, but boys with FXS omit more function words and show greater narrative incoherence than matched autistic peers (Klusek et al. 2014; Maltman et al. 2023). Their repetitive behavior profiles also differ. FXS is marked by high‐frequency motor stereotypies but fewer insistence‐on‐sameness rituals (Reisinger et al. 2020). Both groups also display sensory hyper‐responsivity, but studies suggest more pervasive auditory, tactile, and visual hypersensitivity in FXS (Rais et al. 2018). Additionally, a recent review describes that studies have identified shared working‐memory and set‐shifting weaknesses, with more severe attentional‐inhibition deficits in FXS (Schmitt et al. 2019). Together, these findings suggest partially divergent neurodevelopmental mechanisms despite superficially similar phenotypes.

Studies using magnetic resonance imaging for direct examinations between FXS and ASD remain limited, but some have begun to make efforts to disentangle our understanding of these mechanisms. A voxel‐based morphometry (VBM) study of adolescents and adults reported that FXS is associated with relative gray‐matter reductions in insular, prefrontal, and cerebellar regions, whereas idiopathic autism shows enlargements in medial temporal and parietal areas (Wilson et al. 2009). In toddlers, studies suggest that neurodevelopment also appears to diverge between FXS and ASD. Hazlett and colleagues found that despite comparable levels of autistic behavior, children with FXS displayed smaller temporal‐limbic volumes and larger caudate nuclei, whereas autistic individuals showed the opposite pattern, underscoring distinct patterns of brain morphology despite overlapping behavioral features emerging as early as two years of age (Hazlett et al. 2009). Further, in a surface‐based analysis, Hoeft et al. reported widespread cortical thinning in prefrontal and superior temporal association areas in FXS toddlers, contrasted with focal thickening in idiopathic autism, alongside opposite volumetric trends in the amygdala–insula salience network (Hoeft et al. 2011). These findings complement the earlier VBM results by Wilson et al. (2009) and more recent longitudinal work showing disorder‐specific subcortical growth trajectories across infancy (Shen et al. 2022). Collectively, these findings indicate that, despite overlapping clinical presentations, FXS and autism follow dissociable cortical‐subcortical developmental paths. However, systematic comparisons of neuroanatomical features between the two conditions that span wider age ranges are scarce and represent a critical gap in this area of research.

Cortical thickness has long been proposed to be associated with age and cognitive functioning. This measure has been shown to increase during childhood, with subtle decreases throughout adolescence, and a sharper decline with older age (De Chastelaine et al. 2019; Fjell et al. 2009; Kelly et al. 2024; Zhou et al. 2013). In childhood specifically, studies have shown lower cortical thickness values in parietal and occipital cortices, with temporal regions showing small increases during middle childhood and preadolescence (Kelly et al. 2024). The observed cortical thickness changes across different ages may serve different purposes and be related to different processes such as myelination, reorganization of dendritic spines, and synaptic pruning (Fjell et al. 2009; Kelly et al. 2024). For adolescents, these processes may be involved in optimizing neural connectivity efficiency, while in aging individuals, cortical thinning may exceed a threshold point in regions such as the frontal cortex, leading to negative deficits in cognition and executive function (De Chastelaine et al. 2019; Fjell et al. 2009; Kelly et al. 2024; Zhou et al. 2013).

The lack of neuroimaging comparative studies between FXS and ASD may in part be due to difficulties that arise when imaging pediatric populations with intellectual and developmental disabilities. In particular, one significant limitation when imaging non‐sedated children is bulk‐motion‐related imaging artifacts that degrade image quality. Motion can lead to signal loss or extreme blurring that limits accurate segmentation of boundaries and fine cortical gray‐matter structures. To address this limitation, in this study, we employed motion‐robust quantitative brain imaging to enable accurate cortex segmentation and thickness estimates in children with FXS and ASD. The imaging sequence we used is based on motion‐corrected 3D anatomical MPnRAGE (Magnetization Prepared n‐contrast Rapid Gradient Echo (Kecskemeti et al. 2016), a method shown to significantly improve image quality in both neurotypical and neurodivergent pediatric populations (Kecskemeti et al. 2018,2021; Kecskemeti and Alexander 2020).

Cortical thickness is a macro‐structural measure and therefore includes contributions of multiple microstructural features including myelination and cell density. While studies have investigated microstructural features in FXS and ASD, most have focused on white matter integrity using fractional anisotropy (FA) and other metrics from diffusion tensor imaging (DTI; Green et al. 2015; Hall et al. 2016). Findings have included FA values that are significantly higher in FXS than in ASD of the inferior longitudinal fasciculus, right uncinate fasciculus, and left cingulum (Green et al. 2015; Hall et al. 2016). These regions as well as the corpus callosum, inferior fronto‐occipital fasciculus, and anterior cingulate cortex have also been found to show FA differences in comparisons between ASD and neurotypical individuals (Peterson et al. 2022). The gray‐matter regions connected by these white matter structures are the computational endpoints that likely contribute to the mechanisms underlying the observed differences in these studies. Furthermore, aberrant myelination in gray matter has been identified as one of only two consistent histological findings in a recent systematic review of post‐mortem autism studies (Fetit et al. 2021), and thus gray‐matter microstructure as related to myelination merits priority consideration for current studies of ASD and FXS.

While FA is a valuable measure to investigate microstructure integrity, it is sensitive to multiple features including axonal orientation dispersion, density, and myelination. Additionally, FA estimation is less reliable in gray matter due to multiple factors such as noise in the diffusion MRI signal, a less prominent presence of highly organized tissue fibers, and partial voluming from large voxel sizes (∼2 mm). The longitudinal relaxometry metric, T1 (Kohli et al. 2022), quantifies the time for the longitudinal magnetization recovery to its equilibrium state. It has long been recognized that differences in qR1 (qR1 = 1/T1) values are related to levels of myelination (Koenig 1991; Mottershead et al. 2003; Turner 2019). Therefore, qR1 has the potential to provide specific biophysical information that can be linked to phenotypic traits across neurodevelopmental disorders both in white and gray‐matter tissues. One added benefit of MPnRAGE is that it supports whole‐brain high‐resolution estimation of qR1 and therefore was used in this study to estimate average qR1 values across regions of the cortex in children with FXS and ASD.

Comparing samples from these two populations directly may provide insights into shared and distinct neuroanatomical features that underlie their observed behavioral similarities and differences. In the current study, we used motion‐corrected 3D anatomical MPnRAGE imaging to investigate cortical thickness and qR1 in a sample of male children with FXS and ASD. We hypothesized that *cortical thickness and qR1 values differ between FXS and ASD groups, particularly in sensory processing cortices*. Importantly, the present study contrasts cortical measures in a monogenetic neurodevelopmental condition (FXS) with those in a clinically defined, etiologically diverse ASD group, and our inferences are limited to group‐level differences between these heterogeneous populations. Still, by integrating the advanced neuroimaging methodologies of the current study with future specific phenotypic characterizations, this work has the potential to add valuable knowledge about the neurobiological basis of FXS and ASD, which is needed for informing future diagnostic and therapeutic approaches.

Because MRI studies in pediatric neurodevelopmental disorders often face recruitment and motion‐related constraints, adequately powered samples are challenging to obtain within a single study. To increase statistical power while retaining a consistent acquisition framework, we augmented the ASD sample by pooling participants from multiple MPnRAGE studies acquired on the same scanner but with minor protocol differences. As multi‐study pooling can introduce protocol‐related shifts in quantitative measures, particularly relaxometry, we applied ComBat harmonization (Fortin et al. 2017) to account for study/protocol effects while preserving variance associated with age and diagnosis. This design allows a larger cross‐sectional comparison of cortical thickness and R1 between FXS and idiopathic ASD while explicitly addressing multi‐protocol measurement differences.

## 2 Methods

### 2.1 Participants

Sixty‐one male participants (11 FXS, 50 ASD), ages 9–18 years, were included in these analyses. The original version of the study included 25 participants (11 FXS, 14 ASD; original study name: FX‐ASD). In order to increase statistical power, 36 ASD participants were added from two existing studies of ASD and neurotypical development (labeled as the HMN studies, Table 1). Table 1 shows the mean age, IQ, and ADOS‐2 Comparison Scores for the participants. ASD participants had a score of at least 5, indicating moderate to severe behavioral symptoms of ASD. Six of the 11 participants with FXS also had a moderate score (5–7), one had a severe score (8–10), while the rest had low scores. For qualitative comparisons, data from 46 typically developing (TD) participants in the HMN studies were plotted alongside the FXS and combined ASD groups. However, to limit the number of multiple comparisons, no inferential statistics were conducted that included this typically developing group. All added participants were matched for age (9–18 years) and sex (males only) to the FX‐ASD study. Protocol differences were addressed via ComBat harmonization (see Data Harmonization/Statistical Analysis).

**TABLE 1:** Demographic information—Age, IQ, ADOS.

	Original study FXS (*n* = 11)	Original study ASD (*n* = 14)	HMN studies ASD (*n* = 36)	HMN studies TD (*n* = 46)
**Age**				
Mean (SD)	15.40 (2.75)	13.89 (3.03)	13.50 (3.04)	12.17 (2.90)
Range	9.60–18.48	9.42–18.98	9.03–17.95	8.95–17.95
**IQ**				
Mean (SD)	54.73 (27.20)	95.38 (23.90)	103.00 (17.59)	113.74 (14.09)
**ADOS‐2 Comparison Score**				
Mean (SD)	5.27 (2.28)	7.71 (1.68)	7.03 (2.53)	NA

*Note*: ADOS = Autism Diagnostic Observation Schedule. This table depicts the mean age, IQ, and ADOS‐2 Comparison Score of the participants. ADOS‐2 Comparison Score is out of 10, with scores of 5–7 indicating moderate behavioral symptoms of ASD, and 8–10 representing a severe presence. Note, only 33/36 ADOS scores were available for HMN ASD. In the case of missing scores, ASD was validated through the use of the Autism Diagnostic Interview–Revised. Only 13/14 IQ scores were available for the original study ASD. The IQ substests used were as follows: 4‐subtest and 2‐subtest WASI‐II FSIQ and KBIT‐2 for HMN studies, Leiter third edition (subtests: Figure Ground, Form Completion, Classification and Analogies, Sequential Order) for the original study.

### 2.2 Data Harmonization

In order to account for differences in imaging protocols across studies, non‐parametric data harmonization using ComBat (Richter et al. 2022) was employed. This harmonization technique accounts for study‐specific additive (on the bias) and multiplicative (on the variance) effects on the MRI data parameter maps (or other outcomes) using a linear model. These correction effects are the expected values of the empirically estimated Bayesian prior distributions either parametrically via expectation maximization or non‐parametrically via importance sampling (Fortin et al. 2017; Richter et al. 2022). Such a prior‐based approach is robust when there are small sample sizes per study since the study‐specific mean, variance, shape, and scale parameters are estimated using combined information across all voxels (or regions; Chu et al. 2023; Fortin et al. 2017; Richter et al. 2022).

### 2.3 MRI Acquisition and Processing

Participants underwent MPnRAGE (Kecskemeti et al. 2016) scanning on a 3T GE MR750 scanner. MPnRAGE data were obtained with a 3D radial acquisition sequence using sinusoidal gradients. For all studies, images were acquired at 1 mm isotropic spatial resolution, with 256 axial slices, and a 256 × 256 in‐plane acquisition matrix. For the FX‐ASD and HM studies, timing parameters were TR = 4.9 ms, TE = 1.8 ms, and TI = 12 ms. The inversion recovery curve was sampled with 386 views (*n*) split at 325 with 4‐degree flip angle and 61 with 8‐degree flip angle. For the *N* study, timing parameters were TR = 4.6 ms, TE = 1.8 ms, and TI = 12 ms. The inversion recovery curve was sampled with 386 views (*n*) split at 437 with 4‐degree flip angle and 112 with 6‐degree flip angle. These details are laid out in Table 2.

**TABLE 2:** Summary table—MPnRAGE acquisition and processing.

Parameter	FX–ASD & HM studies	*N* study
Scanner	3 T GE MR750	3 T GE MR750
Sequence	3D radial, sinusoidal gradients	3D radial, sinusoidal gradients
Spatial resolution	1 mm isotropic	1 mm isotropic
Number of slices	256 axial	256 axial
In‐plane matrix	256 × 256	256 × 256
TR (ms)	4.9	4.6
TE (ms)	1.8	1.8
TI (ms)	12	12
Total views (n)	386	549
Flip angle 1	325 views @ 4°	437 views @ 4°
Flip angle 2	61 views @ 8°	112 views @ 6°
Reconstruction	Retrospective, motion‐corrected	Retrospective, motion‐corrected
Outputs	T1‐weighted, qR1 maps	T1‐weighted, qR1 maps
Post‐processing	FreeSurfer *recon‐all*, Destrieux atlas	FreeSurfer *recon‐all*, Destrieux atlas

Motion‐corrected image reconstruction was performed retrospectively (Kecskemeti et al. 2018) and produced 3D anatomical T1‐weighted and qR1 (= 1/T1) maps. The T1‐weighted volumes were processed using the “*recon‐all*” routine part of FreeSurfer (Dale et al. 1999; Fischl et al. 2002) to produce cortex parcellations based on the Destrieux atlas (Destrieux et al. 2010) as well as cortical boundaries.

### 2.4 Statistical Analysis

Average qR1 and cortical thickness measures were extracted from 74 cortical regions per hemisphere of the Destrieux atlas. Data were harmonized across studies using ComBat (Fortin et al. 2017; Richter et al. 2022) with age and diagnosis (ASD, FXS, TD) as covariates and imaging protocol as the batch effect. Imaging data before and after harmonization are plotted by group, study, and age and are provided in Section 1 of the Supporting Information. As expected, cortical thickness measures appear to be minimally affected by protocol‐related differences (Supporting Information Section 1B). On the other hand, impacts of imaging protocol on qR1 are more evident as shown on the plots in Section 1A of the Supporting Information. These plots also indicate that those differences are adequately resolved by the data harmonization.

Subsequently, each measure was modeled linearly with age, group, and age‐by‐group terms. This analysis was done three times. First, using the original study data only (FX‐ASD). Second, comparing FXS to data from ASD participants in the added group. A third time, comparing FXS to ASD data from the original and added groups combined. False discovery rate (FDR) was used to control the number of false positives due to multiple comparisons.

## 3 Results

**FIGURE 1:**
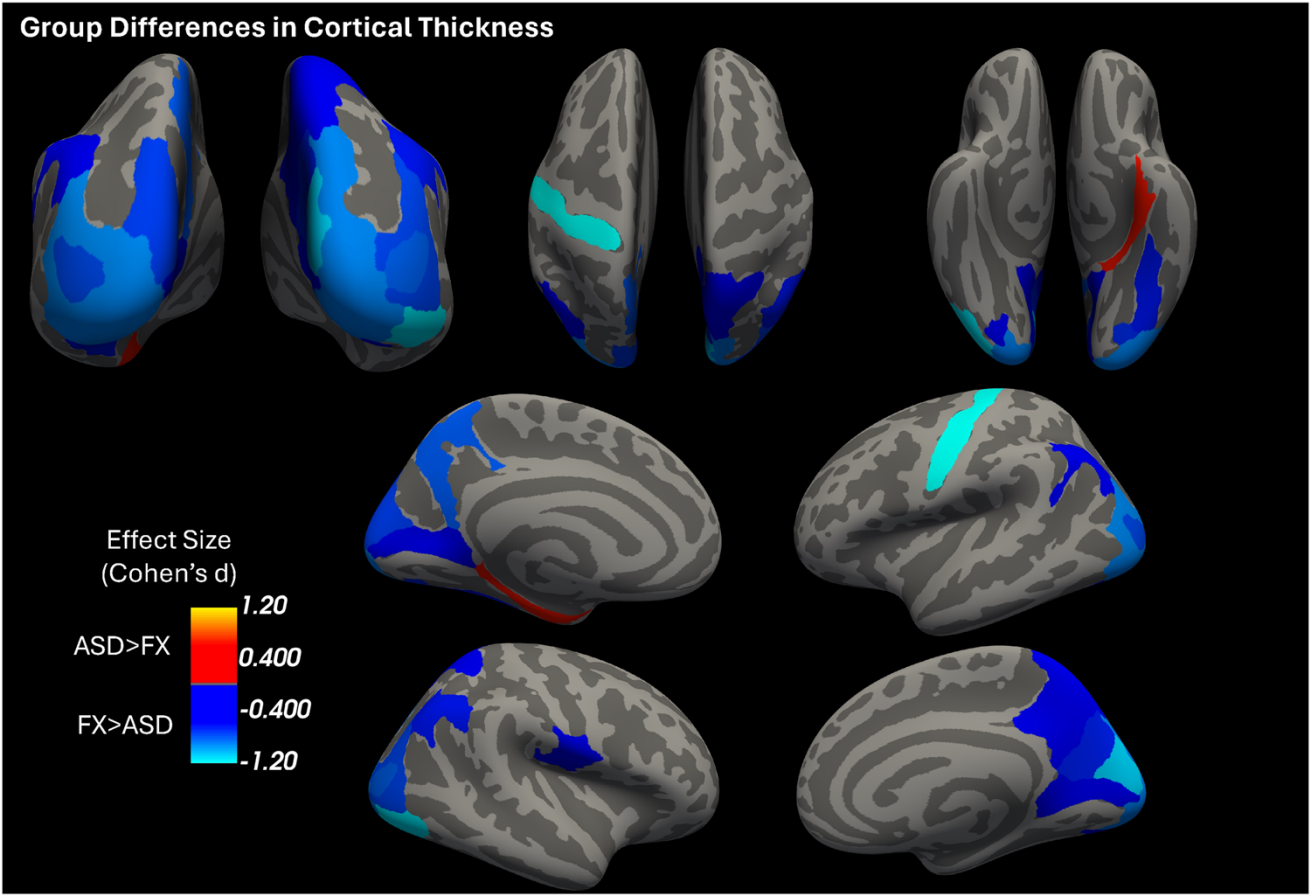
Significant group differences (*p* < 0.05, FDR corrected) in cortical thickness between FXS and ASD. These were found primarily within occipital, parietal, and temporal regions and are from the original FX data with combined ASD data from original and HMN studies.

**FIGURE 2:**
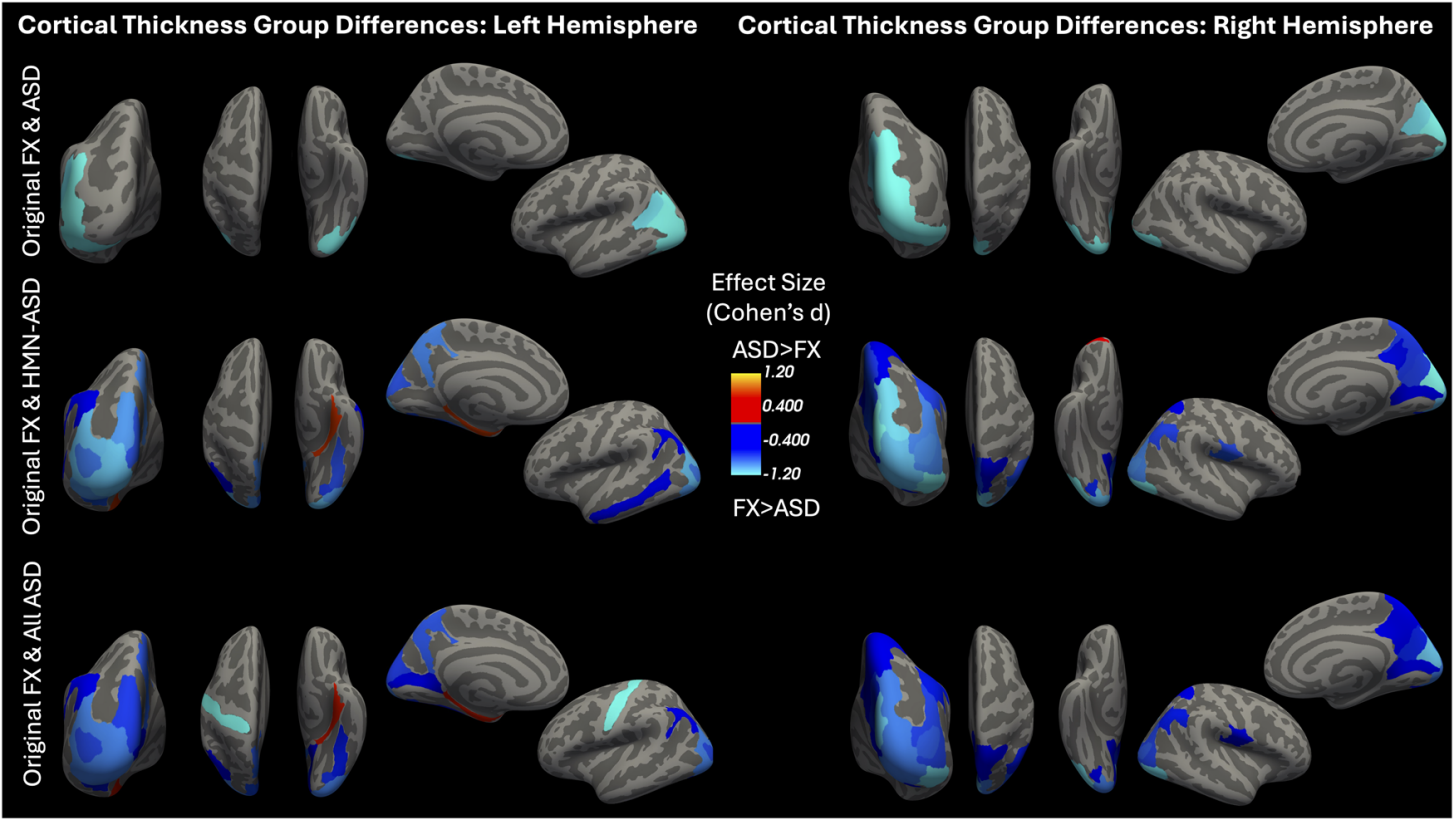
Cortical thickness differences between groups in each hemisphere. Top row: Data exclusively from original study (FX‐ASD). Middle row: Results from original FXS participants and from age‐matched added participants in HMN studies. Bottom row: Original FX data with combined ASD data from original and HMN studies.

Significant group differences in cortical thickness for FXS and ASD were found primarily within occipital, parietal, and temporal regions. Anatomical representations of these results are highlighted in Figures 1 and 2. Detailed statistical testing results are shown in Table S1 of the Supporting Information Section 2A.

As seen in Figure 2, the findings of significant group differences in several of these regions persist even for the smaller ASD sample size in the original dataset.

**FIGURE 3:**
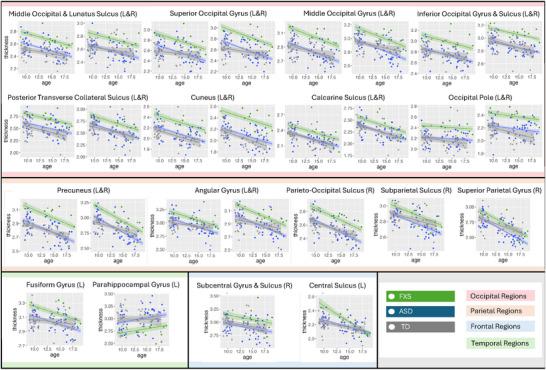
Developmental trajectories from regions where cortical thickness between the FXS and ASD groups was significantly different. This includes FXS and ASD data from the original as well as ASD and TD HMN studies. Age is in years. Thickness is in millimeters. TD: typically developing. R: right hemisphere. L: left hemisphere.

With the exception of the parahippocampal gyrus, all areas with significant differences indicate increased cortical thickness in the FXS group and an inverse relationship with age for both groups (Figure 3). Interestingly, in all these regions, the ASD group distributions appear more similar to those of the typically developing group.

No significant group differences in qR1 were found after FDR correction. However, the non‐FDR corrected qR1 data indicate some interesting trends (Supporting Information Section 2B). For example, in the bilateral precuneus, compared to autistic children, participants with FXS had increased cortical thickness but showed trending levels of decreased qR1. Also, in the left parahippocampal gyrus, which showed decreased cortical thickness in FXS, compared to autism, there is a trend for increased qR1 in FXS, compared to autism.

## 4 Discussion

The findings from our neuroimaging study reveal distinct patterns of cortical thickness alterations and mostly similar developmental trajectories between FXS and ASD in male children, providing important insights into the neurobiological underpinnings of these neurodevelopmental conditions. Differential patterns observed across visual‐processing, parietal, and limbic regions suggest disorder‐specific mechanisms of cortical maturation that may contribute to the characteristic phenotypes of each condition.

### 4.1 Enhanced Visual‐Processing Cortical Thickness in FXS

Individuals with FXS show statistically increased cortical thickness across multiple visual‐processing regions, including the occipital gyri, calcarine sulcus, cuneus, and occipital pole, suggesting alterations in visual cortex development that may underlie the sensory processing features that are characteristic of this condition (Croom et al. 2024; Rahmatullah et al. 2023). The calcarine sulcus, which houses the primary visual cortex (V1), undergoes significant developmental changes during gestation and early postnatal life (Li et al. 2024a, 2024b). The increased cortical thickness in FXS may reflect altered pruning processes or delayed maturation of visual cortical circuits.

This finding aligns with evidence from animal models of FXS, where sensory hypersensitivity and altered temporal processing have been documented (Croom et al. 2023, 2024). The Fragile X Messenger Ribonucleoprotein (FMRP) plays crucial roles in synaptic plasticity and dendritic spine development (Ibenbrahim et al. 2022; Kuznitsov‐Yanovsky et al. 2022), and its absence in FXS leads to disrupted protein synthesis regulation at synapses (Sidorov et al. 2013). The increased cortical thickness in visual regions may reflect impaired synaptic pruning mechanisms, as FMRP normally regulates the translation of proteins essential for synaptic refinement and elimination of inappropriate connections (Kennedy et al. 2020).

### 4.2 Parietal‐Occipital Network Alterations and Spatial Processing

The extensive involvement of parietal regions, including the precuneus, angular gyrus, superior parietal gyrus, and parieto‐occipital sulcus, in the FXS group increased cortical thickness suggests alterations in the dorsal visual stream and spatial processing networks. These regions are critical components of the default mode network (DMN) and are involved in visuospatial attention, spatial working memory, and self‐referential processing (Bathelt and Geurts 2021; Lee et al. 2022). The precuneus, in particular, serves as a hub for integrating information across brain networks and is essential for consciousness and self‐awareness (Xiao et al. 2023).

The increased cortical thickness in these regions in FXS may contribute to the altered spatial processing and attention difficulties observed in this condition. Research has shown that individuals with FXS exhibit differences in attention and are particularly susceptible to distractors (Rahmatullah et al. 2023), which may be related to structural alterations in these attention‐related parietal regions. The finding that autistic individuals showed more typical cortical thickness values in these regions relative to typically developing controls suggests that the spatial processing difficulties in FXS may have distinct neural substrates from those in autism.

### 4.3 Parahippocampal Gyrus: Memory and Spatial Navigation

Interestingly, the parahippocampal gyrus was the only region where the autism group showed statistically increased cortical thickness compared to the FXS group, and both groups demonstrated positive age relationships in this region. The parahippocampal gyrus is essential for memory formation, spatial navigation, and episodic memory consolidation (Huntgeburth and Petrides 2012; Imai et al. 2023; Kang et al. 2022). This region is part of the medial temporal lobe memory system and shows altered structure and function in various neurodevelopmental and psychiatric conditions (Fernández et al. 2020; Imai et al. 2023).

The positive relationship between age and cortical thickness in this region for both groups, contrasting with the inverse relationships observed elsewhere, suggests active developmental processes continuing through adolescence (Ducharme et al. 2016; Kelly et al. 2024). The differential thickness patterns in the parahippocampal gyrus between FXS and ASD may reflect distinct approaches to memory processing and spatial navigation in these conditions. In ASD, the increased thickness in this region might be associated with the enhanced local processing and attention to detail that characterizes the condition, while the relatively thinner cortex in FXS may contribute to the memory and learning difficulties commonly observed in this syndrome (Prieto et al. 2021; Sidorov et al. 2013). This, given that in FXS, memory deficits may be more directly related to the absence of FMRP and its effects on synaptic plasticity in hippocampal circuits (Sidorov et al. 2013).

### 4.4 Cross‐Sectional Age‐Related Associations

In this cross‐sectional sample, in both groups, cortical thickness was negatively associated with age in most of the regions with significant group differences. This pattern is consistent with prior developmental neuroimaging work showing that, on average, many cortical regions exhibit age‐related thinning across late childhood and adolescence, often attributed to multiple co‐occurring maturational processes (e.g., synaptic remodeling and increasing intracortical myelination; Bieneck et al. 2021; You et al. 2024). However, because our data are not longitudinal, these associations should be interpreted as between‐subject age relationships rather than within‐individual developmental trajectories.

The significant age‐by‐group interaction in the central sulcus, with a stronger inverse relationship in the FXS group, suggests altered cross‐sectional age‐related associations in sensorimotor regions. The central sulcus contains the primary motor and somatosensory cortices, and the stronger age‐related thinning in FXS may reflect accelerated or dysregulated pruning processes in these regions. This finding is consistent with the motor coordination difficulties and sensory processing differences commonly observed in FXS (Croom et al. 2024; Hourani and Pouladi 2024). The convergence of cortical thickness values between groups at older ages suggests that some aspects of cortical development may eventually normalize, though functional differences may persist. Again, longitudinal follow‐up would be required to determine whether this reflects true differences in within‐individual rates of change, differences in the timing of maturational processes, or cohort‐related factors.

### 4.5 Absence of qR1 Differences

The longitudinal magnetization relaxation rate, qR1, is sensitive to myelin content and shows developmental changes during infancy and early childhood (Grotheer et al. 2022; Kühne et al. 2021). In the present study, we did not observe significant group differences in cortical qR1 after correction for multiple comparisons. This null result is nevertheless informative in the context of the robust cortical thickness differences observed because it suggests that the macrostructural group effects captured by cortical thickness are not accompanied by large, region‐averaged differences in qR1 across the cortex within this late childhood–adolescent age range.

Importantly, the absence of significant qR1 differences should not be interpreted as evidence that myelination‐related biology is irrelevant to FXS or ASD. As previously indicated, post‐mortem work in ASD indicates that aberrant myelination in gray matter is among the more consistent histological findings (Fetit et al. 2021), and R1 is a myelin‐sensitive quantitative MRI marker with reported potentially biophysical grounding (Koenig 1991; Mottershead et al. 2003; Shafee et al. 2015; Sprooten et al. 2019; Turner 2019). Several non‐mutually exclusive explanations could account for our null qR1 finding despite cortical thickness differences. First, group differences in intracortical myelin may be spatially focal (restricted to specific cortical areas), subtle in magnitude, or heterogeneous across individuals, reducing power to detect region‐averaged effects in a modest cross‐sectional sample. Second, qR1, while myelin‐sensitive, is not myelin‐specific and can also vary with other tissue factors and microstructural composition, which may dilute group contrasts when averaged over large cortical regions. Third, the relationship between cortical thickness and myelin may be depth‐dependent rather than uniform across the cortical ribbon. Deeper cortical layers are expected to show stronger positive associations with myelin content, whereas superficial layers may not follow the same relationship (Shafee et al. 2015; Sprooten et al. 2019). As a result, region‐averaged cortical qR1 may fail to capture layer‐resolved differences that could coexist with robust thickness effects.

Our findings also differ from animal work reporting delayed myelination in FXS models (Pacey et al. 2013), which could reflect species differences, developmental timing, or the possibility that any myelination delays are more prominent earlier in development and may partially normalize by late childhood/adolescence. Finally, although MPnRAGE enables robust whole‐brain qR1 mapping, the spatial resolution and region‐averaging approach used here may still be insufficient to detect depth‐resolved intracortical myelin differences. Future studies that incorporate longitudinal designs, larger samples, and higher‐resolution or depth‐resolved approaches to intracortical myelin (or complementary quantitative contrasts) will be important for determining whether sensory‐cortical thickness differences between FXS and ASD are accompanied by more subtle or layer‐specific myelination‐related alterations.

### 4.6 Mechanistic Considerations and FMRP Function

The observed cortical thickness alterations in FXS can be understood within the context of FMRP's role in neural development. FMRP is a translational repressor that regulates the synthesis of proteins crucial for synaptic development, maintenance, and plasticity (Edwards et al. 2022; Kuznitsov‐Yanovsky et al. 2022). In the absence of FMRP, there is dysregulated protein synthesis leading to altered dendritic spine morphology, increased spine density, and immature spine characteristics (Ibenbrahim et al. 2022; Sunamura et al. 2018).

The increased cortical thickness observed in multiple brain regions in FXS may reflect the accumulation of immature or aberrant synapses due to impaired pruning mechanisms (Castrén 2016; Telias et al. 2015). During normal development, synaptic pruning eliminates weak or inappropriate connections, leading to cortical thinning. The disruption of this process in FXS could result in the retention of excess synapses and increased cortical thickness, particularly in regions where FMRP normally plays important regulatory roles.

### 4.7 Implications for Understanding Autism–Fragile X Relationships

While autism and FXS share many clinical features and FXS is considered a leading monogenetic cause of autism spectrum behaviors, the distinct patterns of cortical thickness alterations observed in this study highlight important neurobiological differences between these conditions. The finding that autism cortical thickness values were generally more similar to typically developing controls suggests that the brain structural alterations in autism may be more subtle or involve different mechanisms than those in FXS in this cross‐sectional sample.

These differences may contribute to the distinct clinical presentations of the two conditions. For example, the enhanced visual‐processing cortical thickness in FXS may relate to the visual attention difficulties and sensory sensitivities characteristic of this condition, while the relative preservation of these regions in autism in combination with other factors may allow for the superior visual processing abilities sometimes observed in autism (Ring et al. 1999; Thérien et al. 2023).

### 4.8 Clinical and Therapeutic Implications

Understanding the distinct developmental trajectories and brain structural differences between FXS and autism has potentially important implications for intervention strategies. If replicated in larger and longitudinal cohorts, age‐dependent patterns of group differences may help identify developmental windows during which imaging markers are most informative for linking brain features to symptom profiles and treatment response. However, the present cross‐sectional design does not allow inferences about within‐individual critical periods or causal timing.

Furthermore, the distinct patterns of brain involvement suggest that therapeutic approaches may need to be tailored to the specific neurobiological profile of each condition. For FXS, interventions targeting sensory processing and visual attention might focus on the altered occipital and parietal networks, while approaches for autism might need to consider different patterns of brain involvement.

### 4.9 Comparison With Prior FXS–ASD Neuroimaging Studies

Direct neuroimaging comparisons of FXS and ASD are relatively sparse, but some existing work as mentioned in the Introduction suggests potentially dissociable neuroanatomical profiles may exist despite overlapping behavioral features. To recapitulate, in their VBM study of adolescents and adults, Wilson et al. (2009) reported relative gray‐matter reductions in insular, prefrontal, and cerebellar regions in FXS, whereas ASD showed enlargements in medial temporal and parietal areas. In toddlers, Hazlett et al. (2009) observed smaller temporal–limbic volumes and larger caudate nuclei in FXS compared with ASD despite similar autism symptom levels. Using a surface‐based approach in toddler boys, Hoeft et al. (2011) reported widespread cortical thinning in prefrontal and superior temporal association areas in FXS contrasted with focal thickening in ASD, along with opposing volumetric patterns within amygdala–insula circuitry. More recently, Shen et al. (2022) demonstrated age‐ and disorder‐specific subcortical developmental patterns across infancy, further supporting the notion that FXS and ASD may follow separable neurodevelopmental profiles. In this context, our results extend prior work by focusing on late childhood to adolescence (9–18 years) using motion‐robust quantitative MRI and surface‐based cortical thickness estimation. The predominant group differences we observed were localized to early sensory cortices (occipital and parietal regions and related visual‐processing areas), which differ from the fronto‐temporal association and salience‐network emphasis in toddler samples (Hoeft et al. 2011) and from VBM findings that reflect composite morphometric properties (Wilson et al. 2009). Importantly, rather than contradicting prior reports, our findings provide complementary evidence that neuroanatomical distinctions between FXS and ASD may manifest in different systems and depend on developmental stage (e.g., age range) and imaging measurement type (VBM/volumetry vs. surface‐based thickness), as well as methodological factors affecting pediatric imaging quality.

### 4.10 FXS Sample Heterogeneity

Given the rage of ADOS‐2 comparison scores observed within the FXS cohort, we conducted an exploratory analysis to evaluate whether cortical thickness differences within FXS could be related to autism symptom severity. We stratified the FXS group into low (ADOS‐2 CS <5; *n* = 4) and moderate‐to‐high (ADOS‐2 CS ≥5; *n* = 7) subgroups. We then modeled cortical thickness as a function of FXS severity subgroup and age within the set of regions that showed significant FXS‐ASD differences in the primary analysis. Two regions, the left fusiform gyrus and the right superior occipital gyrus, had nominal significant differences with lower cortical thickness in the “moderate‐to‐high” subgroup. However, no effects survived FDR correction for multiple comparisons (see Supporting Information Section 3A). These results should be interpreted cautiously, given the small subgroup size and limited statistical power, and they do not provide strong evidence that the primary FXS‐ASD cortical thickness effects are driven by a severity‐defined subset within the FXS cohort. Nevertheless, the variability in ADOS‐2 CS underscores the importance of improved phenotyping and larger samples in future studies to more rigorously test whether autism symptom dimensions within FXS map onto distinct neuroanatomical patterns.

### 4.11 Future Directions and Limitations

While these findings provide valuable insights into the neurobiological differences between FXS and autism, several important considerations should guide future research. The sample size, while adequate for detecting the observed differences, would benefit from expansion to allow for more detailed subgroup analyses and to examine potential sex differences, as this study included only male participants. We pooled additional ASD participants from two related MPnRAGE studies to increase power, and protocol differences were addressed via ComBat harmonization. Although ComBat reduces protocol‐related bias, residual protocol effects are possible. We therefore present pre/post‐harmonization measures (Supporting Information) and report that thickness was minimally protocol‐sensitive, whereas qR1 showed clearer protocol effects that were mitigated after harmonization.

Additionally, longitudinal studies following the same individuals over time would provide more definitive evidence about developmental trajectories and may help identify predictive biomarkers for clinical outcomes. The integration of cortical thickness measures with other neuroimaging modalities, such as functional connectivity and diffusion‐weighted imaging, could provide a more comprehensive understanding of how structural alterations relate to functional differences.

Further, a key interpretive consideration is that the ASD group represents a behaviorally defined, etiologically heterogeneous population (i.e., “idiopathic/non‐syndromic ASD”), while FXS, despite having a single known genetic etiology, also shows marked phenotypic variability. Therefore, observed group differences should not be interpreted as mapping onto a single causal mechanism for ASD or FXS nor as diagnostic biomarkers. Instead, they reflect average differences between two heterogeneous groups. Unmeasured variation in clinical profiles including cognitive ability, symptom severity, comorbidities, or medication use and etiologic factors within the ASD group may contribute to variability in cortical measures and attenuate effect sizes. It should also be noted that ASD participants in this study did not undergo fragile X genetic testing to rule out a comorbidity, but ASD individuals with a previously known diagnosis of FXS were excluded. Future work that incorporates deeper phenotyping and etiologic stratification such as genetic findings, comorbidity subgroups, and dimensional symptom measures will be important for identifying which subgroups or symptom dimensions are most strongly associated with the neuroanatomical patterns reported here.

Still, the current findings contribute to our understanding of the neurobiological bases of FXS and autism, highlighting both shared and distinct features of these neurodevelopmental conditions. The patterns of cortical thickness alterations and their cross‐sectional age‐related associations provide insights into the mechanisms underlying the clinical phenotypes and may inform future therapeutic approaches tailored to the specific brain differences observed in each condition.

## Author Contributions

Jose M. Guerrero‐Gonzalez, Anna K. Lowe, Andrew L. Alexander, and Audra M. Sterling contributed to the conceptualization, investigation, validation, and writing of the original draft. Jose M. Guerrero‐Gonzalez, Anna K. Lowe, Steven R. Kecskemeti, Andrew L. Alexander, and Audra M. Sterling contributed to methodology; and Jose M. Guerrero‐Gonzalez, Anna K. Lowe, and Steven R. Kecskemeti contributed to software. Jose M. Guerrero‐Gonzalez, Anna K. Lowe, and Brittany G. Travers contributed to data curation; Jose M. Guerrero‐Gonzalez, Anna K. Lowe, Brittany G. Travers, and Andrew L. Alexander contributed to formal analysis; and Jose M. Guerrero‐Gonzalez and Anna K. Lowe contributed to visualization. Jose M. Guerrero‐Gonzalez, Brittany G. Travers, Andrew L. Alexander, and Audra M. Sterling provided supervision, while Brittany G. Travers, Andrew L. Alexander, and Audra M. Sterling contributed to funding acquisition, project administration, and resources. Jose M. Guerrero‐Gonzalez, Anna K. Lowe, Steven R. Kecskemeti, Brittany G. Travers, Andrew L. Alexander, and Audra M. Sterling contributed to writing the review and editing.

## Funding

This work was supported by the NIDCD K23 DC016639 (AMS), NICHD P50 HD105353 (Waisman Center Core Grant), NICHD R01 HD108868, Hartwell Foundation Individual Biomedical Award (BGT), Brain and Behavior Research Foundation NARSAD Young Investigator Award (BGT), Eunice Kennedy Shriver National Institute of Child Health and Human Development R01 HD094715 (BGT).

## Ethics Statement

The study protocols were approved by the University of Wisconsin‐Madison Institutional Review Board, and data were collected following written informed consent.

## Conflicts of Interest

The authors declare no conflicts of interest.

## Supporting information




**Supplementary Materials**: brb371375‐sup‐0001‐SuppMat.pdf

## Data Availability

The data that support the findings of this study are available on request from the corresponding author. The data are not publicly available due to privacy or ethical restrictions.
