# Supplementary material for: Cerebral Cortex Morphometry and Relaxometry in Male Children With Fragile X Syndrome and Autism
Source: Brain Behav. 2026 Apr 30;16(5):e71375. doi: 10.1002/brb3.71375 (PMC13133409; doi:10.1002/brb3.71375)
Supplement: Supplementary file 1 — Supplementary Materials: brb371375‐sup‐0001‐SuppMat.pdf [file BRB3-16-e71375-s001.pdf]

Supplement

1A. Cortical Thickness Data Harmonization
Average cortical thickness measures were extracted from 74 cortical regions per hemisphere of the Destrieux atlas. Data were harmonized across studies using ComBat (Fortin et al., 2017; Richter et al., 2022) with age and diagnosis (ASD, FXS, TD) as covariates and imaging protocol as batch effect. Below, imaging data before and after harmonization are plotted by group, study, and age.

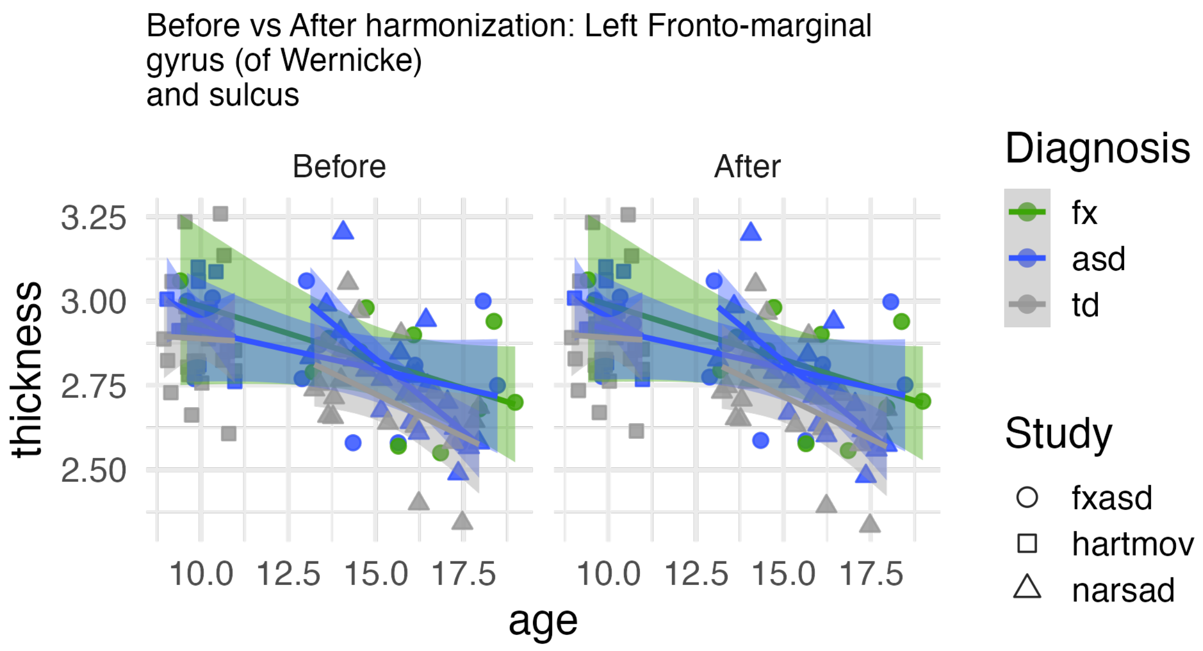

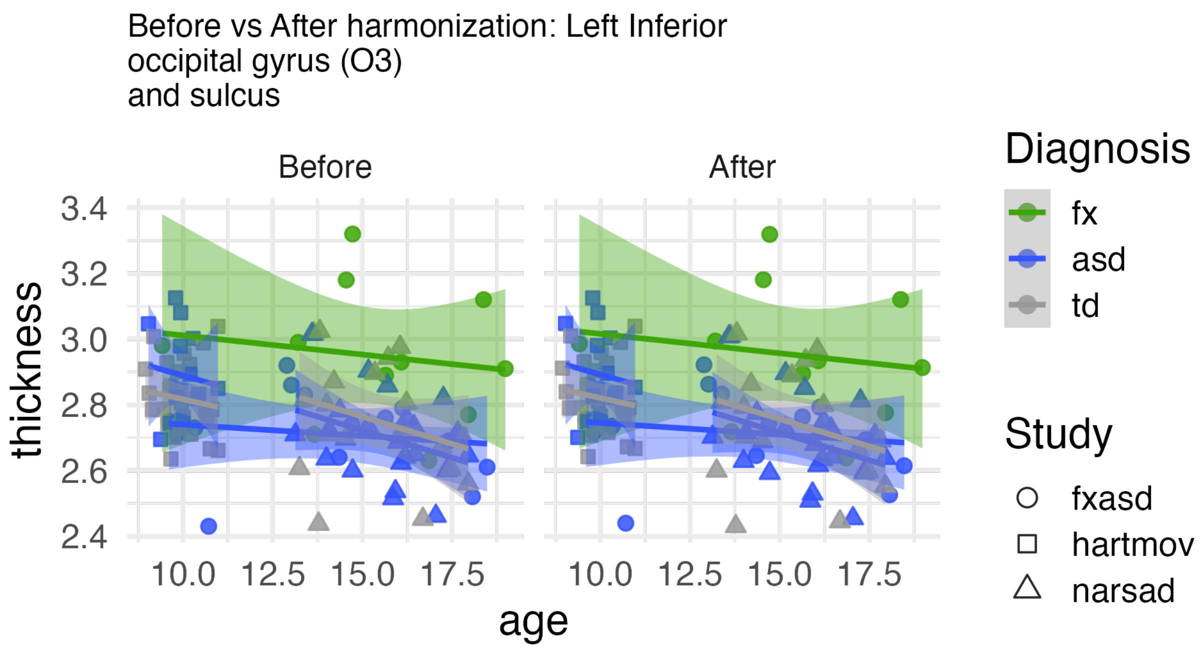

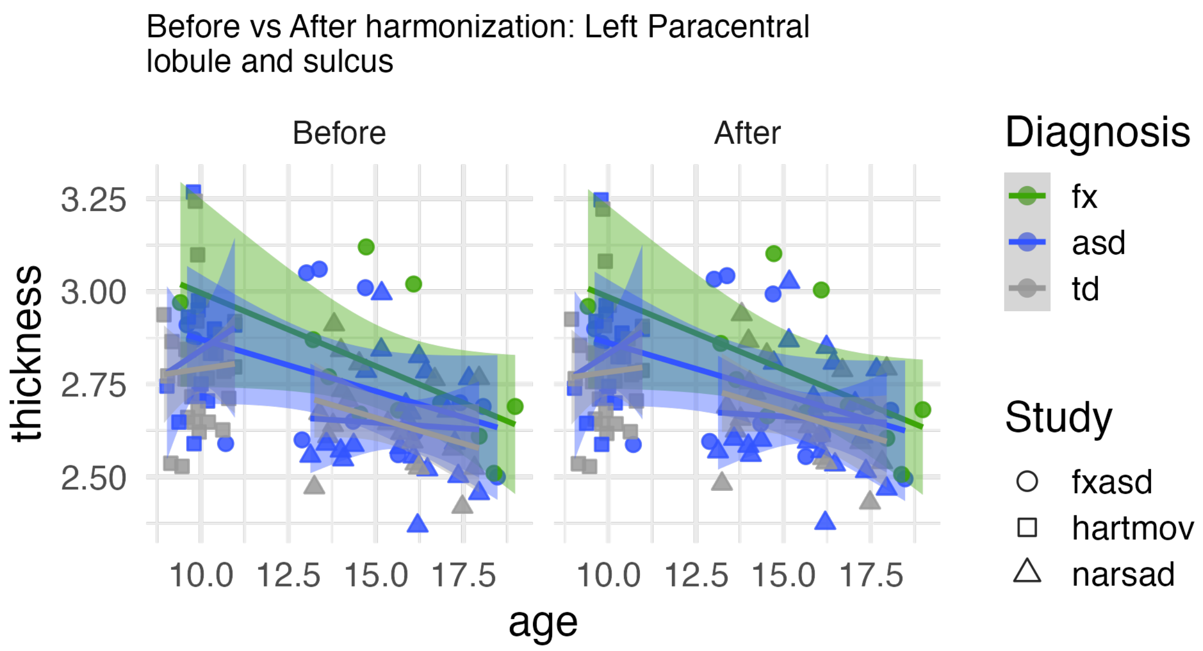
Before vs After harmonization: Left Paracentral
lobule and sulcus
Before
After
thickness
3.25
3.00
2.75
2.50
10.0
12.5
15.0
17.5
age
Diagnosis
fx
asd
td
Study
fxasd
hartmov
narsad

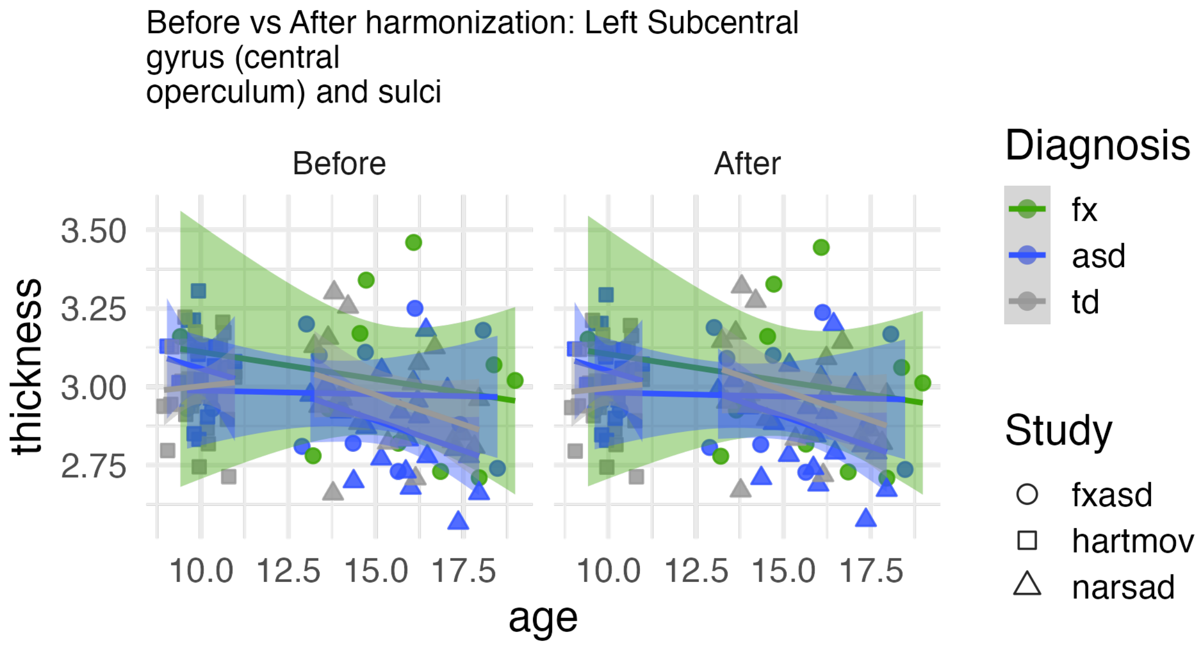
Before vs After harmonization: Left Subcentral
gyrus (central
operculum) and sulci
Before
After
thickness
3.50
3.25
3.00
2.75
10.0
12.5
15.0
17.5
age
Diagnosis
fx
asd
td
Study
fxasd
hartmov
narsad

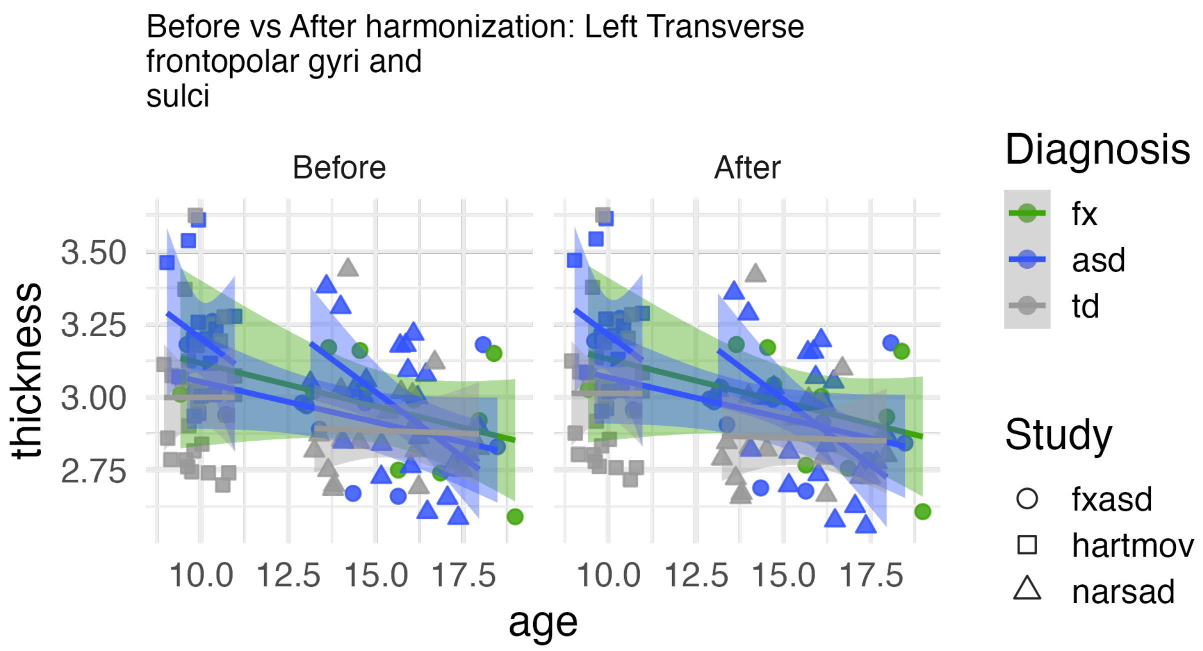
Before vs After harmonization: Left Transverse
frontopolar gyri and
sulci
Before
After
thickness
3.50
3.25
3.00
2.75
10.0
12.5
15.0
17.5
age
Diagnosis
fx
asd
td
Study
fxasd
hartmov
narsad

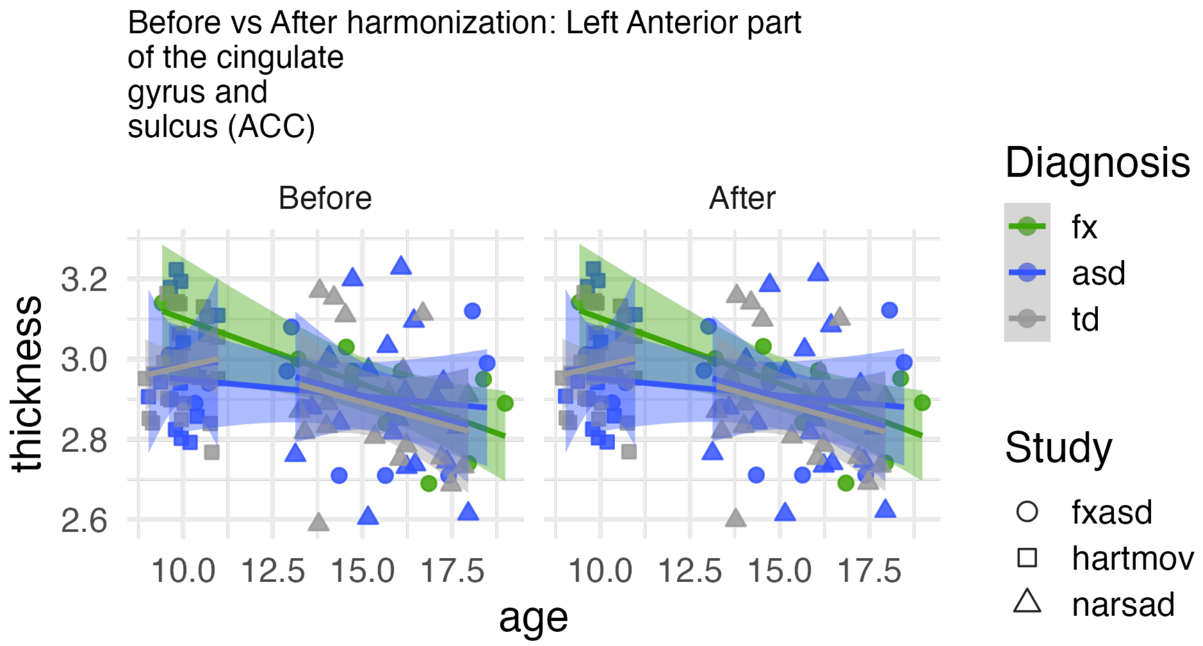
Before vs After harmonization: Left Anterior part
of the cingulate
gyrus and
sulcus (ACC)
Before
After
thickness
3.2
3.0
2.8
2.6
10.0
12.5
15.0
17.5
age
Diagnosis
fx
asd
td
Study
fxasd
hartmov
narsad

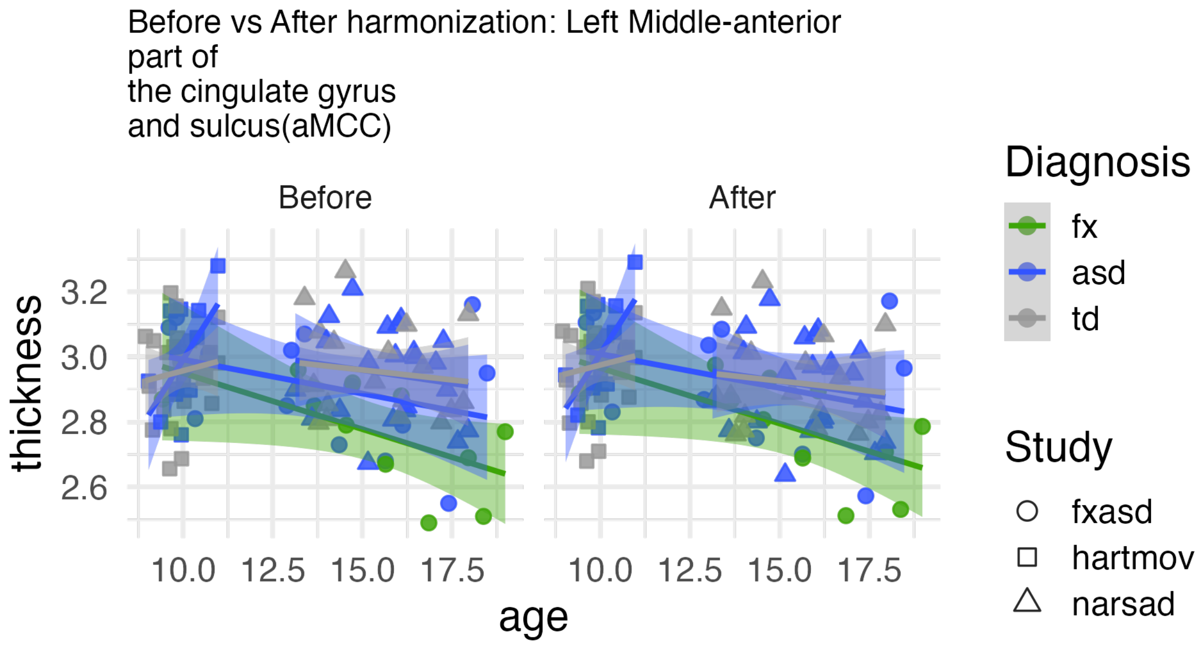
Before vs After harmonization: Left Middle-anterior
part of
the cingulate gyrus
and sulcus(aMCC)
Before
After
thickness
3.2
3.0
2.8
2.6
10.0
12.5
15.0
17.5
age
Diagnosis
fx
asd
td
Study
fxasd
hartmov
narsad

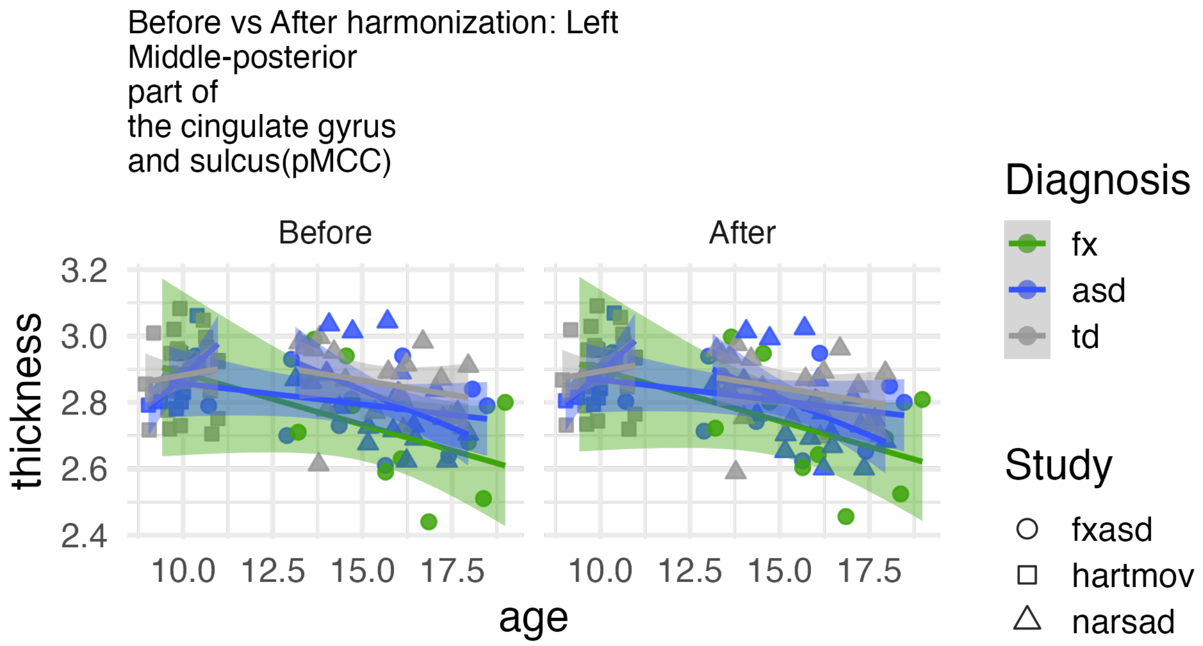
Before vs After harmonization: Left
Middle-posterior
part of
the cingulate gyrus
and sulcus(pMCC)
Before
After
thickness
3.2
3.0
2.8
2.6
2.4
10.0
12.5
15.0
17.5
age
Diagnosis
fx
asd
td
Study
fxasd
hartmov
narsad

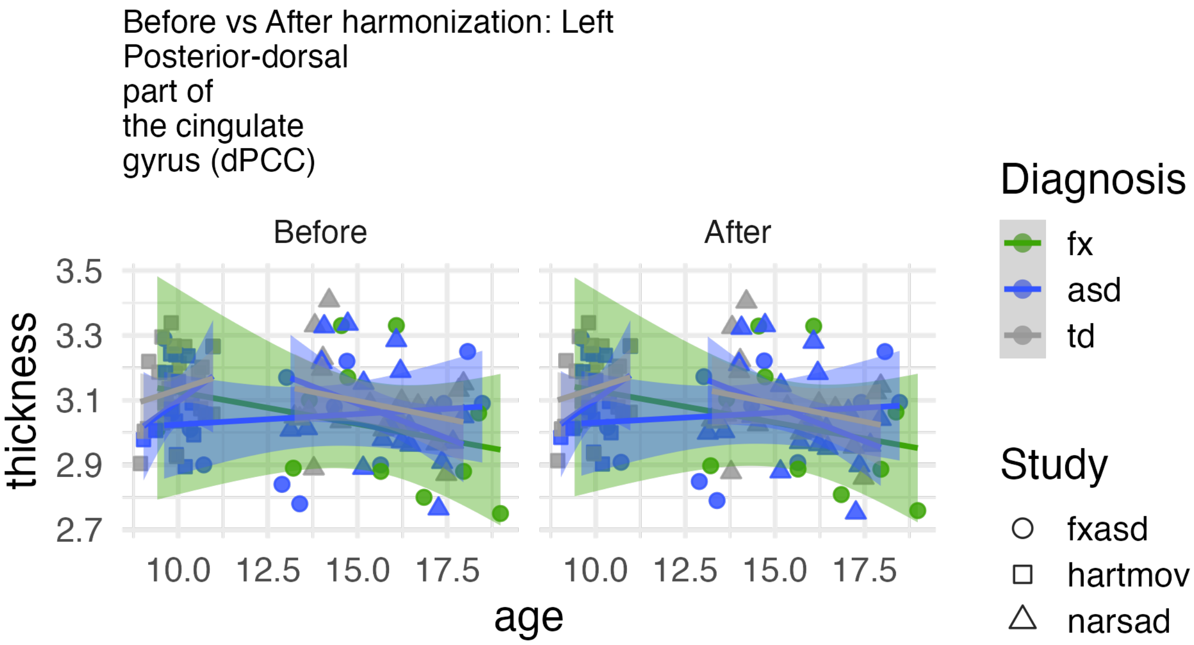
Before vs After harmonization: Left
Posterior-dorsal
part of
the cingulate
gyrus (dPCC)
Before
After
thickness
3.5
3.3
3.1
2.9
2.7
10.0
12.5
15.0
17.5
age
Diagnosis
fx
asd
td
Study
fxasd
hartmov
narsad

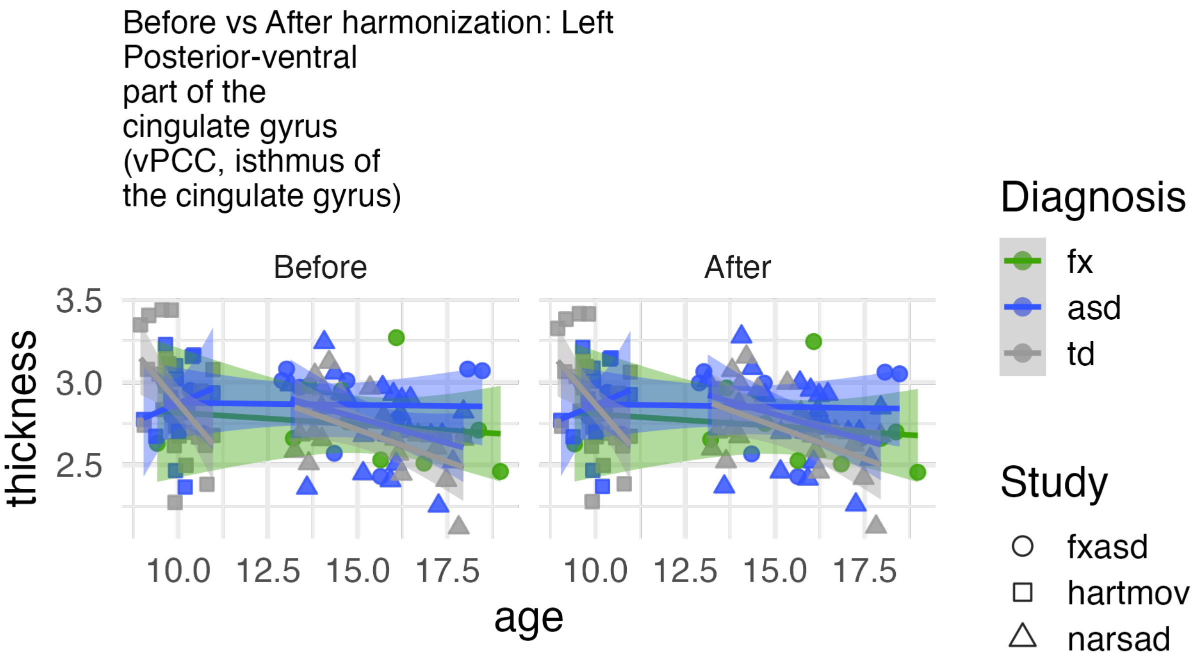
Before vs After harmonization: Left
Posterior-ventral
part of the
cingulate gyrus
(vPCC, isthmus of
the cingulate gyrus)
Before
After
thickness
3.5
3.0
2.5
10.0
12.5
15.0
17.5
age
Diagnosis
fx
asd
td
Study
fxasd
hartmov
narsad

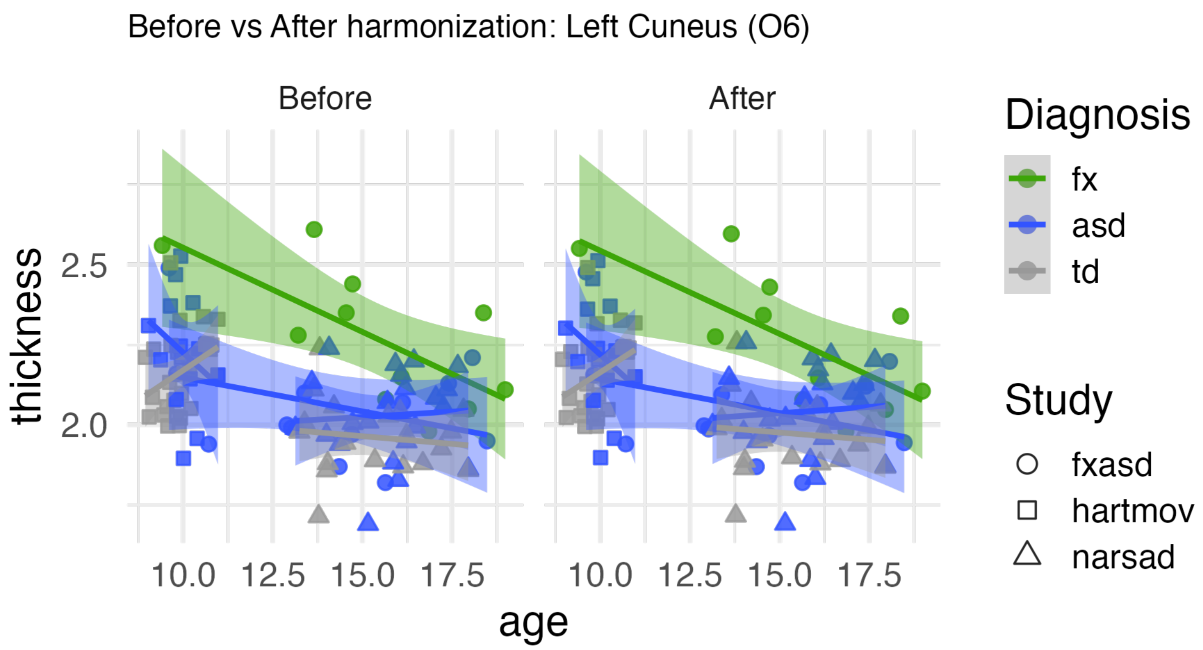

Before vs After harmonization: Left Cuneus (O6)
Before
After
thickness
2.5
2.0
10.0
12.5
15.0
17.5
age
Diagnosis
fx
asd
td
Study
fxasd
hartmov
narsad

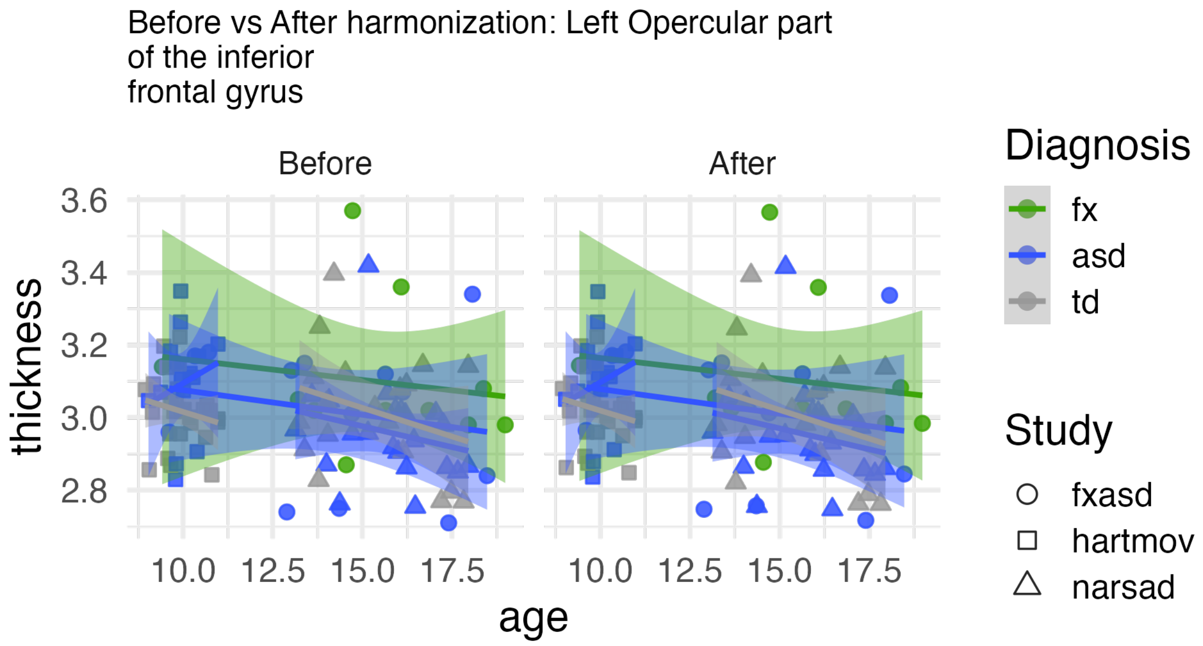

Before vs After harmonization: Left Opercular part of the inferior frontal gyrus
Before
After
thickness
3.6
3.4
3.2
3.0
2.8
10.0
12.5
15.0
17.5
age
Diagnosis
fx
asd
td
Study
fxasd
hartmov
narsad

Before vs After harmonization: Left Orbital part of
the inferior frontal
gyrus

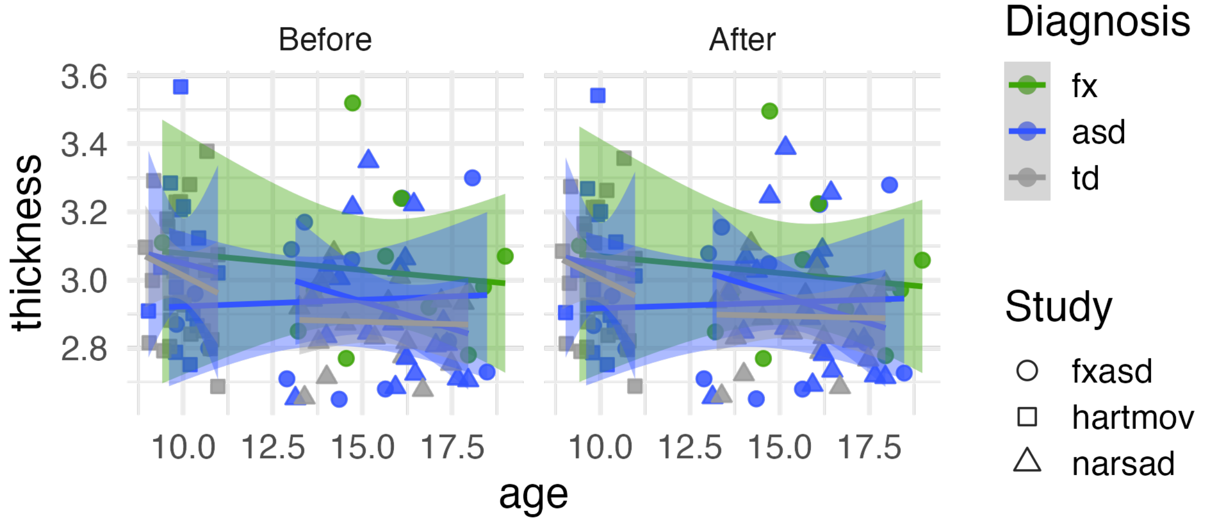

Before vs After harmonization: Left Triangular
part of the inferior
frontal gyrus

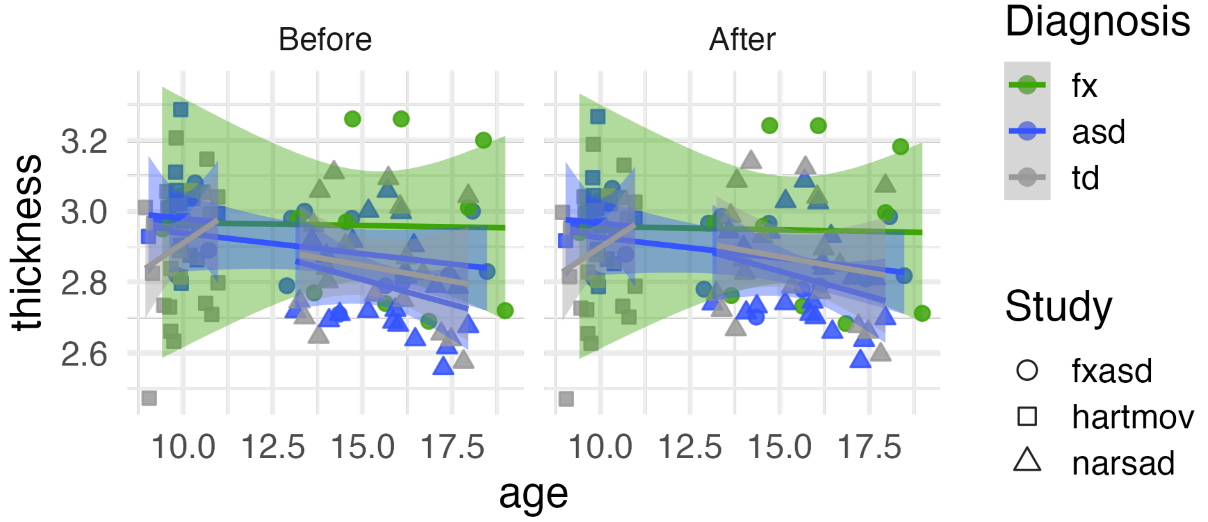

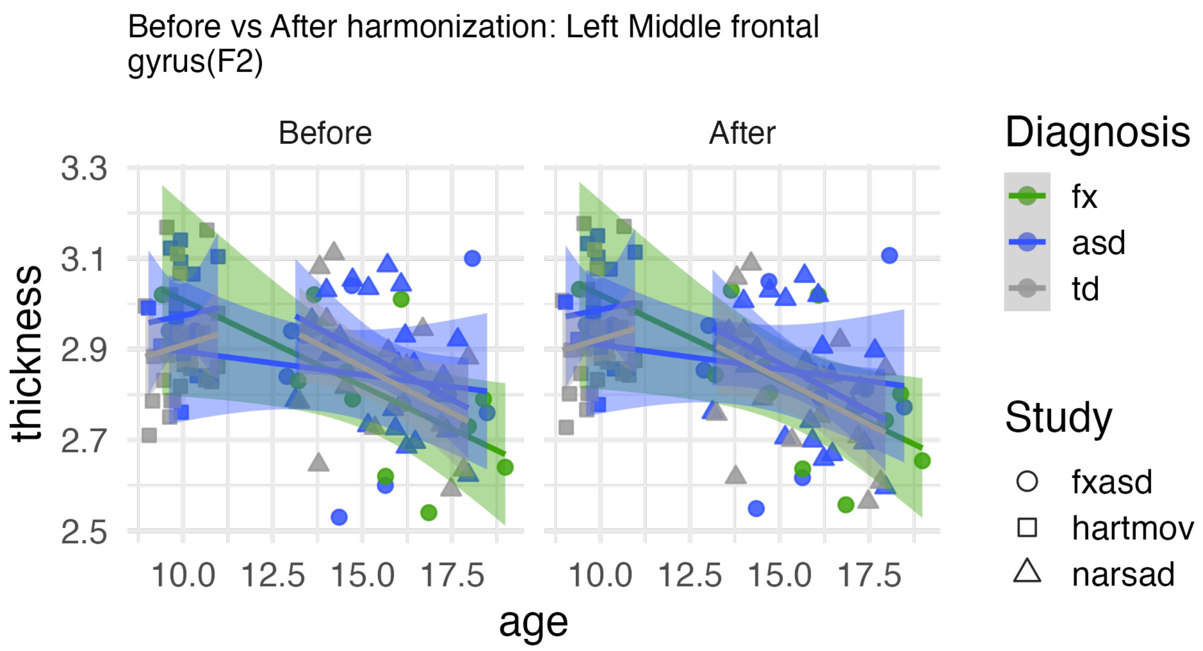

Before vs After harmonization: Left Middle frontal gyrus(F2)
Before
After
thickness
3.3
3.1
2.9
2.7
2.5
10.0
12.5
15.0
17.5
age
Diagnosis
fx
asd
td
Study
fxasd
hartmov
narsad

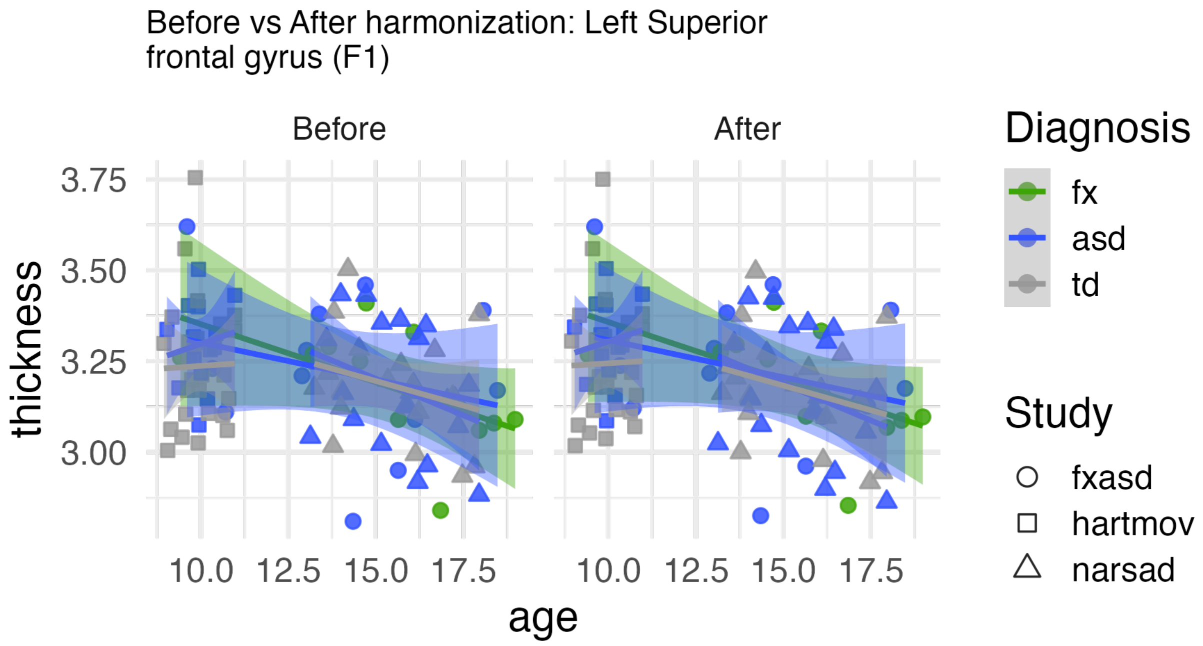

Before vs After harmonization: Left Superior frontal gyrus (F1)
Before
After
thickness
3.75
3.50
3.25
3.00
10.0
12.5
15.0
17.5
age
Diagnosis
fx
asd
td
Study
fxasd
hartmov
narsad

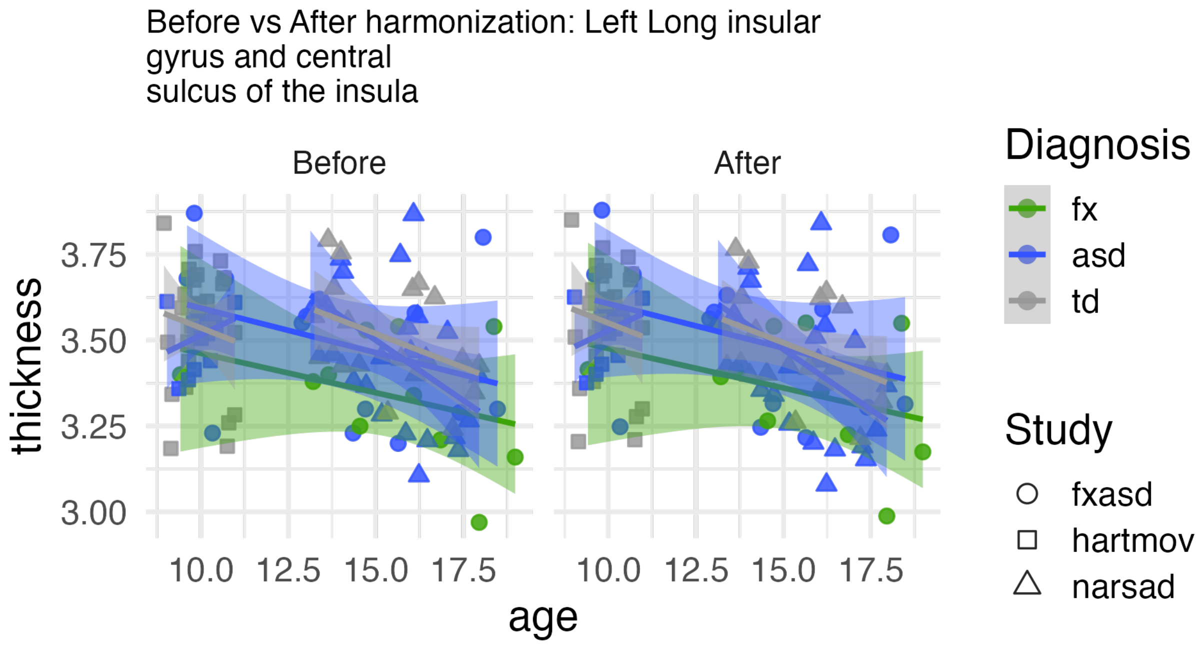
Before vs After harmonization: Left Long insular
gyrus and central
sulcus of the insula
Before
After
thickness
3.75
3.50
3.25
3.00
10.0
12.5
15.0
17.5
age
Diagnosis
fx
asd
td
Study
fxasd
hartmov
narsad

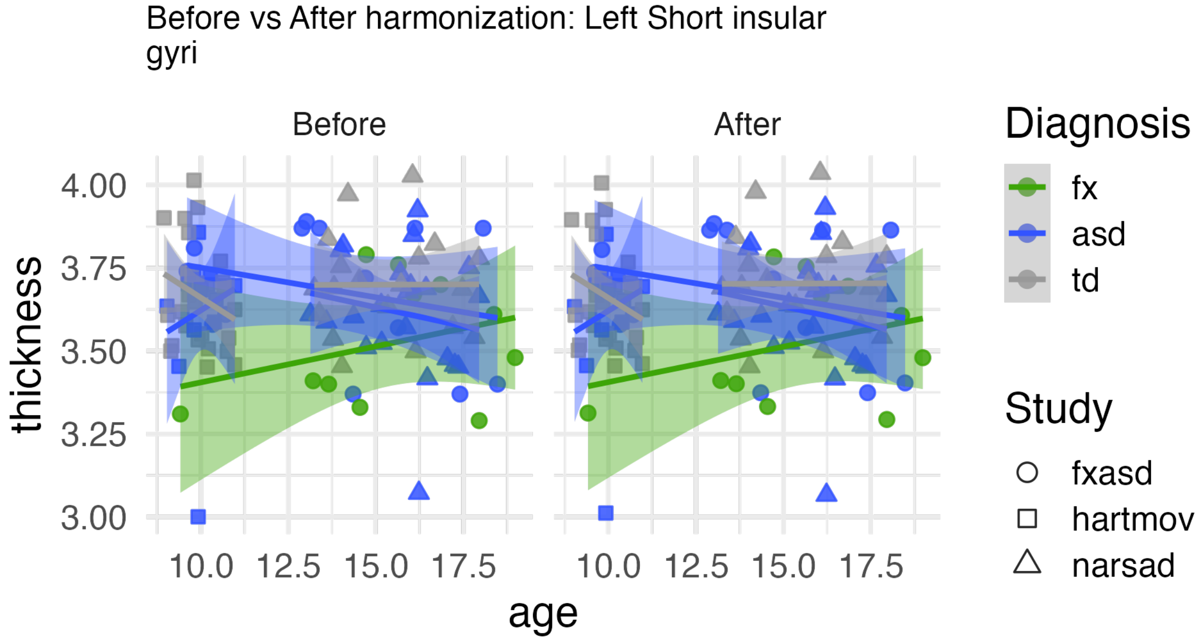
Before vs After harmonization: Left Short insular
gyri
Before
After
thickness
4.00
3.75
3.50
3.25
3.00
10.0
12.5
15.0
17.5
age
Diagnosis
fx
asd
td
Study
fxasd
hartmov
narsad

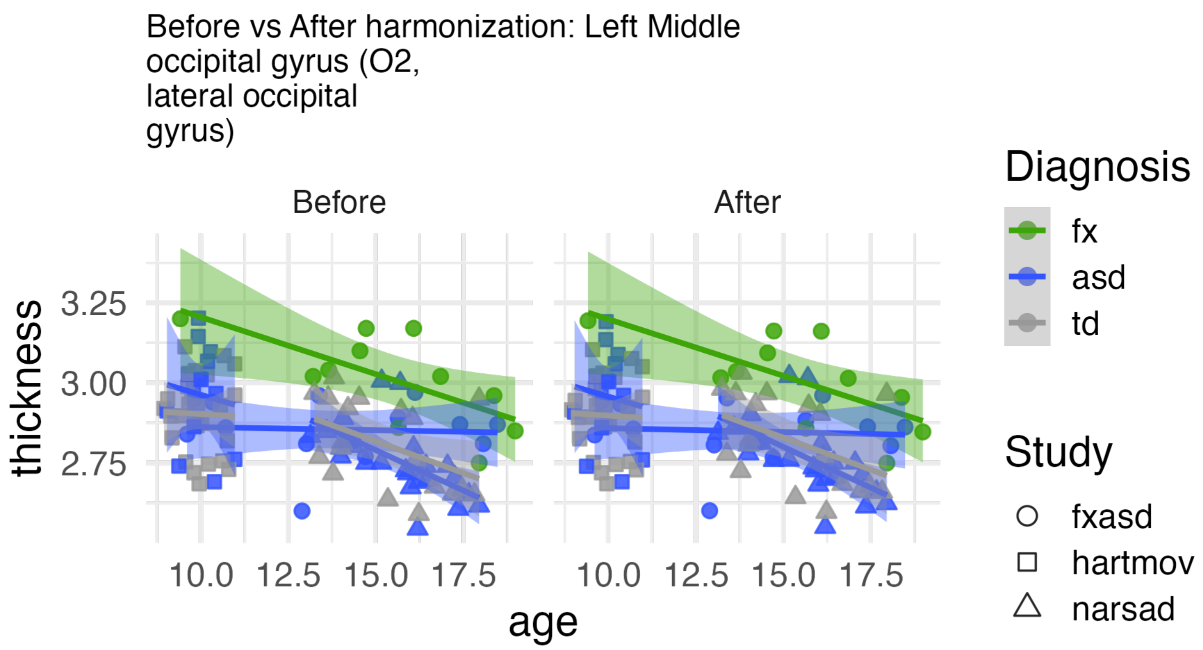
Before vs After harmonization: Left Middle
occipital gyrus (O2,
lateral occipital
gyrus)
Before
After
thickness
3.25
3.00
2.75
10.0
12.5
15.0
17.5
age
Diagnosis
fx
asd
td
Study
fxasd
hartmov
narsad

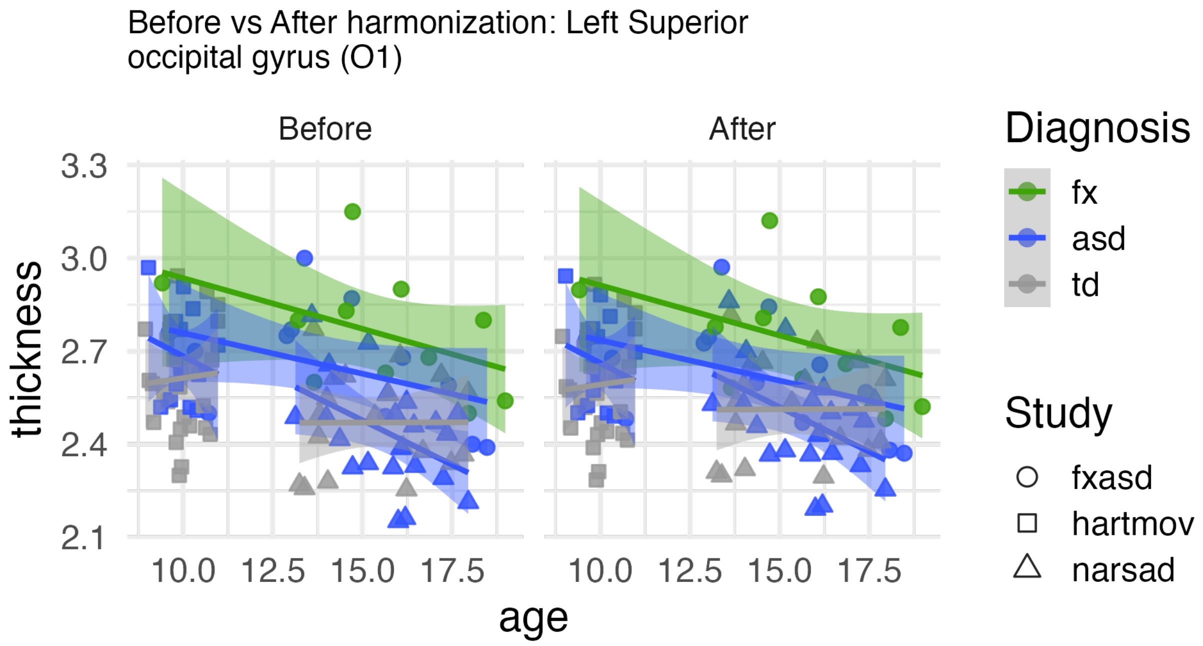
Before vs After harmonization: Left Superior
occipital gyrus (O1)
Before
After
thickness
3.3
3.0
2.7
2.4
2.1
10.0
12.5
15.0
17.5
age
Diagnosis
fx
asd
td
Study
fxasd
hartmov
narsad

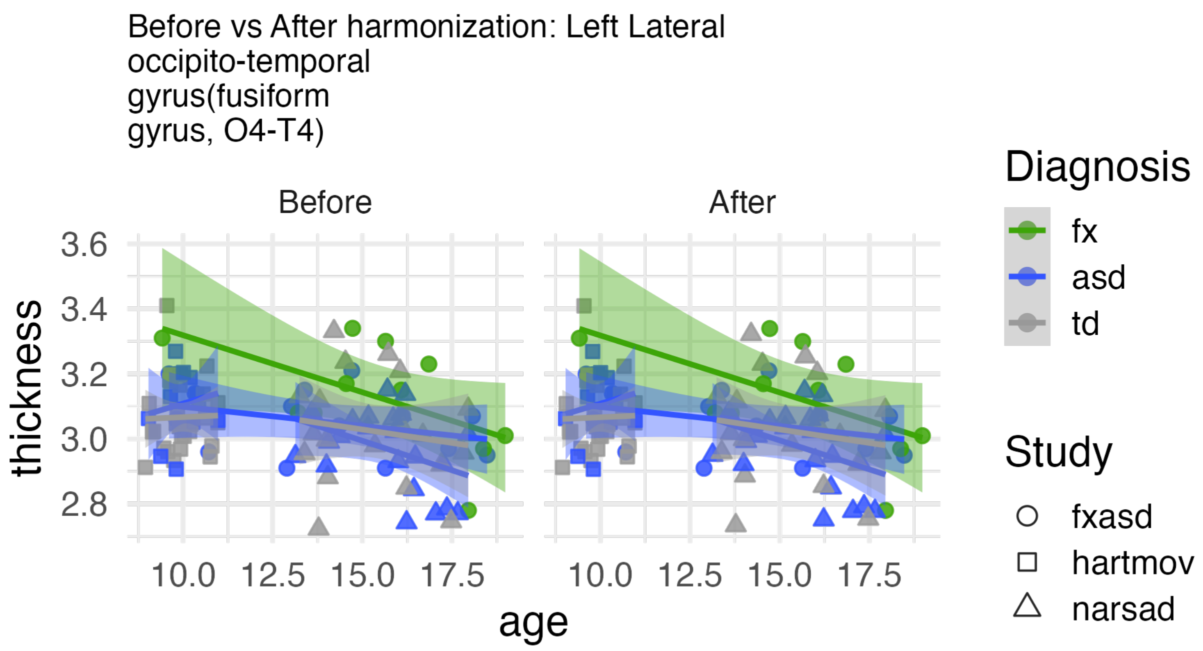
Before vs After harmonization: Left Lateral
occipito-temporal
gyrus(fusiform
gyrus, O4-T4)
Before
After
thickness
3.6
3.4
3.2
3.0
2.8
10.0
12.5
15.0
17.5
age
Diagnosis
fx
asd
td
Study
fxasd
hartmov
narsad

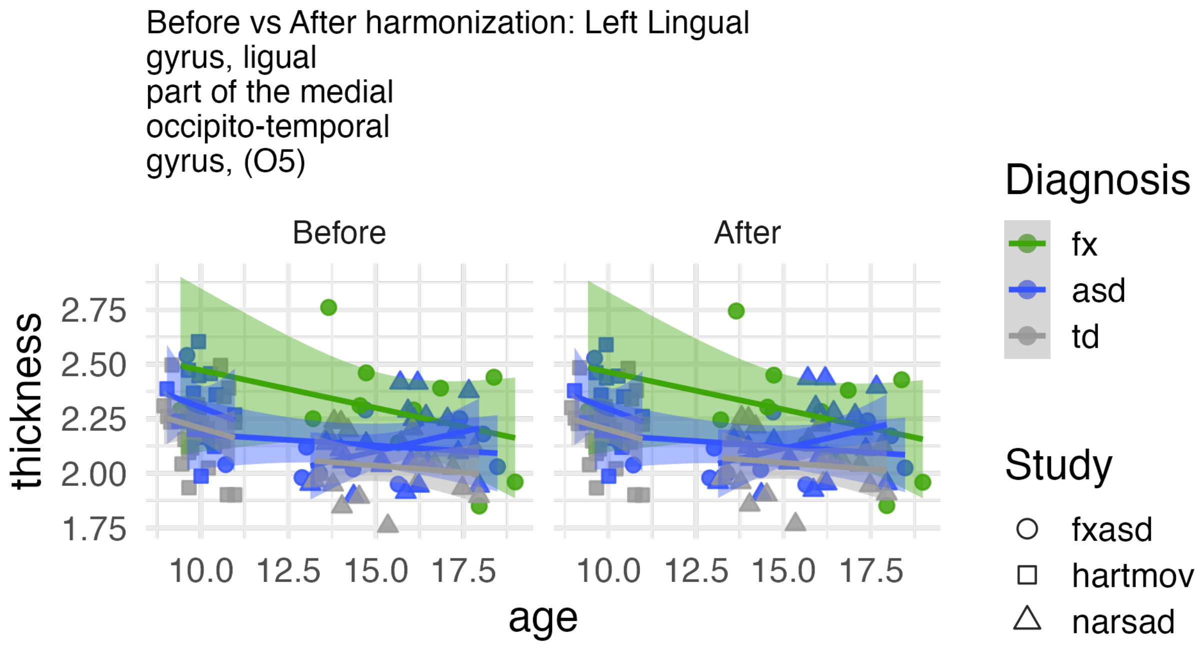
Before vs After harmonization: Left Lingual
gyrus, ligual
part of the medial
occipito-temporal
gyrus, (O5)
Before
After
thickness
2.75
2.50
2.25
2.00
1.75
10.0
12.5
15.0
17.5
age
Diagnosis
fx
asd
td
Study
fxasd
hartmov
narsad

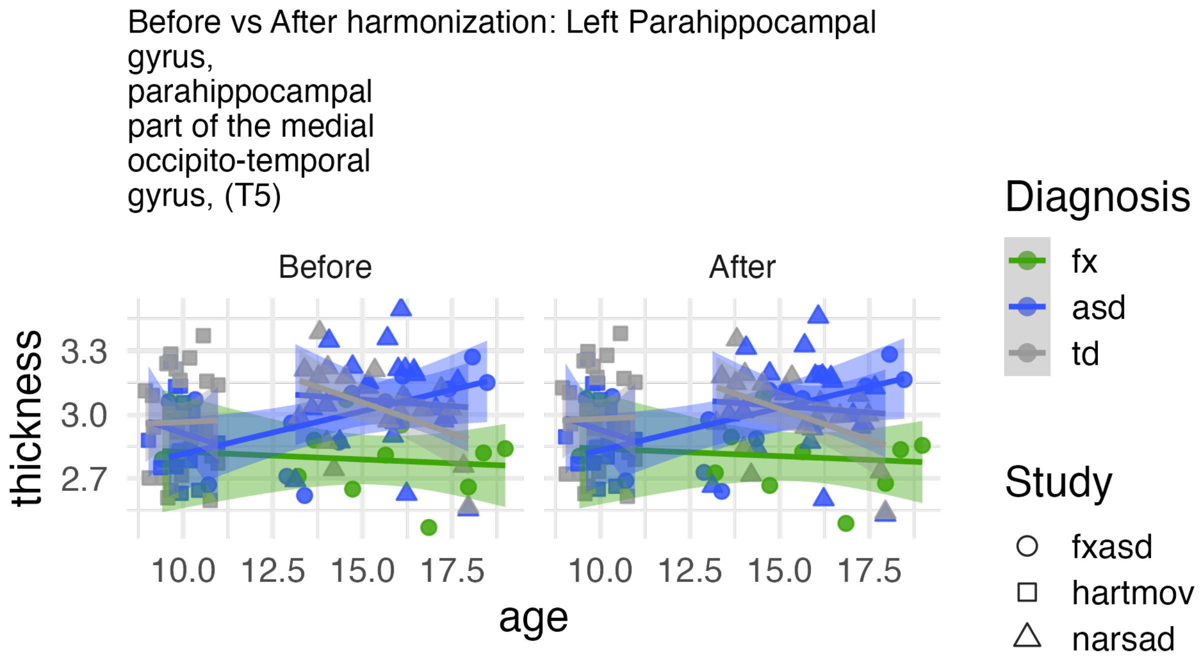

Before vs After harmonization: Left Parahippocampal gyrus,
parahippocampal part of the medial occipito-temporal gyrus, (T5)
Before
After
thickness
3.3
3.0
2.7
10.0 12.5 15.0 17.5
age
Diagnosis
fx
asd
td
Study
fxasd
hartmov
narsad

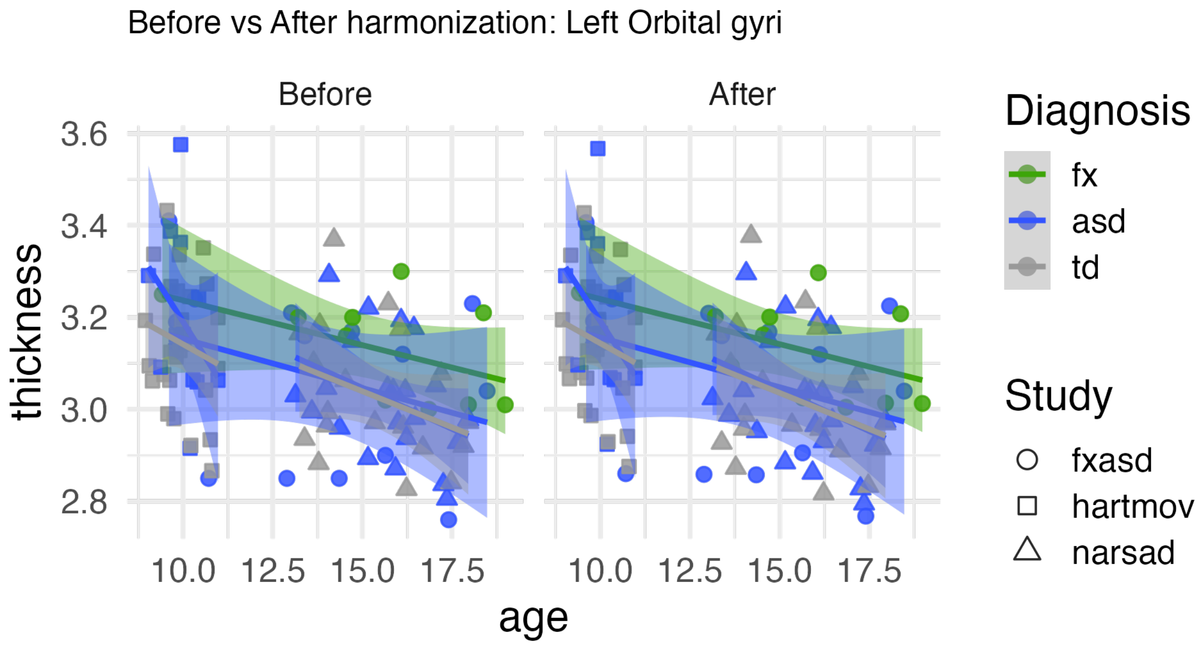

Before vs After harmonization: Left Orbital gyri
Before
After
thickness
3.6
3.4
3.2
3.0
2.8
10.0 12.5 15.0 17.5
age
Diagnosis
fx
asd
td
Study
fxasd
hartmov
narsad

Before vs After harmonization: Left Angular gyrus

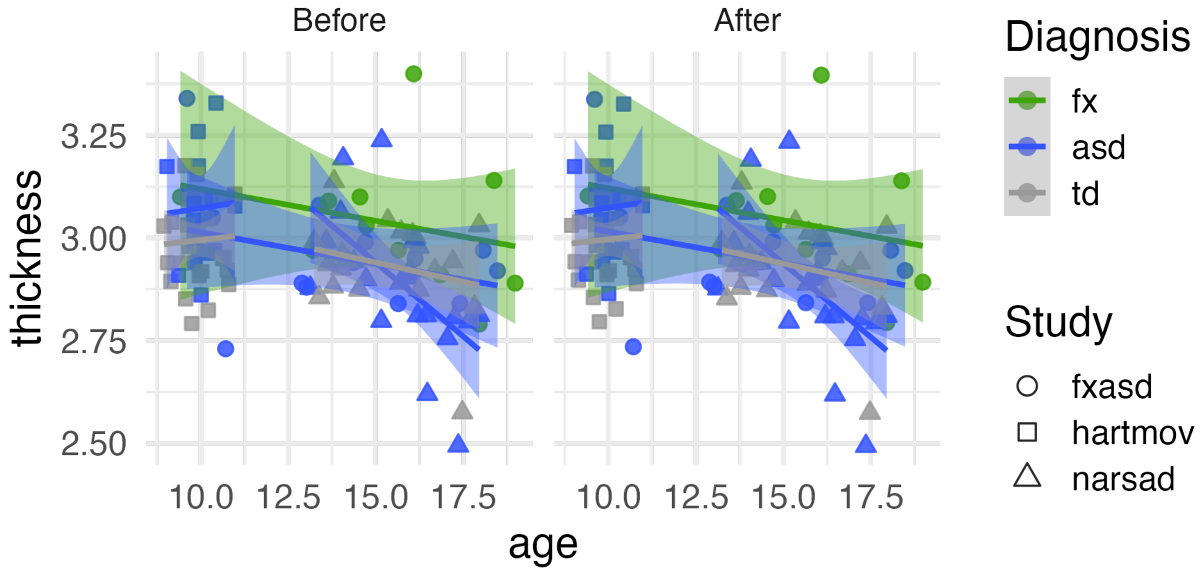

Before vs After harmonization: Left Supramarginal gyrus

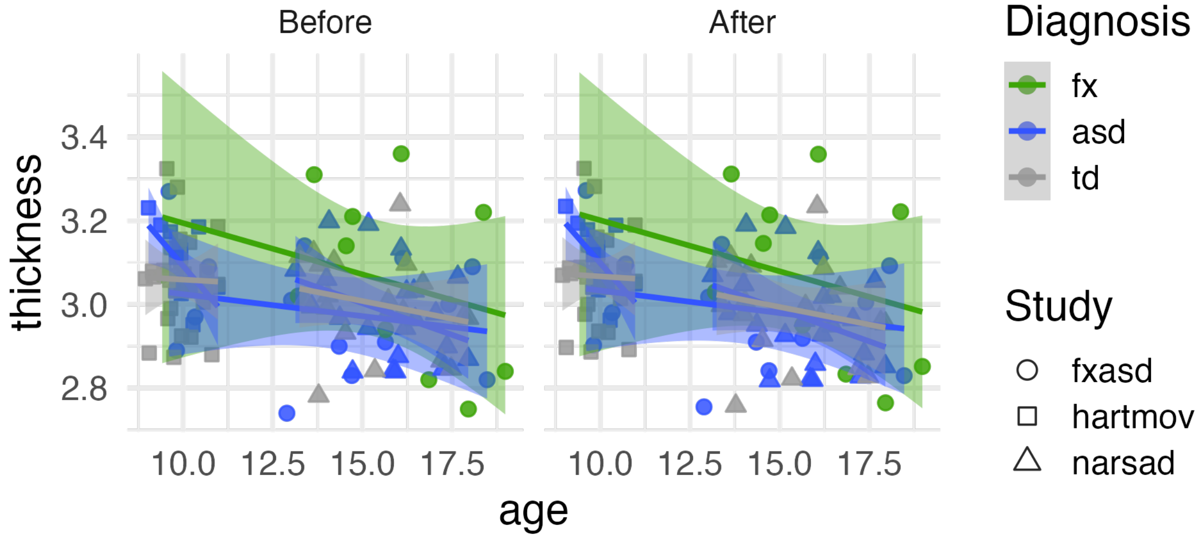

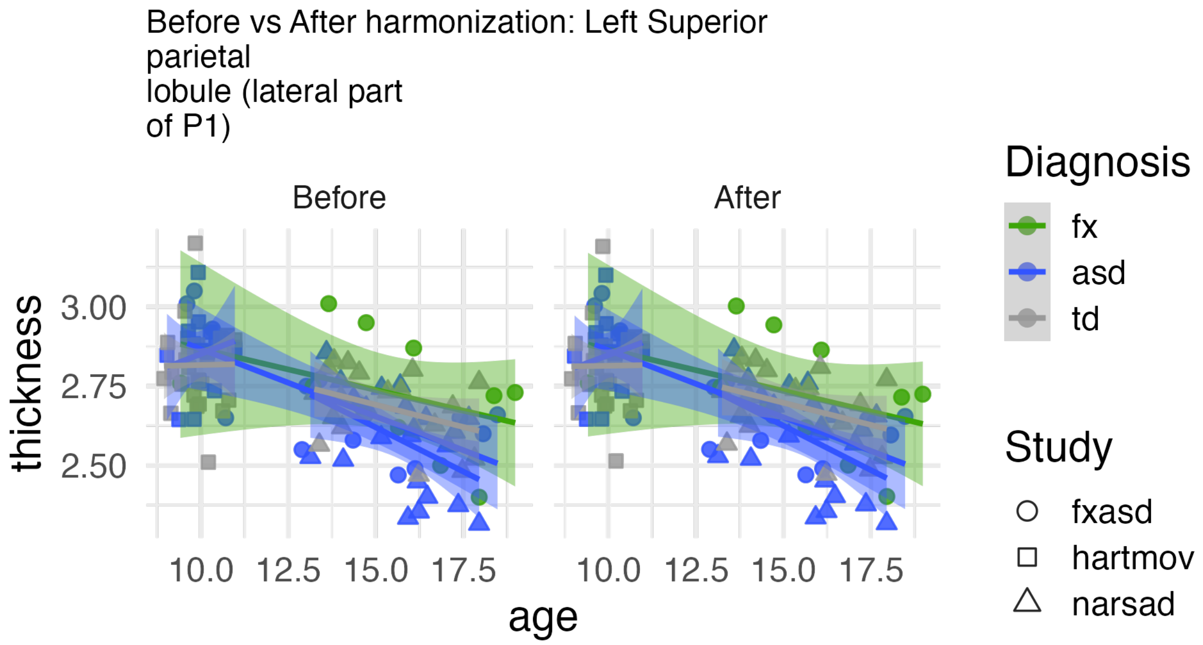

Before vs After harmonization: Left Superior parietal lobule (lateral part of P1)
Before
After
thickness
3.00
2.75
2.50
10.0
12.5
15.0
17.5
age
Diagnosis
fx
asd
td
Study
fxasd
hartmov
narsad

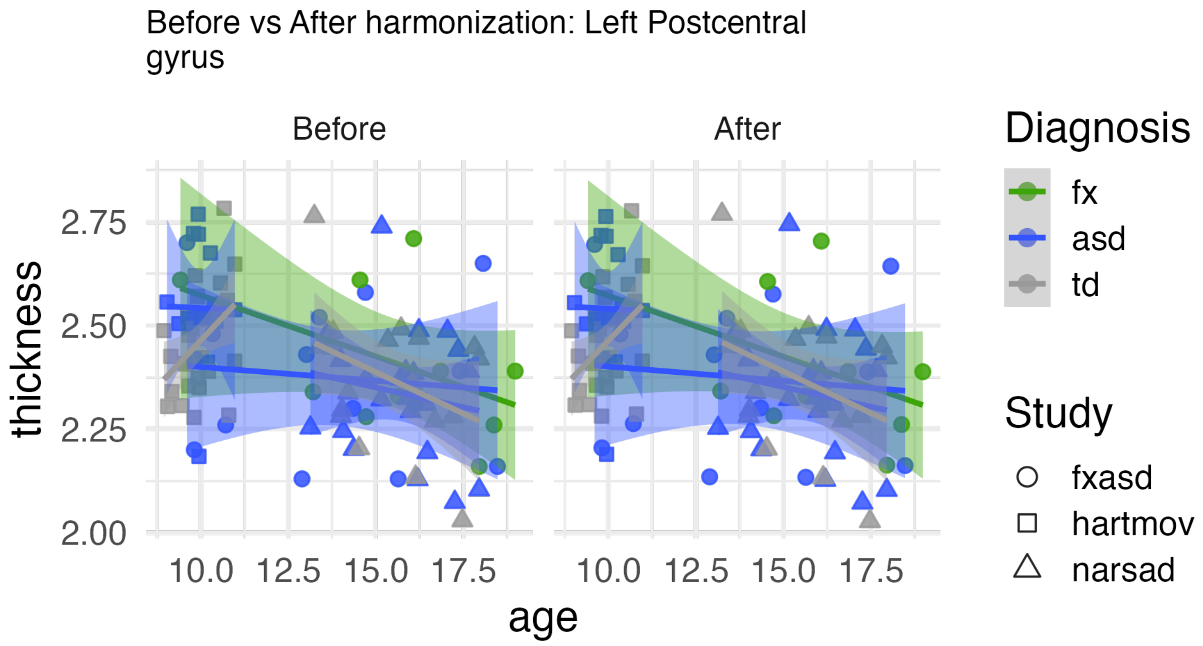

Before vs After harmonization: Left Postcentral gyrus
Before
After
thickness
2.75
2.50
2.25
2.00
10.0
12.5
15.0
17.5
age
Diagnosis
fx
asd
td
Study
fxasd
hartmov
narsad

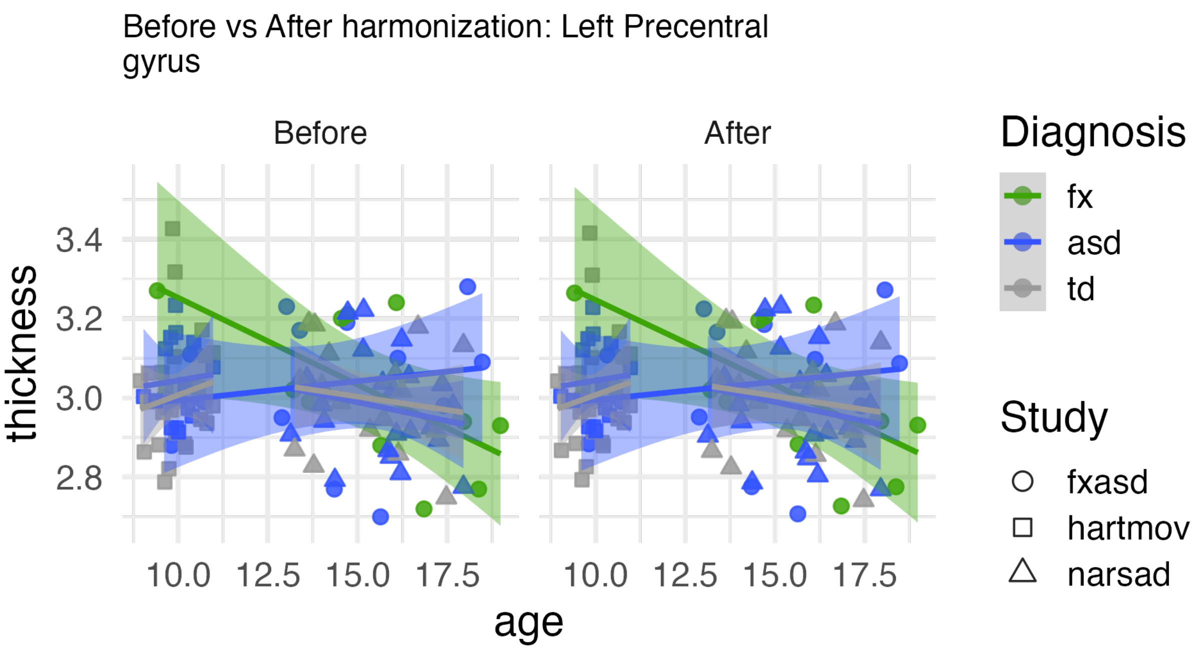

Before vs After harmonization: Left Precentral
gyrus
Before
After
thickness
3.4
3.2
3.0
2.8
10.0
12.5
15.0
17.5
age
Diagnosis
fx
asd
td
Study
fxasd
hartmov
narsad

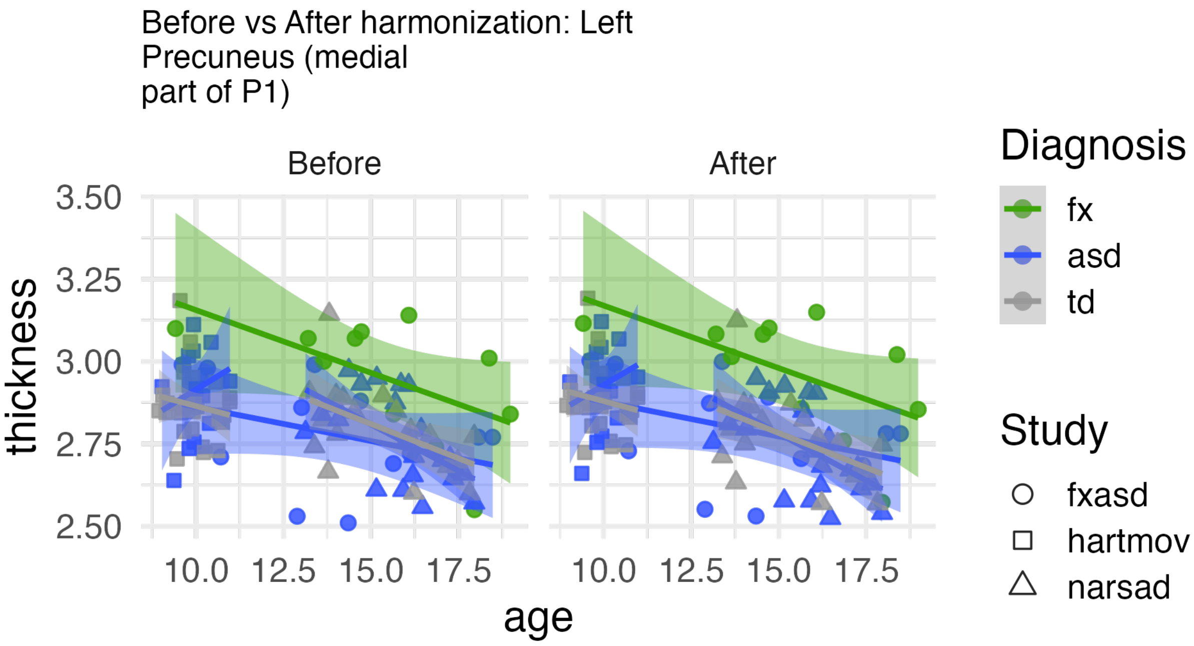

Before vs After harmonization: Left
Precuneus (medial
part of P1)
Before
After
thickness
3.50
3.25
3.00
2.75
2.50
10.0
12.5
15.0
17.5
age
Diagnosis
fx
asd
td
Study
fxasd
hartmov
narsad

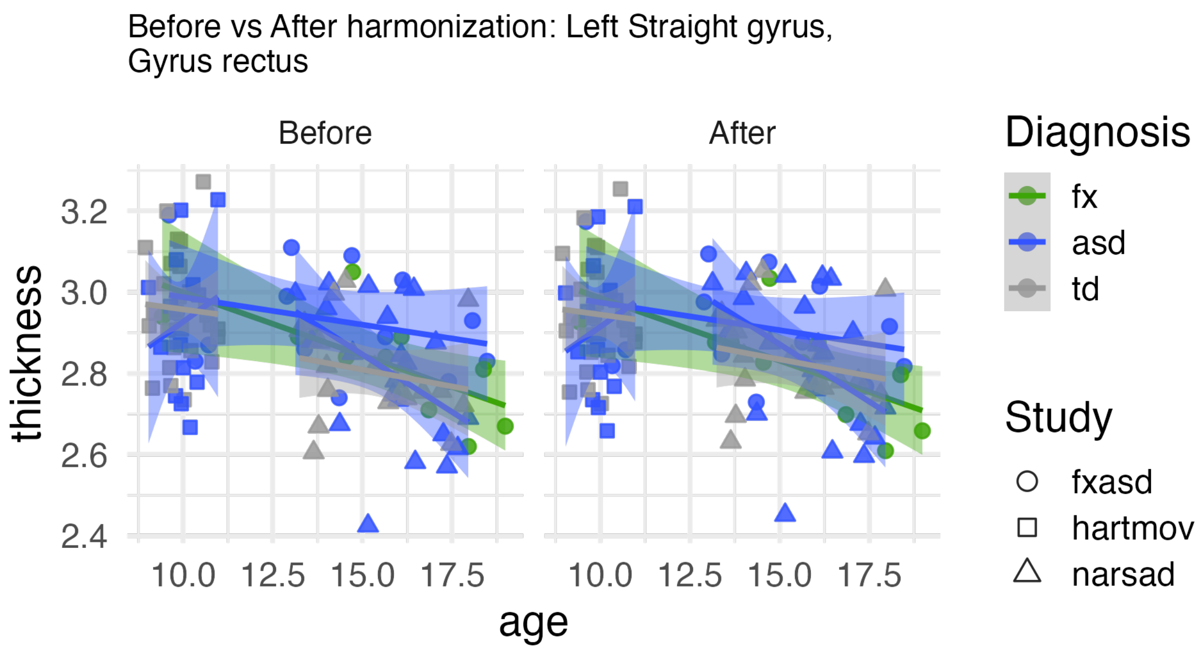

Before vs After harmonization: Left Straight gyrus,
Gyrus rectus
Before
After
thickness
3.2
3.0
2.8
2.6
2.4
10.0
12.5
15.0
17.5
age
Diagnosis
fx
asd
td
Study
fxasd
hartmov
narsad

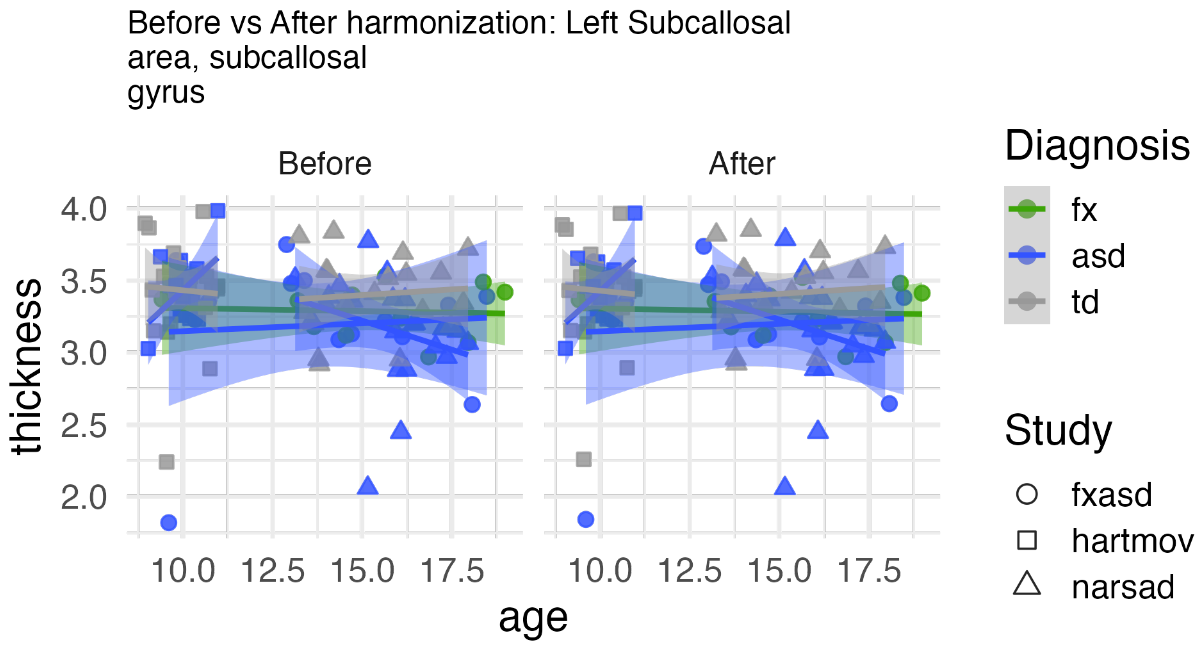

Before vs After harmonization: Left Subcallosal
area, subcallosal
gyrus
Before
After
thickness
4.0
3.5
3.0
2.5
2.0
10.0
12.5
15.0
17.5
age
Diagnosis
fx
asd
td
Study
fxasd
hartmov
narsad

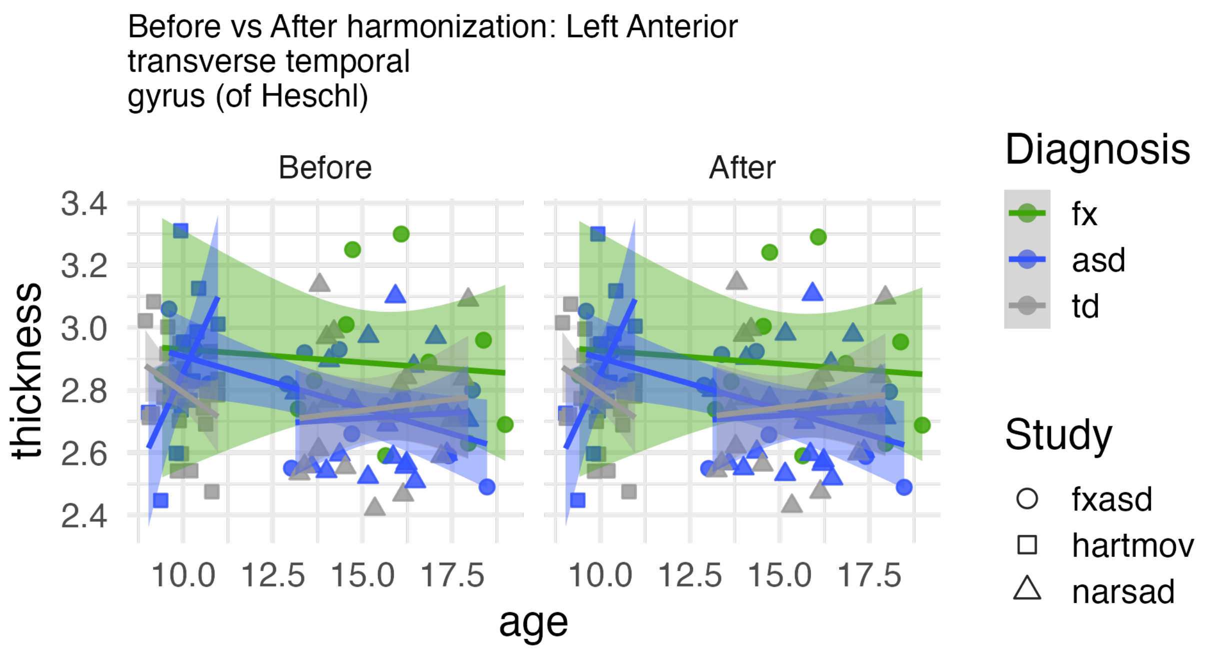
Before vs After harmonization: Left Anterior
transverse temporal
gyrus (of Heschl)
Before
After
thickness
age
Diagnosis
fx
asd
td
Study
fxasd
hartmov
narsad

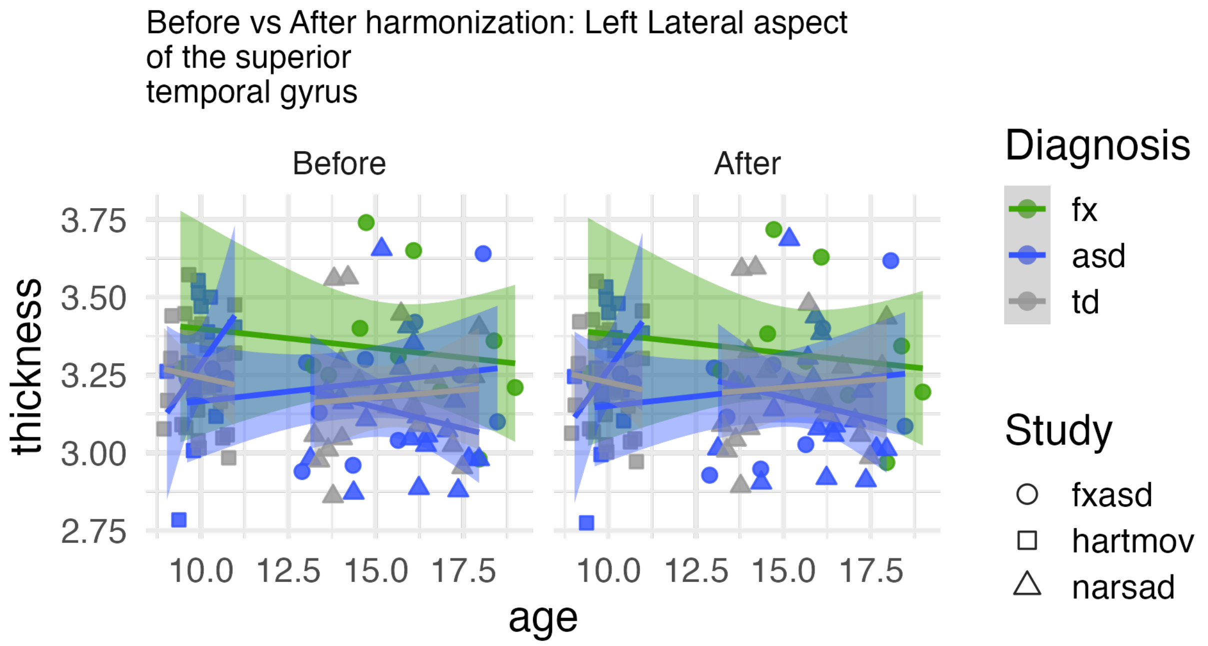
Before vs After harmonization: Left Lateral aspect
of the superior
temporal gyrus
Before
After
thickness
age
Diagnosis
fx
asd
td
Study
fxasd
hartmov
narsad

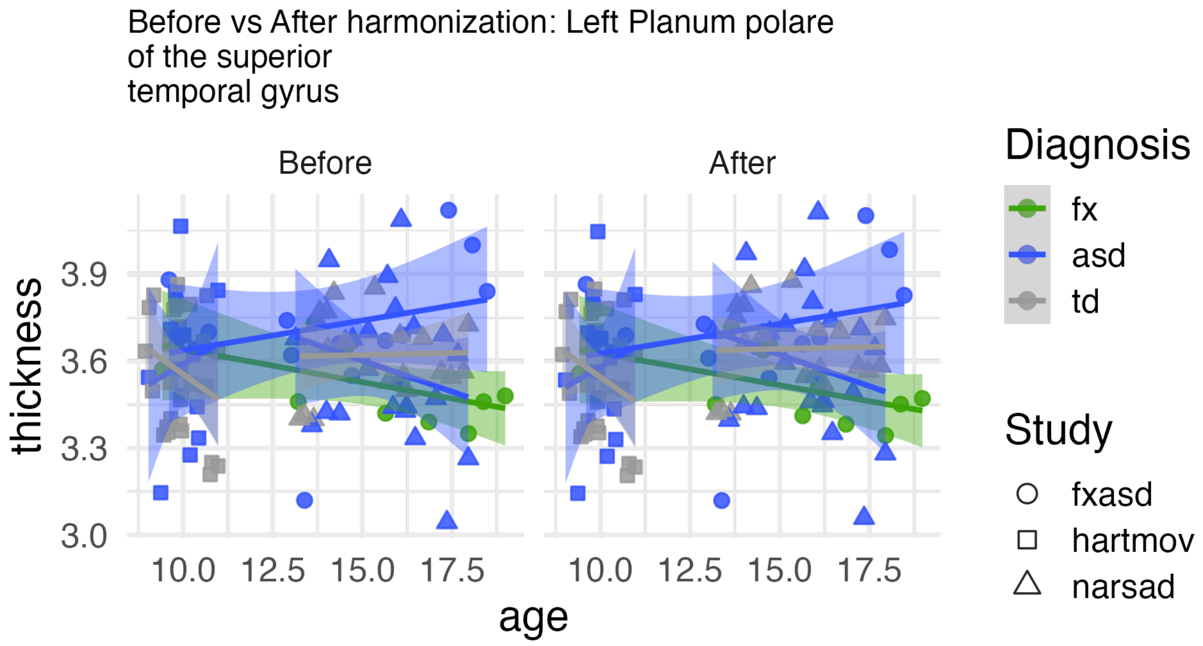

Before vs After harmonization: Left Planum polare
of the superior
temporal gyrus
Before
After
thickness
3.9
3.6
3.3
3.0
10.0
12.5
15.0
17.5
age
Diagnosis
fx
asd
td
Study
fxasd
hartmov
narsad

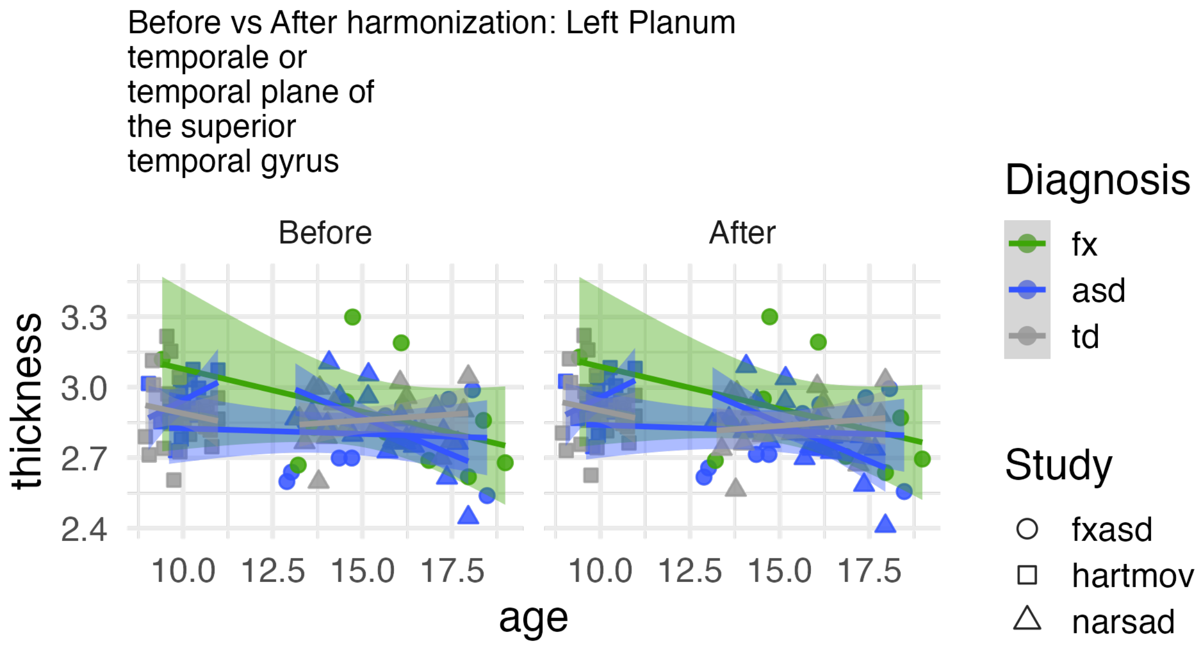

Before vs After harmonization: Left Planum
temporale or
temporal plane of
the superior
temporal gyrus
Before
After
thickness
3.3
3.0
2.7
2.4
10.0
12.5
15.0
17.5
age
Diagnosis
fx
asd
td
Study
fxasd
hartmov
narsad

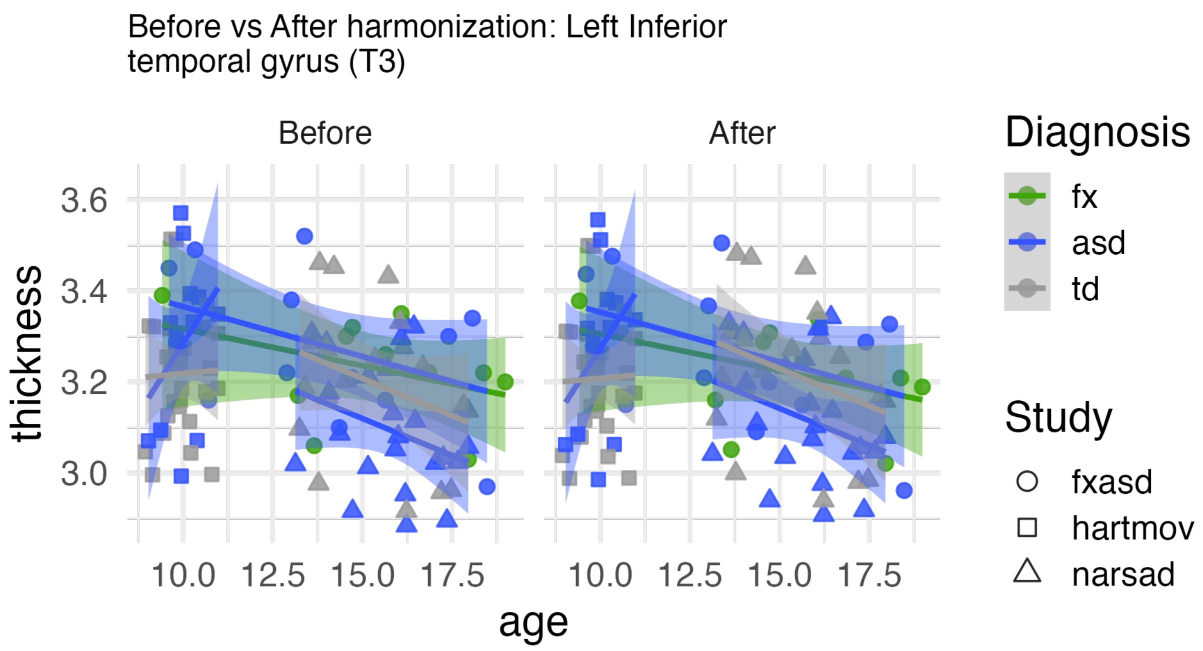
Before vs After harmonization: Left Inferior temporal gyrus (T3)
Before
After
thickness
3.6
3.4
3.2
3.0
10.0
12.5
15.0
17.5
age
Diagnosis
fx
asd
td
Study
fxasd
hartmov
narsad

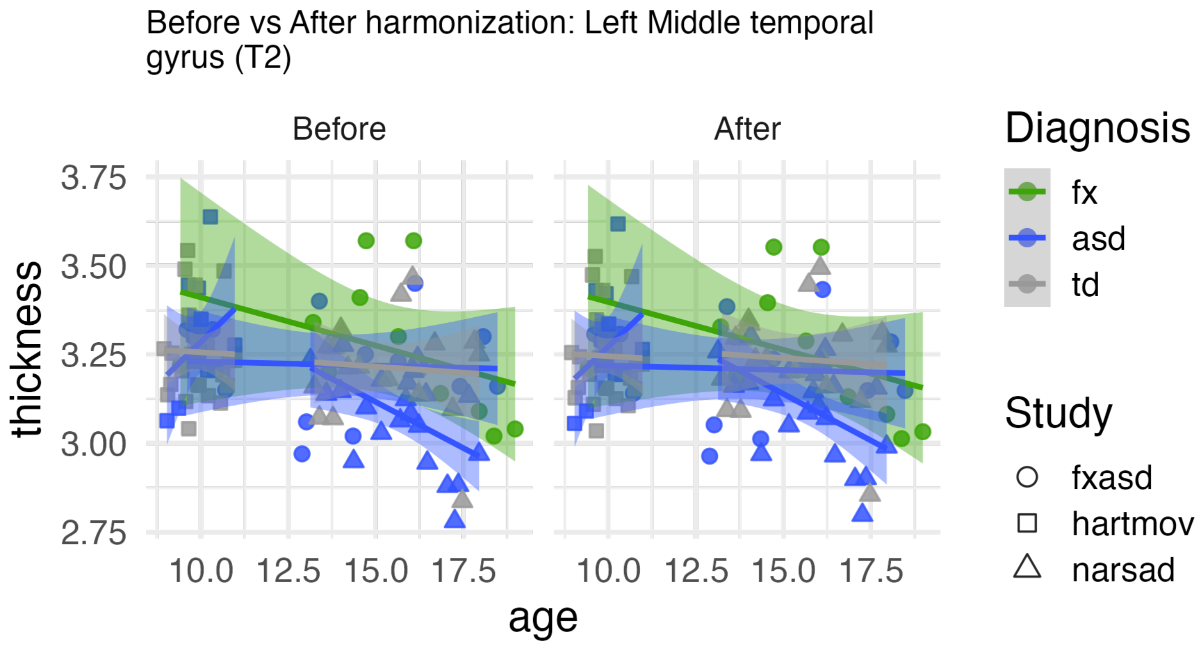
Before vs After harmonization: Left Middle temporal gyrus (T2)
Before
After
thickness
3.75
3.50
3.25
3.00
2.75
10.0
12.5
15.0
17.5
age
Diagnosis
fx
asd
td
Study
fxasd
hartmov
narsad

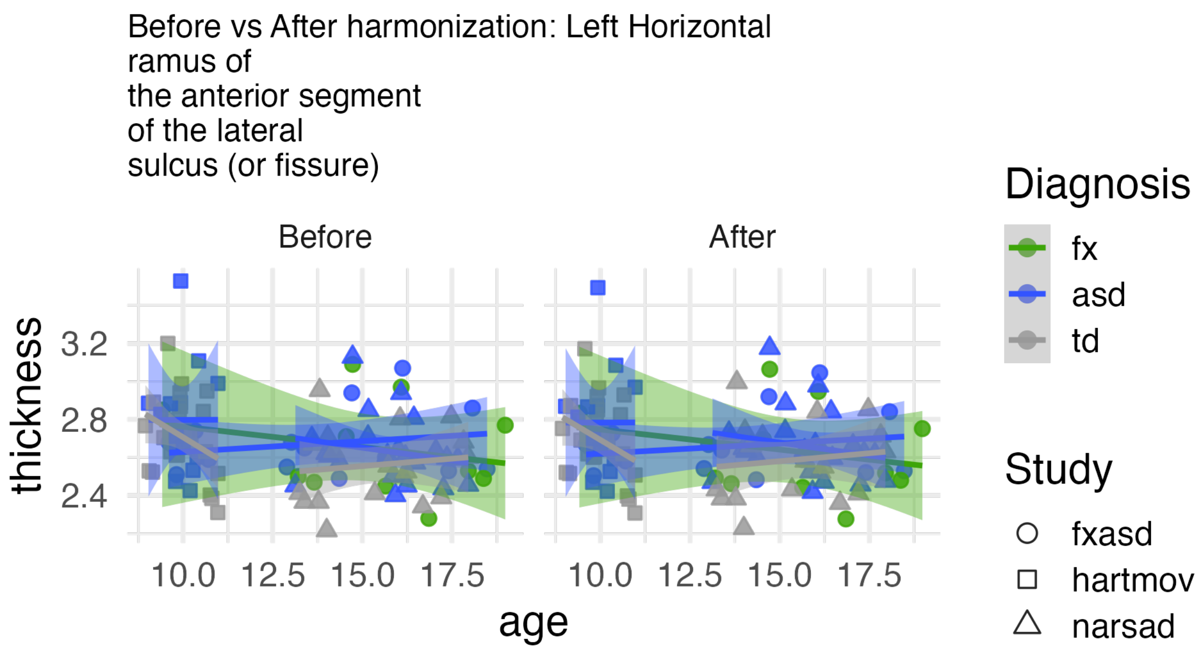

Before vs After harmonization: Left Horizontal ramus of the anterior segment of the lateral sulcus (or fissure)
Before
After
thickness
3.2
2.8
2.4
10.0
12.5
15.0
17.5
age
Diagnosis
fx
asd
td
Study
fxasd
hartmov
narsad

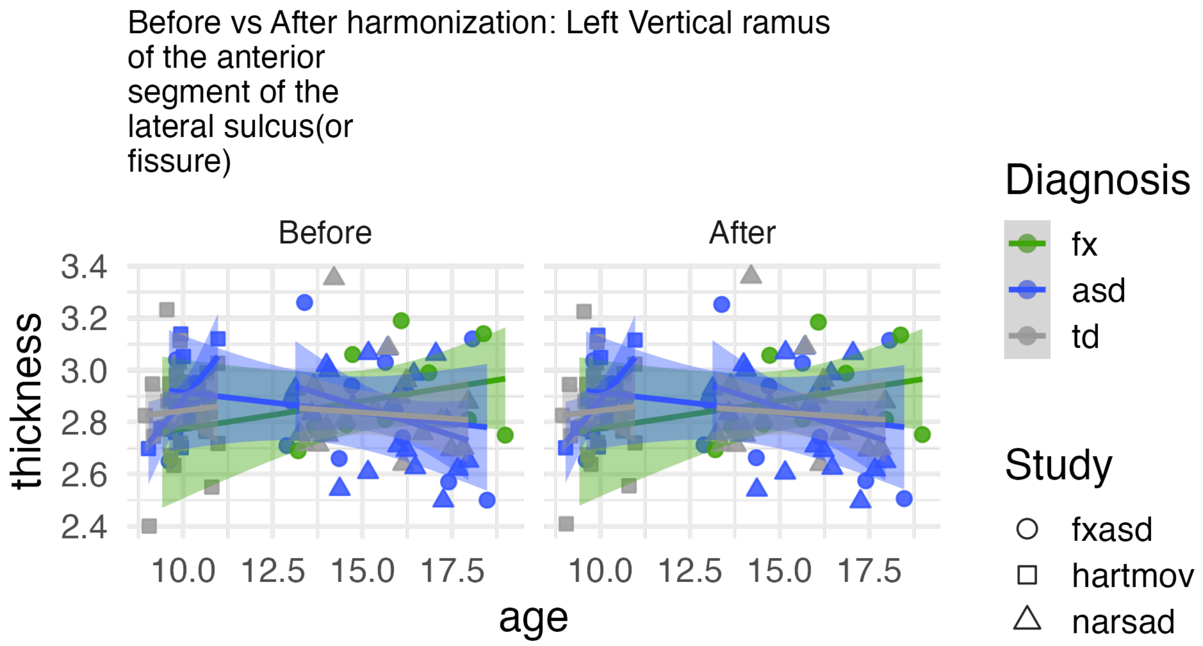

Before vs After harmonization: Left Vertical ramus of the anterior segment of the lateral sulcus(or fissure)
Before
After
thickness
3.4
3.2
3.0
2.8
2.6
2.4
10.0
12.5
15.0
17.5
age
Diagnosis
fx
asd
td
Study
fxasd
hartmov
narsad

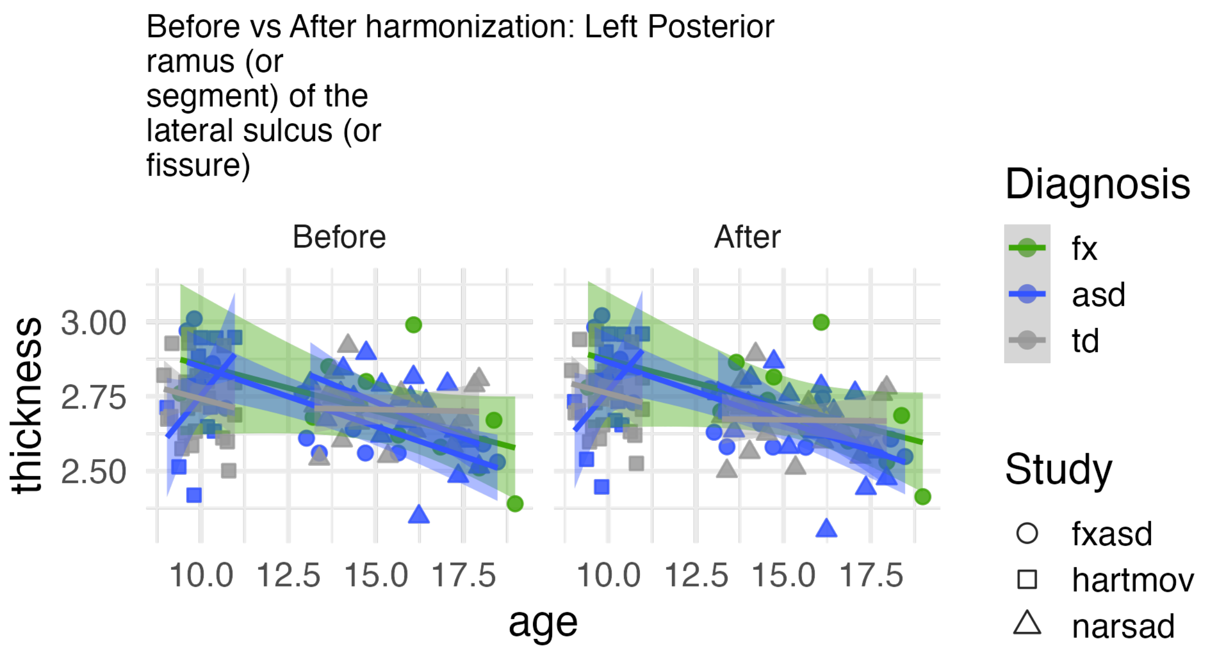
Before vs After harmonization: Left Posterior ramus (or segment) of the lateral sulcus (or fissure)
Before
After
thickness
3.00
2.75
2.50
10.0
12.5
15.0
17.5
age
Diagnosis
fx
asd
td
Study
fxasd
hartmov
narsad

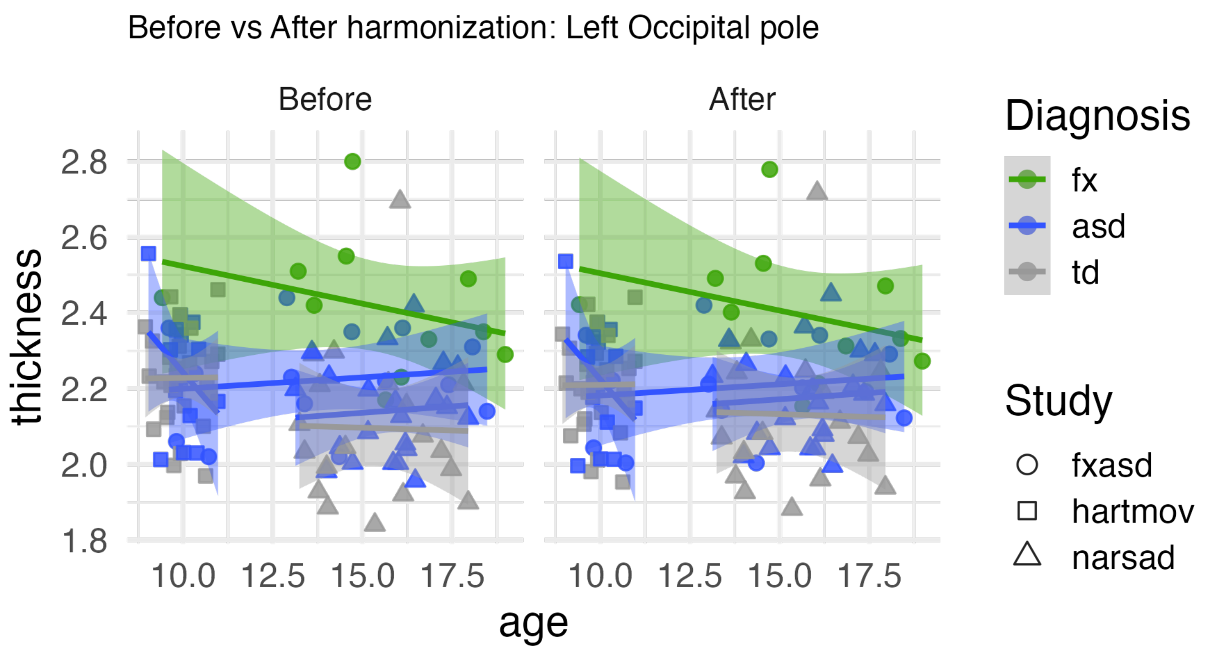
Before vs After harmonization: Left Occipital pole
Before
After
thickness
2.8
2.6
2.4
2.2
2.0
1.8
10.0
12.5
15.0
17.5
age
Diagnosis
fx
asd
td
Study
fxasd
hartmov
narsad

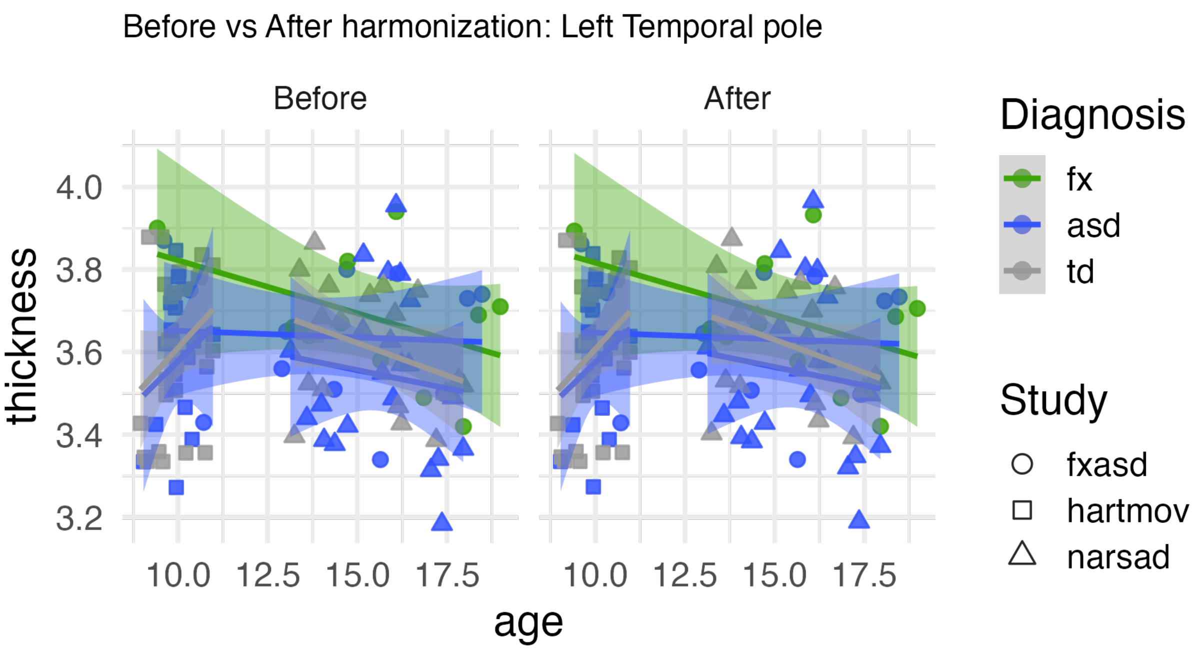
Before vs After harmonization: Left Temporal pole
Before
After
Diagnosis
fx
asd
td
Study
fxasd
hartmov
narsad
thickness
4.0
3.8
3.6
3.4
3.2
10.0
12.5
15.0
17.5
age

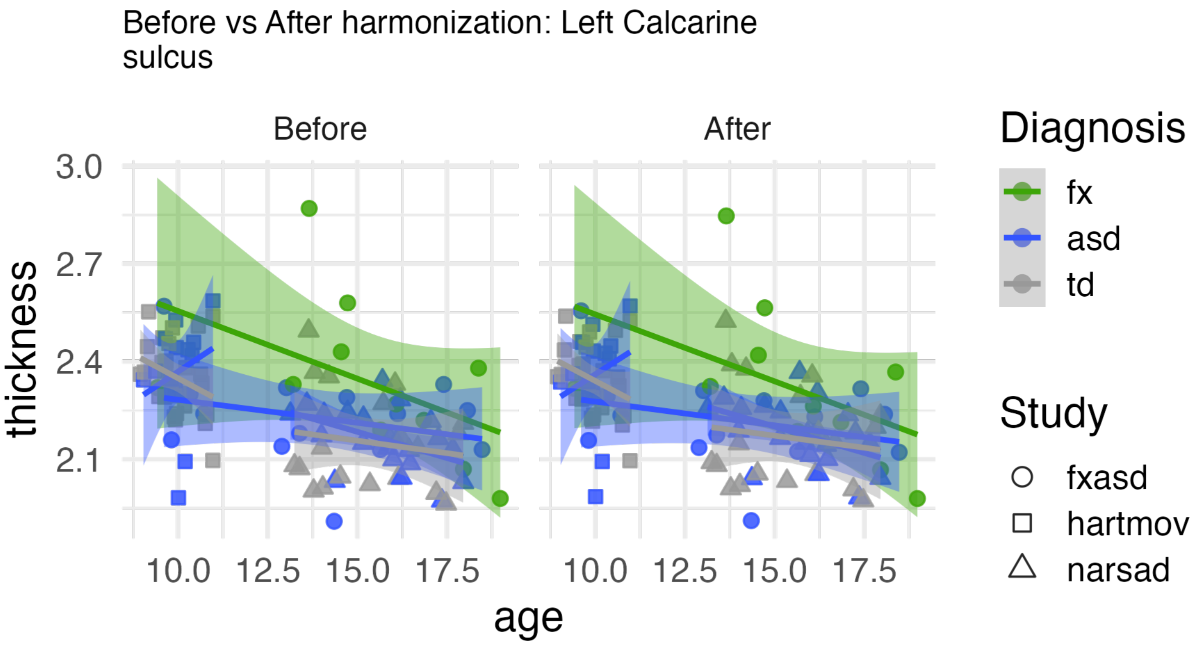
Before vs After harmonization: Left Calcarine sulcus
Before
After
Diagnosis
fx
asd
td
Study
fxasd
hartmov
narsad
thickness
3.0
2.7
2.4
2.1
10.0
12.5
15.0
17.5
age

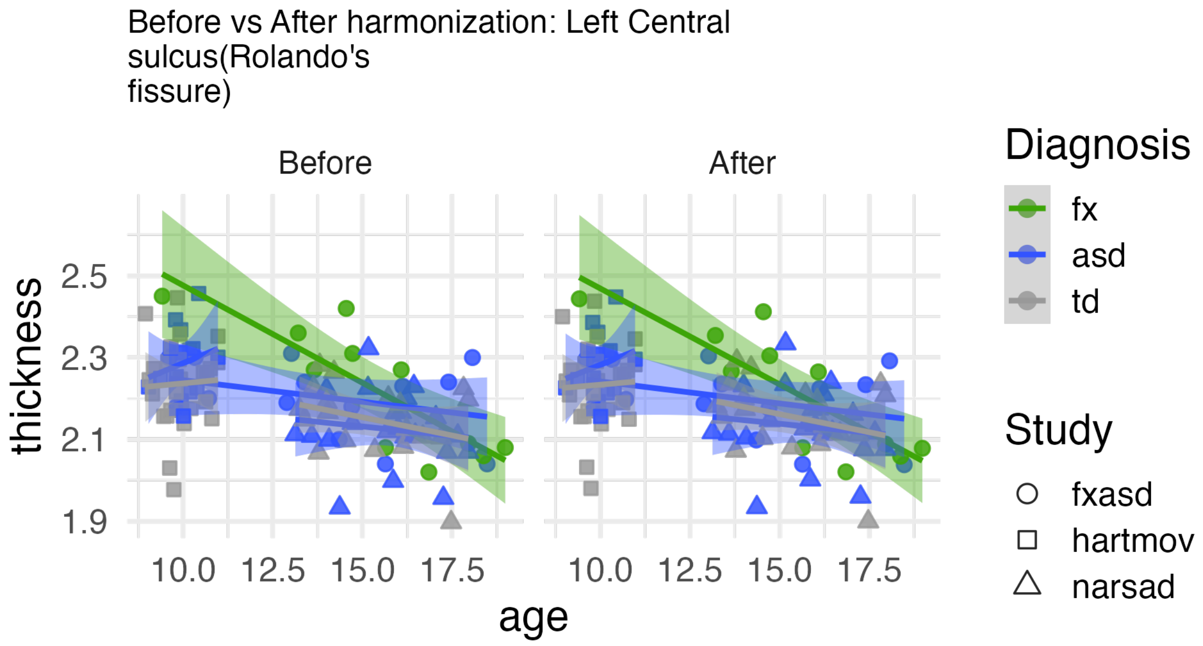
Before vs After harmonization: Left Central sulcus(Rolando's fissure)
Before
After
thickness
2.5
2.3
2.1
1.9
10.0
12.5
15.0
17.5
age
Diagnosis
fx
asd
td
Study
fxasd
hartmov
narsad

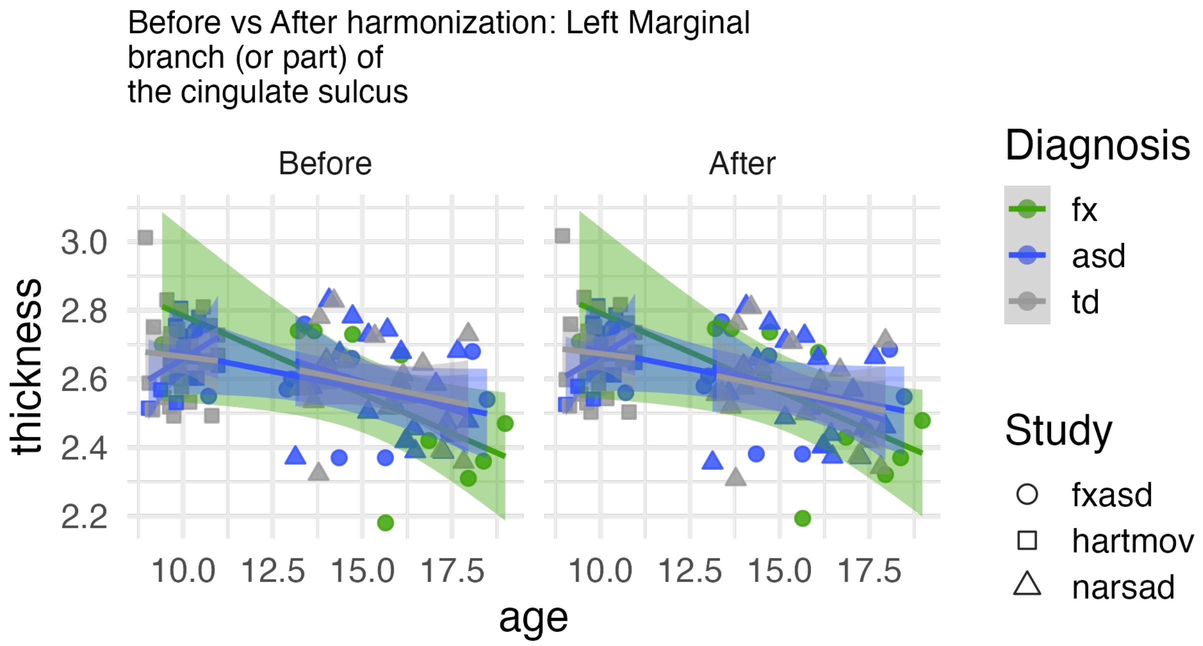
Before vs After harmonization: Left Marginal branch (or part) of the cingulate sulcus
Before
After
thickness
3.0
2.8
2.6
2.4
2.2
10.0
12.5
15.0
17.5
age
Diagnosis
fx
asd
td
Study
fxasd
hartmov
narsad

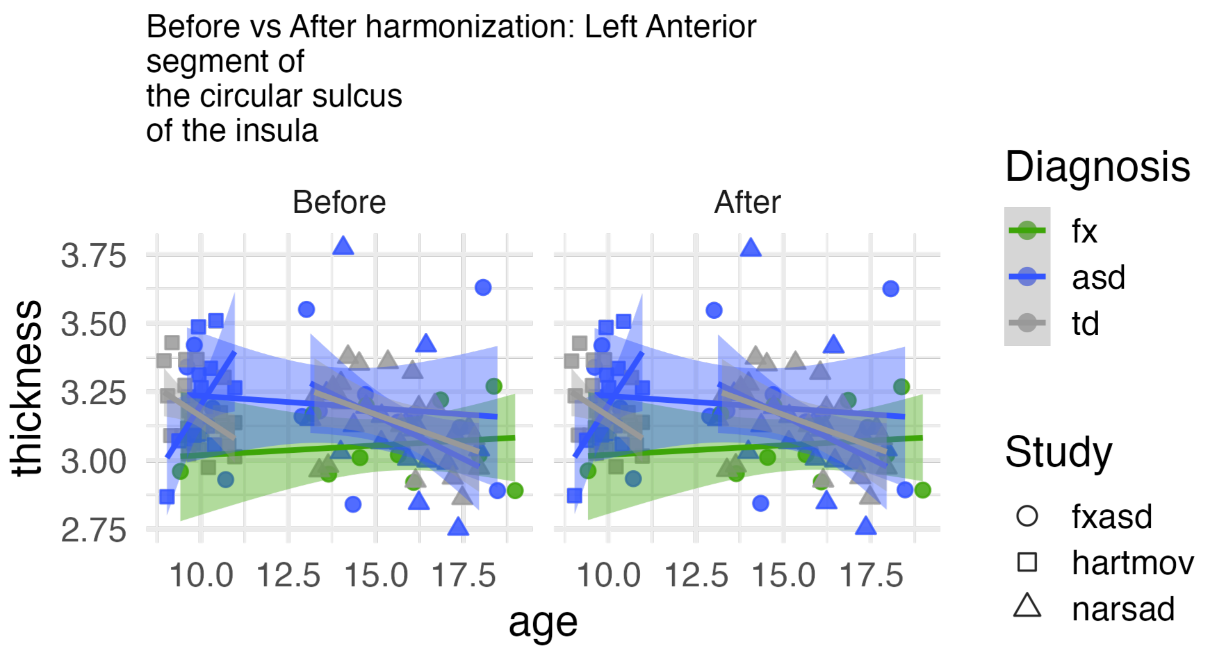
Before vs After harmonization: Left Anterior
segment of
the circular sulcus
of the insula
Before
After
thickness
3.75
3.50
3.25
3.00
2.75
10.0
12.5
15.0
17.5
age
Diagnosis
fx
asd
td
Study
fxasd
hartmov
narsad

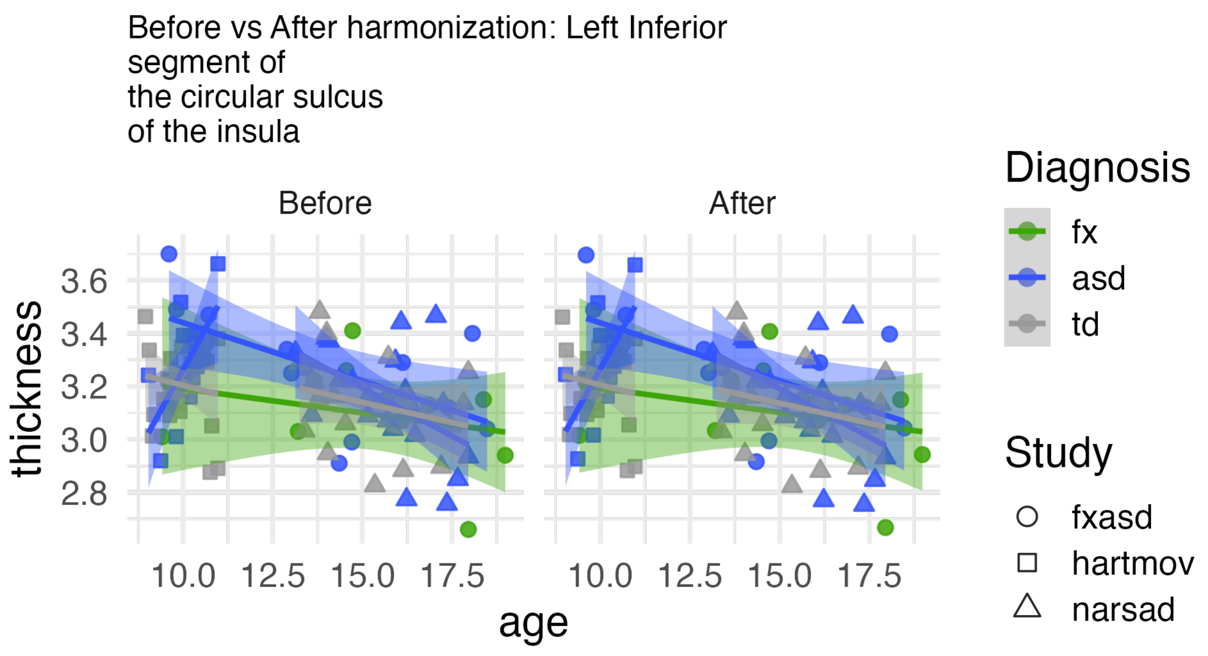
Before vs After harmonization: Left Inferior
segment of
the circular sulcus
of the insula
Before
After
thickness
3.6
3.4
3.2
3.0
2.8
10.0
12.5
15.0
17.5
age
Diagnosis
fx
asd
td
Study
fxasd
hartmov
narsad

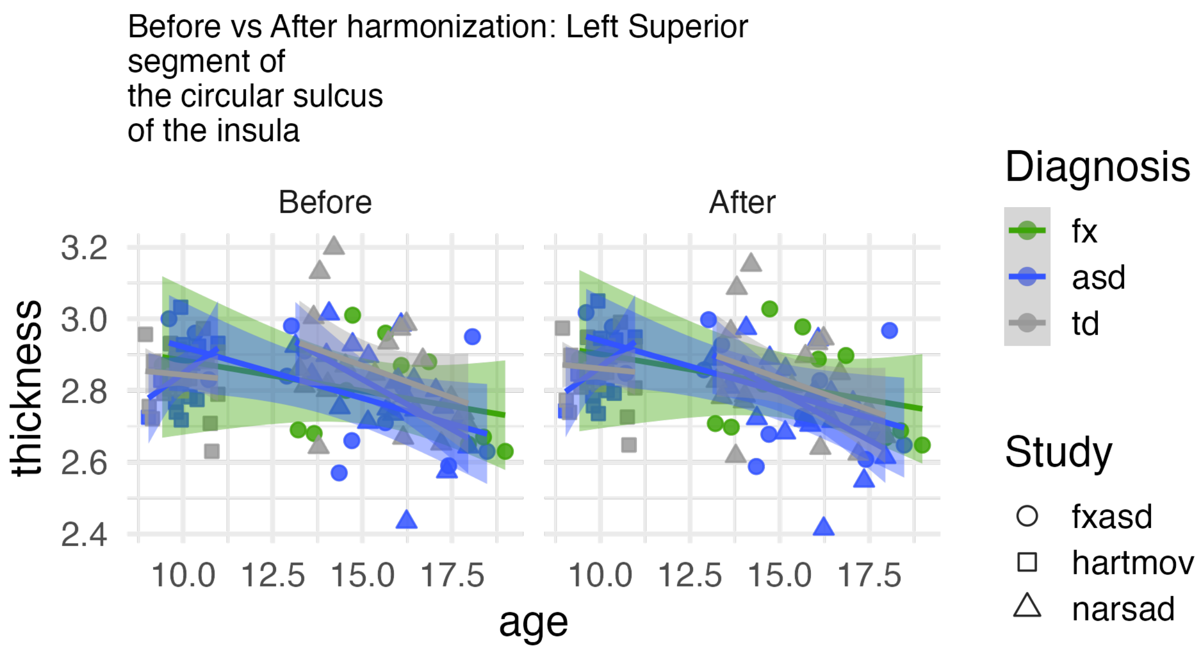
Before vs After harmonization: Left Superior
segment of
the circular sulcus
of the insula
Before
After
thickness
3.2
3.0
2.8
2.6
2.4
10.0
12.5
15.0
17.5
age
Diagnosis
fx
asd
td
Study
fxasd
hartmov
narsad

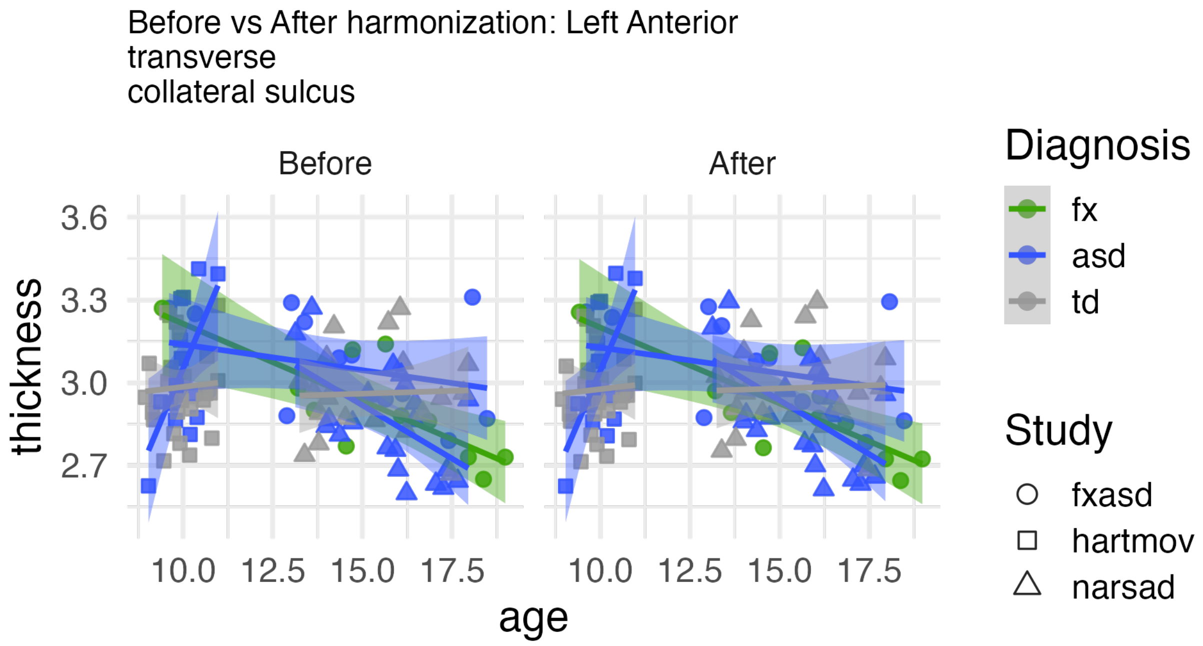
Before vs After harmonization: Left Anterior
transverse
collateral sulcus
Before
After
thickness
3.6
3.3
3.0
2.7
10.0
12.5
15.0
17.5
age
Diagnosis
fx
asd
td
Study
fxasd
hartmov
narsad

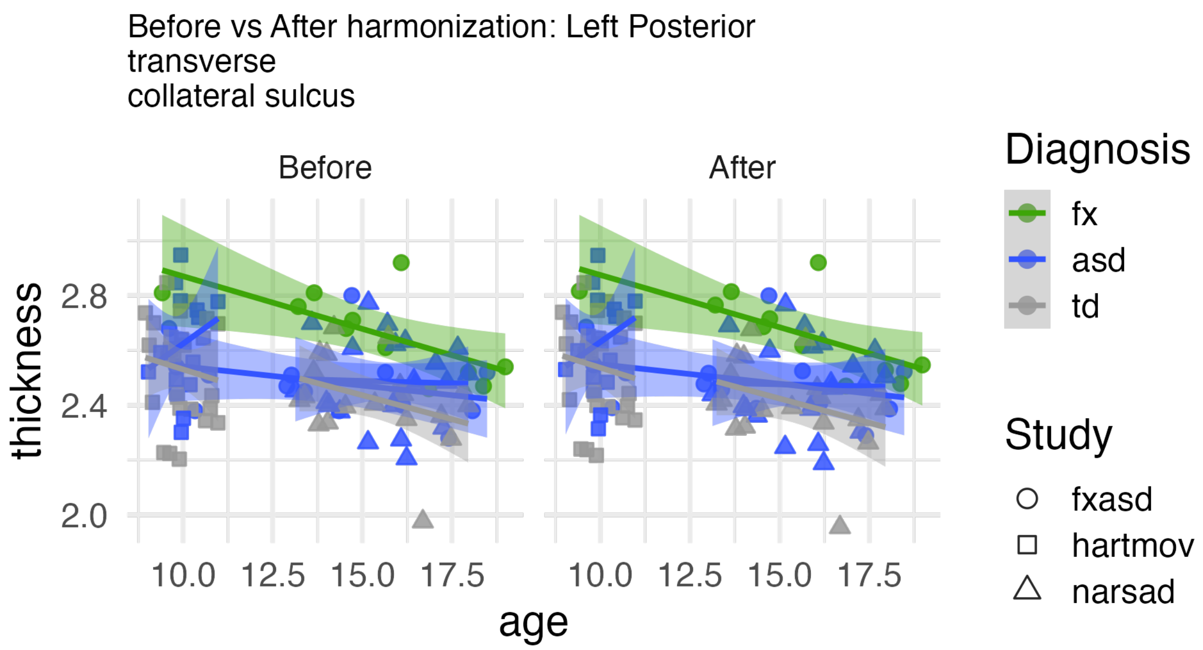
Before vs After harmonization: Left Posterior
transverse
collateral sulcus
Before
After
thickness
2.8
2.4
2.0
10.0 12.5 15.0 17.5
age
Diagnosis
fx
asd
td
Study
fxasd
hartmov
narsad

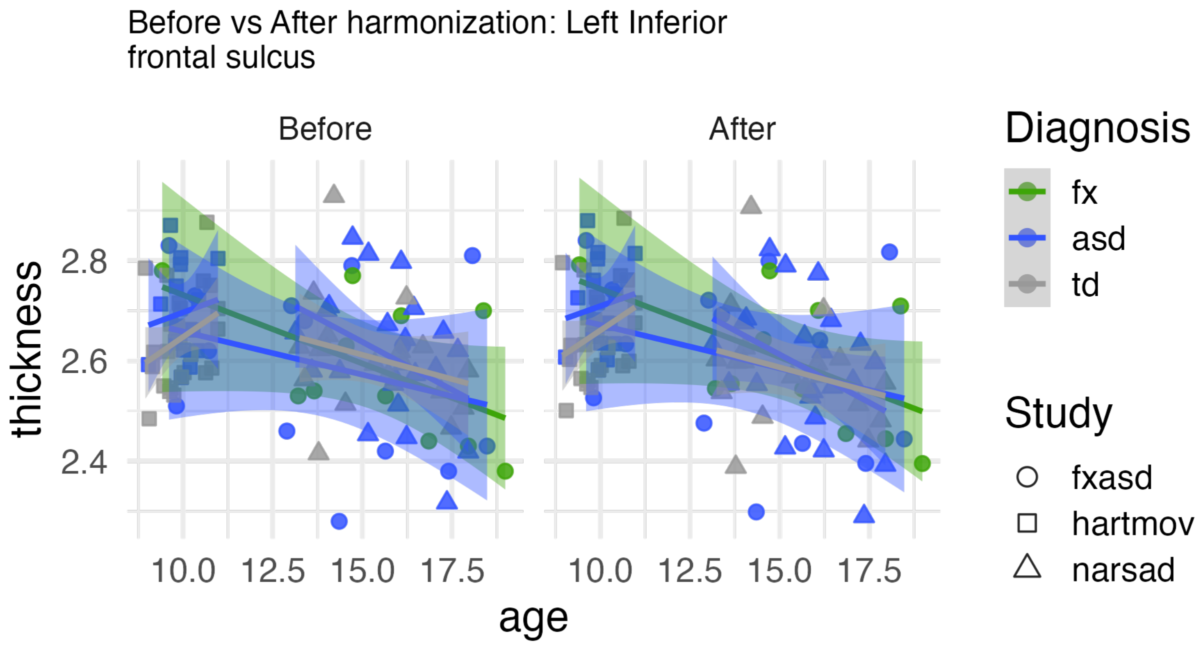
Before vs After harmonization: Left Inferior
frontal sulcus
Before
After
thickness
2.8
2.6
2.4
10.0 12.5 15.0 17.5
age
Diagnosis
fx
asd
td
Study
fxasd
hartmov
narsad

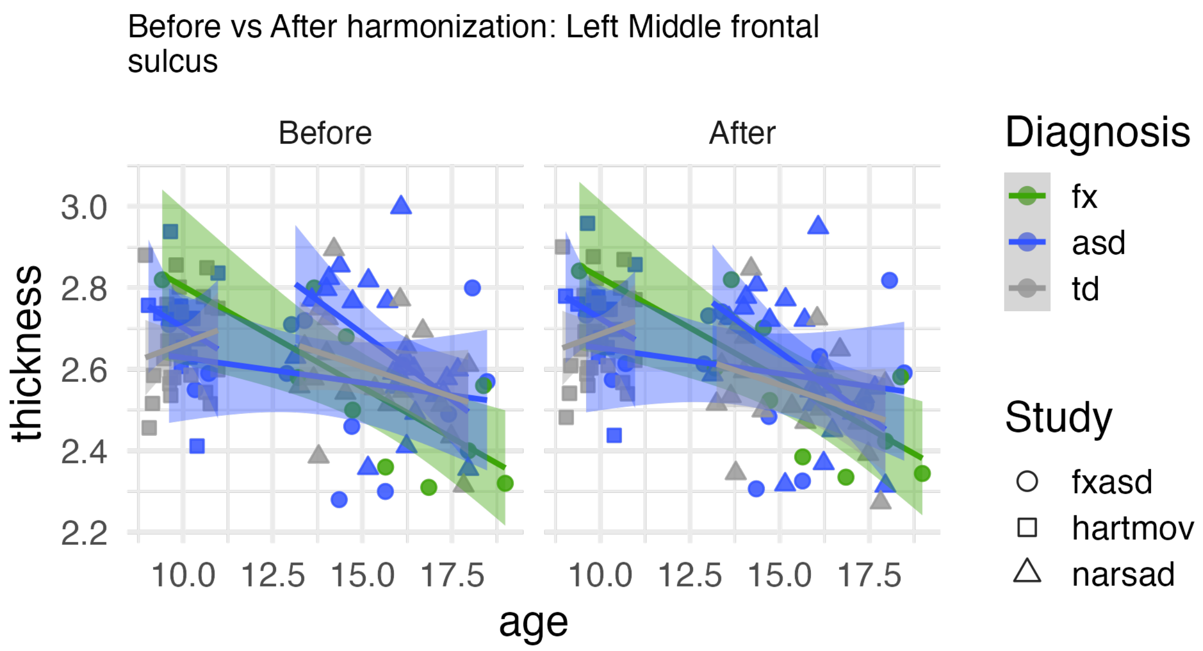
Before vs After harmonization: Left Middle frontal sulcus
Before
After
thickness
3.0
2.8
2.6
2.4
2.2
10.0
12.5
15.0
17.5
age
Diagnosis
fx
asd
td
Study
fxasd
hartmov
narsad

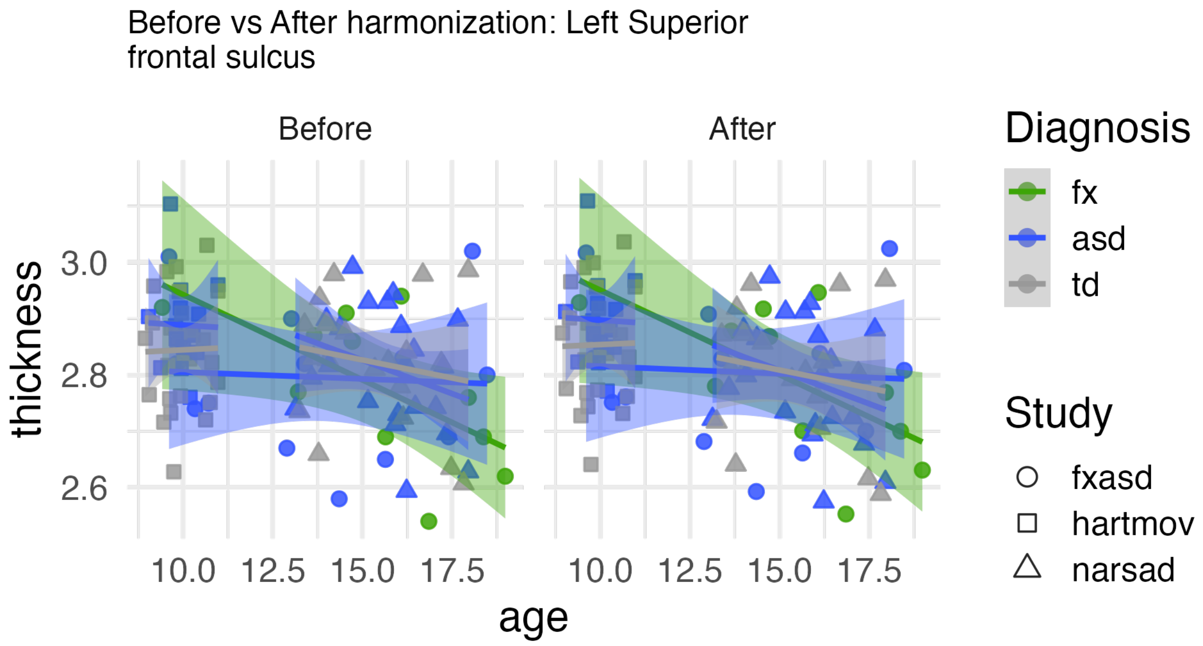
Before vs After harmonization: Left Superior frontal sulcus
Before
After
thickness
3.0
2.8
2.6
10.0
12.5
15.0
17.5
age
Diagnosis
fx
asd
td
Study
fxasd
hartmov
narsad

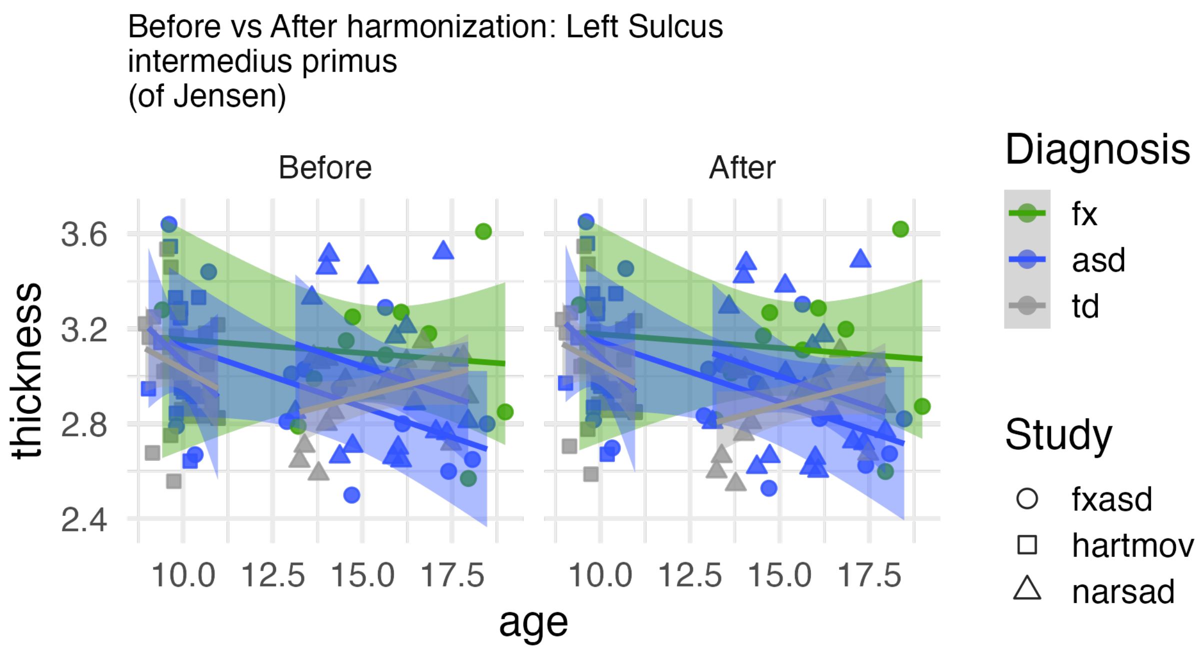
Before vs After harmonization: Left Sulcus intermedius primus (of Jensen)
Before
After
thickness
3.6
3.2
2.8
2.4
10.0
12.5
15.0
17.5
age
Diagnosis
fx
asd
td
Study
fxasd
hartmov
narsad

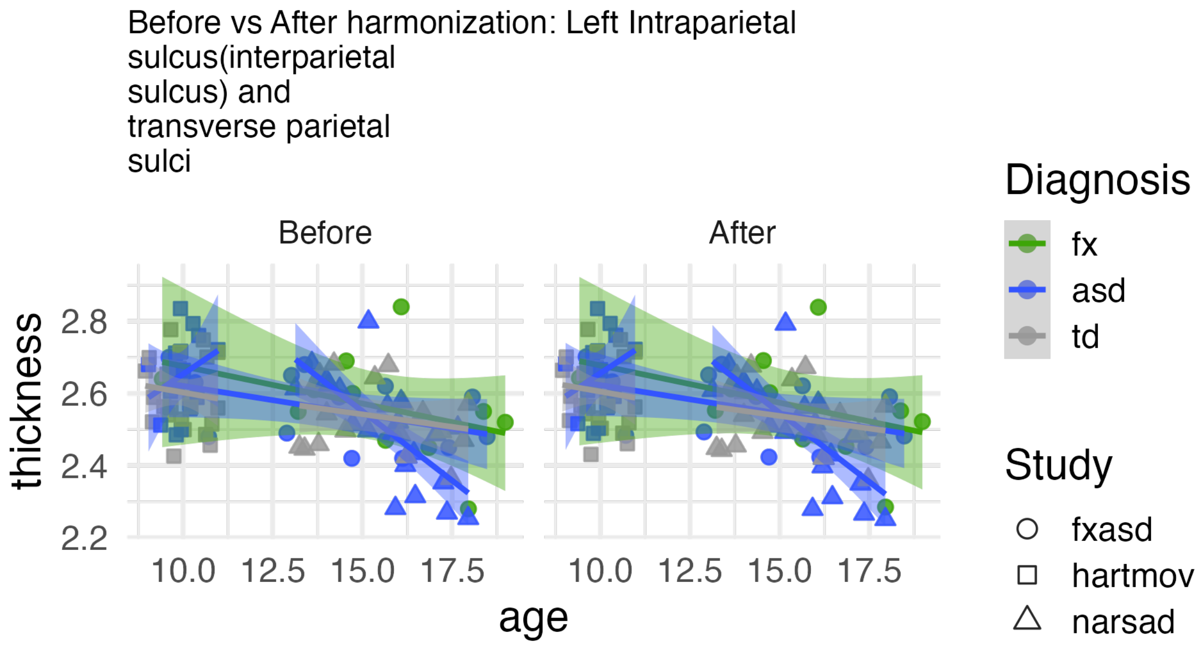
Before vs After harmonization: Left Intraparietal sulcus(interparietal sulcus) and transverse parietal sulci
Before
After
thickness
2.8
2.6
2.4
2.2
10.0
12.5
15.0
17.5
age
Diagnosis
fx
asd
td
Study
fxasd
hartmov
narsad

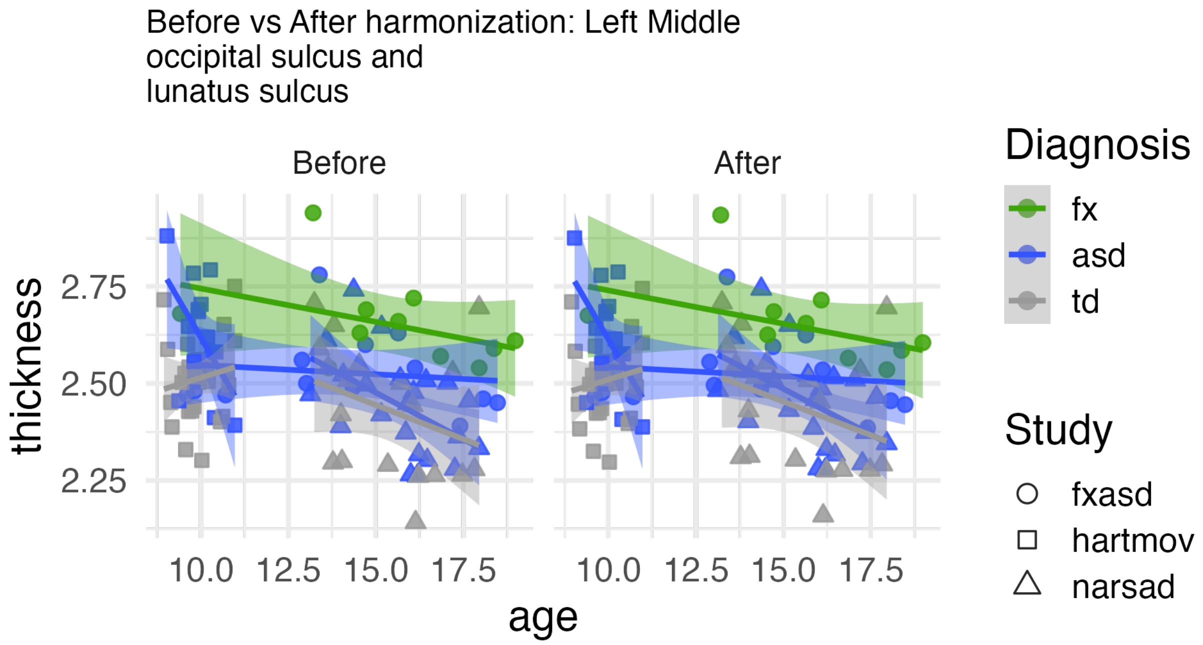
Before vs After harmonization: Left Middle
occipital sulcus and
lunatus sulcus
Before
After
thickness
2.75
2.50
2.25
10.0
12.5
15.0
17.5
age
Diagnosis
fx
asd
td
Study
fxasd
hartmov
narsad

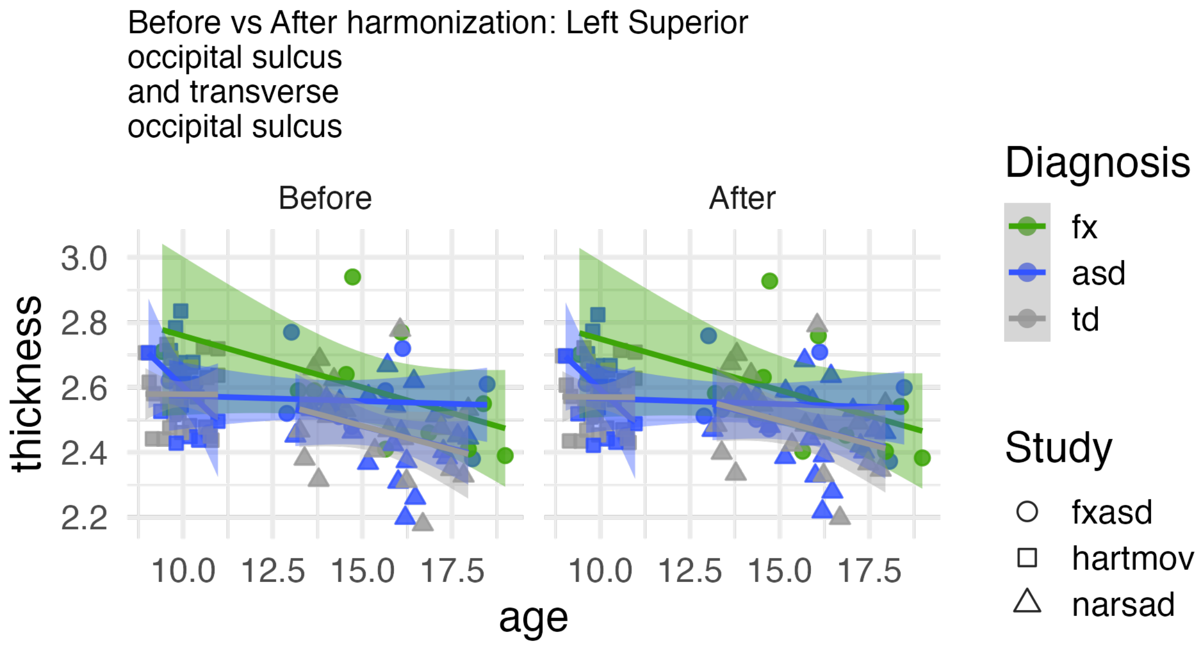
Before vs After harmonization: Left Superior
occipital sulcus
and transverse
occipital sulcus
Before
After
thickness
3.0
2.8
2.6
2.4
2.2
10.0
12.5
15.0
17.5
age
Diagnosis
fx
asd
td
Study
fxasd
hartmov
narsad

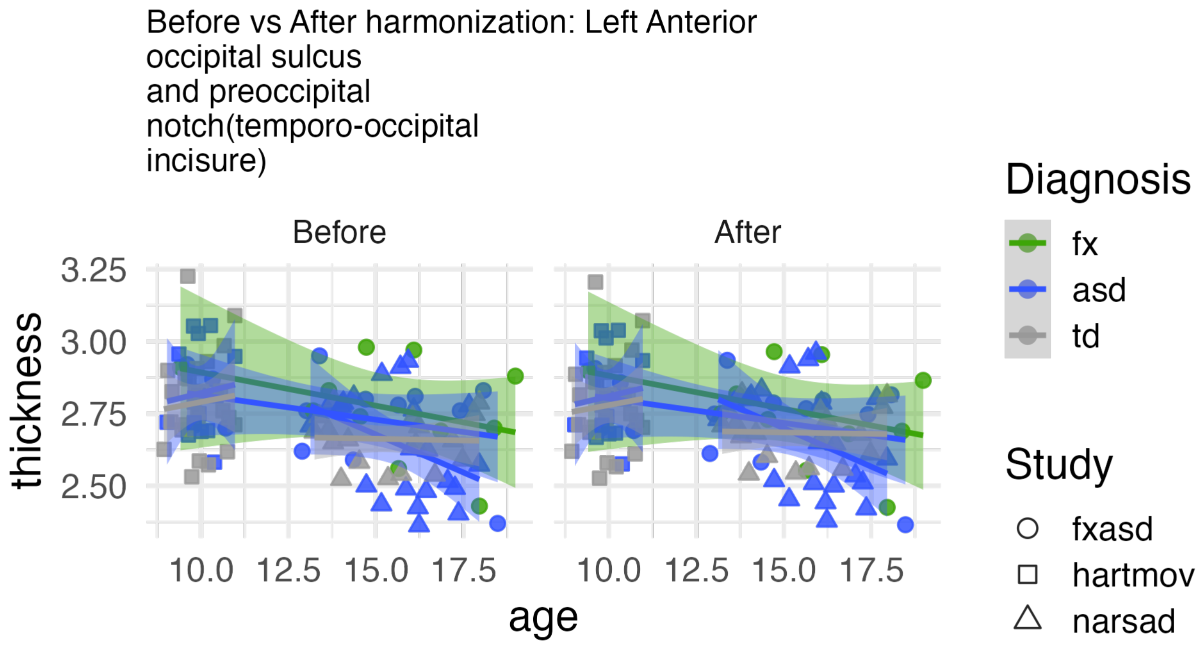
Before vs After harmonization: Left Anterior
occipital sulcus
and preoccipital
notch(temporo-occipital
incisure)
Before
After
thickness
3.25
3.00
2.75
2.50
10.0
12.5
15.0
17.5
age
Diagnosis
fx
asd
td
Study
fxasd
hartmov
narsad

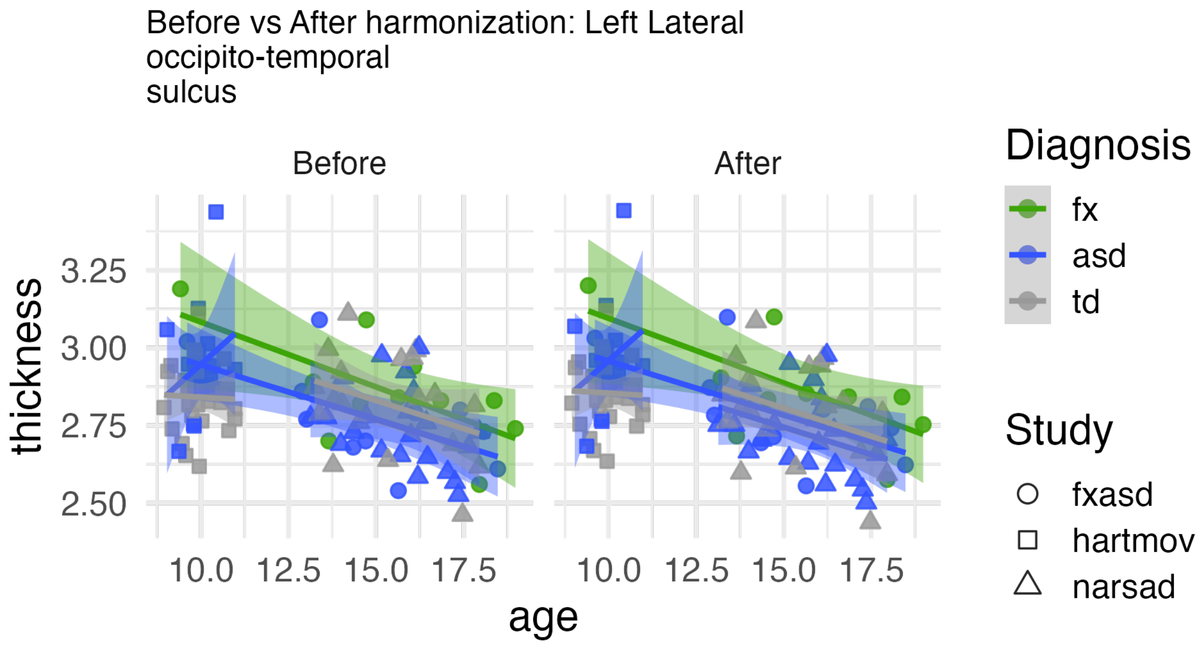
Before vs After harmonization: Left Lateral
occipito-temporal
sulcus
Before
After
thickness
3.25
3.00
2.75
2.50
10.0
12.5
15.0
17.5
age
Diagnosis
fx
asd
td
Study
fxasd
hartmov
narsad

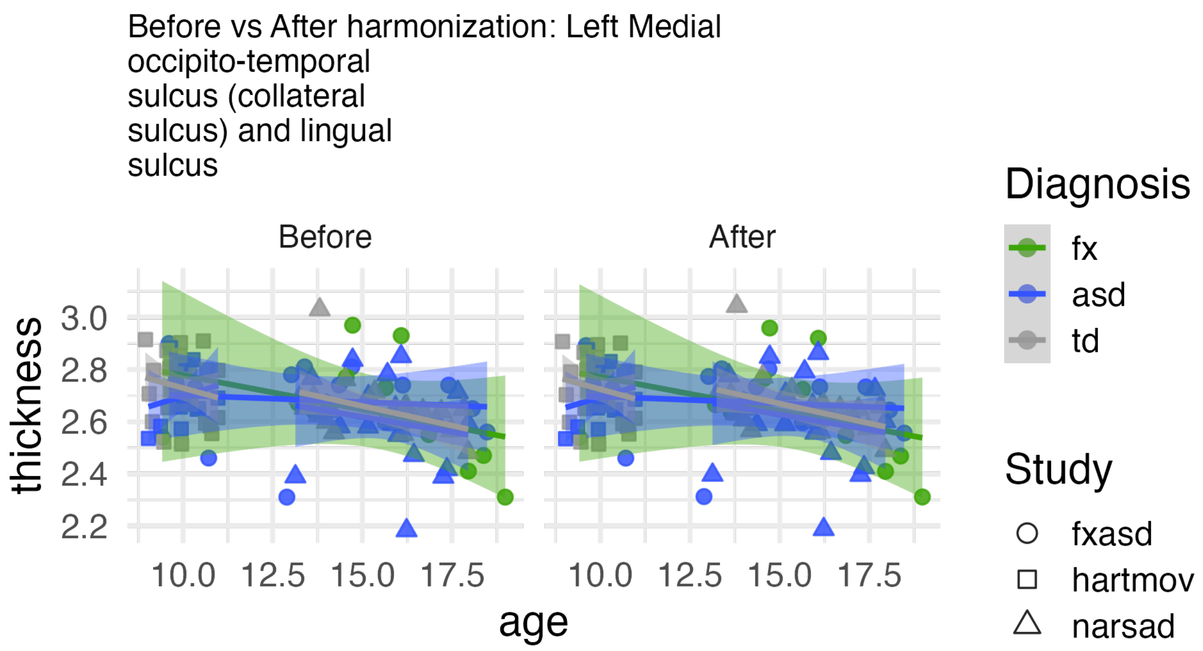
Before vs After harmonization: Left Medial occipito-temporal sulcus (collateral sulcus) and lingual sulcus
Before
After
thickness
3.0
2.8
2.6
2.4
2.2
10.0
12.5
15.0
17.5
age
Diagnosis
fx
asd
td
Study
fxasd
hartmov
narsad

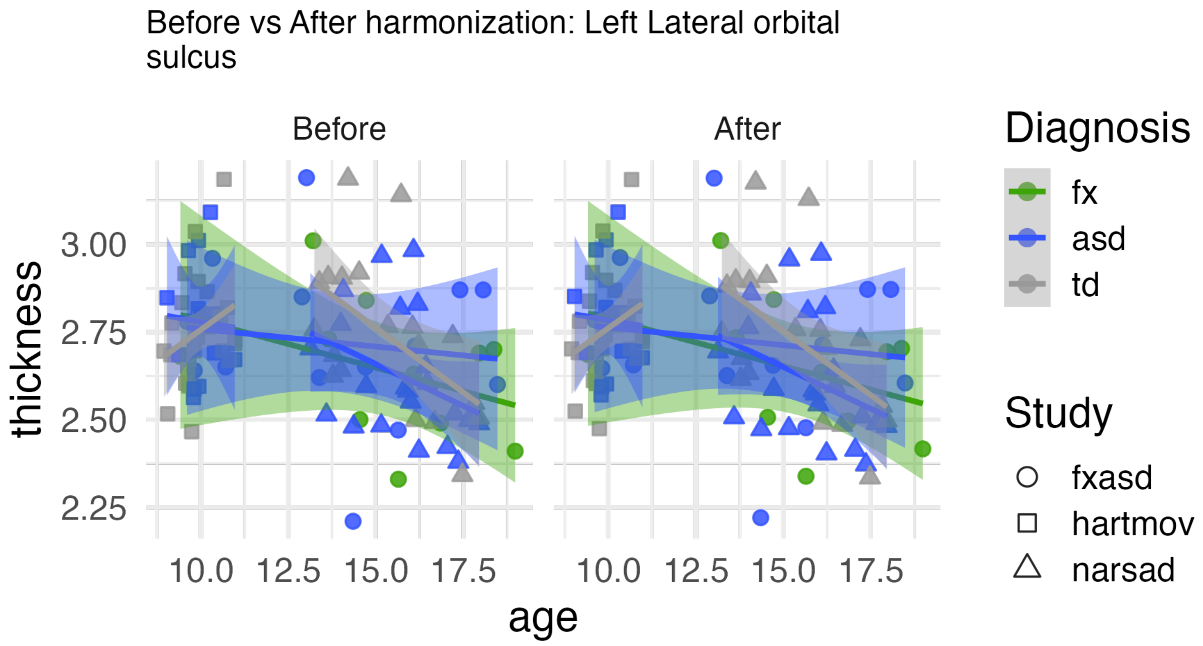
Before vs After harmonization: Left Lateral orbital sulcus
Before
After
thickness
3.00
2.75
2.50
2.25
10.0
12.5
15.0
17.5
age
Diagnosis
fx
asd
td
Study
fxasd
hartmov
narsad

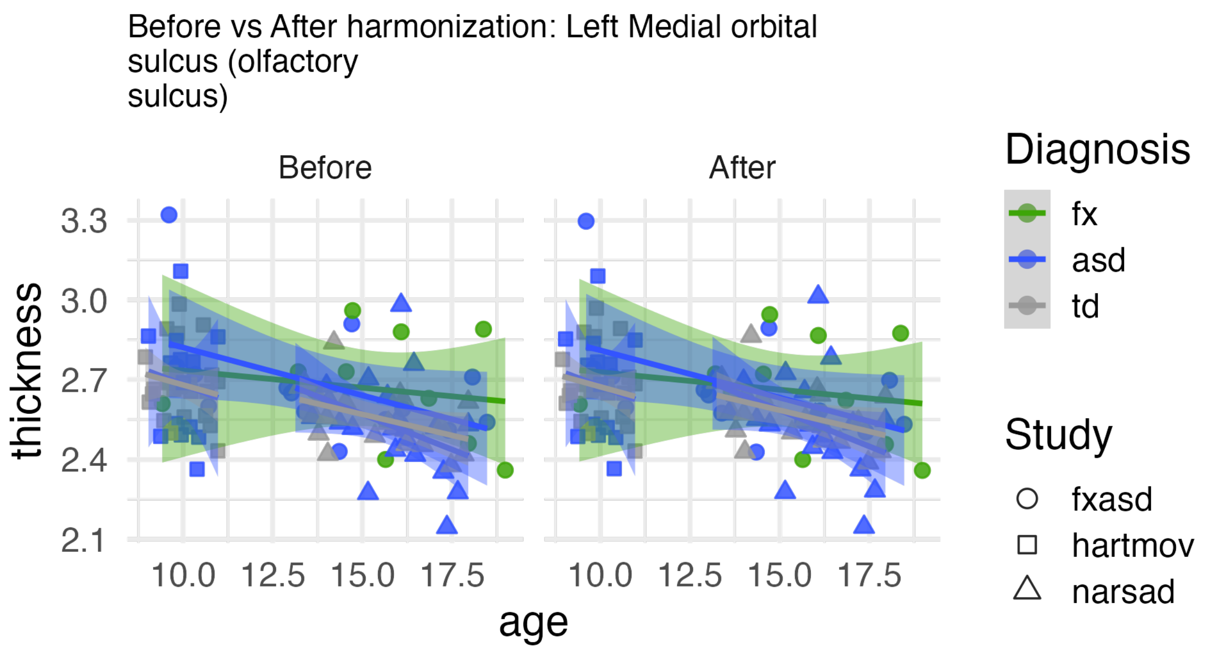

Before vs After harmonization: Left Medial orbital
sulcus (olfactory
sulcus)
Before
After
3.3
3.0
2.7
2.4
2.1
thickness
10.0
12.5
15.0
17.5
age
Diagnosis
fx
asd
td
Study
fxasd
hartmov
narsad

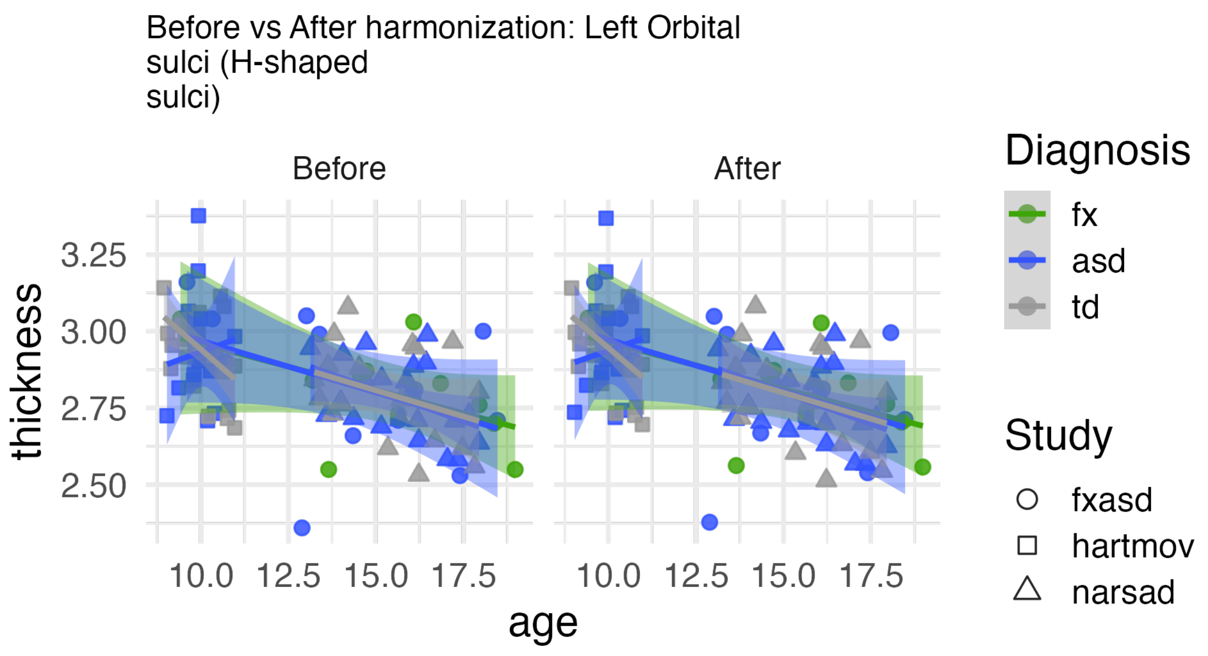

Before vs After harmonization: Left Orbital
sulci (H-shaped
sulci)
Before
After
3.25
3.00
2.75
2.50
thickness
10.0
12.5
15.0
17.5
age
Diagnosis
fx
asd
td
Study
fxasd
hartmov
narsad

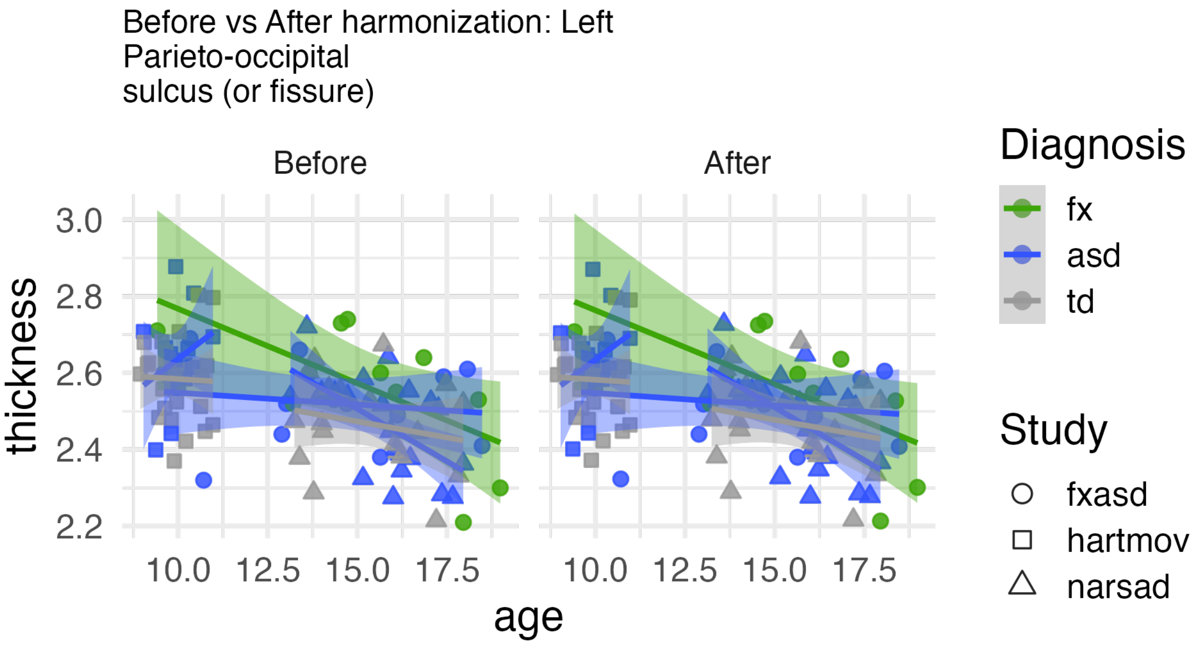

Before vs After harmonization: Left Parieto-occipital sulcus (or fissure)
Before
After
thickness
3.0
2.8
2.6
2.4
2.2
10.0
12.5
15.0
17.5
age
Diagnosis
fx
asd
td
Study
fxasd
hartmov
narsad

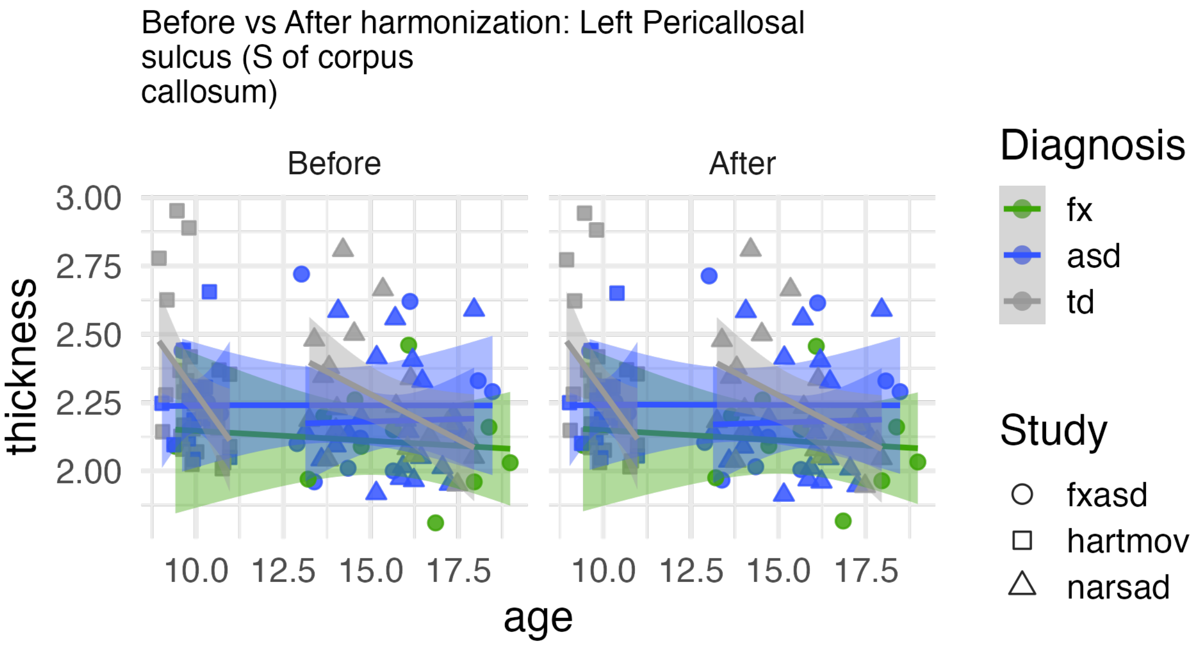

Before vs After harmonization: Left Pericallosal sulcus (S of corpus callosum)
Before
After
thickness
3.00
2.75
2.50
2.25
2.00
10.0
12.5
15.0
17.5
age
Diagnosis
fx
asd
td
Study
fxasd
hartmov
narsad

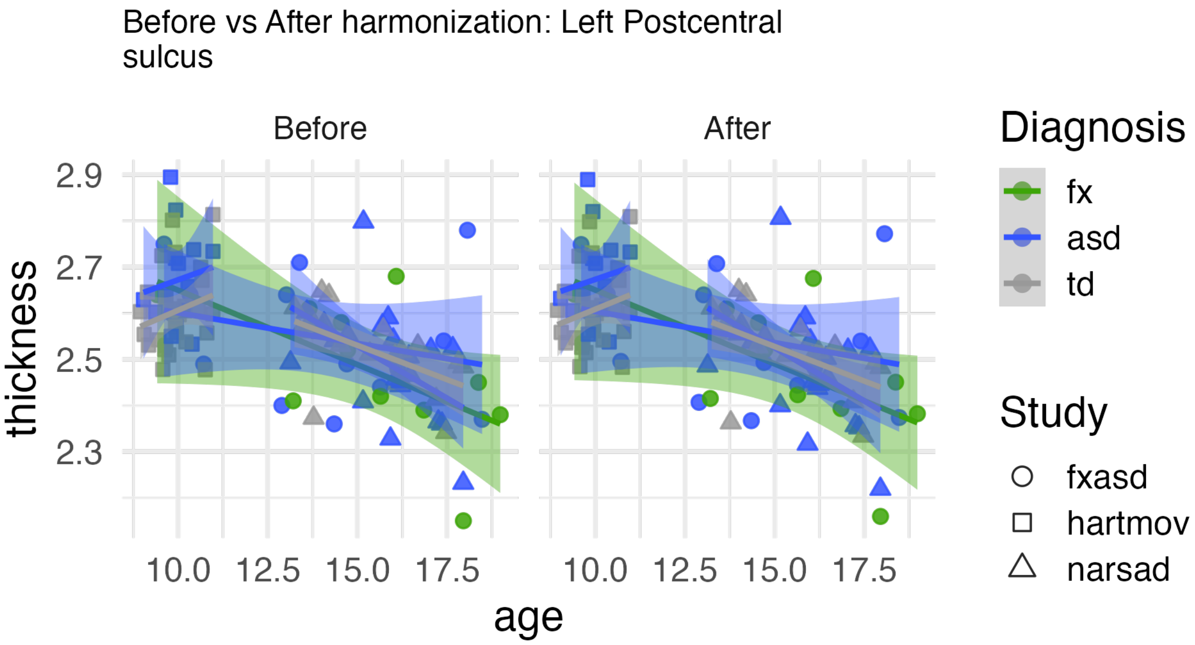
Before vs After harmonization: Left Postcentral
sulcus
Before
After
2.9
2.7
2.5
2.3
thickness
10.0
12.5
15.0
17.5
age
Diagnosis
fx
asd
td
Study
fxasd
hartmov
narsad

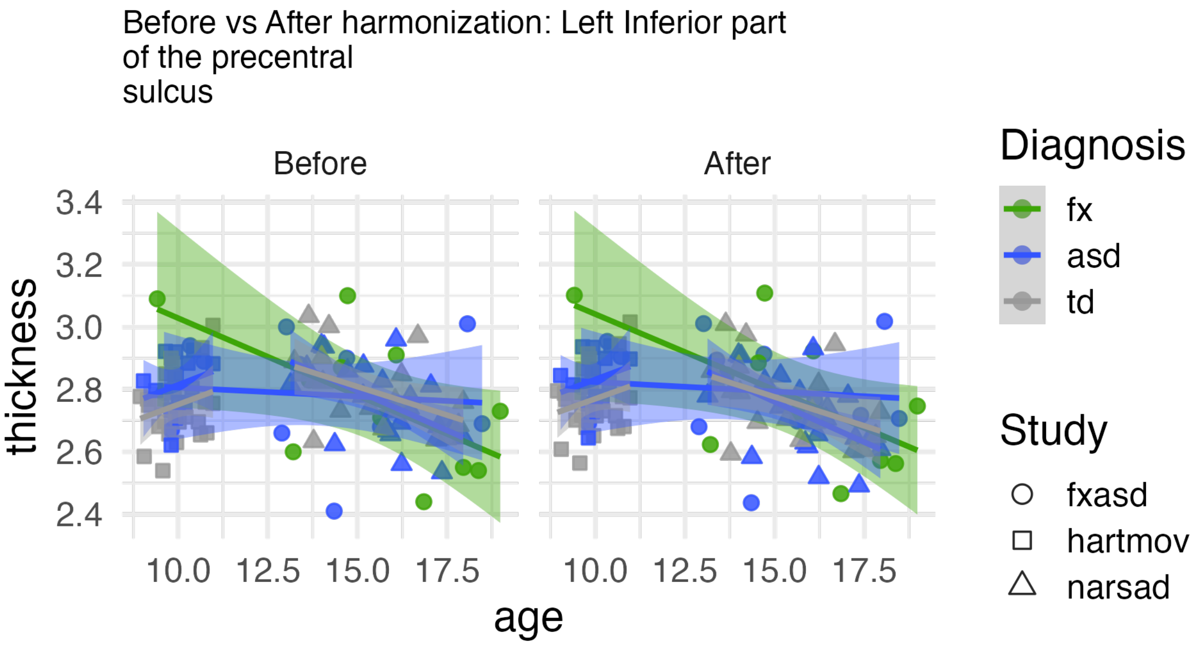
Before vs After harmonization: Left Inferior part
of the precentral
sulcus
Before
After
3.4
3.2
3.0
2.8
2.6
2.4
thickness
10.0
12.5
15.0
17.5
age
Diagnosis
fx
asd
td
Study
fxasd
hartmov
narsad

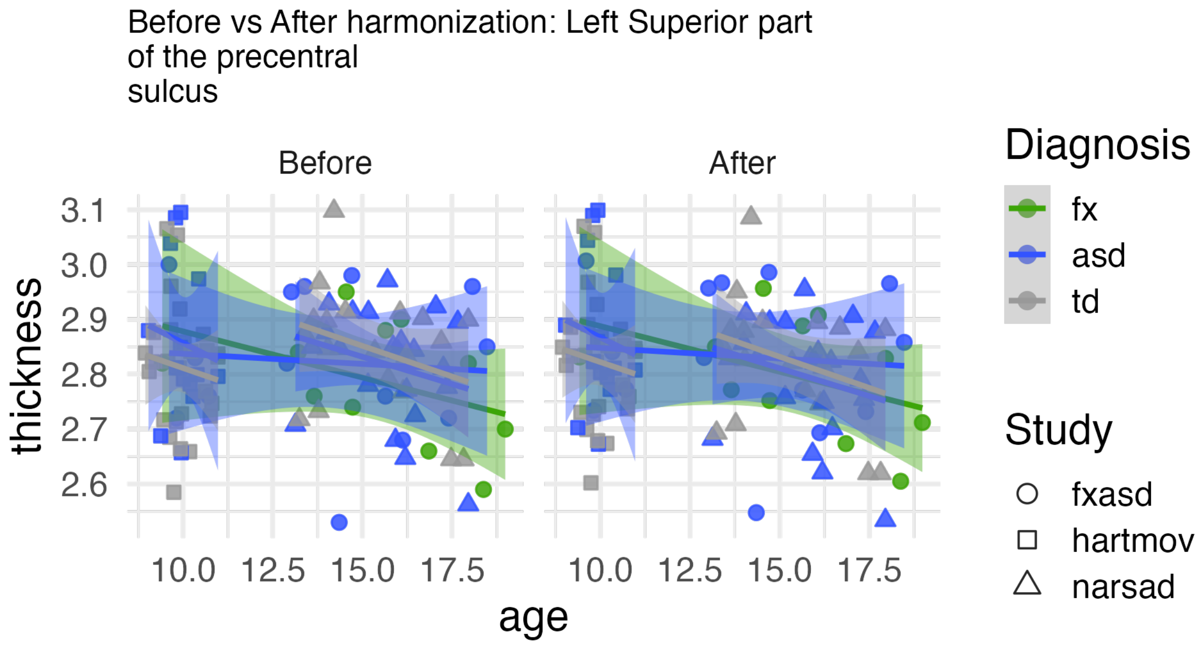
Before vs After harmonization: Left Superior part
of the precentral
sulcus
Before
After
thickness
3.1
3.0
2.9
2.8
2.7
2.6
10.0 12.5 15.0 17.5
age
Diagnosis
fx
asd
td
Study
fxasd
hartmov
narsad

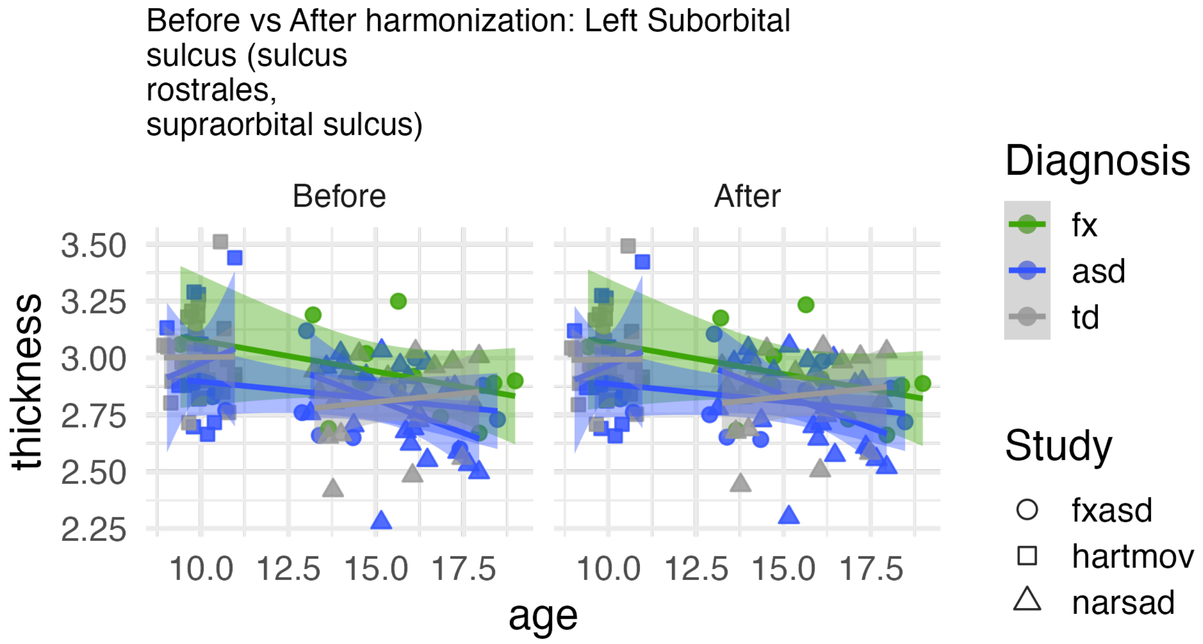
Before vs After harmonization: Left Suborbital
sulcus (sulcus
rostrales,
supraorbital sulcus)
Before
After
thickness
3.50
3.25
3.00
2.75
2.50
2.25
10.0 12.5 15.0 17.5
age
Diagnosis
fx
asd
td
Study
fxasd
hartmov
narsad

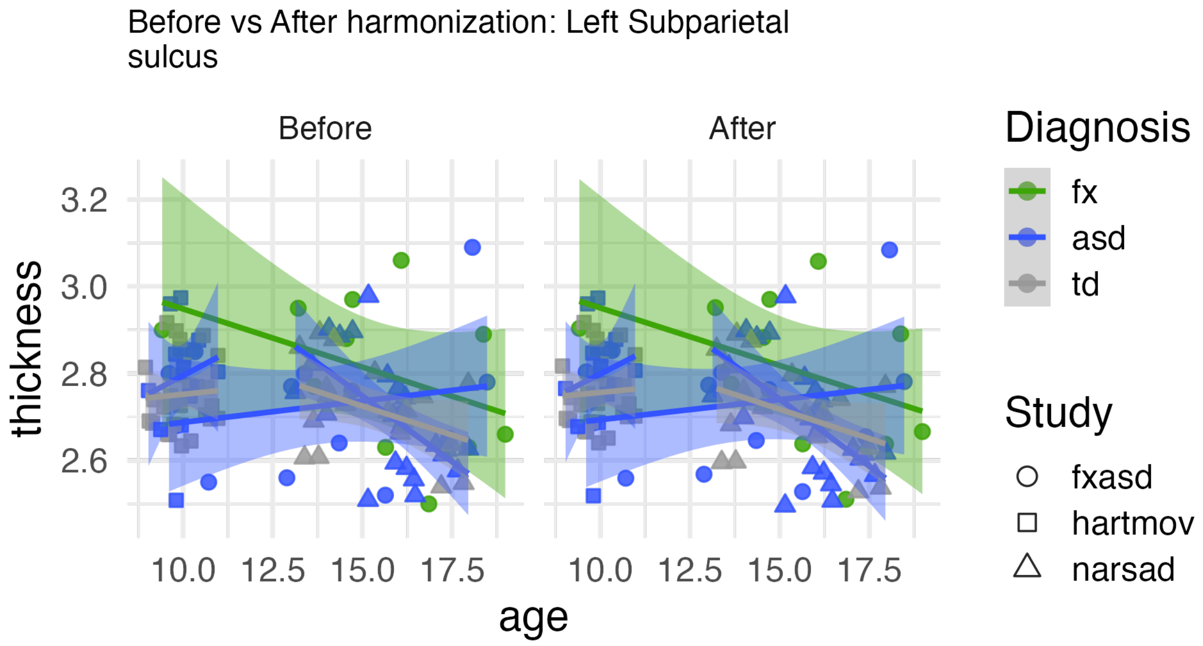
Before vs After harmonization: Left Subparietal sulcus
Before
After
thickness
3.2
3.0
2.8
2.6
10.0
12.5
15.0
17.5
age
Diagnosis
fx
asd
td
Study
fxasd
hartmov
narsad

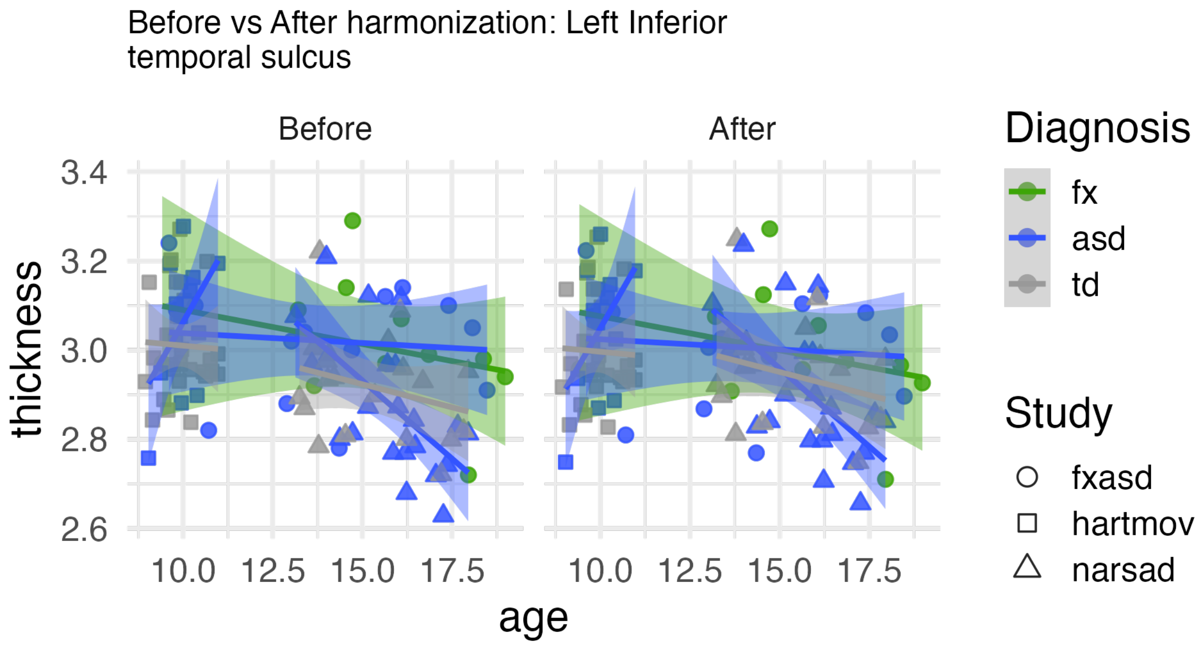
Before vs After harmonization: Left Inferior temporal sulcus
Before
After
thickness
3.4
3.2
3.0
2.8
2.6
10.0
12.5
15.0
17.5
age
Diagnosis
fx
asd
td
Study
fxasd
hartmov
narsad

Before vs After harmonization: Left Superior
temporal
sulcus (parallel
sulcus)

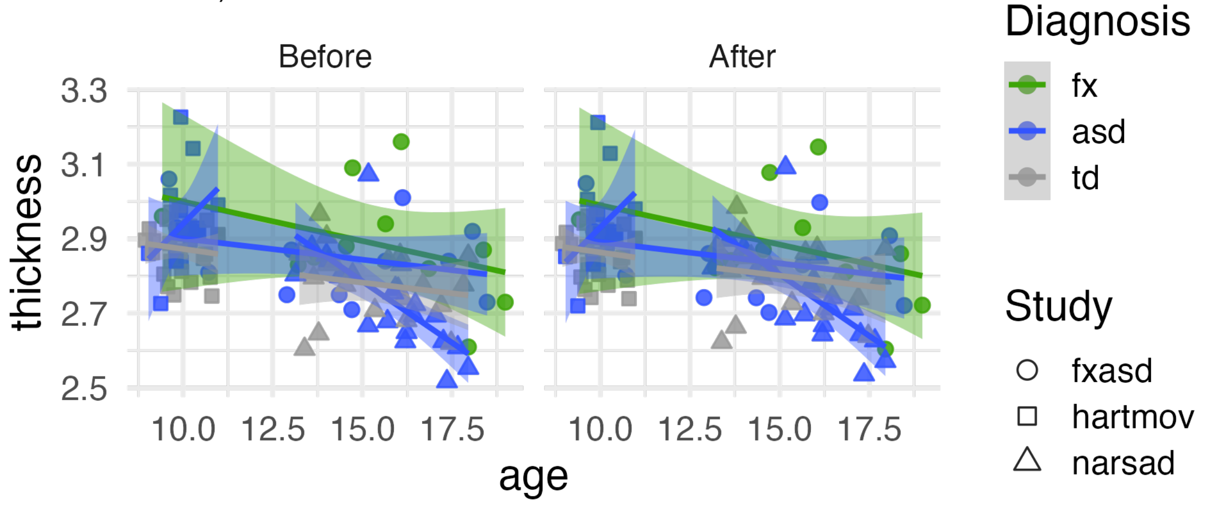

Before vs After harmonization: Left Transverse
temporal sulcus

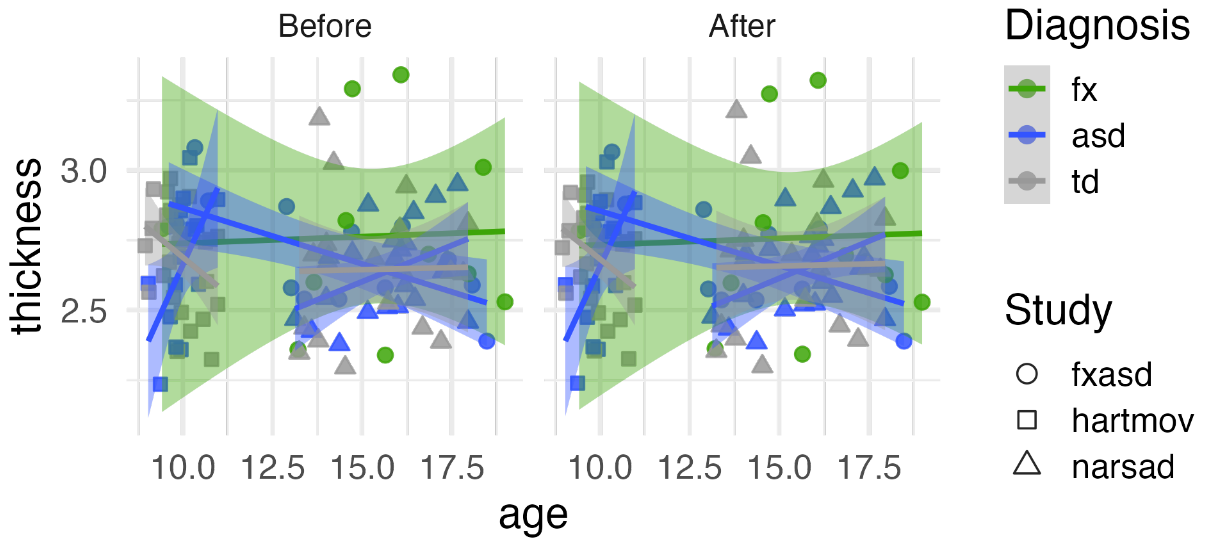

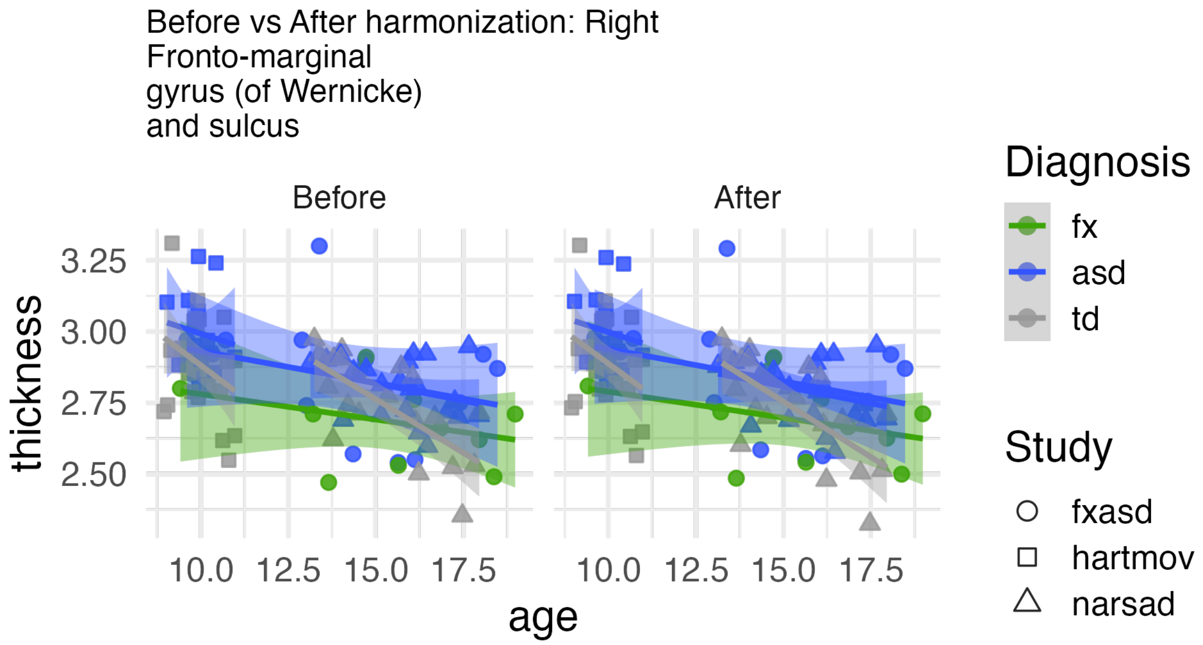
Before vs After harmonization: Right
Fronto-marginal
gyrus (of Wernicke)
and sulcus
Before
After
thickness
3.25
3.00
2.75
2.50
10.0
12.5
15.0
17.5
age
Diagnosis
fx
asd
td
Study
fxasd
hartmov
narsad

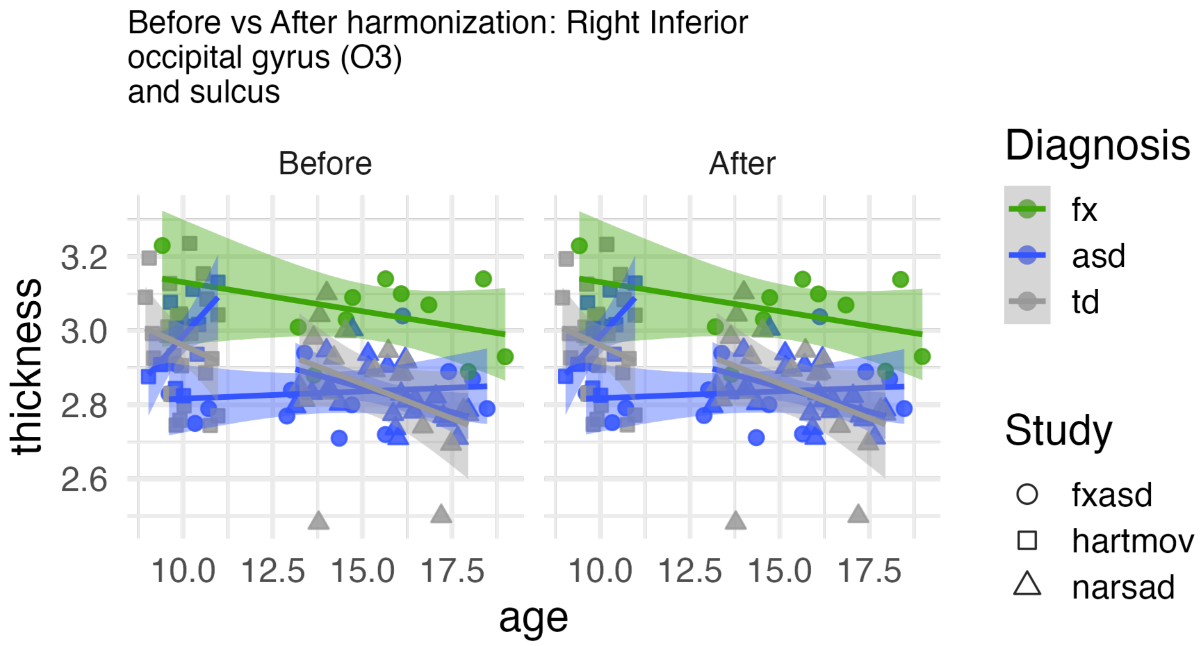
Before vs After harmonization: Right Inferior
occipital gyrus (O3)
and sulcus
Before
After
thickness
3.2
3.0
2.8
2.6
10.0
12.5
15.0
17.5
age
Diagnosis
fx
asd
td
Study
fxasd
hartmov
narsad

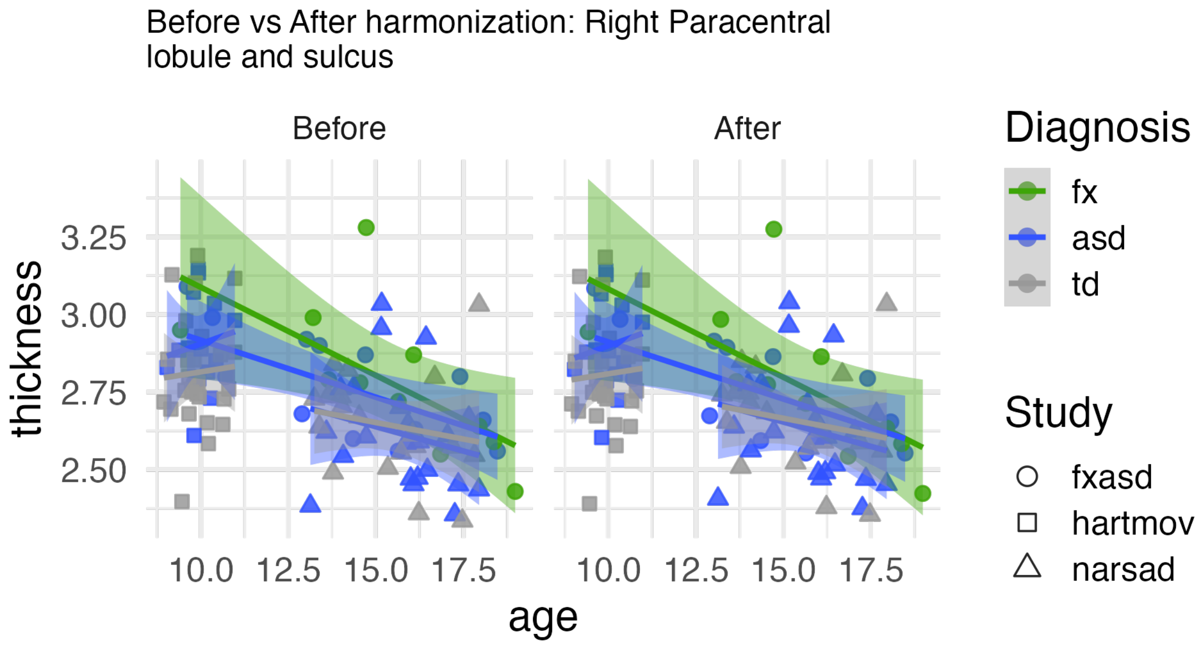
Before vs After harmonization: Right Paracentral lobule and sulcus
Before
After
thickness
3.25
3.00
2.75
2.50
10.0
12.5
15.0
17.5
age
Diagnosis
fx
asd
td
Study
fxasd
hartmov
narsad

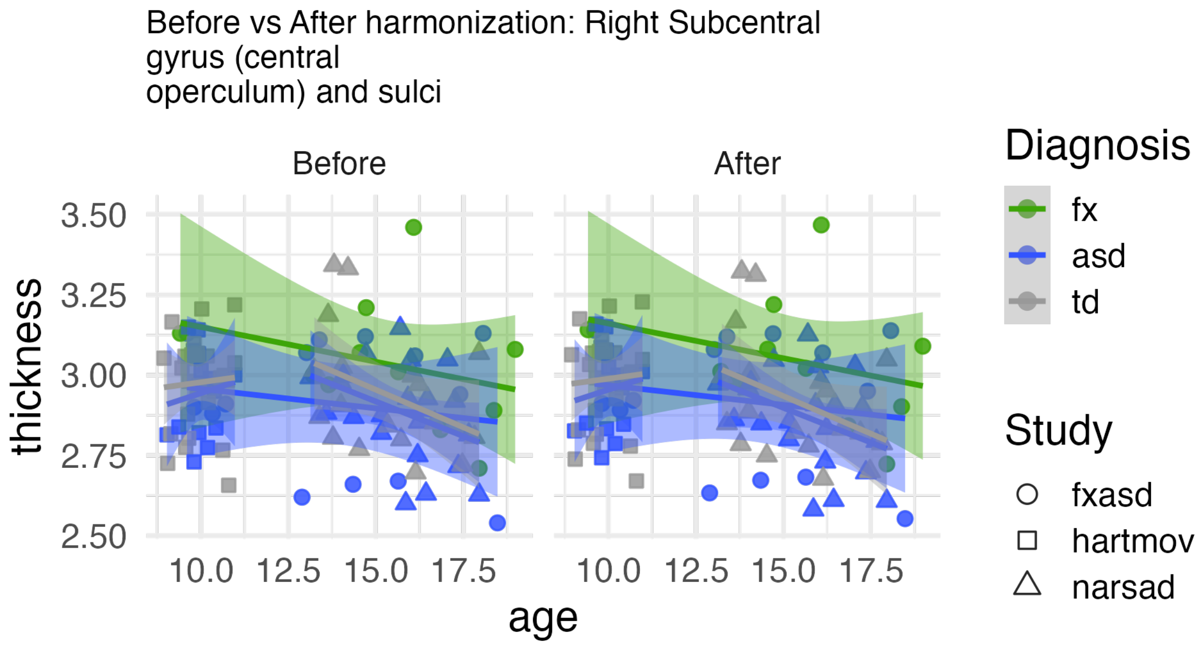
Before vs After harmonization: Right Subcentral gyrus (central operculum) and sulci
Before
After
thickness
3.50
3.25
3.00
2.75
2.50
10.0
12.5
15.0
17.5
age
Diagnosis
fx
asd
td
Study
fxasd
hartmov
narsad

Before vs After harmonization: Right Transverse
frontopolar gyri and
sulci

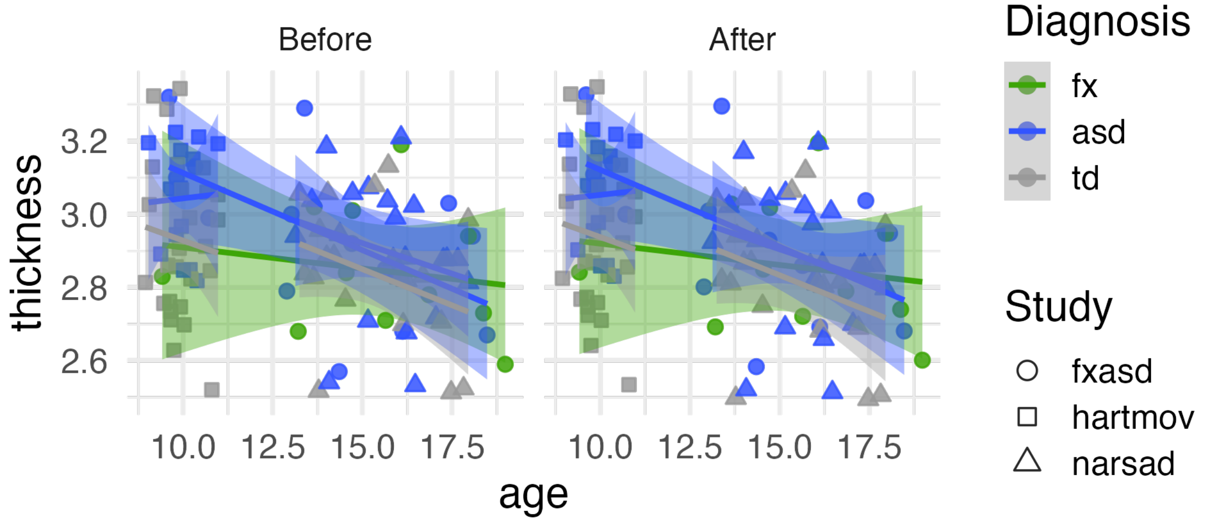

Before vs After harmonization: Right Anterior part
of the cingulate
gyrus and
sulcus (ACC)

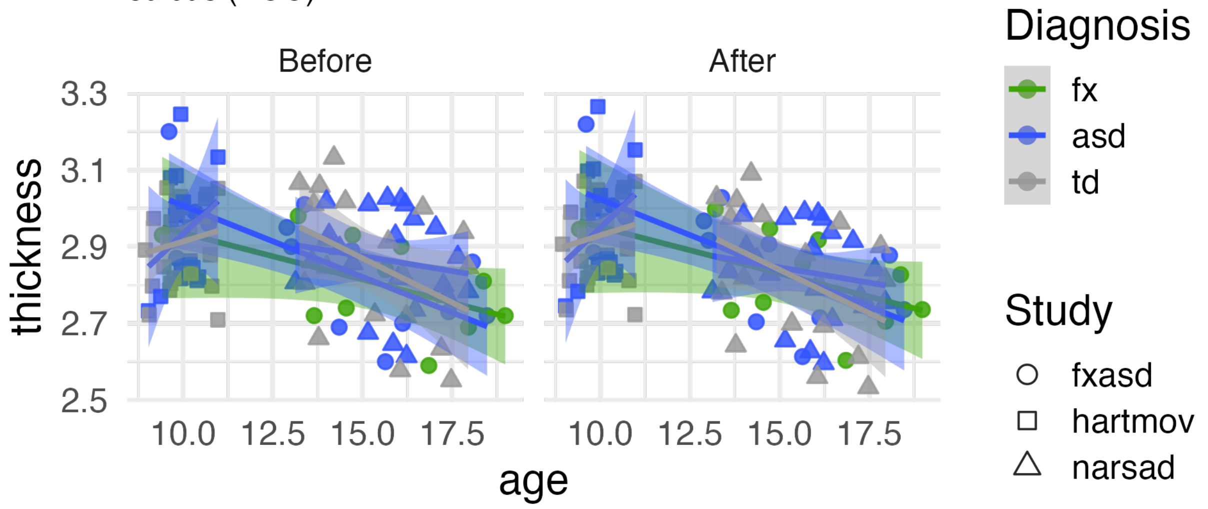

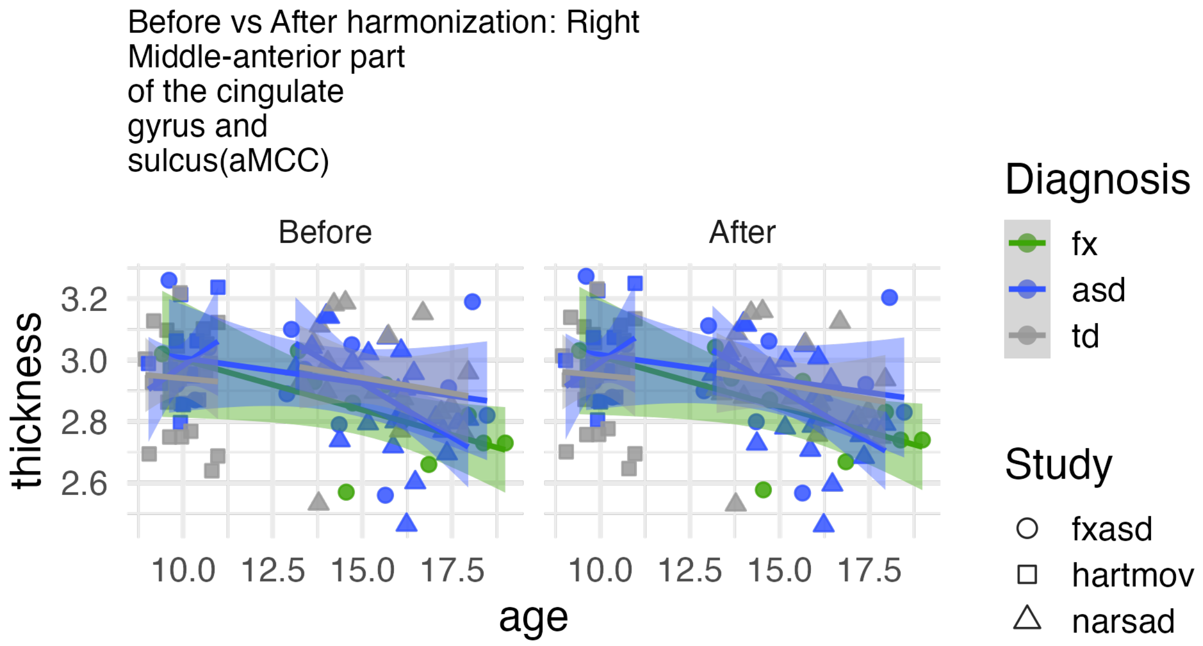
Before vs After harmonization: Right
Middle-anterior part
of the cingulate
gyrus and
sulcus(aMCC)
Before
After
thickness
3.2
3.0
2.8
2.6
10.0
12.5
15.0
17.5
age
Diagnosis
fx
asd
td
Study
fxasd
hartmov
narsad

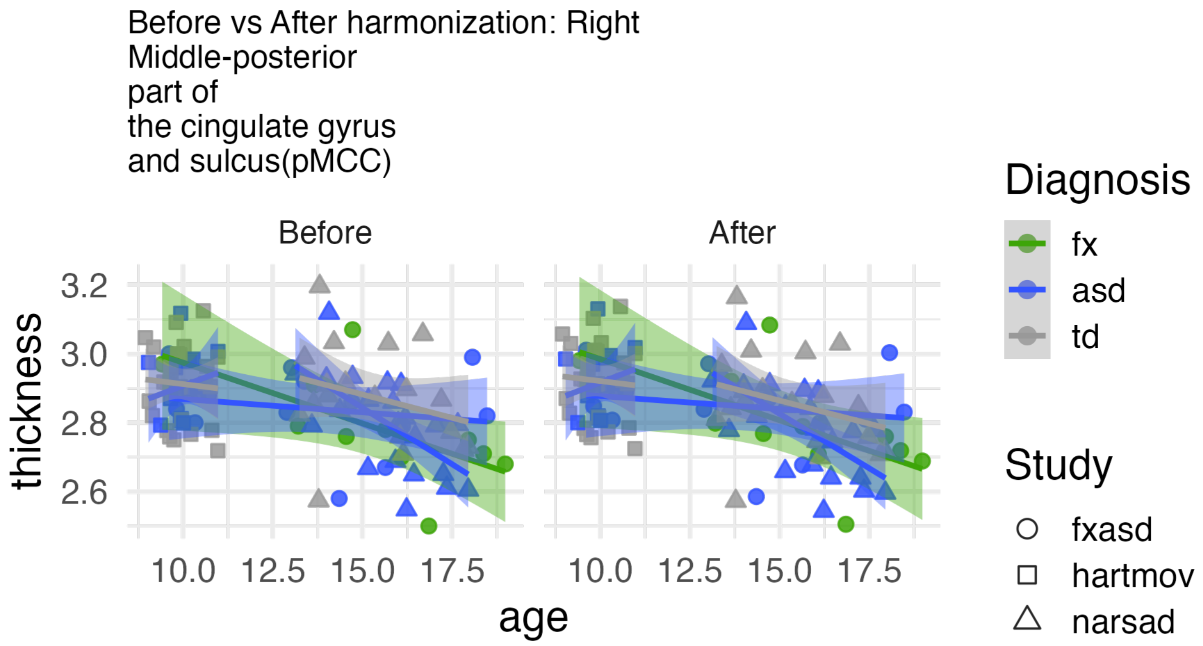
Before vs After harmonization: Right
Middle-posterior
part of
the cingulate gyrus
and sulcus(pMCC)
Before
After
thickness
3.2
3.0
2.8
2.6
10.0
12.5
15.0
17.5
age
Diagnosis
fx
asd
td
Study
fxasd
hartmov
narsad

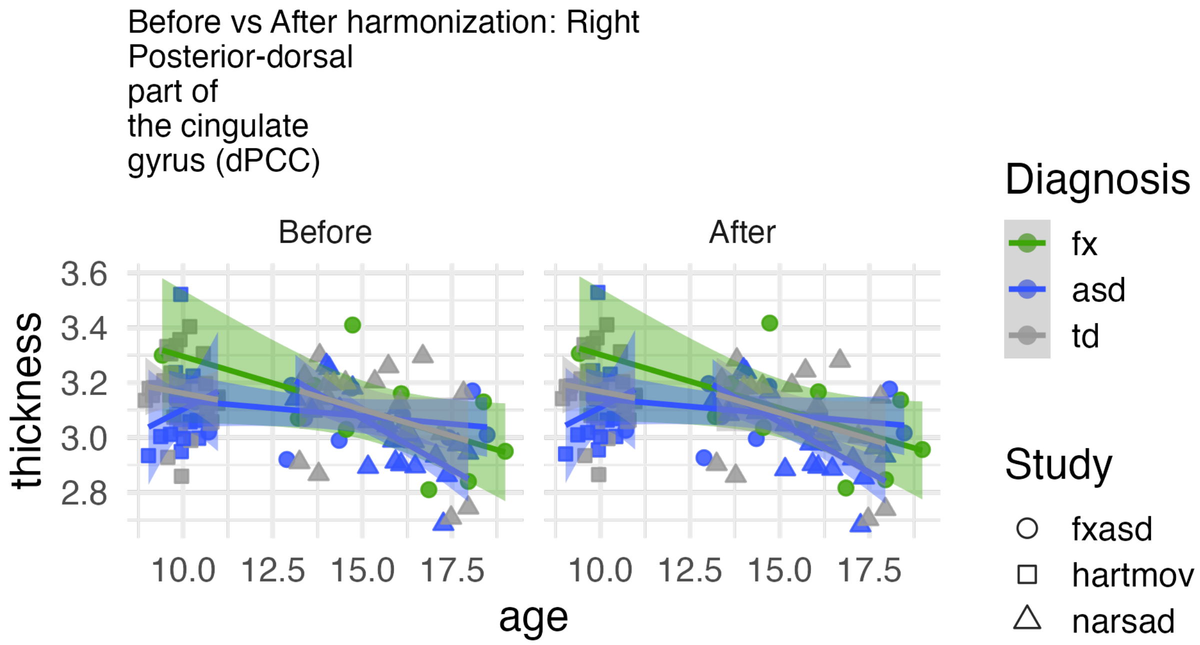
Before vs After harmonization: Right
Posterior-dorsal
part of
the cingulate
gyrus (dPCC)
Before
After
thickness
3.6
3.4
3.2
3.0
2.8
10.0
12.5
15.0
17.5
age
Diagnosis
fx
asd
td
Study
fxasd
hartmov
narsad

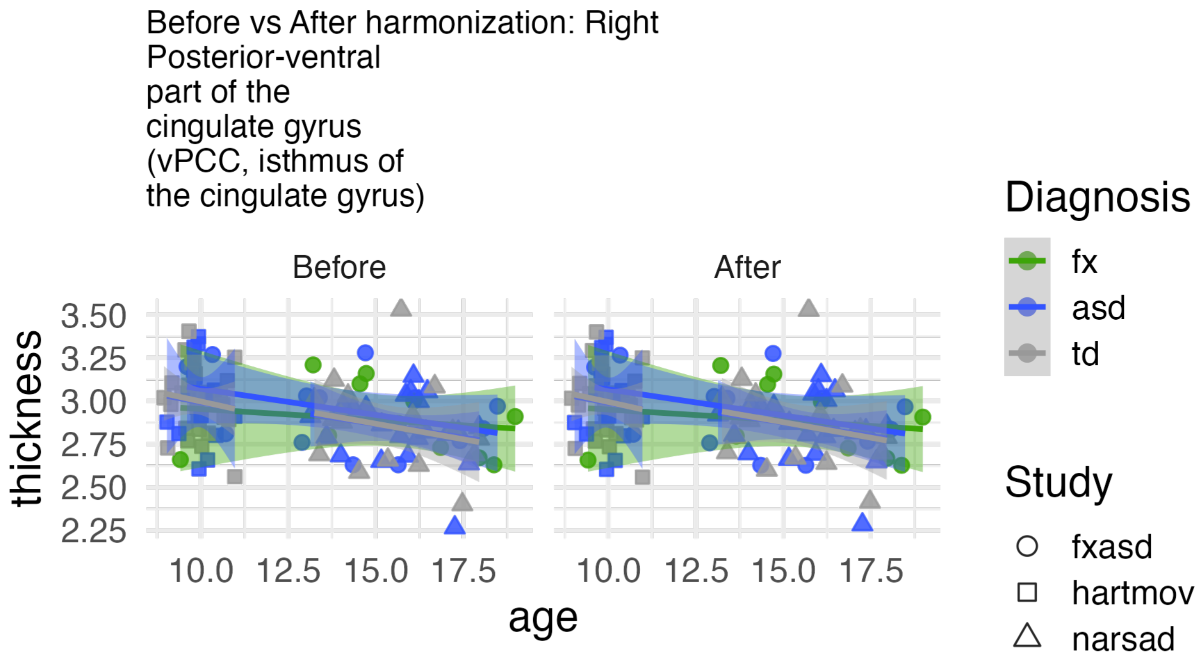
Before vs After harmonization: Right
Posterior-ventral
part of the
cingulate gyrus
(vPCC, isthmus of
the cingulate gyrus)
Before
After
thickness
3.50
3.25
3.00
2.75
2.50
2.25
10.0
12.5
15.0
17.5
age
Diagnosis
fx
asd
td
Study
fxasd
hartmov
narsad

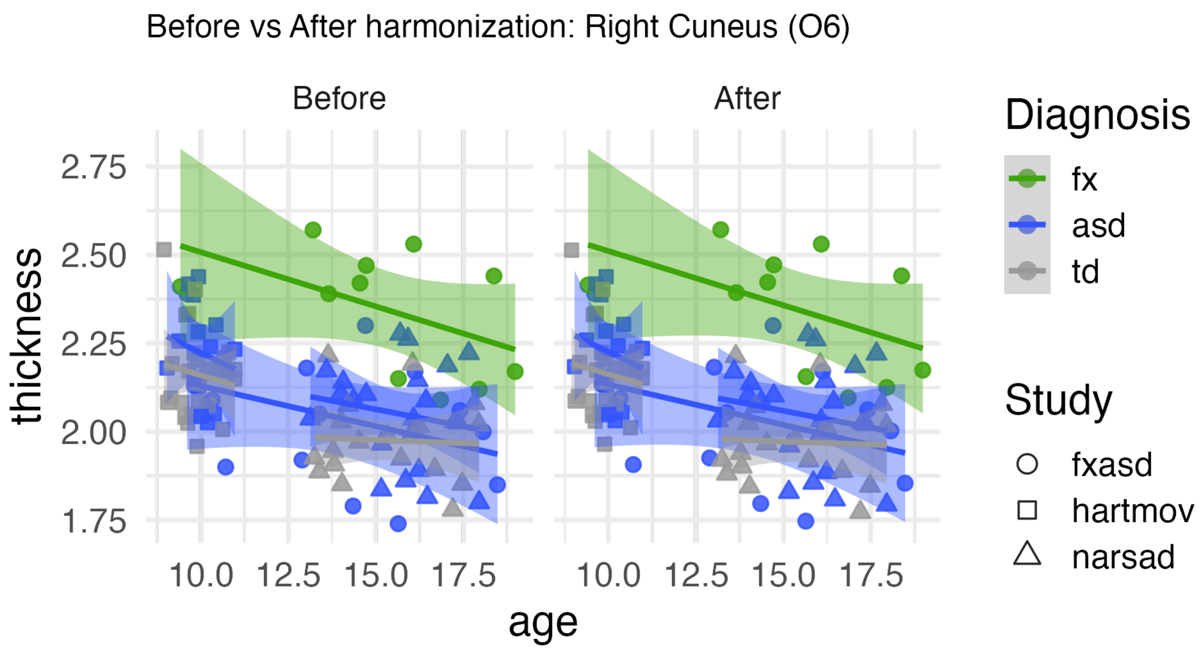

Before vs After harmonization: Right Cuneus (O6)
Before
After
thickness
2.75
2.50
2.25
2.00
1.75
10.0 12.5 15.0 17.5
10.0 12.5 15.0 17.5
age
Diagnosis
fx
asd
td
Study
fxasd
hartmov
narsad

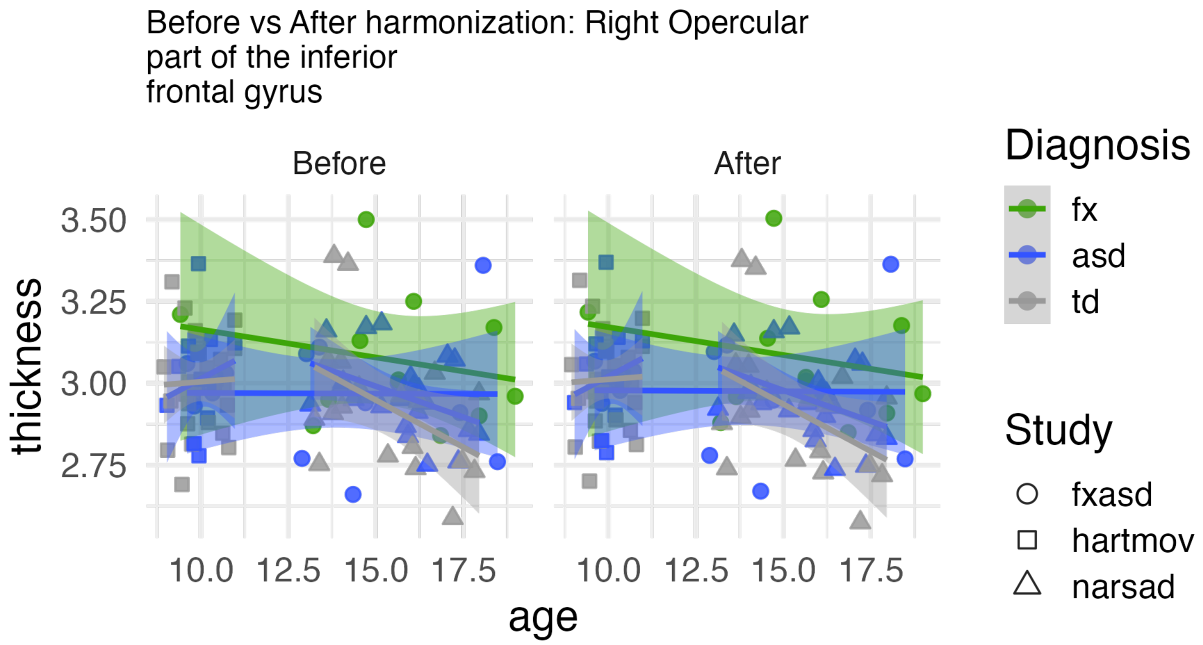

Before vs After harmonization: Right Opercular
part of the inferior
frontal gyrus
Before
After
thickness
3.50
3.25
3.00
2.75
10.0 12.5 15.0 17.5
10.0 12.5 15.0 17.5
age
Diagnosis
fx
asd
td
Study
fxasd
hartmov
narsad

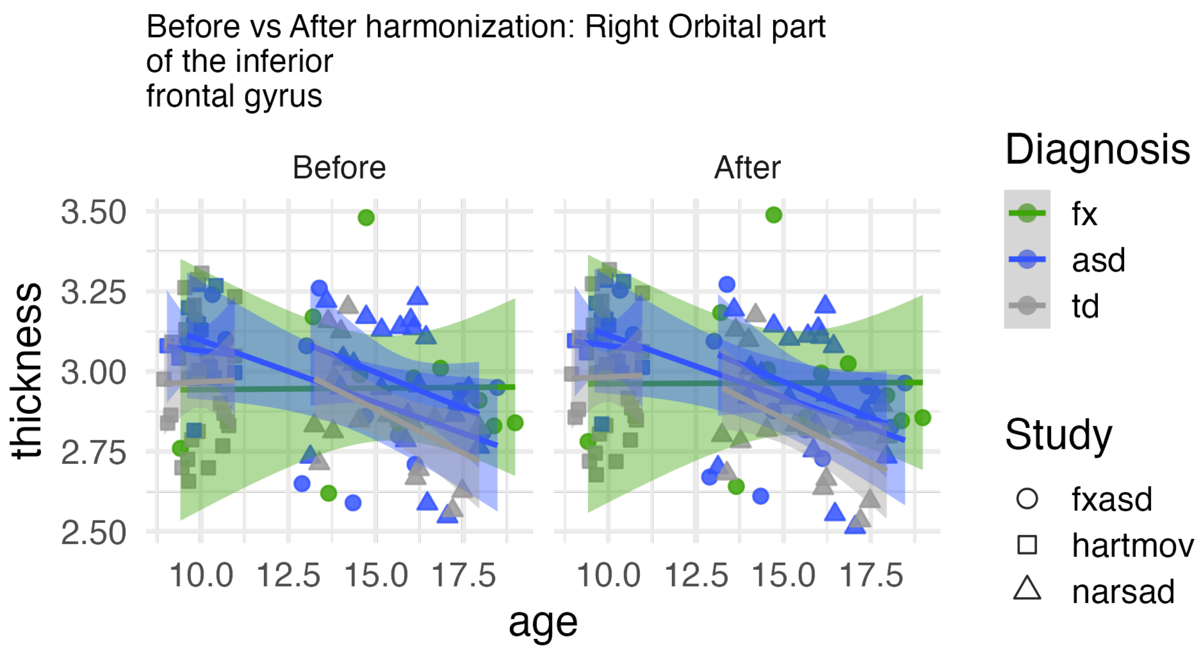

Before vs After harmonization: Right Orbital part
of the inferior
frontal gyrus
Before
After
thickness
3.50
3.25
3.00
2.75
2.50
10.0
12.5
15.0
17.5
age
Diagnosis
fx
asd
td
Study
fxasd
hartmov
narsad

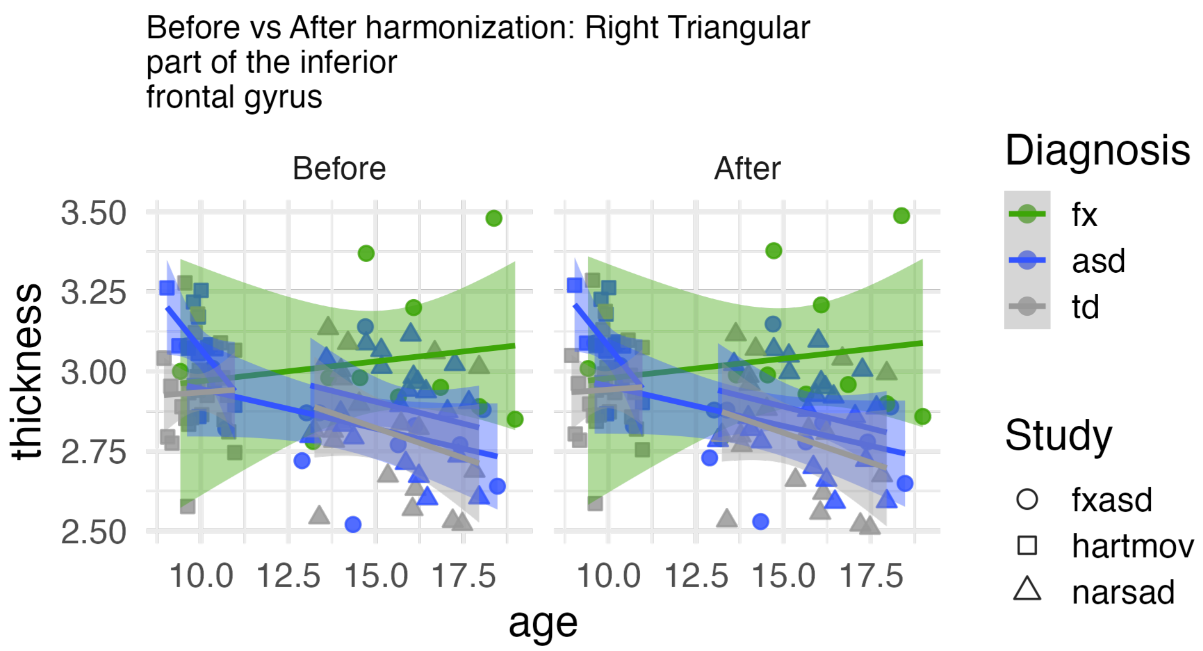

Before vs After harmonization: Right Triangular
part of the inferior
frontal gyrus
Before
After
thickness
3.50
3.25
3.00
2.75
2.50
10.0
12.5
15.0
17.5
age
Diagnosis
fx
asd
td
Study
fxasd
hartmov
narsad

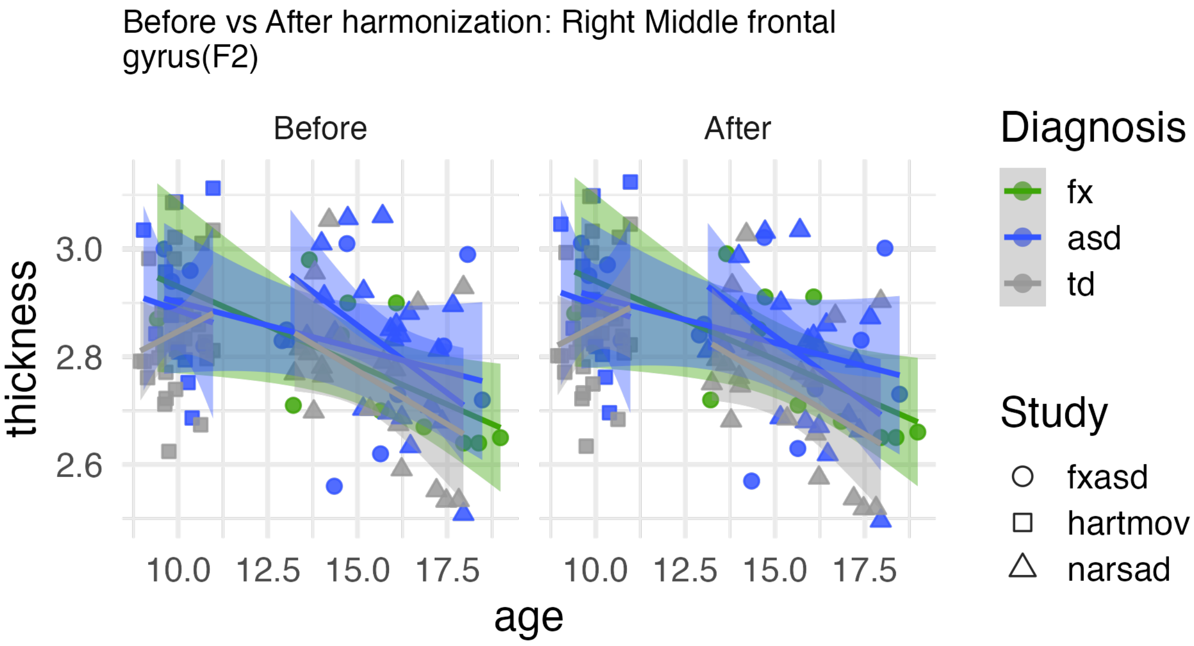
Before vs After harmonization: Right Middle frontal gyrus(F2)
Before
After
thickness
3.0
2.8
2.6
10.0
12.5
15.0
17.5
age
Diagnosis
fx
asd
td
Study
fxasd
hartmov
narsad

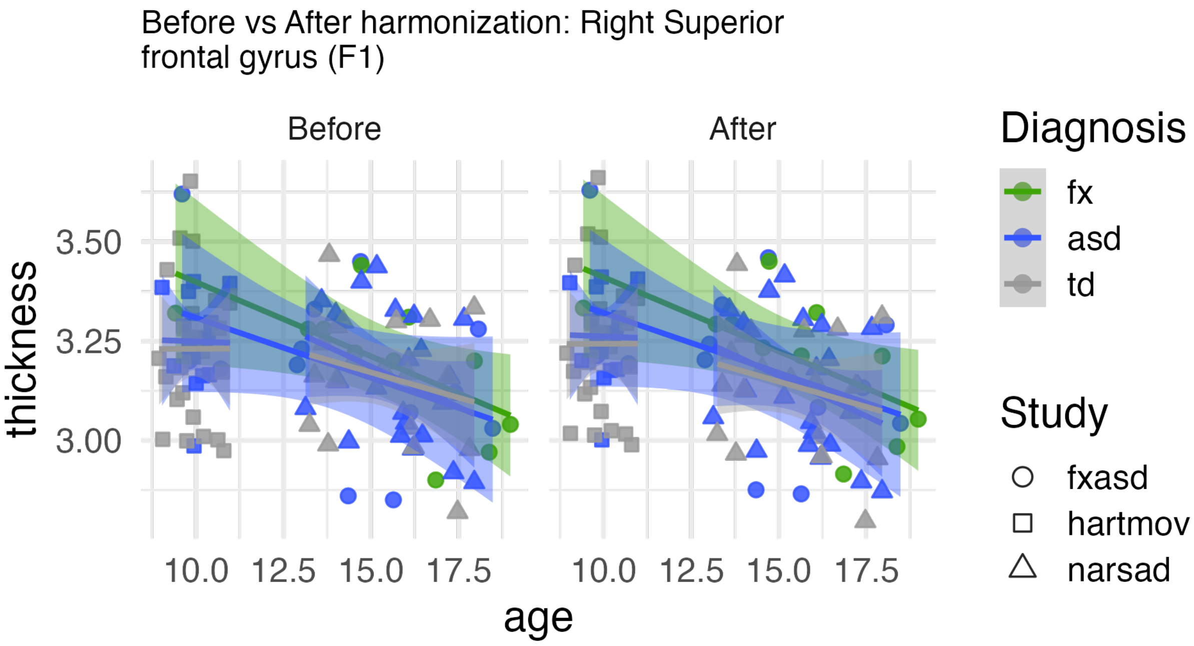
Before vs After harmonization: Right Superior frontal gyrus (F1)
Before
After
thickness
3.50
3.25
3.00
10.0
12.5
15.0
17.5
age
Diagnosis
fx
asd
td
Study
fxasd
hartmov
narsad

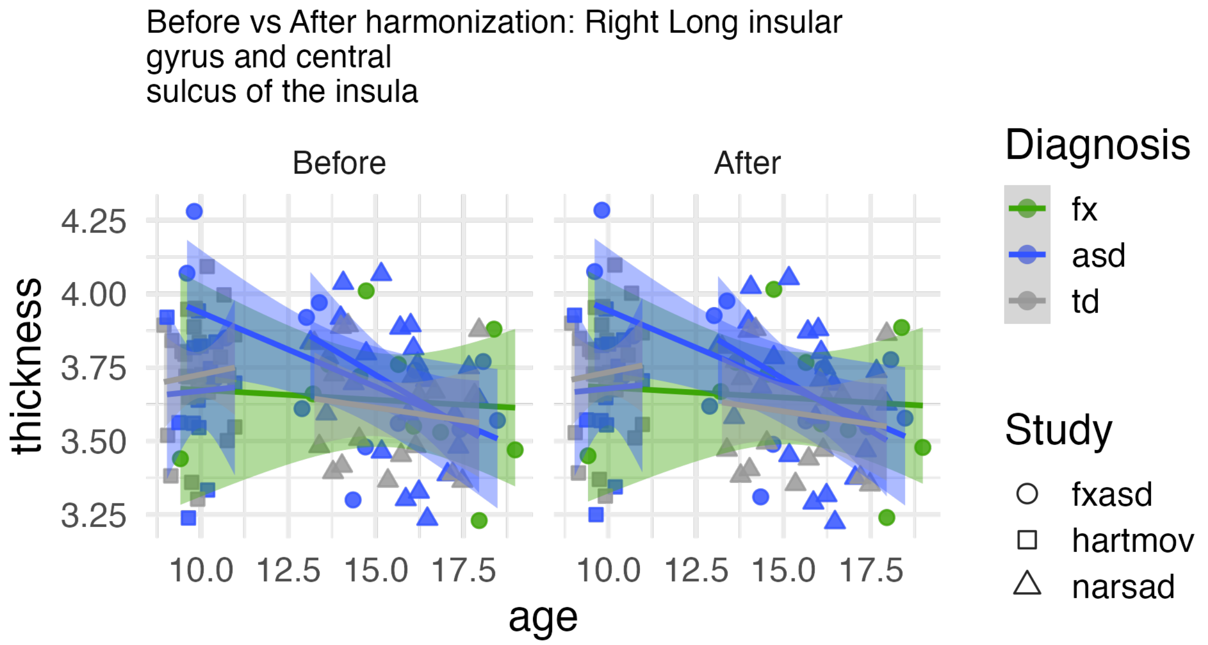
Before vs After harmonization: Right Long insular
gyrus and central
sulcus of the insula
Before
After
thickness
4.25
4.00
3.75
3.50
3.25
10.0
12.5
15.0
17.5
age
Diagnosis
fx
asd
td
Study
fxasd
hartmov
narsad

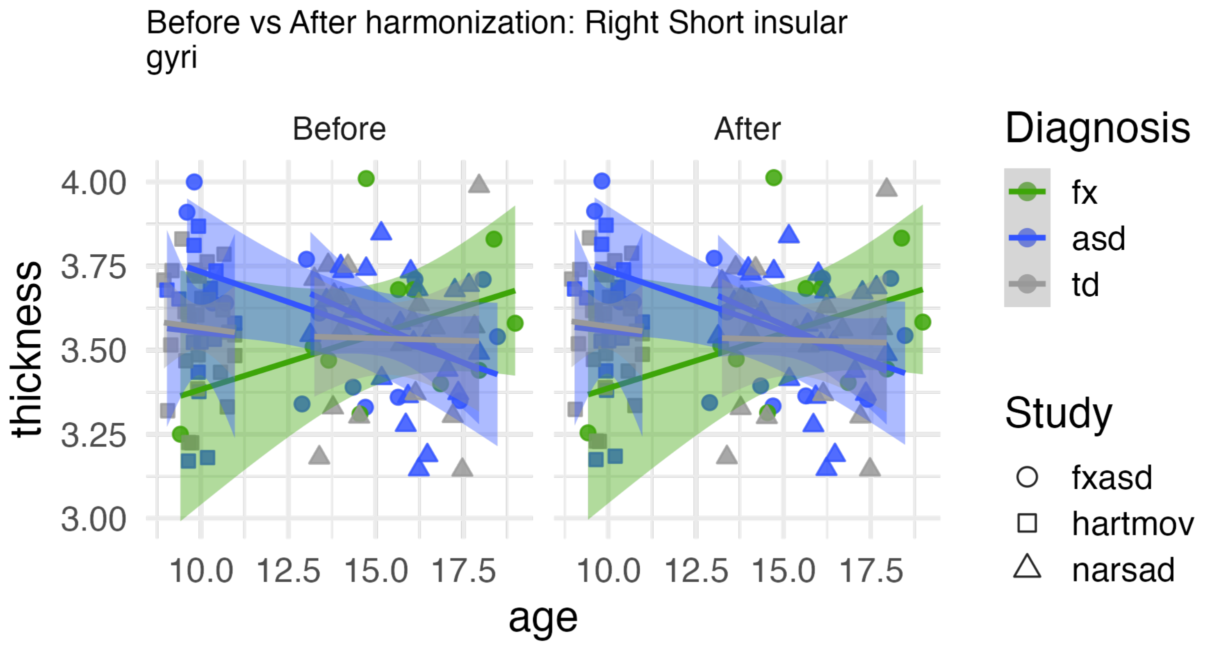
Before vs After harmonization: Right Short insular
gyri
Before
After
thickness
4.00
3.75
3.50
3.25
3.00
10.0
12.5
15.0
17.5
age
Diagnosis
fx
asd
td
Study
fxasd
hartmov
narsad

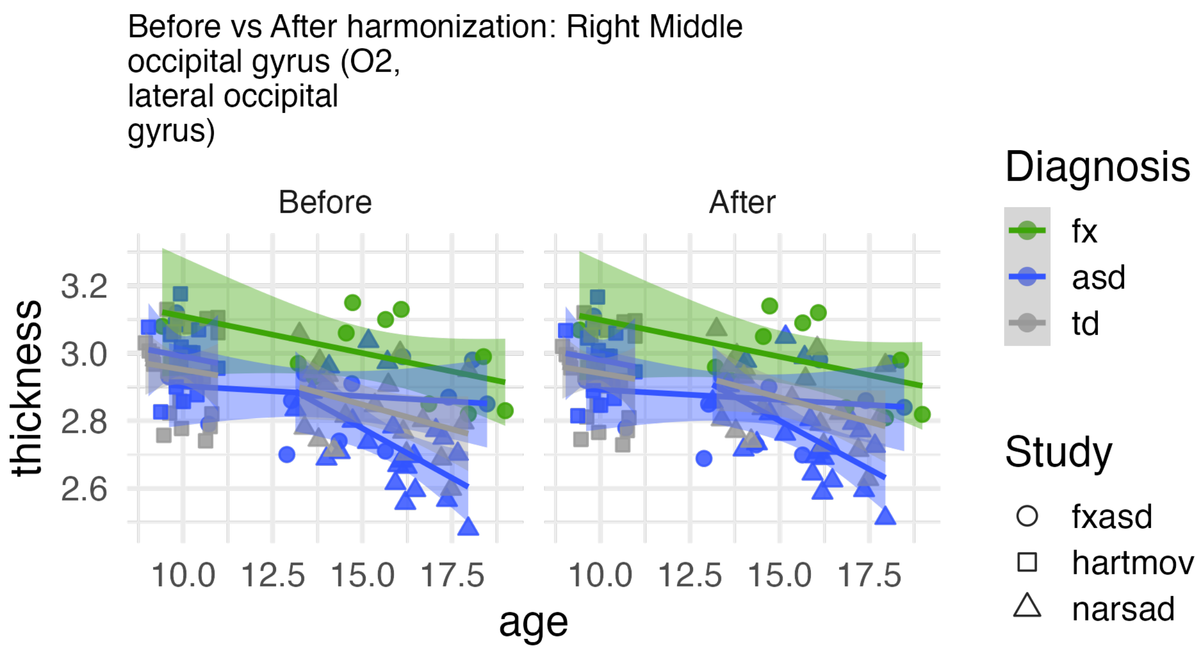
Before vs After harmonization: Right Middle
occipital gyrus (O2,
lateral occipital
gyrus)
Before
After
thickness
3.2
3.0
2.8
2.6
10.0
12.5
15.0
17.5
age
Diagnosis
fx
asd
td
Study
fxasd
hartmov
narsad

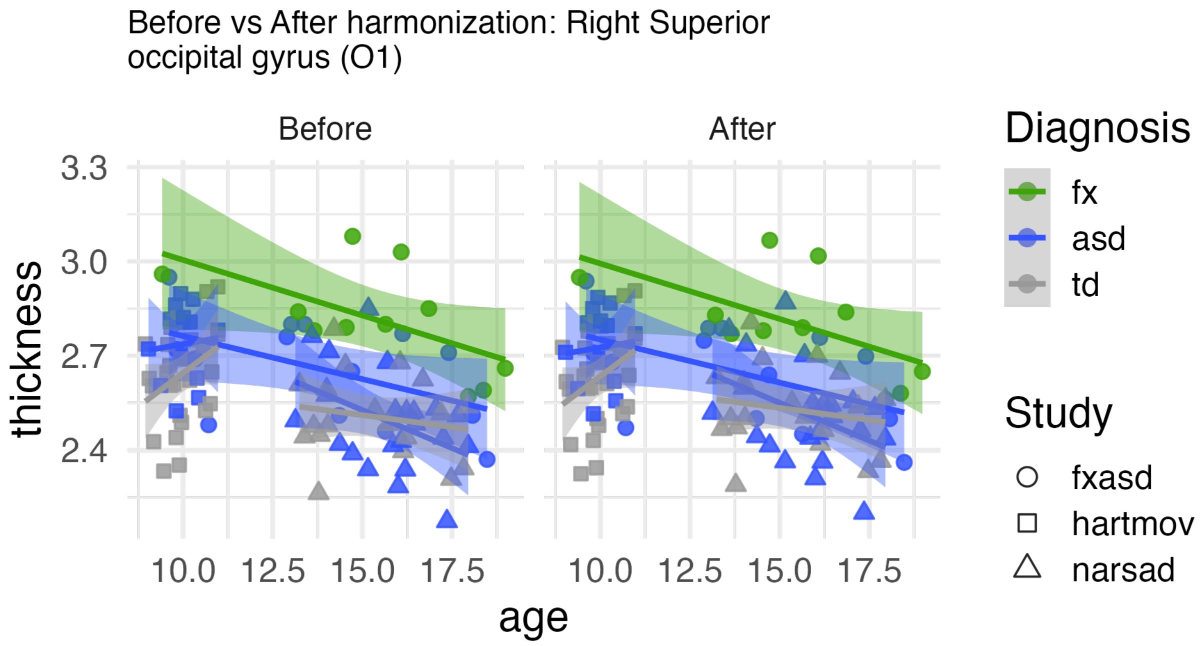
Before vs After harmonization: Right Superior
occipital gyrus (O1)
Before
After
thickness
3.3
3.0
2.7
2.4
10.0
12.5
15.0
17.5
age
Diagnosis
fx
asd
td
Study
fxasd
hartmov
narsad

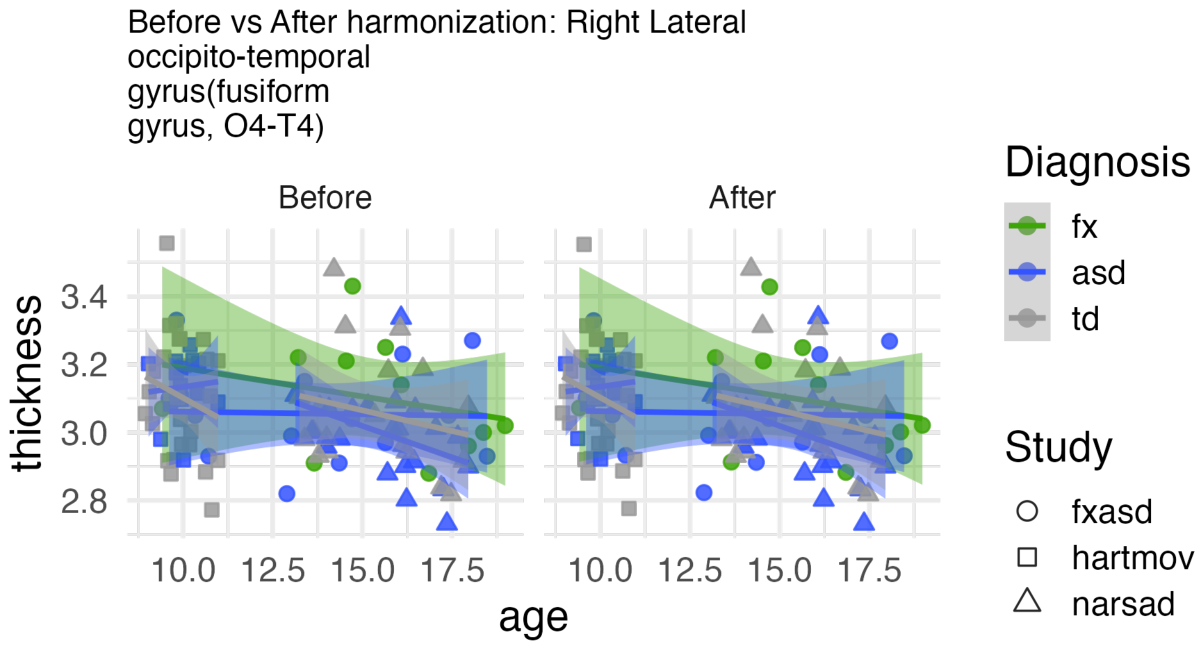
Before vs After harmonization: Right Lateral
occipito-temporal
gyrus(fusiform
gyrus, O4-T4)
Before
After
thickness
3.4
3.2
3.0
2.8
10.0
12.5
15.0
17.5
age
Diagnosis
fx
asd
td
Study
fxasd
hartmov
narsad

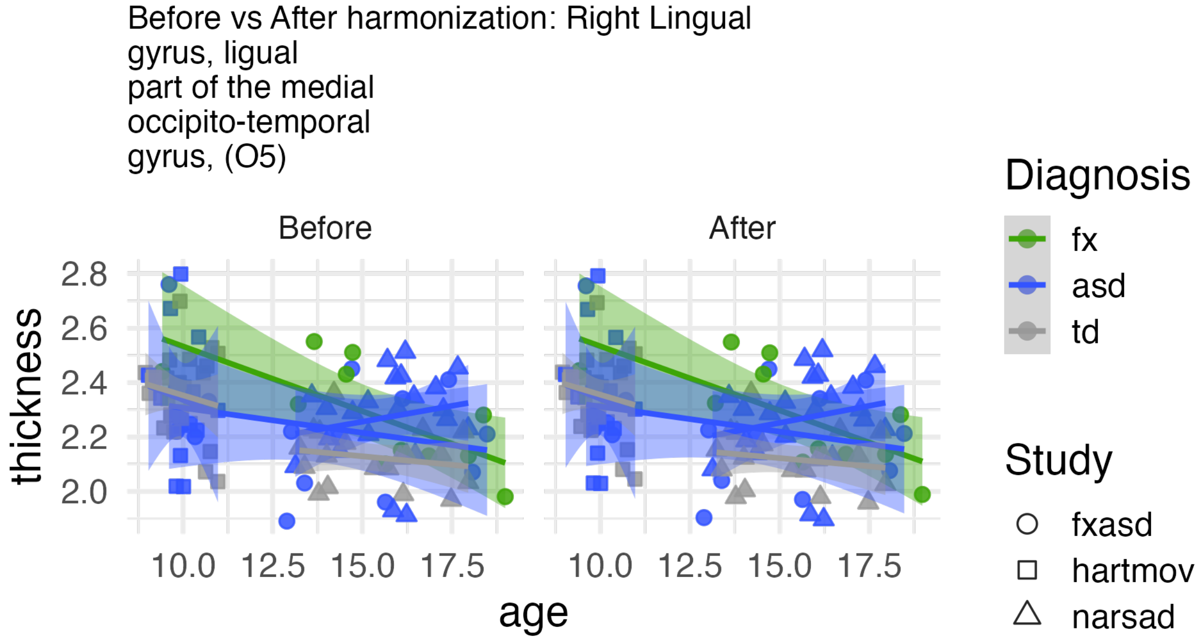
Before vs After harmonization: Right Lingual
gyrus, ligual
part of the medial
occipito-temporal
gyrus, (O5)
Before
After
thickness
2.8
2.6
2.4
2.2
2.0
10.0
12.5
15.0
17.5
age
Diagnosis
fx
asd
td
Study
fxasd
hartmov
narsad

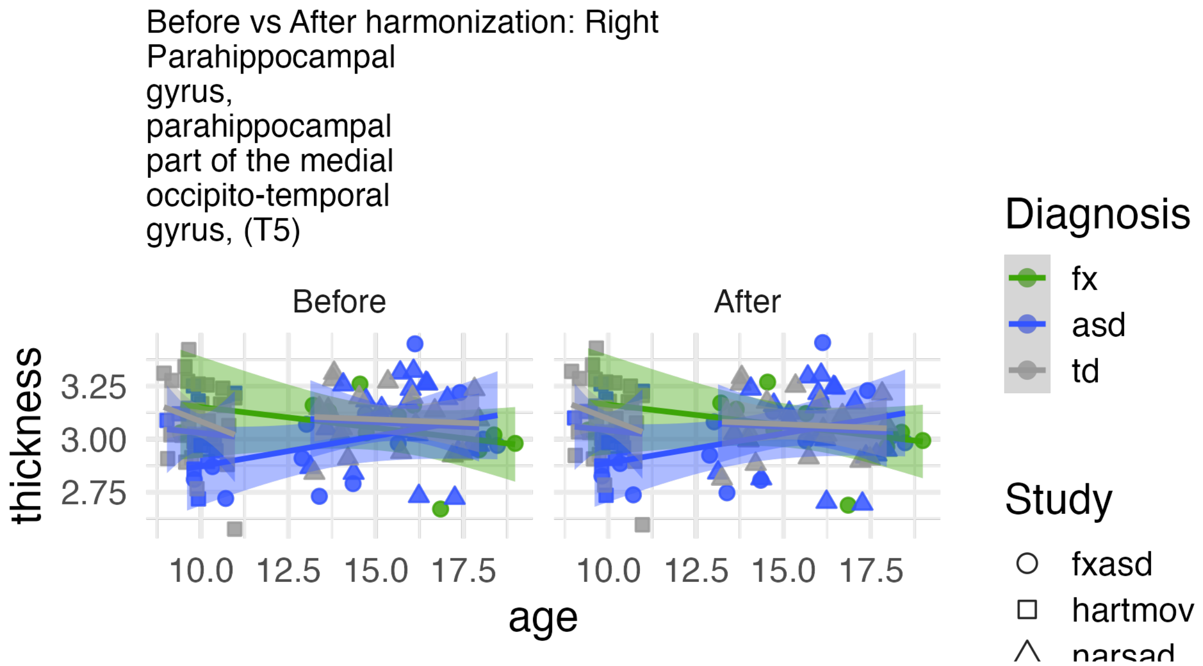
Before vs After harmonization: Right Parahippocampal gyrus, parahippocampal part of the medial occipito-temporal gyrus, (T5)
Before
After
thickness
3.25
3.00
2.75
10.0
12.5
15.0
17.5
age
Diagnosis
fx
asd
td
Study
fxasd
hartmov
narsad

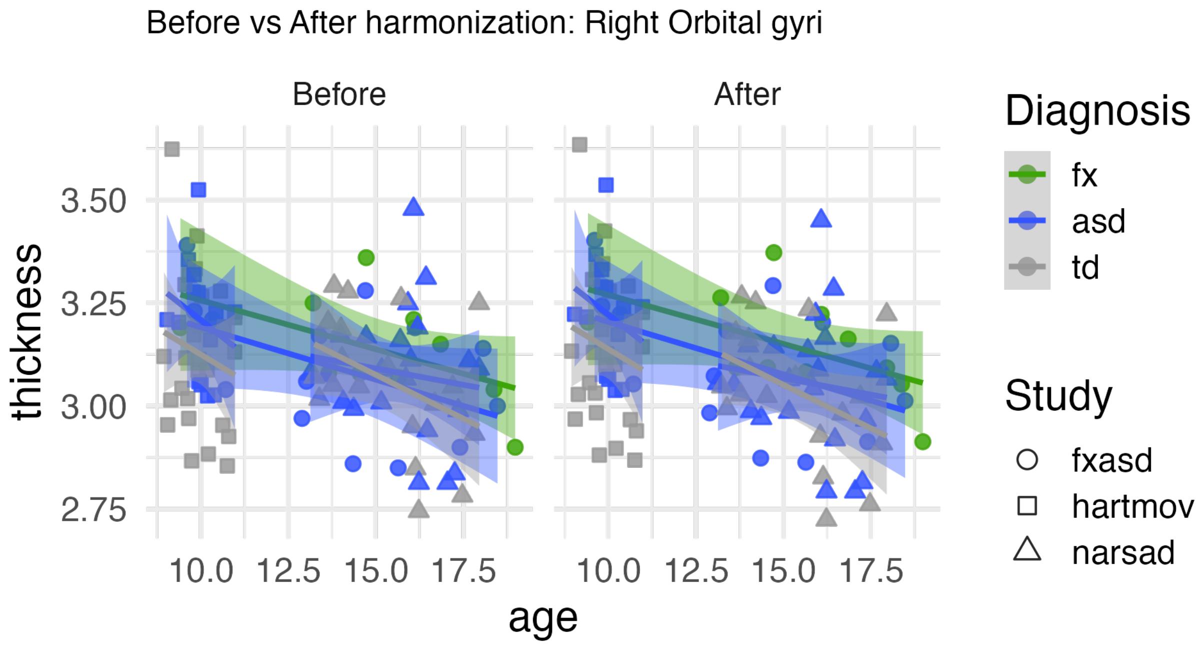
Before vs After harmonization: Right Orbital gyri
Before
After
thickness
3.50
3.25
3.00
2.75
10.0
12.5
15.0
17.5
age
Diagnosis
fx
asd
td
Study
fxasd
hartmov
narsad

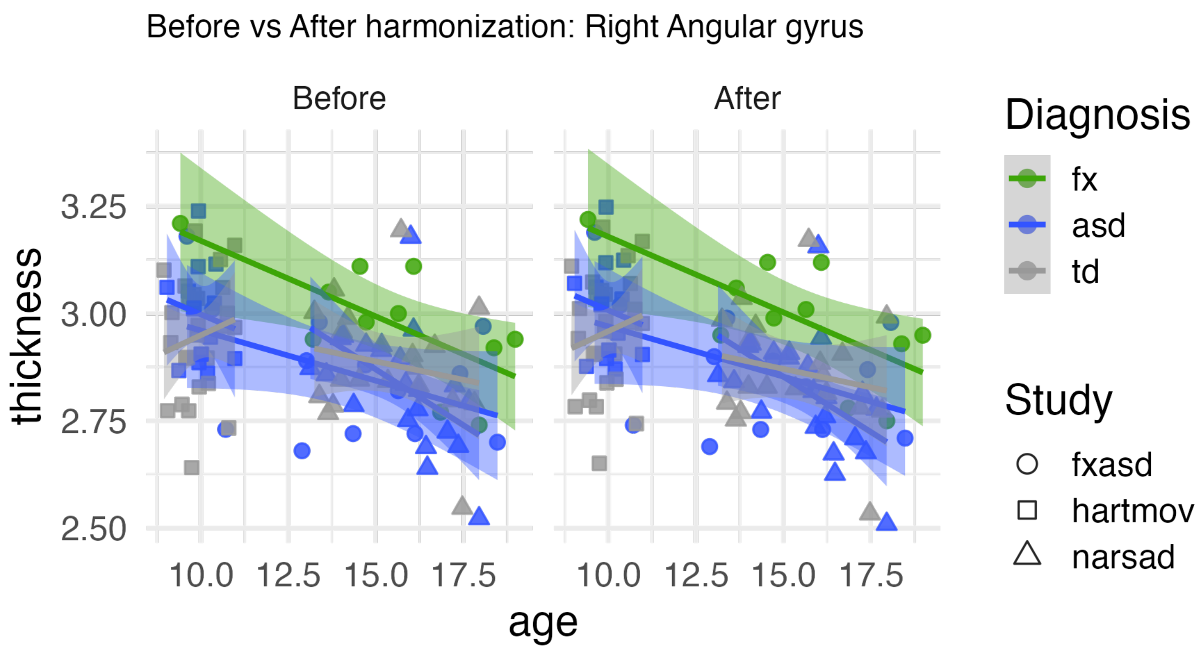
Before vs After harmonization: Right Angular gyrus
Before
After
thickness
3.25
3.00
2.75
2.50
10.0
12.5
15.0
17.5
age
Diagnosis
fx
asd
td
Study
fxasd
hartmov
narsad

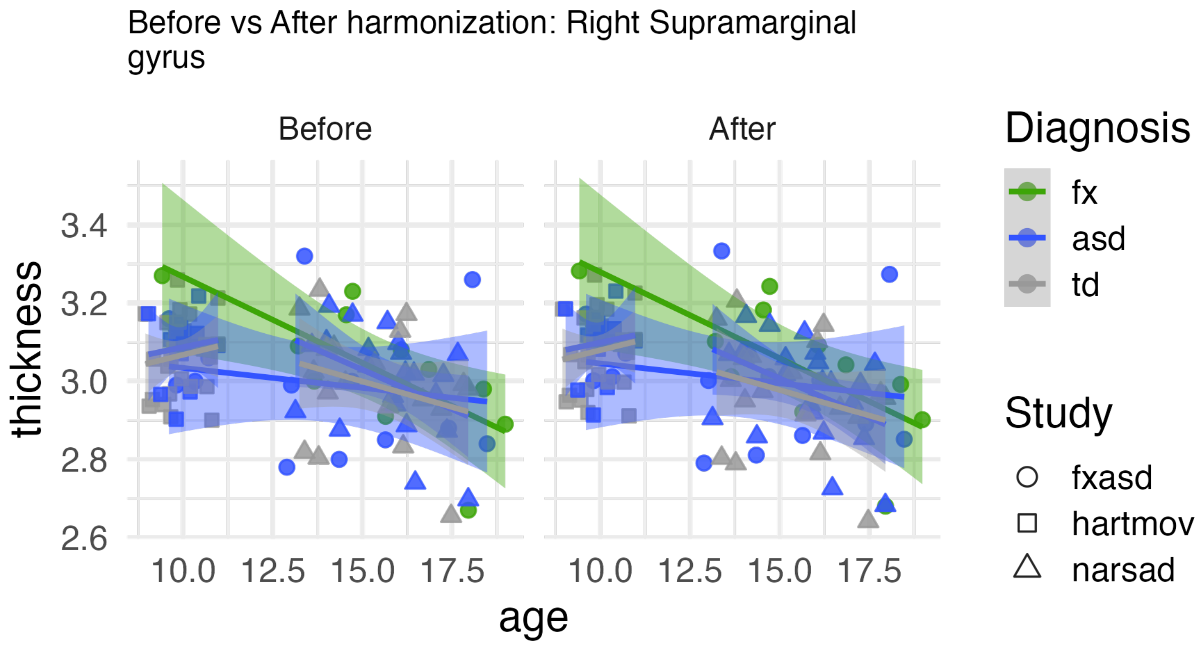
Before vs After harmonization: Right Supramarginal gyrus
Before
After
thickness
3.4
3.2
3.0
2.8
2.6
10.0
12.5
15.0
17.5
age
Diagnosis
fx
asd
td
Study
fxasd
hartmov
narsad

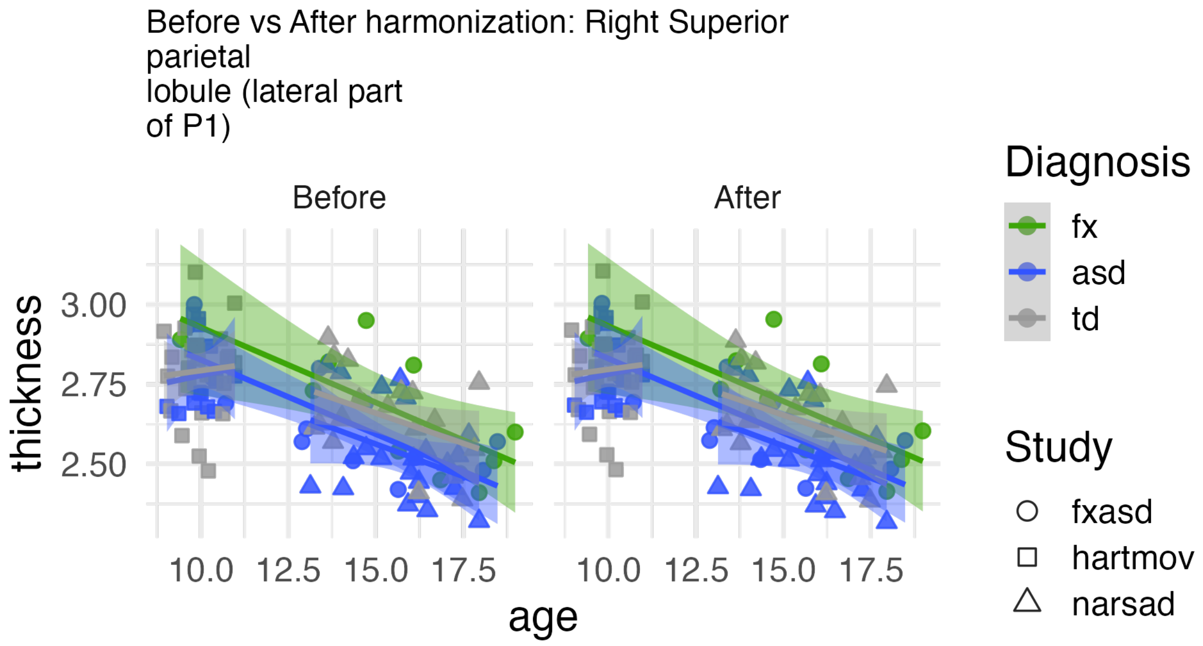
Before vs After harmonization: Right Superior parietal lobule (lateral part of P1)
Before
After
thickness
3.00
2.75
2.50
10.0
12.5
15.0
17.5
age
Diagnosis
fx
asd
td
Study
fxasd
hartmov
narsad

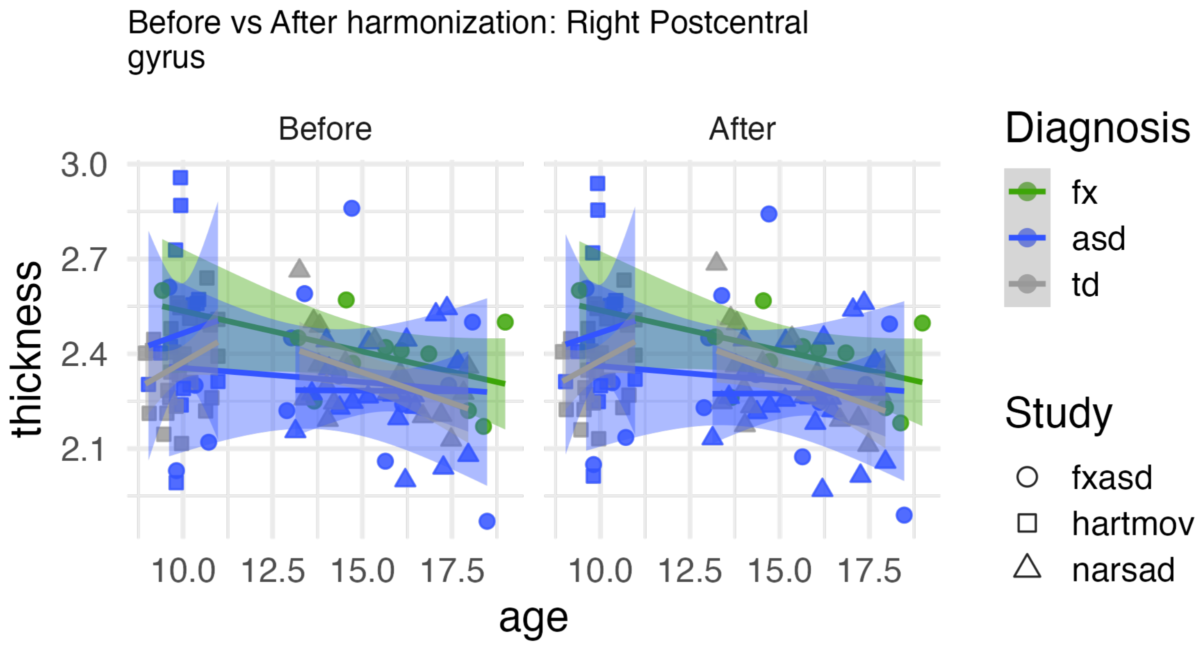
Before vs After harmonization: Right Postcentral gyrus
Before
After
thickness
3.0
2.7
2.4
2.1
10.0
12.5
15.0
17.5
age
Diagnosis
fx
asd
td
Study
fxasd
hartmov
narsad

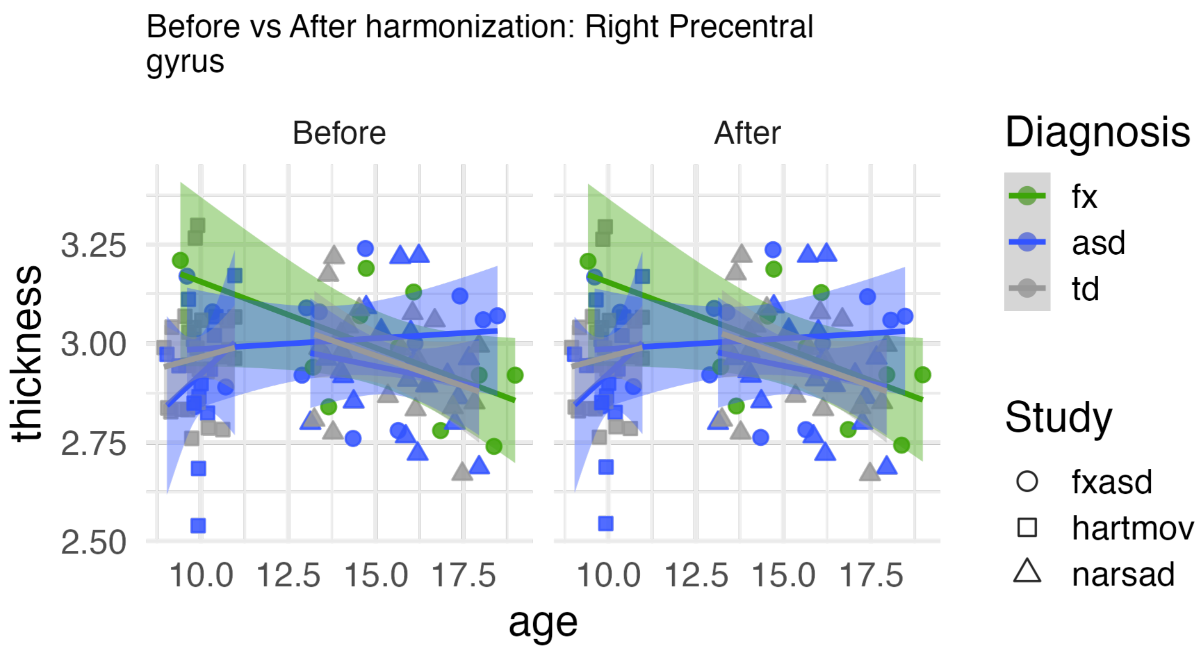
Before vs After harmonization: Right Precentral gyrus
Before
After
thickness
3.25
3.00
2.75
2.50
10.0
12.5
15.0
17.5
age
Diagnosis
fx
asd
td
Study
fxasd
hartmov
narsad

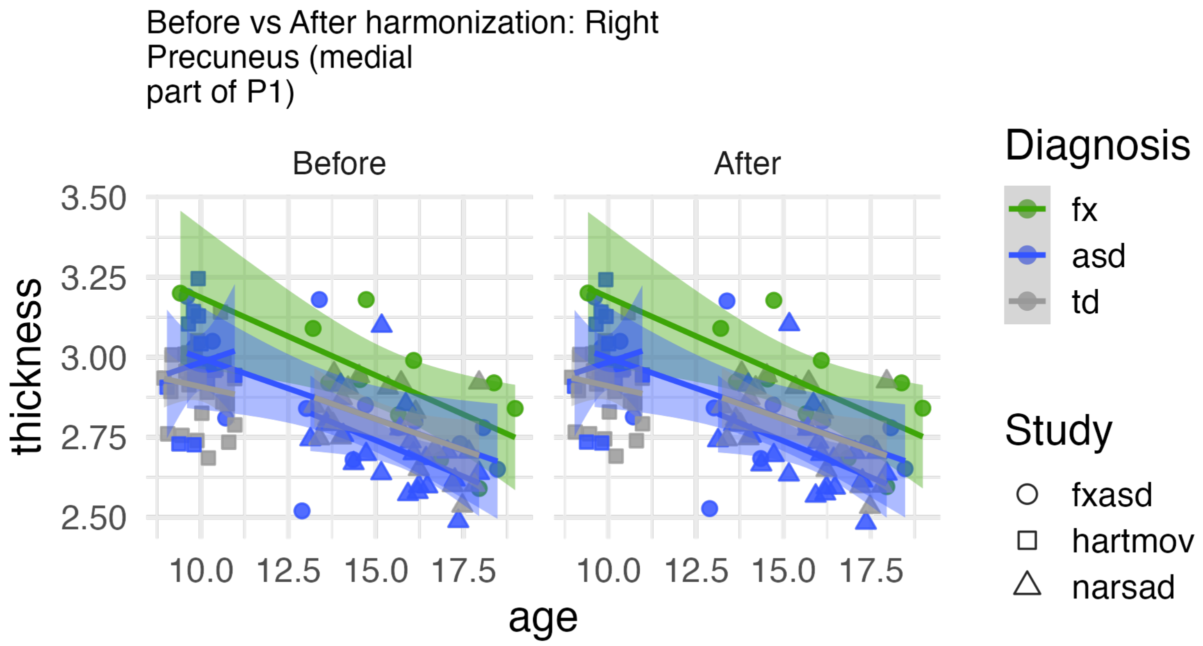
Before vs After harmonization: Right Precuneus (medial part of P1)
Before
After
thickness
3.50
3.25
3.00
2.75
2.50
10.0
12.5
15.0
17.5
age
Diagnosis
fx
asd
td
Study
fxasd
hartmov
narsad

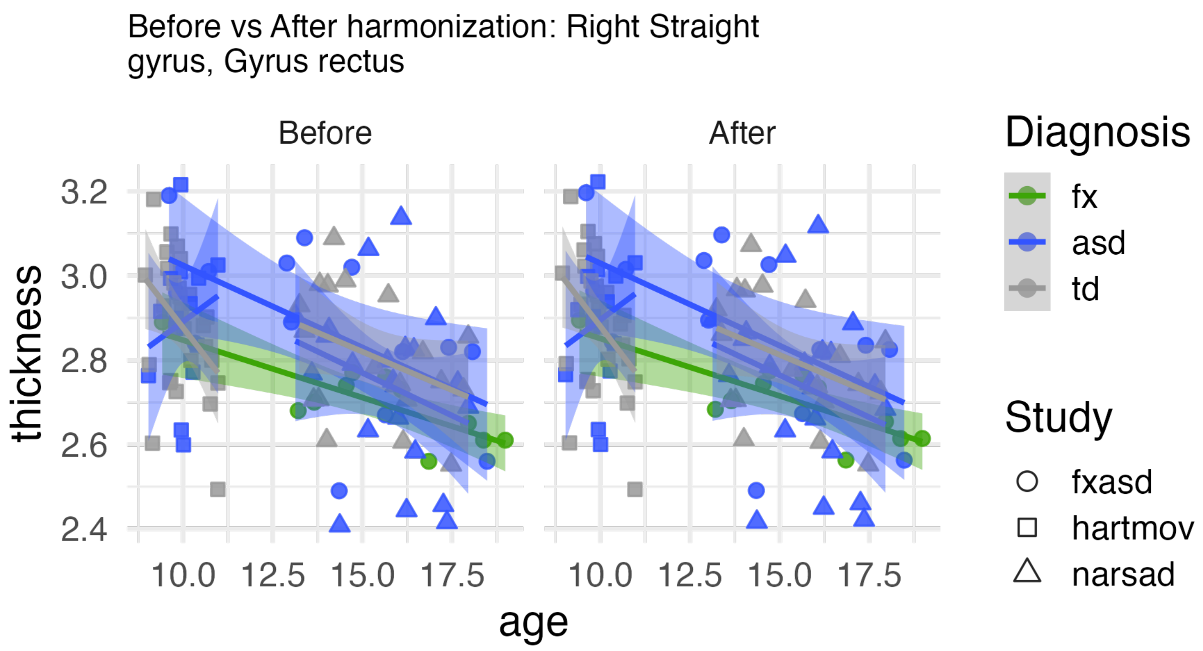
Before vs After harmonization: Right Straight
gyrus, Gyrus rectus
Before
After
thickness
3.2
3.0
2.8
2.6
2.4
10.0 12.5 15.0 17.5
10.0 12.5 15.0 17.5
age
Diagnosis
fx
asd
td
Study
fxasd
hartmov
narsad

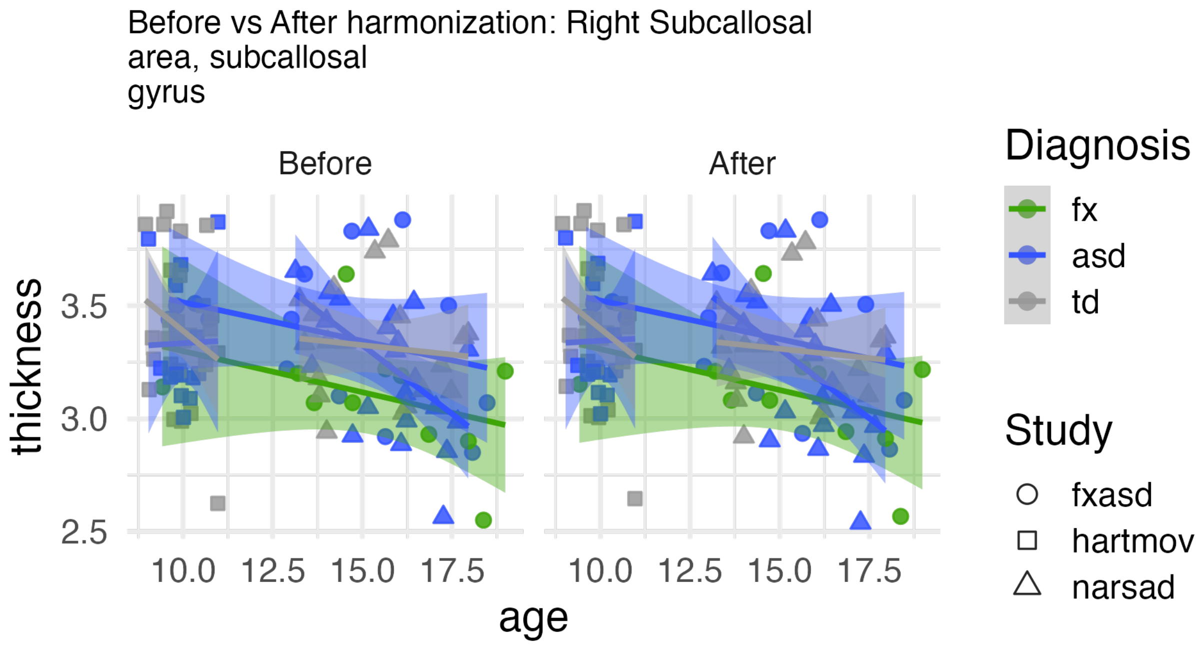
Before vs After harmonization: Right Subcallosal
area, subcallosal
gyrus
Before
After
thickness
3.5
3.0
2.5
10.0 12.5 15.0 17.5
10.0 12.5 15.0 17.5
age
Diagnosis
fx
asd
td
Study
fxasd
hartmov
narsad

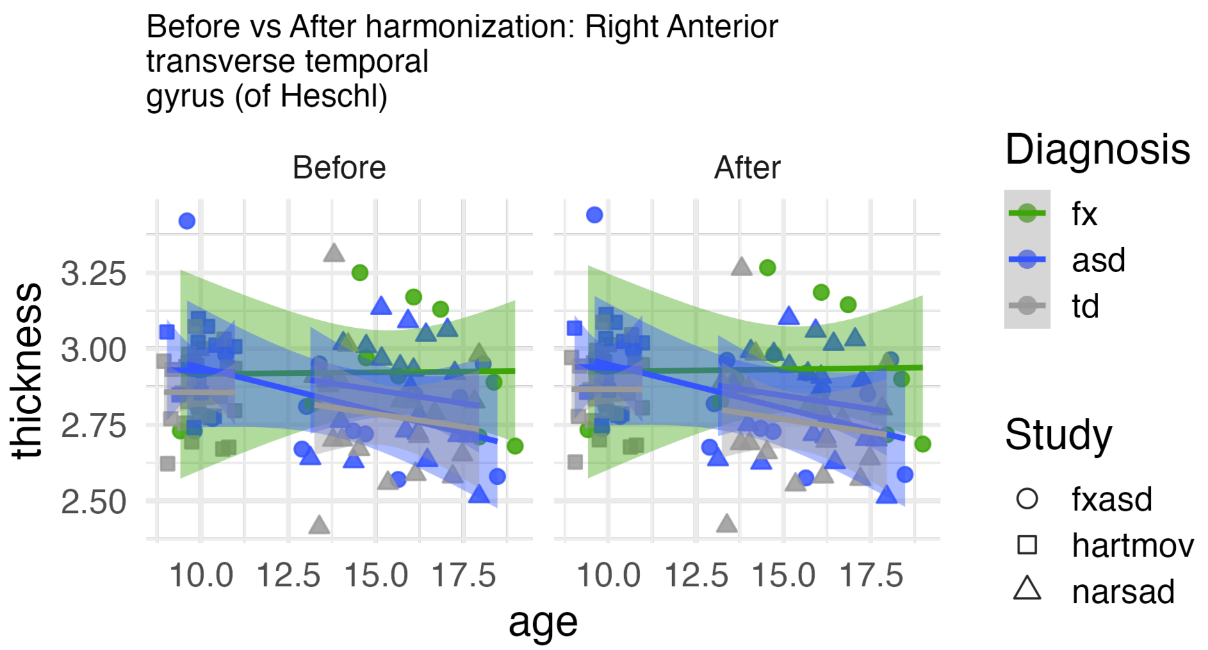
Before vs After harmonization: Right Anterior
transverse temporal
gyrus (of Heschl)
Before
After
thickness
3.25
3.00
2.75
2.50
10.0
12.5
15.0
17.5
age
Diagnosis
fx
asd
td
Study
fxasd
hartmov
narsad

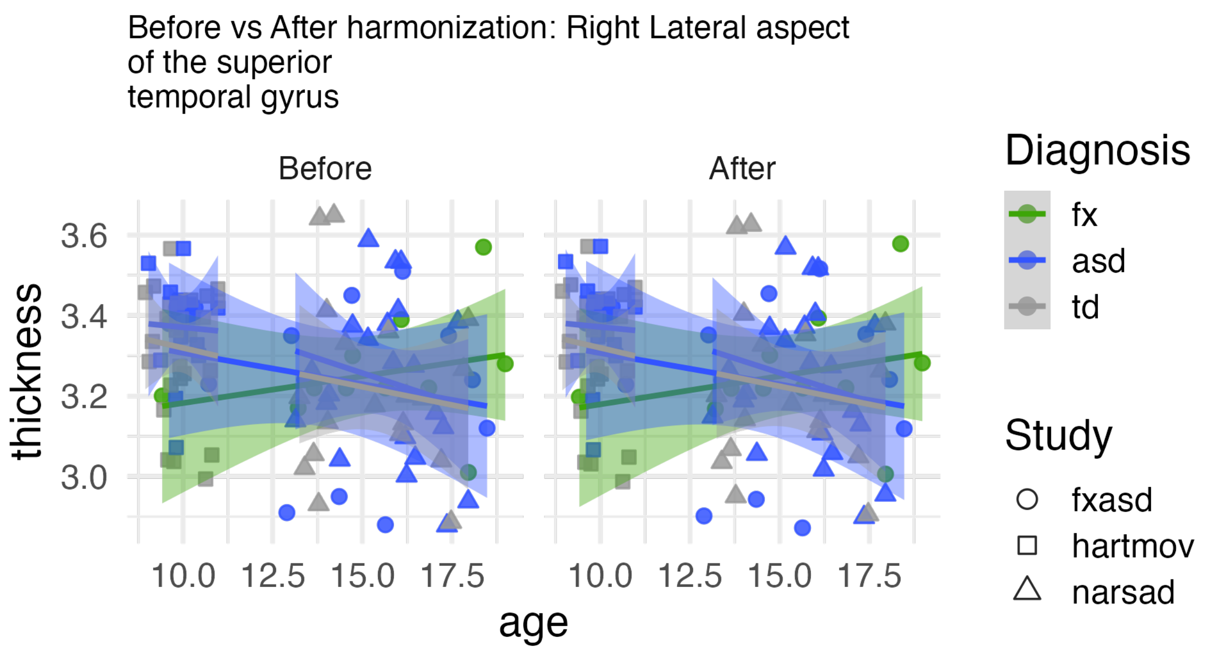
Before vs After harmonization: Right Lateral aspect
of the superior
temporal gyrus
Before
After
thickness
3.6
3.4
3.2
3.0
10.0
12.5
15.0
17.5
age
Diagnosis
fx
asd
td
Study
fxasd
hartmov
narsad

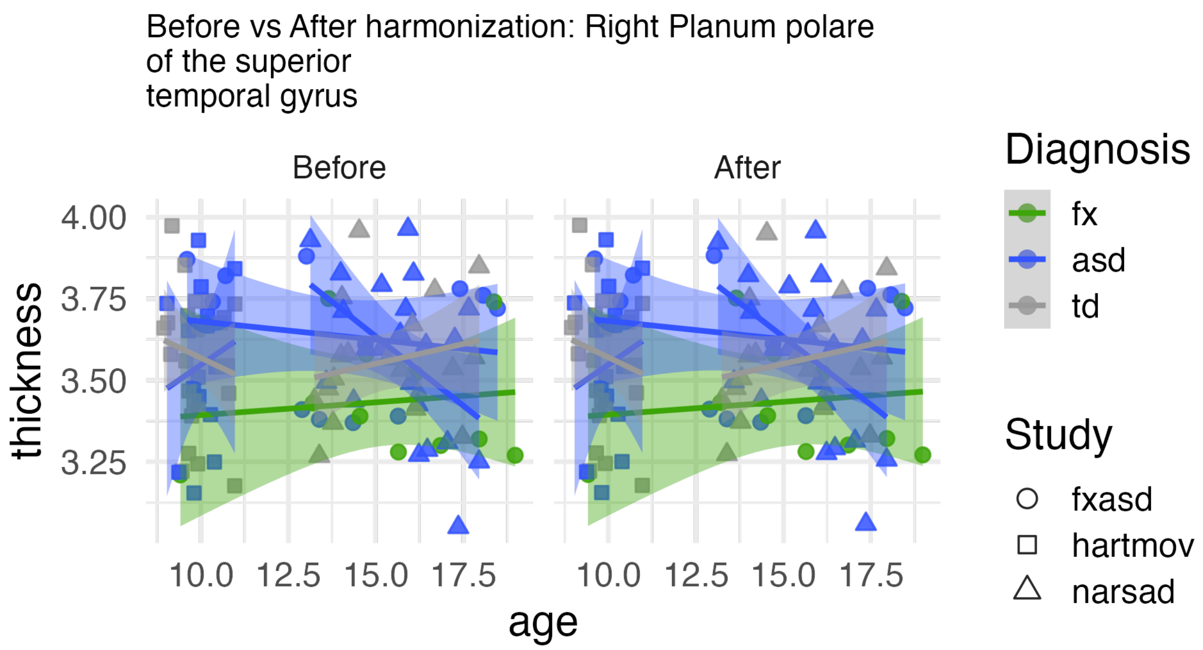
Before vs After harmonization: Right Planum polare
of the superior
temporal gyrus
Before
After
thickness
4.00
3.75
3.50
3.25
10.0
12.5
15.0
17.5
age
Diagnosis
fx
asd
td
Study
fxasd
hartmov
narsad

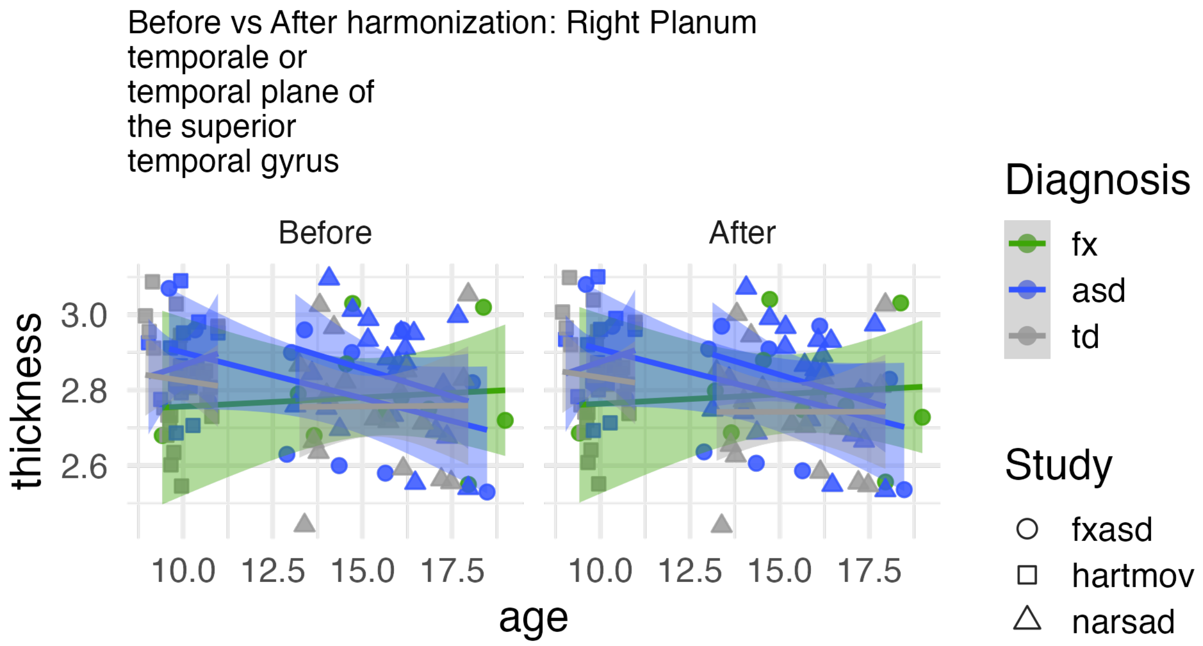
Before vs After harmonization: Right Planum
temporale or
temporal plane of
the superior
temporal gyrus
Before
After
thickness
3.0
2.8
2.6
10.0
12.5
15.0
17.5
age
Diagnosis
fx
asd
td
Study
fxasd
hartmov
narsad

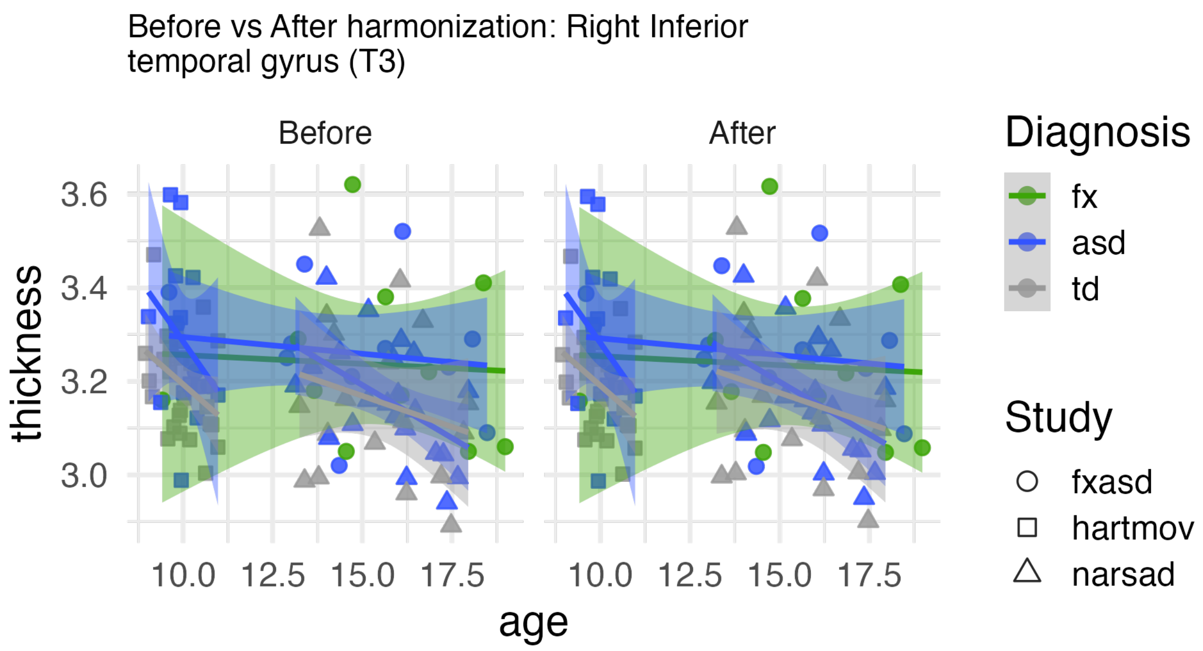
Before vs After harmonization: Right Inferior
temporal gyrus (T3)
Before
After
thickness
3.6
3.4
3.2
3.0
10.0
12.5
15.0
17.5
age
Diagnosis
fx
asd
td
Study
fxasd
hartmov
narsad

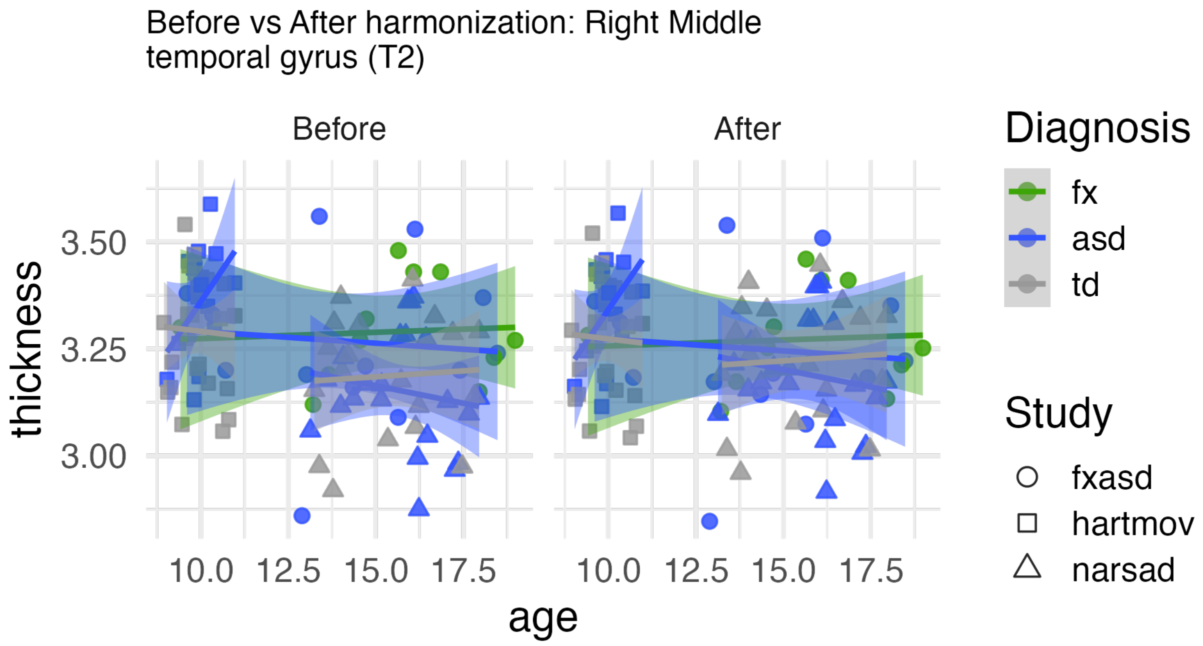
Before vs After harmonization: Right Middle
temporal gyrus (T2)
Before
After
thickness
3.50
3.25
3.00
10.0
12.5
15.0
17.5
age
Diagnosis
fx
asd
td
Study
fxasd
hartmov
narsad

Before vs After harmonization: Right Horizontal ramus of
the anterior segment
of the lateral
sulcus (or fissure)

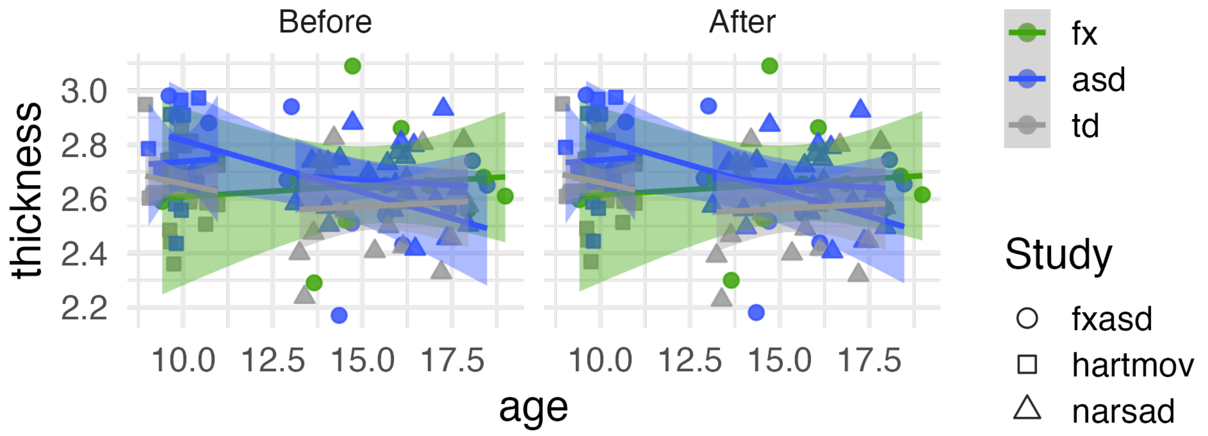

Before vs After harmonization: Right Vertical ramus
of the anterior
segment of the
lateral sulcus(or
fissure)

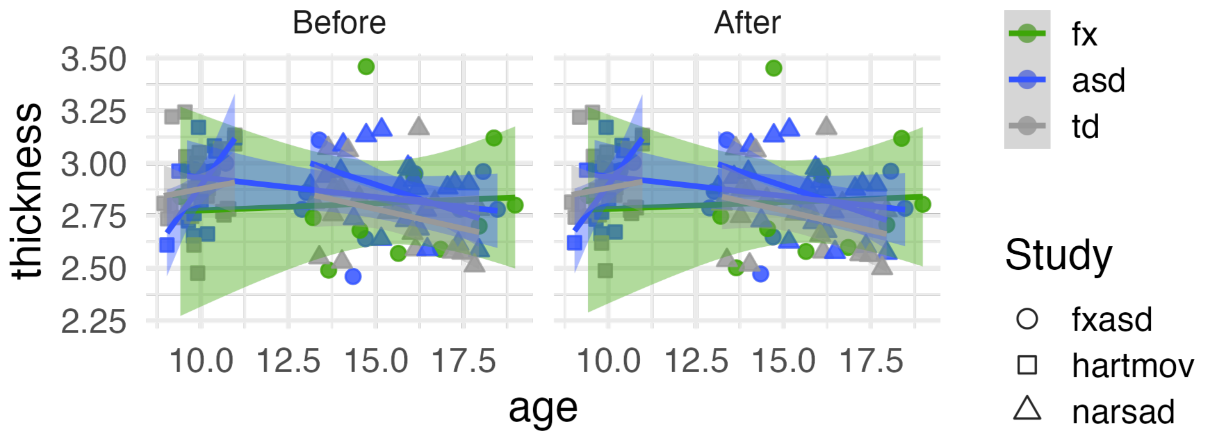

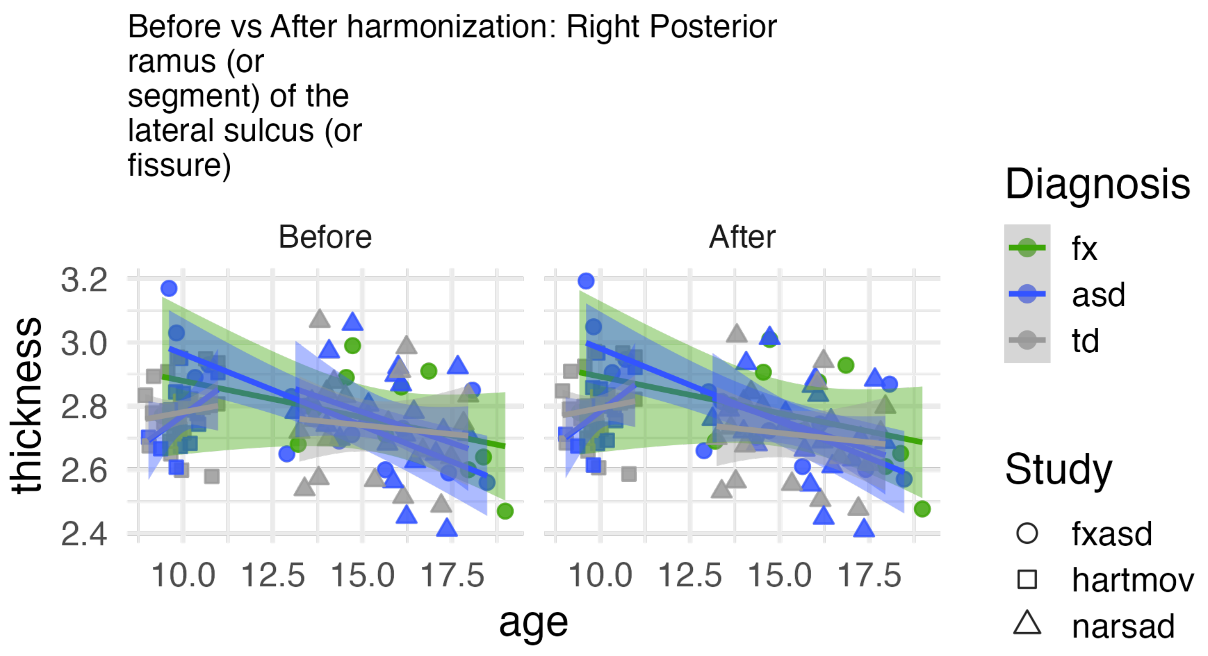
Before vs After harmonization: Right Posterior ramus (or segment) of the lateral sulcus (or fissure)
Before
After
thickness
3.2
3.0
2.8
2.6
2.4
10.0
12.5
15.0
17.5
age
Diagnosis
fx
asd
td
Study
fxasd
hartmov
narsad

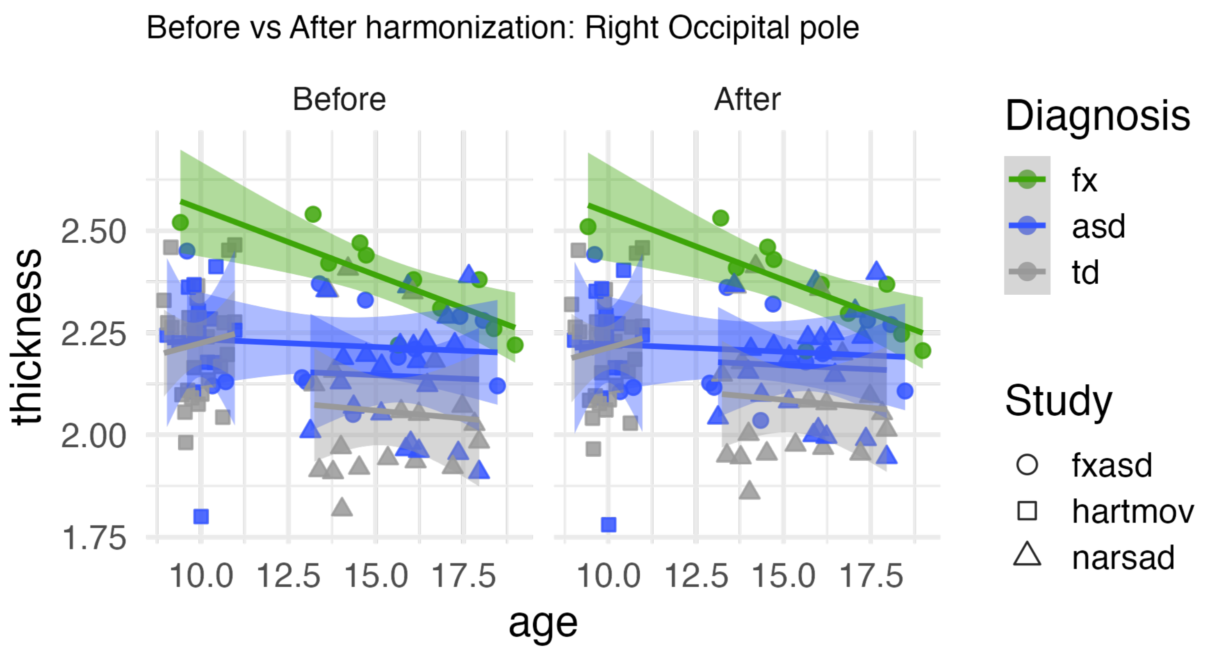
Before vs After harmonization: Right Occipital pole
Before
After
thickness
2.50
2.25
2.00
1.75
10.0
12.5
15.0
17.5
age
Diagnosis
fx
asd
td
Study
fxasd
hartmov
narsad

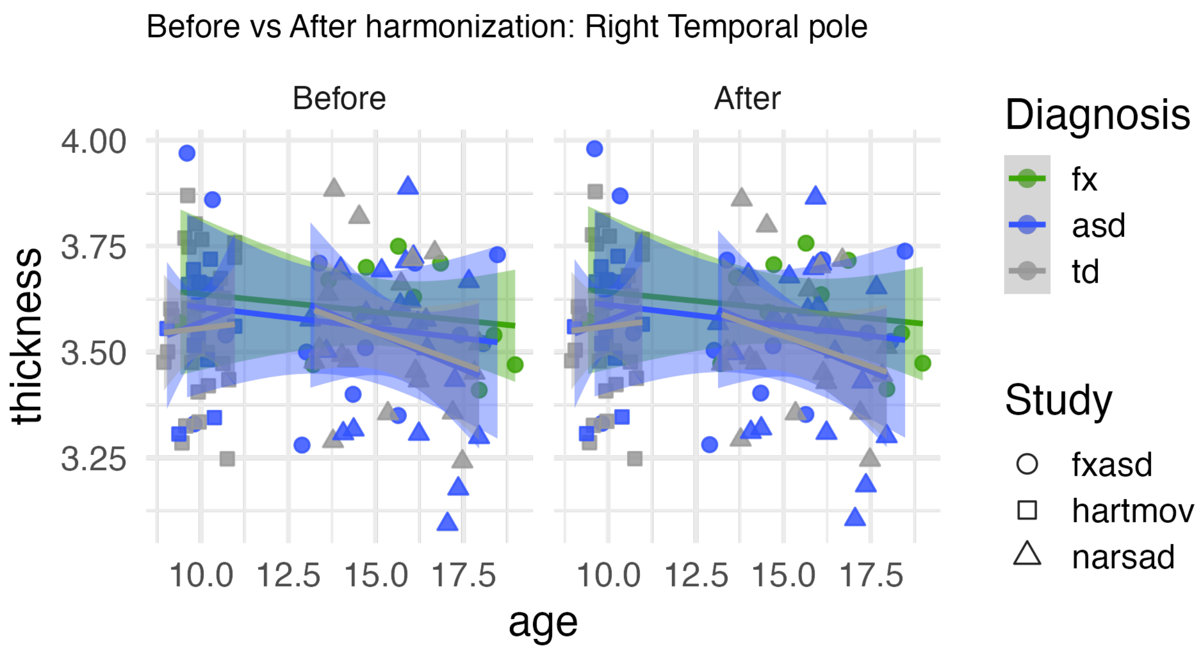
Before vs After harmonization: Right Temporal pole
Before
After
thickness
4.00
3.75
3.50
3.25
10.0
12.5
15.0
17.5
age
Diagnosis
fx
asd
td
Study
fxasd
hartmov
narsad

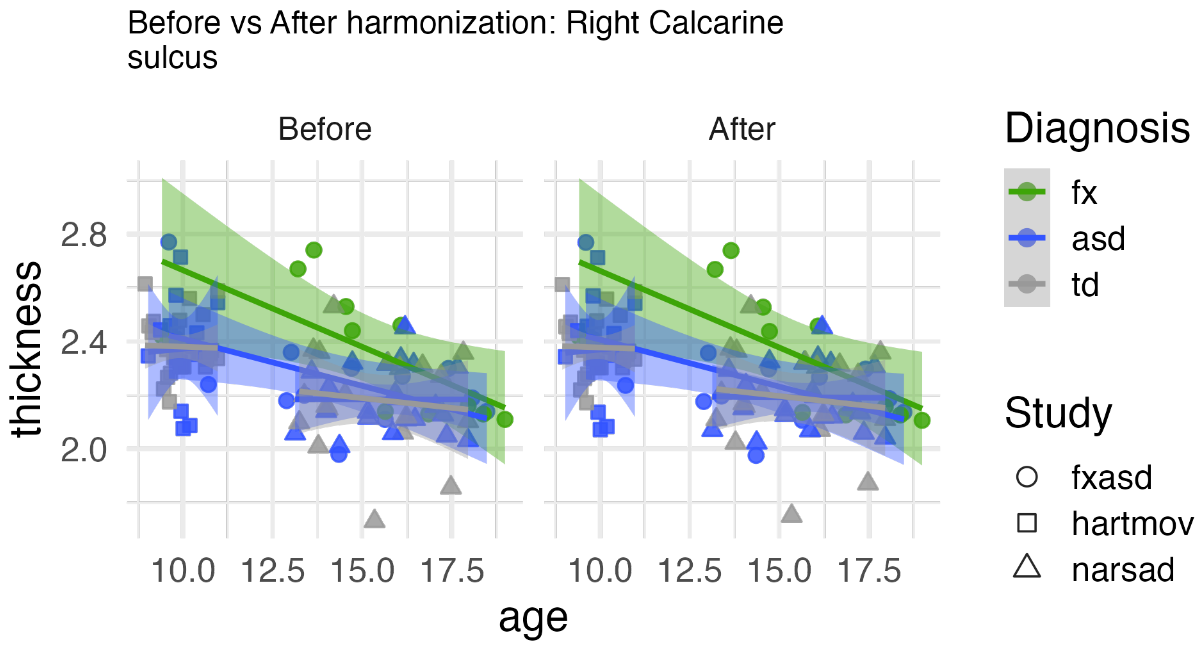
Before vs After harmonization: Right Calcarine sulcus
Before
After
thickness
2.8
2.4
2.0
10.0
12.5
15.0
17.5
age
Diagnosis
fx
asd
td
Study
fxasd
hartmov
narsad

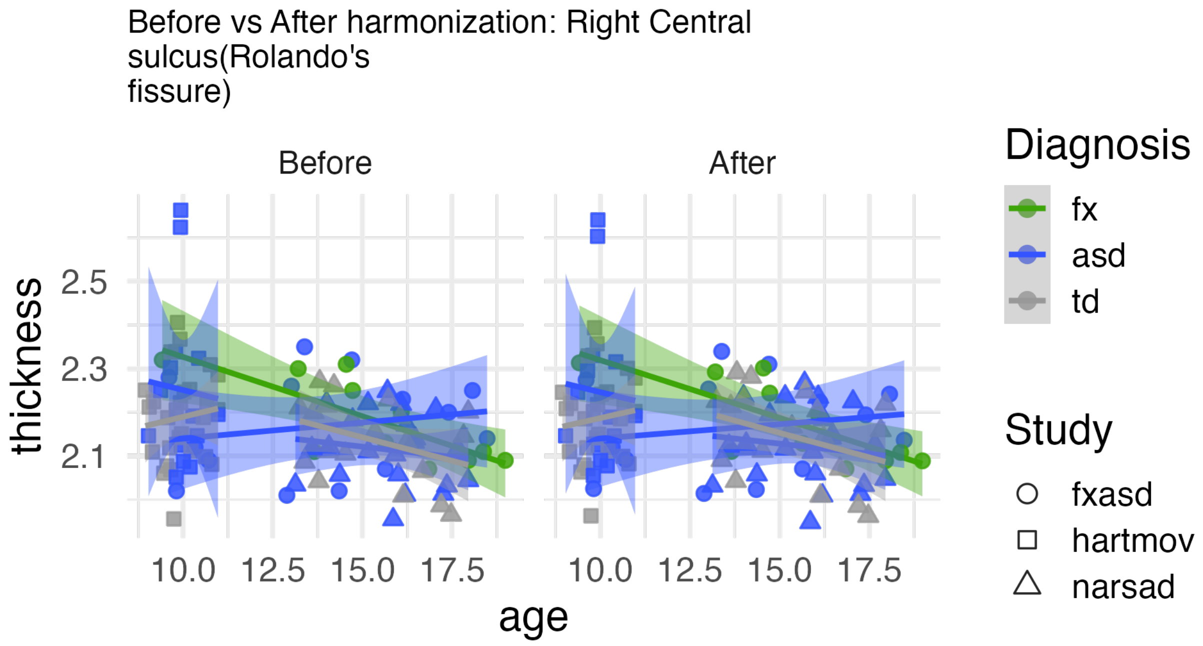

Before vs After harmonization: Right Central sulcus(Rolando's fissure)
Before
After
thickness
2.5
2.3
2.1
10.0
12.5
15.0
17.5
age
Diagnosis
fx
asd
td
Study
fxasd
hartmov
narsad

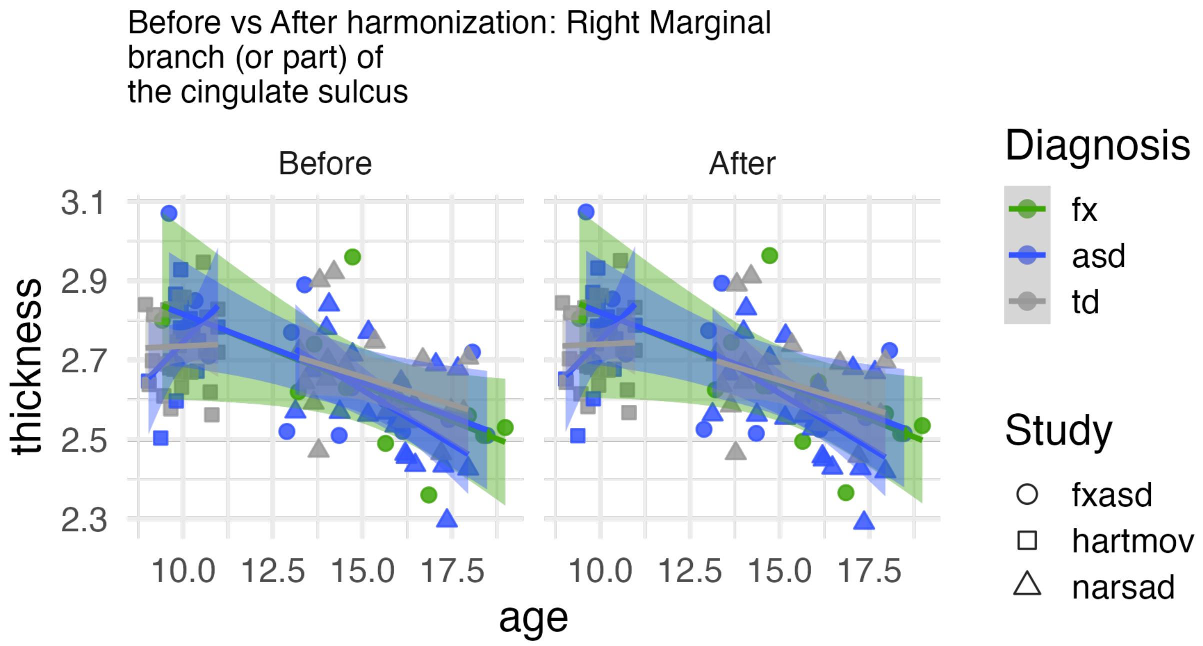

Before vs After harmonization: Right Marginal branch (or part) of the cingulate sulcus
Before
After
thickness
3.1
2.9
2.7
2.5
2.3
10.0
12.5
15.0
17.5
age
Diagnosis
fx
asd
td
Study
fxasd
hartmov
narsad

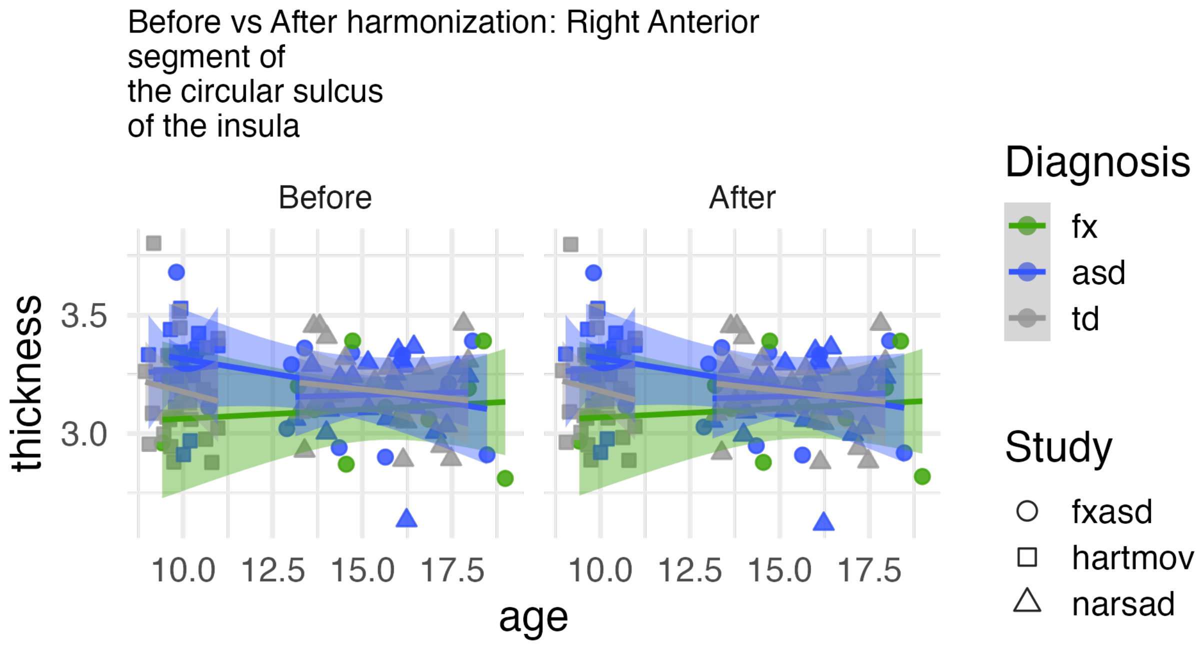

Before vs After harmonization: Right Anterior segment of the circular sulcus of the insula
Before
After
thickness
3.5
3.0
10.0
12.5
15.0
17.5
age
Diagnosis
fx
asd
td
Study
fxasd
hartmov
narsad

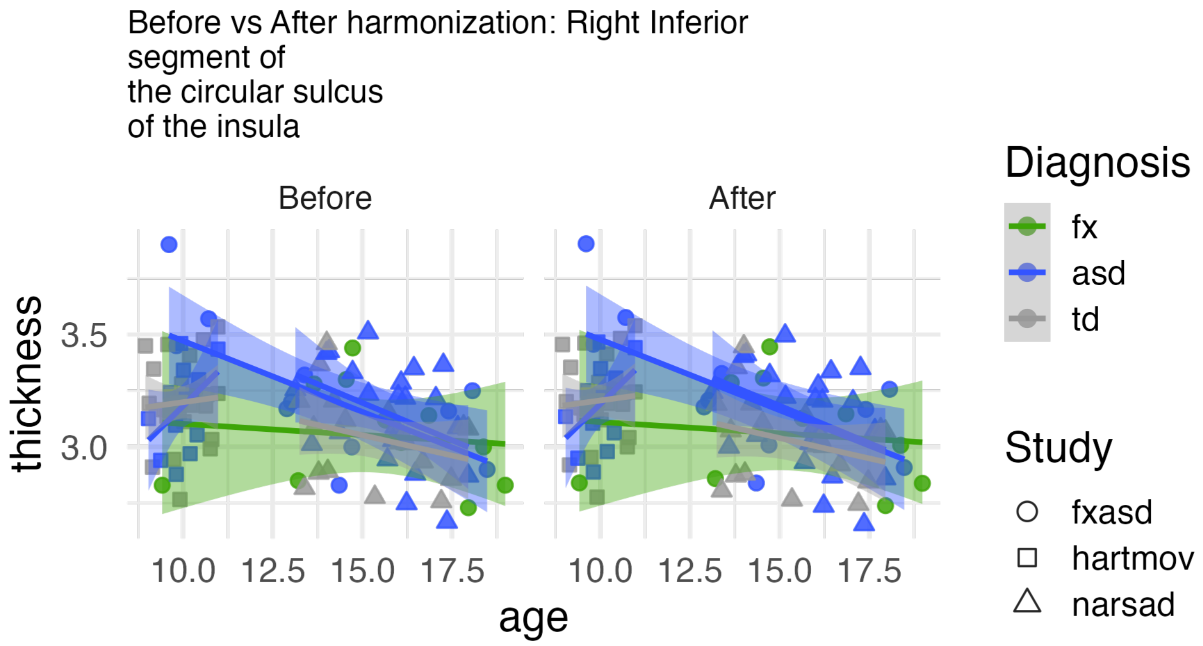

Before vs After harmonization: Right Inferior segment of the circular sulcus of the insula
Before
After
thickness
3.5
3.0
10.0
12.5
15.0
17.5
age
Diagnosis
fx
asd
td
Study
fxasd
hartmov
narsad

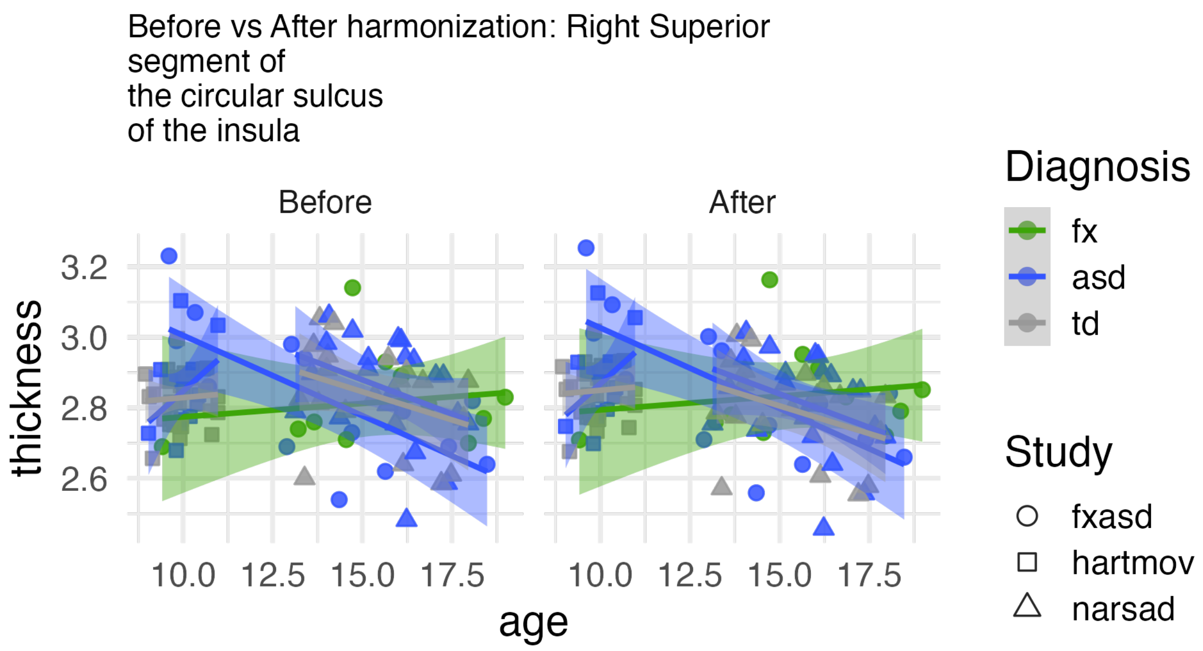
Before vs After harmonization: Right Superior
segment of
the circular sulcus
of the insula
Before
After
thickness
3.2
3.0
2.8
2.6
10.0
12.5
15.0
17.5
age
Diagnosis
fx
asd
td
Study
fxasd
hartmov
narsad

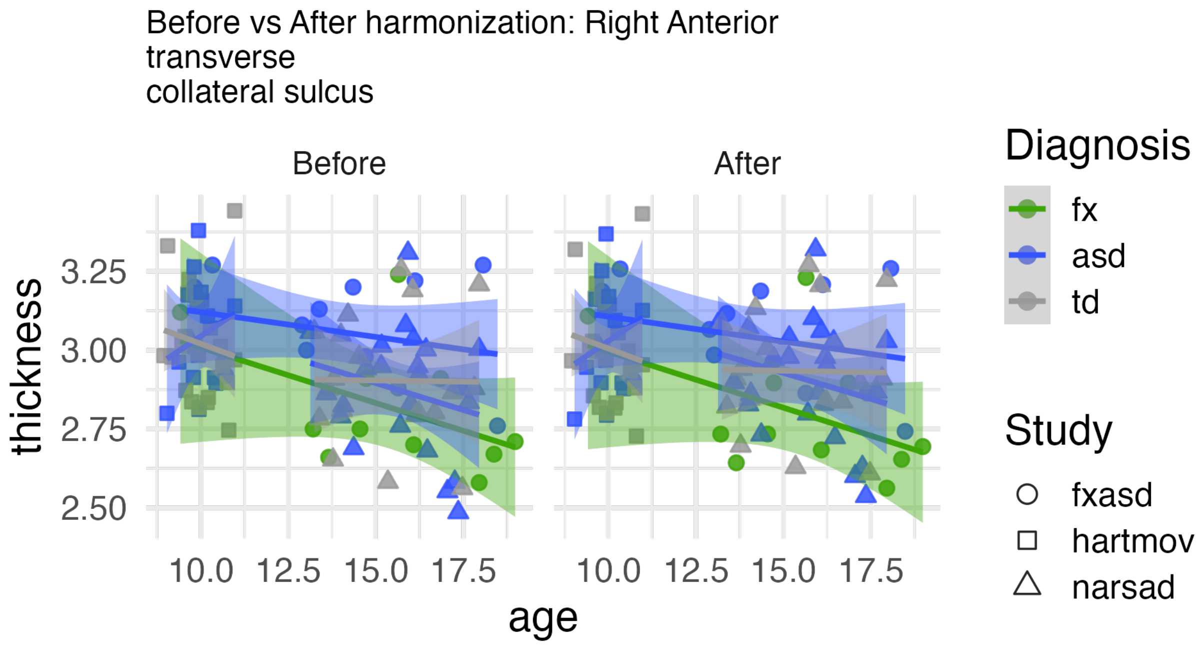
Before vs After harmonization: Right Anterior
transverse
collateral sulcus
Before
After
thickness
3.25
3.00
2.75
2.50
10.0
12.5
15.0
17.5
age
Diagnosis
fx
asd
td
Study
fxasd
hartmov
narsad

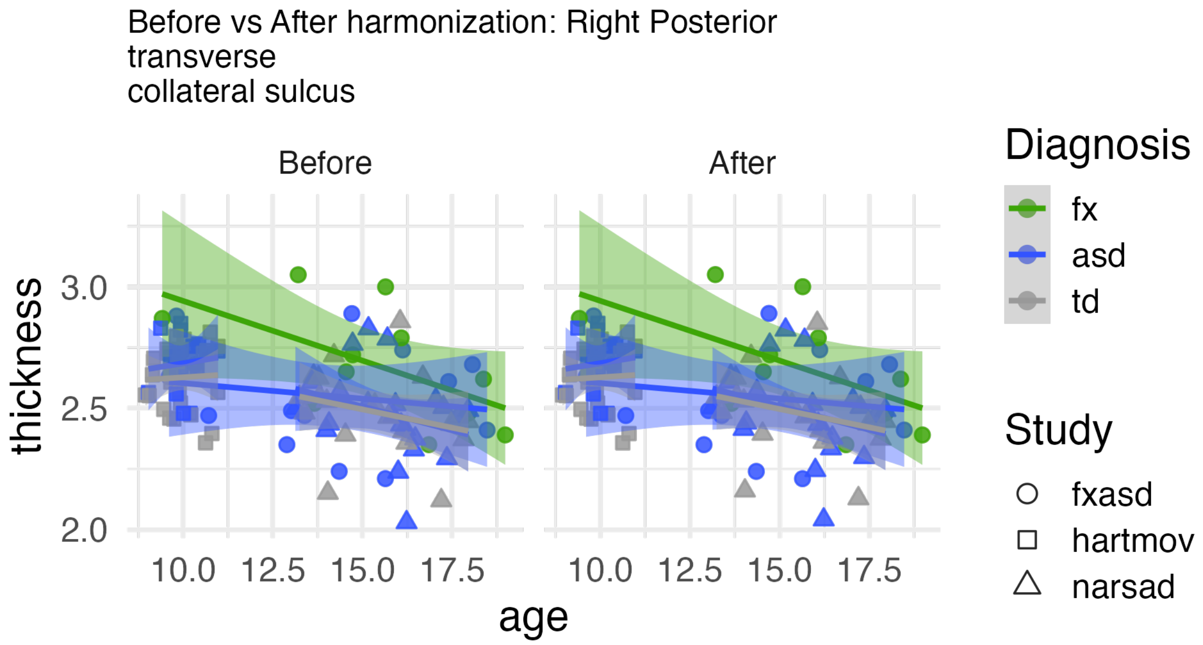
Before vs After harmonization: Right Posterior transverse
collateral sulcus
Before
After
thickness
3.0
2.5
2.0
10.0
12.5
15.0
17.5
age
Diagnosis
fx
asd
td
Study
fxasd
hartmov
narsad

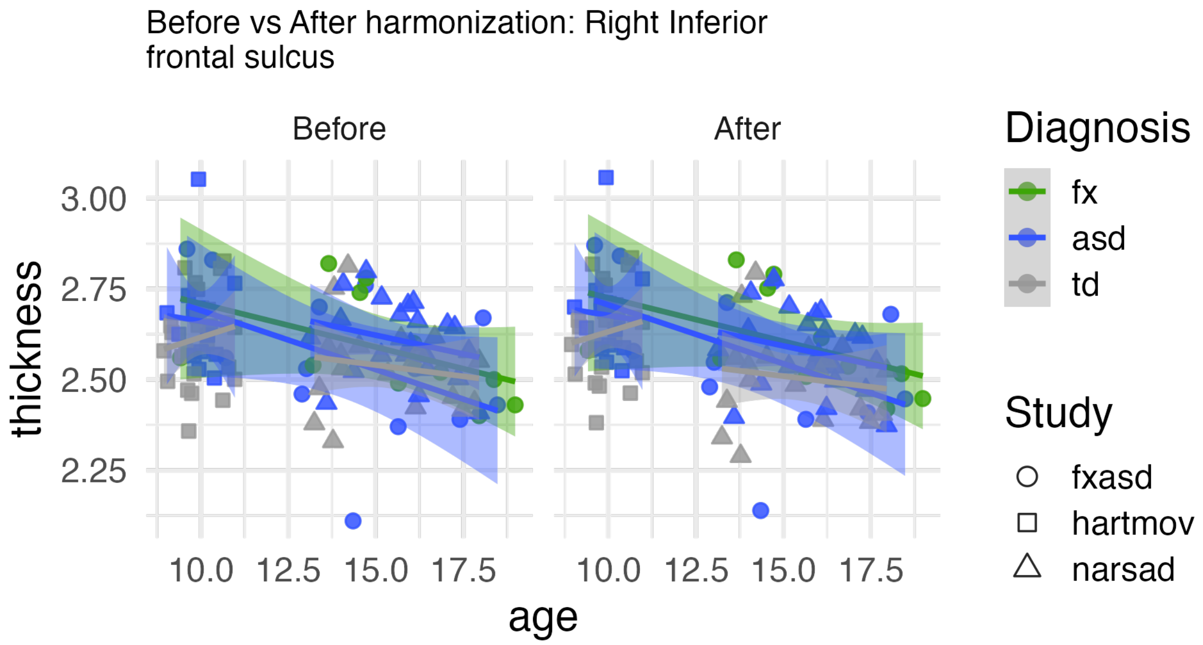
Before vs After harmonization: Right Inferior
frontal sulcus
Before
After
thickness
3.00
2.75
2.50
2.25
10.0
12.5
15.0
17.5
age
Diagnosis
fx
asd
td
Study
fxasd
hartmov
narsad

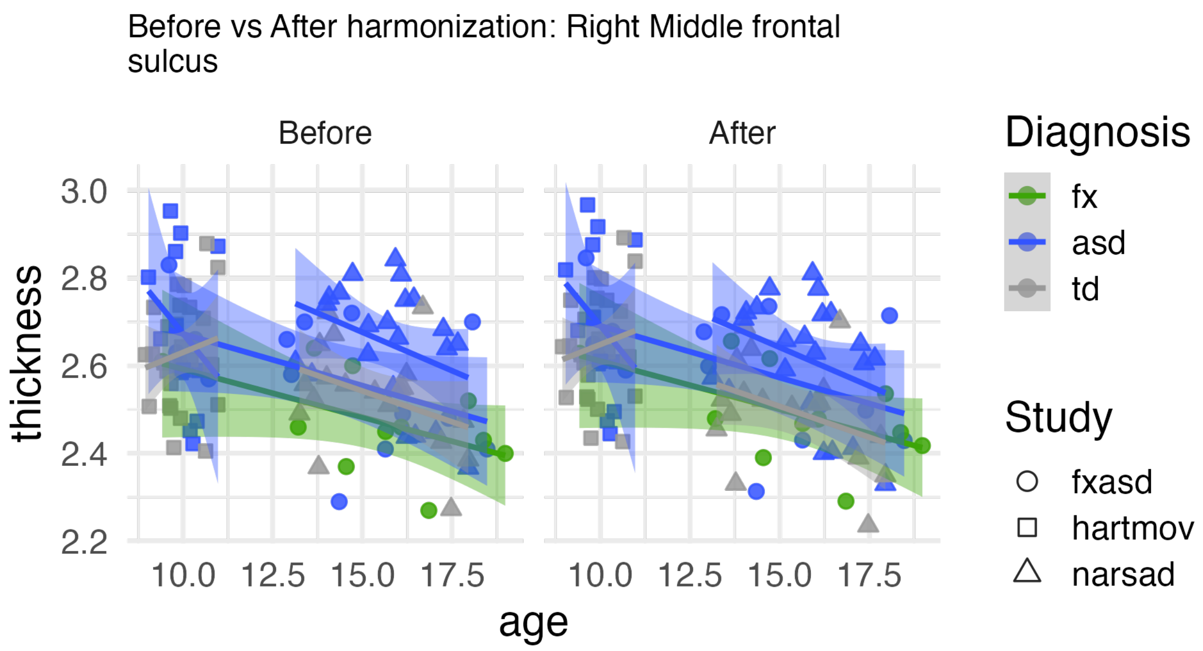

Before vs After harmonization: Right Middle frontal sulcus
Before
After
thickness
3.0
2.8
2.6
2.4
2.2
10.0
12.5
15.0
17.5
age
Diagnosis
fx
asd
td
Study
fxasd
hartmov
narsad

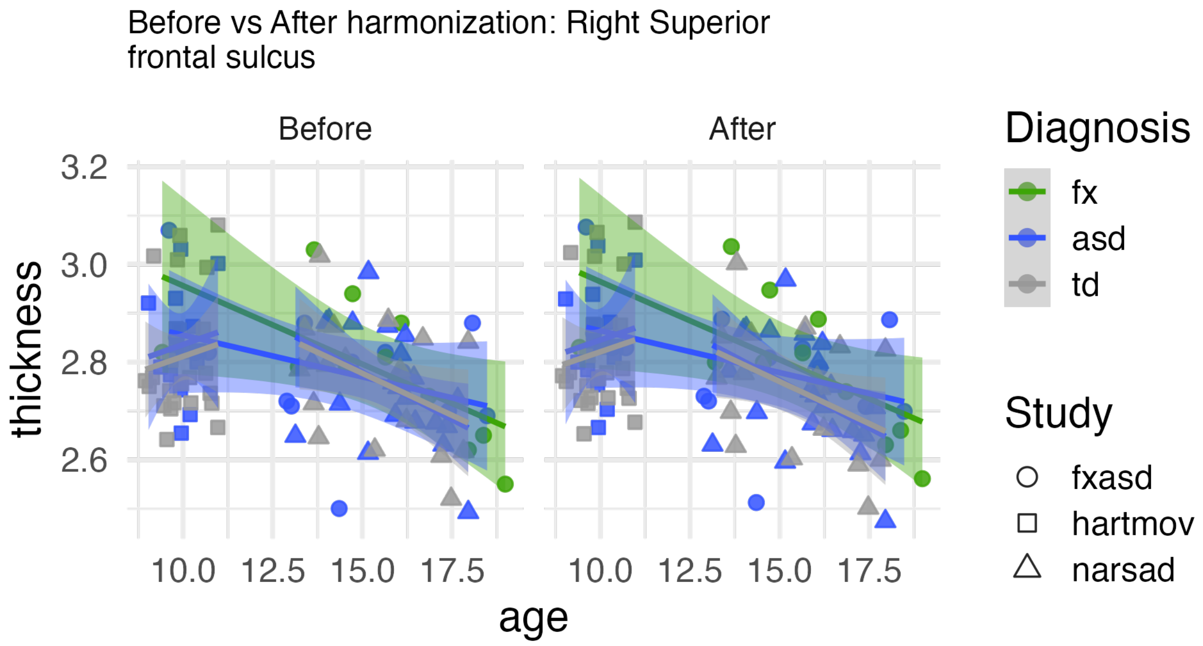

Before vs After harmonization: Right Superior frontal sulcus
Before
After
thickness
3.2
3.0
2.8
2.6
10.0
12.5
15.0
17.5
age
Diagnosis
fx
asd
td
Study
fxasd
hartmov
narsad

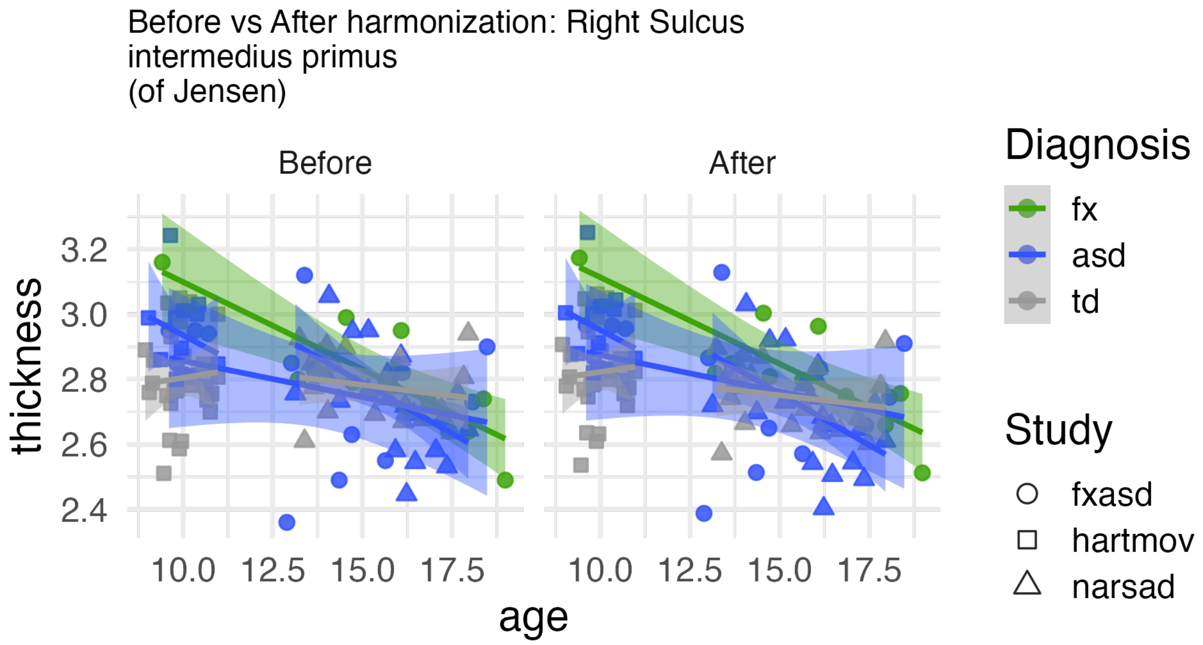

Before vs After harmonization: Right Sulcus
intermedius primus
(of Jensen)
Before
After
thickness
3.2
3.0
2.8
2.6
2.4
10.0
12.5
15.0
17.5
age
Diagnosis
fx
asd
td
Study
fxasd
hartmov
narsad

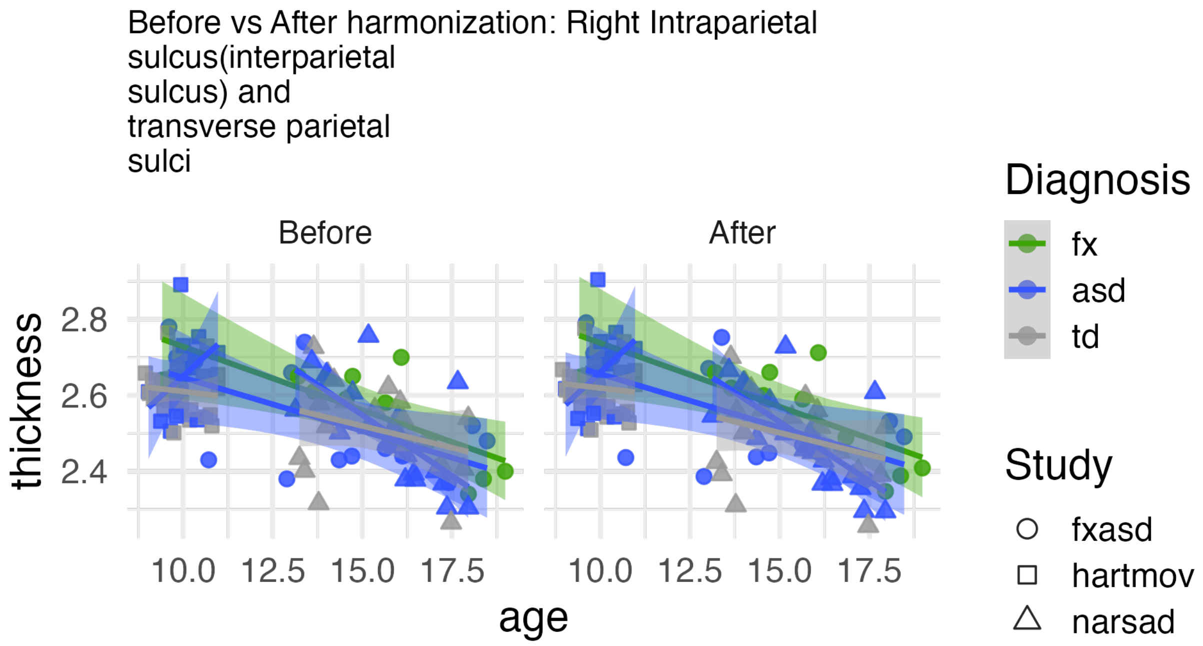

Before vs After harmonization: Right Intraparietal
sulcus(interparietal
sulcus) and
transverse parietal
sulci
Before
After
thickness
2.8
2.6
2.4
10.0
12.5
15.0
17.5
age
Diagnosis
fx
asd
td
Study
fxasd
hartmov
narsad

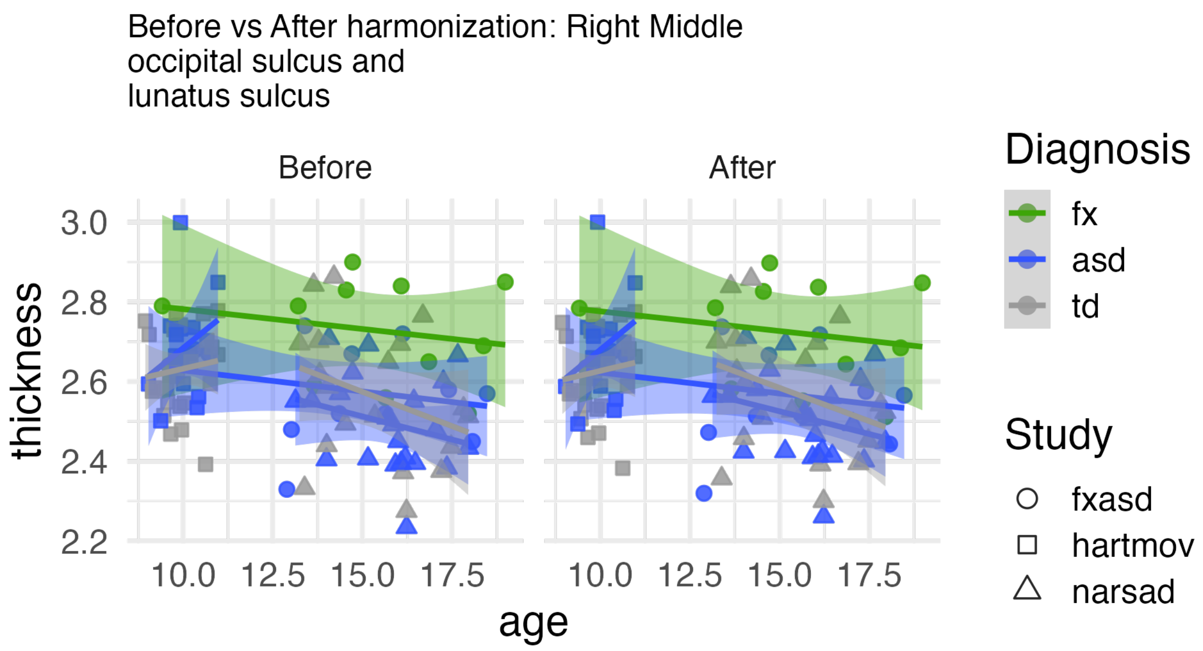

Before vs After harmonization: Right Middle
occipital sulcus and
lunatus sulcus
Before
After
thickness
3.0
2.8
2.6
2.4
2.2
10.0
12.5
15.0
17.5
age
Diagnosis
fx
asd
td
Study
fxasd
hartmov
narsad

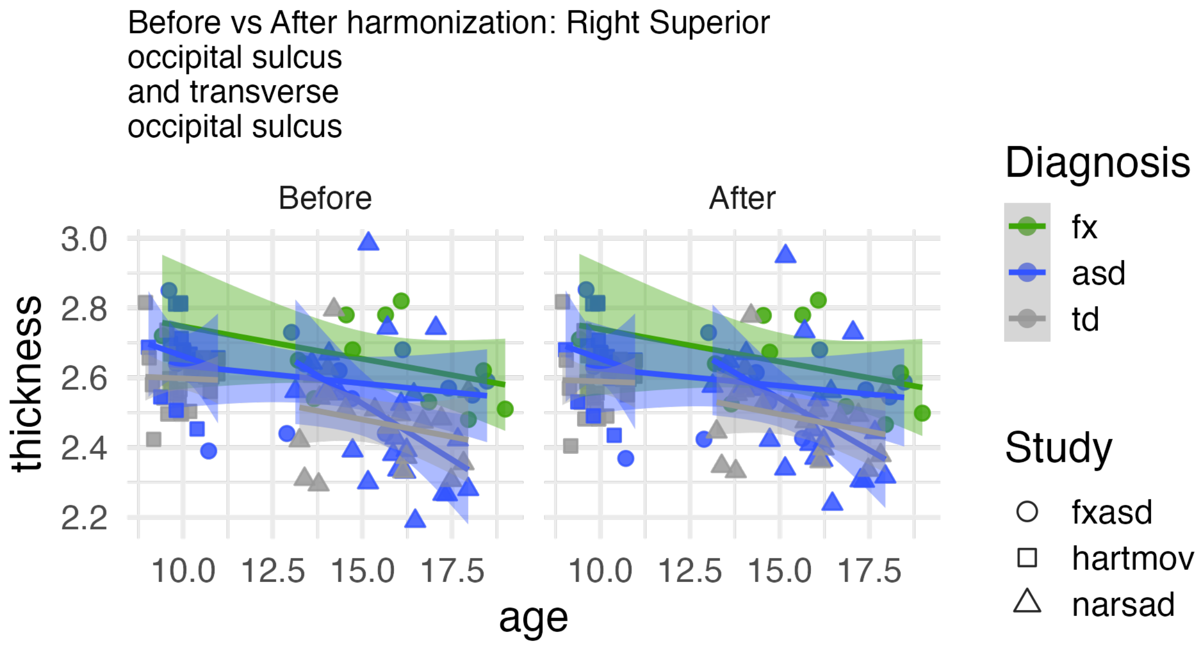

Before vs After harmonization: Right Superior
occipital sulcus
and transverse
occipital sulcus
Before
After
thickness
3.0
2.8
2.6
2.4
2.2
10.0
12.5
15.0
17.5
age
Diagnosis
fx
asd
td
Study
fxasd
hartmov
narsad

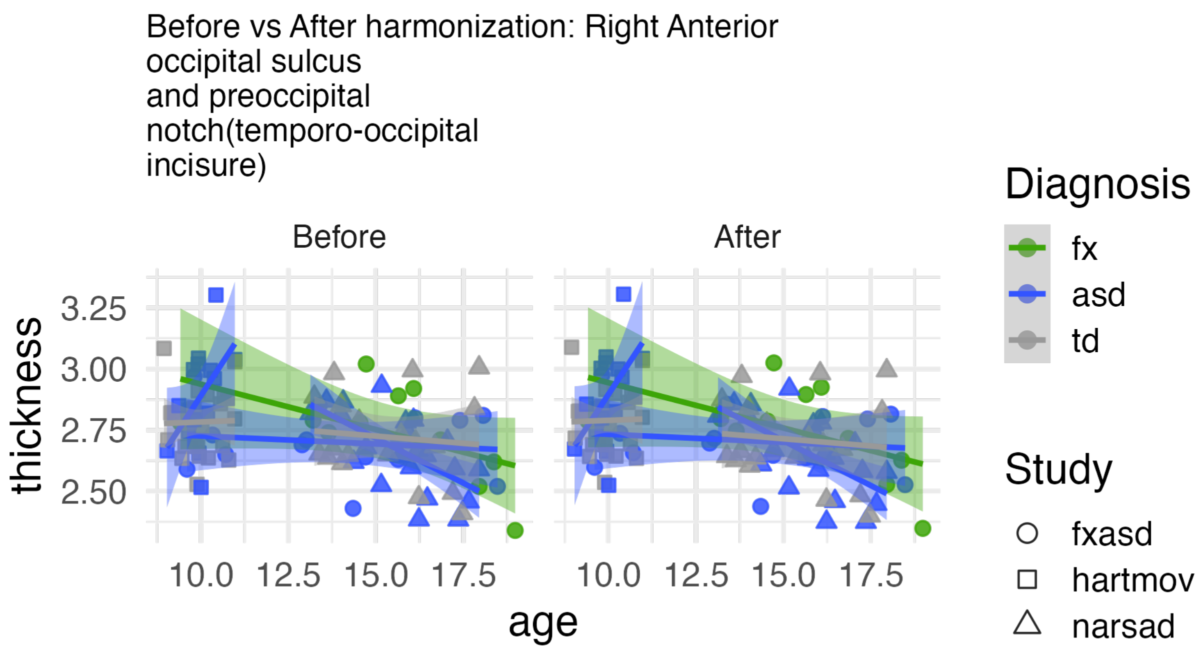
Before vs After harmonization: Right Anterior
occipital sulcus
and preoccipital
notch(temporo-occipital
incisure)
Before
After
thickness
3.25
3.00
2.75
2.50
10.0
12.5
15.0
17.5
age
Diagnosis
fx
asd
td
Study
fxasd
hartmov
narsad

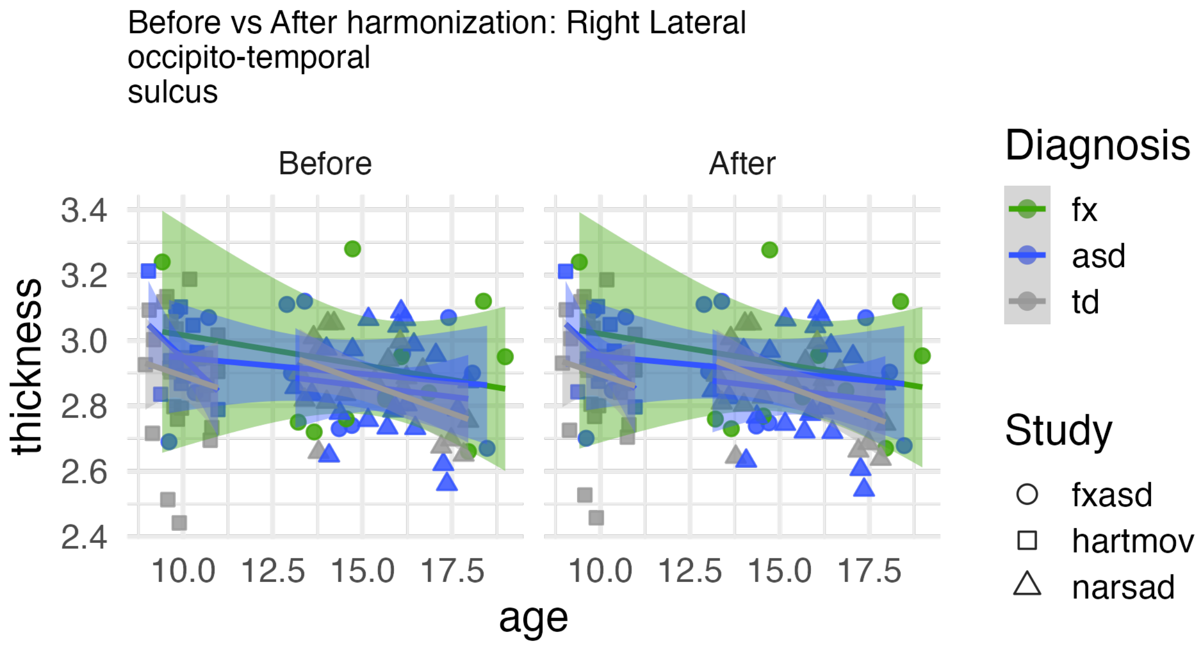
Before vs After harmonization: Right Lateral
occipito-temporal
sulcus
Before
After
thickness
3.4
3.2
3.0
2.8
2.6
2.4
10.0
12.5
15.0
17.5
age
Diagnosis
fx
asd
td
Study
fxasd
hartmov
narsad

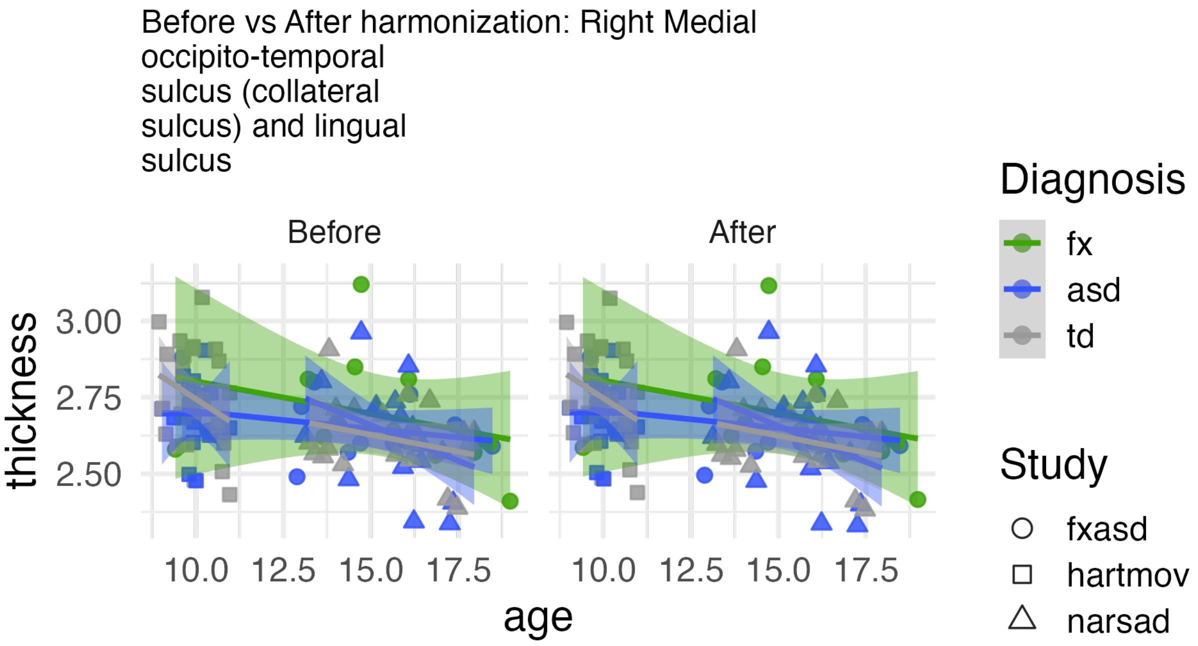

Before vs After harmonization: Right Medial occipito-temporal sulcus (collateral sulcus) and lingual sulcus
Before
After
thickness
3.00
2.75
2.50
10.0
12.5
15.0
17.5
age
Diagnosis
fx
asd
td
Study
fxasd
hartmov
narsad

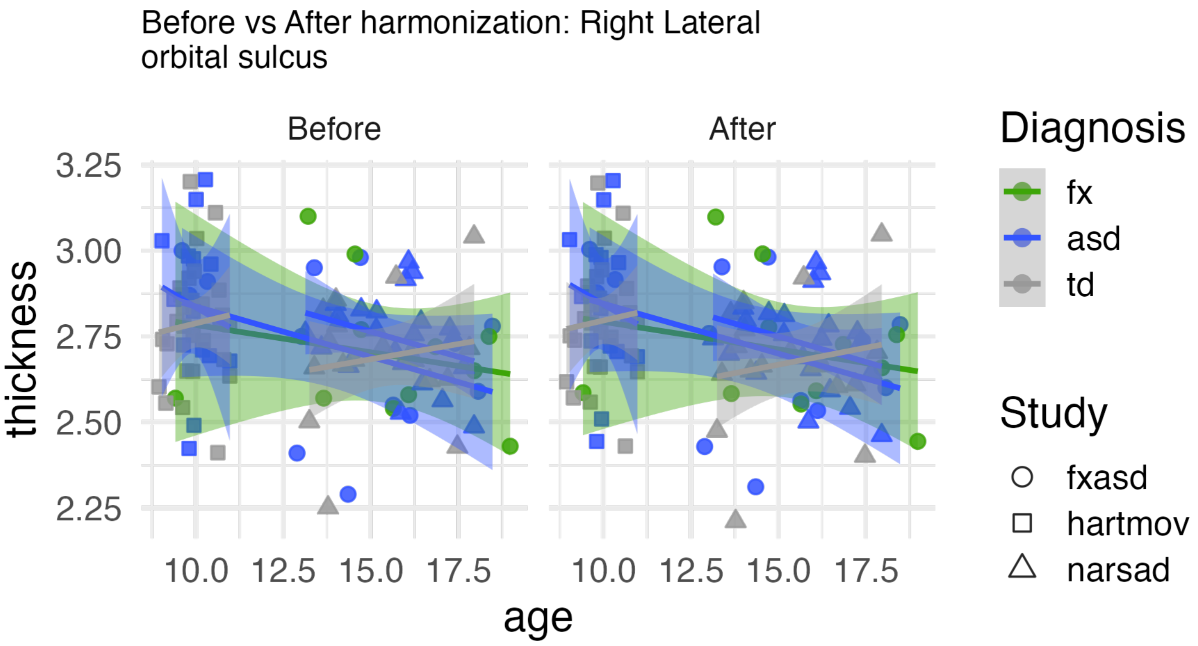

Before vs After harmonization: Right Lateral orbital sulcus
Before
After
thickness
3.25
3.00
2.75
2.50
2.25
10.0
12.5
15.0
17.5
age
Diagnosis
fx
asd
td
Study
fxasd
hartmov
narsad

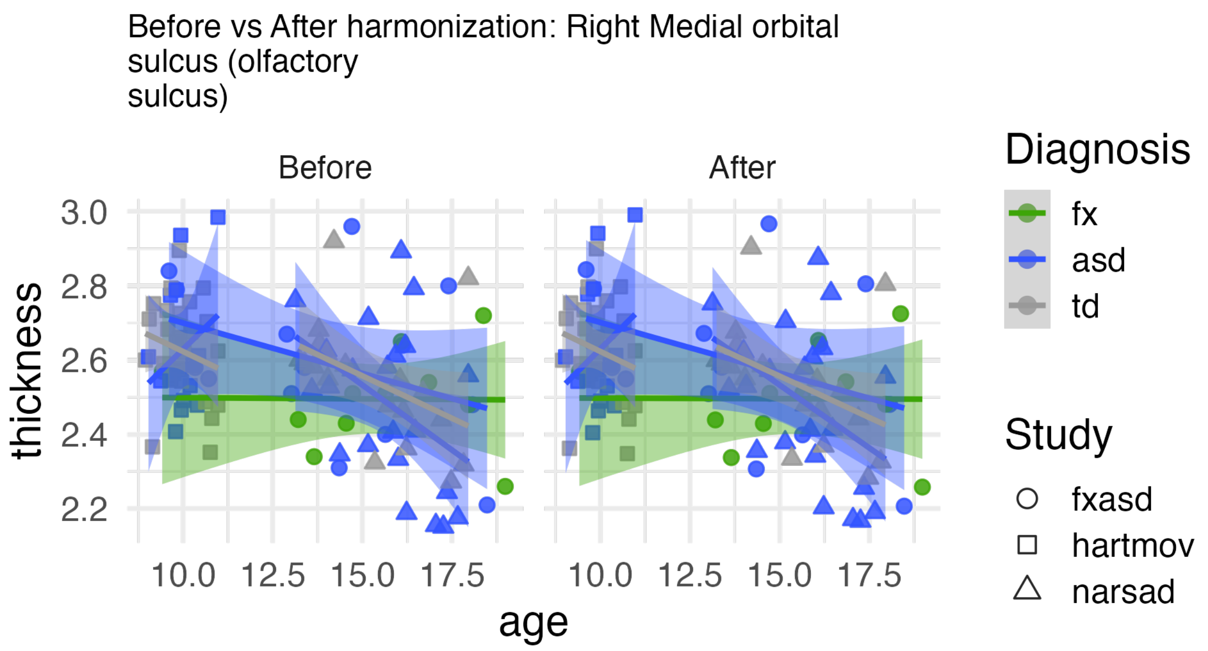
Before vs After harmonization: Right Medial orbital
sulcus (olfactory
sulcus)
Before
After
thickness
3.0
2.8
2.6
2.4
2.2
10.0
12.5
15.0
17.5
age
Diagnosis
fx
asd
td
Study
fxasd
hartmov
narsad

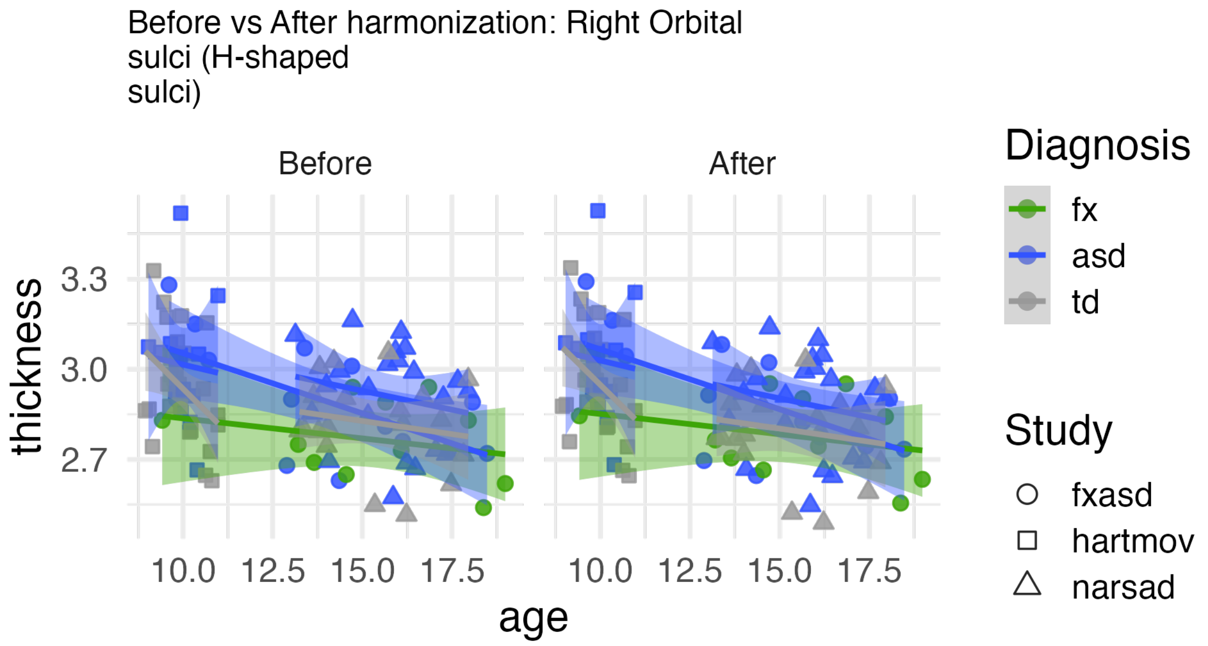
Before vs After harmonization: Right Orbital
sulci (H-shaped
sulci)
Before
After
thickness
3.3
3.0
2.7
10.0
12.5
15.0
17.5
age
Diagnosis
fx
asd
td
Study
fxasd
hartmov
narsad

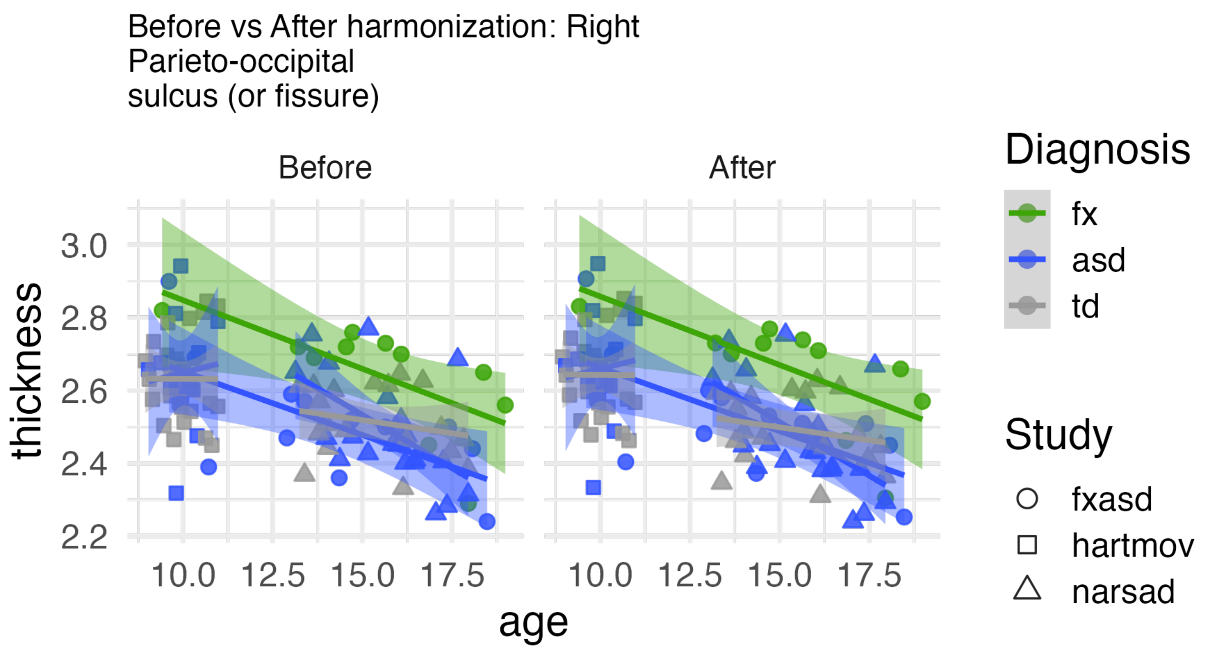
Before vs After harmonization: Right
Parieto-occipital
sulcus (or fissure)
Before
After
thickness
3.0
2.8
2.6
2.4
2.2
10.0
12.5
15.0
17.5
age
Diagnosis
fx
asd
td
Study
fxasd
hartmov
narsad

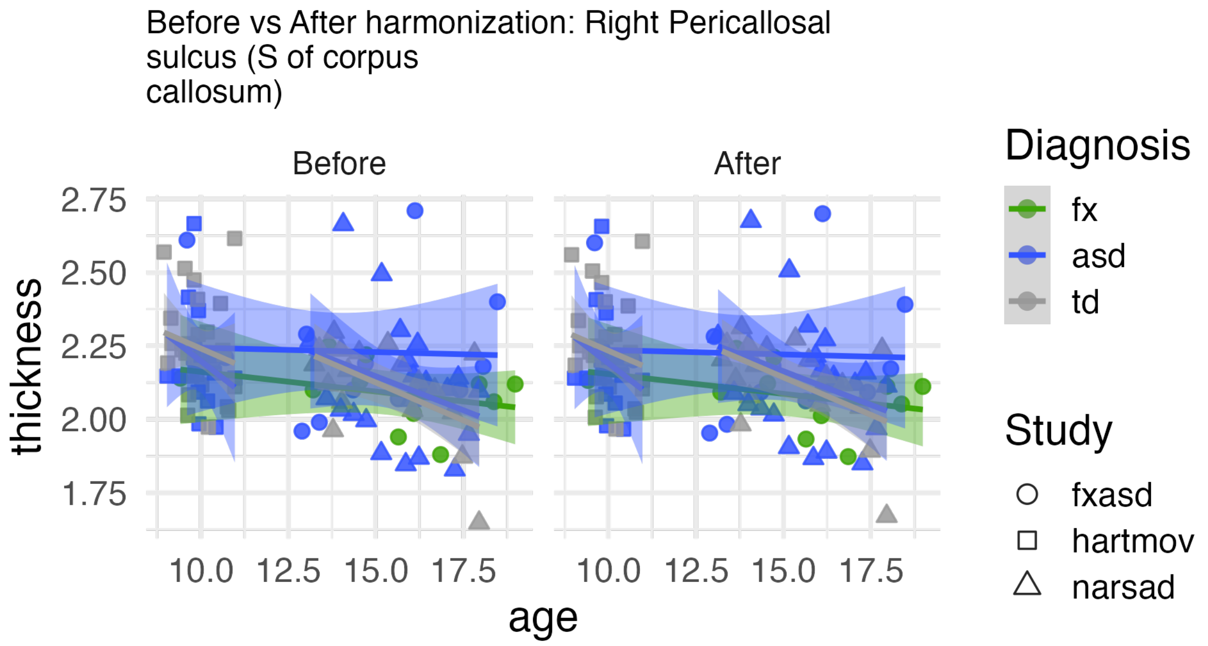
Before vs After harmonization: Right Pericallosal
sulcus (S of corpus
callosum)
Before
After
thickness
2.75
2.50
2.25
2.00
1.75
10.0
12.5
15.0
17.5
age
Diagnosis
fx
asd
td
Study
fxasd
hartmov
narsad

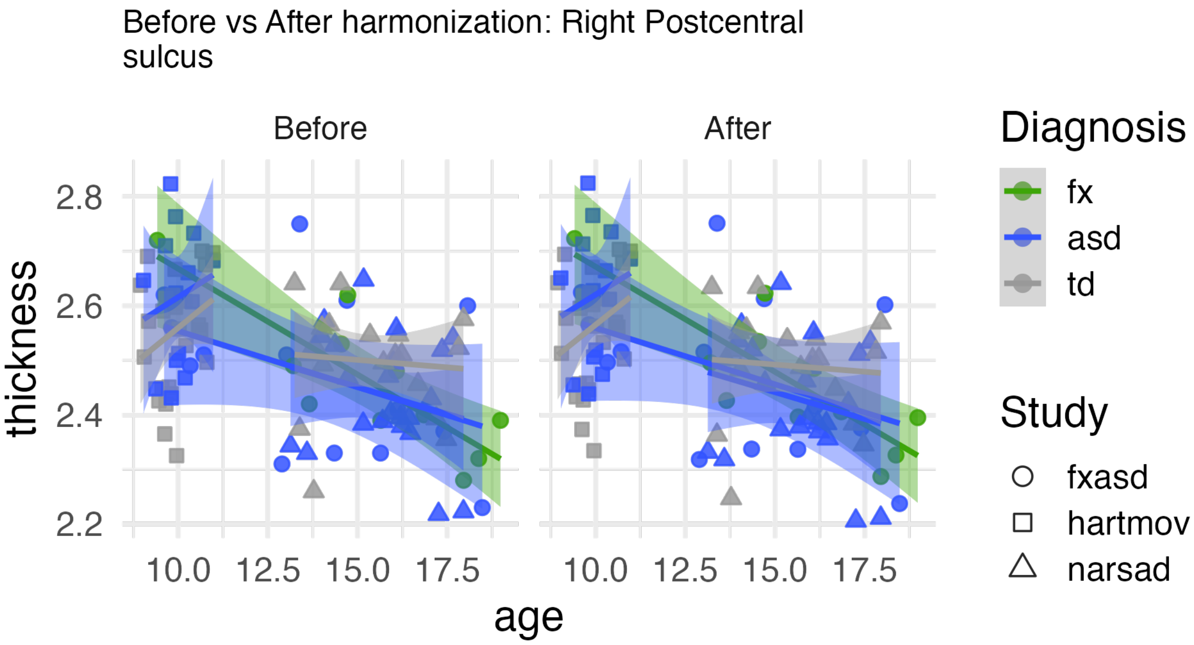
Before vs After harmonization: Right Postcentral sulcus
Before
After
thickness
2.8
2.6
2.4
2.2
10.0
12.5
15.0
17.5
age
Diagnosis
fx
asd
td
Study
fxasd
hartmov
narsad

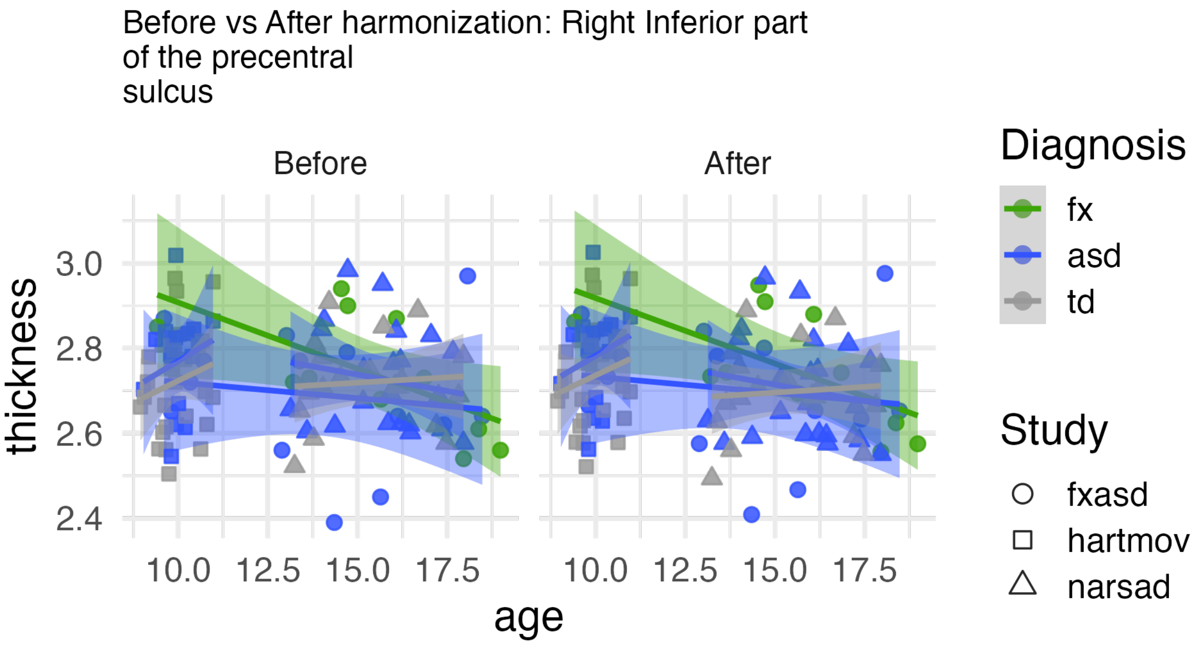
Before vs After harmonization: Right Inferior part of the precentral sulcus
Before
After
thickness
3.0
2.8
2.6
2.4
10.0
12.5
15.0
17.5
age
Diagnosis
fx
asd
td
Study
fxasd
hartmov
narsad

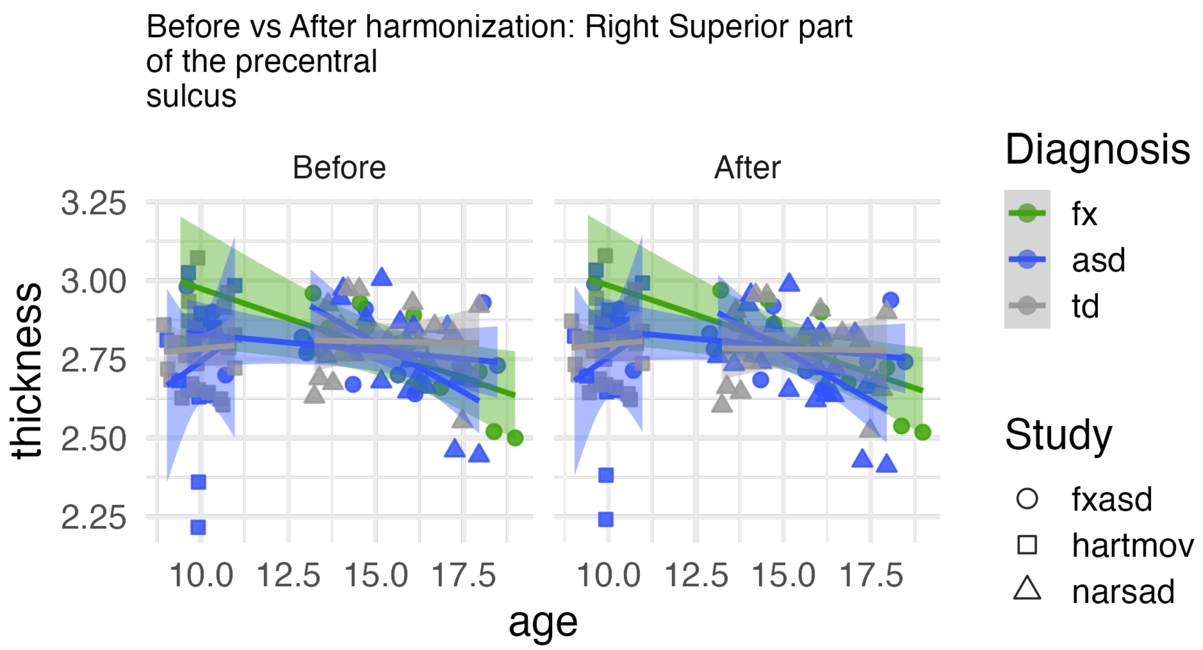
Before vs After harmonization: Right Superior part
of the precentral
sulcus
Before
After
thickness
3.25
3.00
2.75
2.50
2.25
10.0
12.5
15.0
17.5
age
Diagnosis
fx
asd
td
Study
fxasd
hartmov
narsad

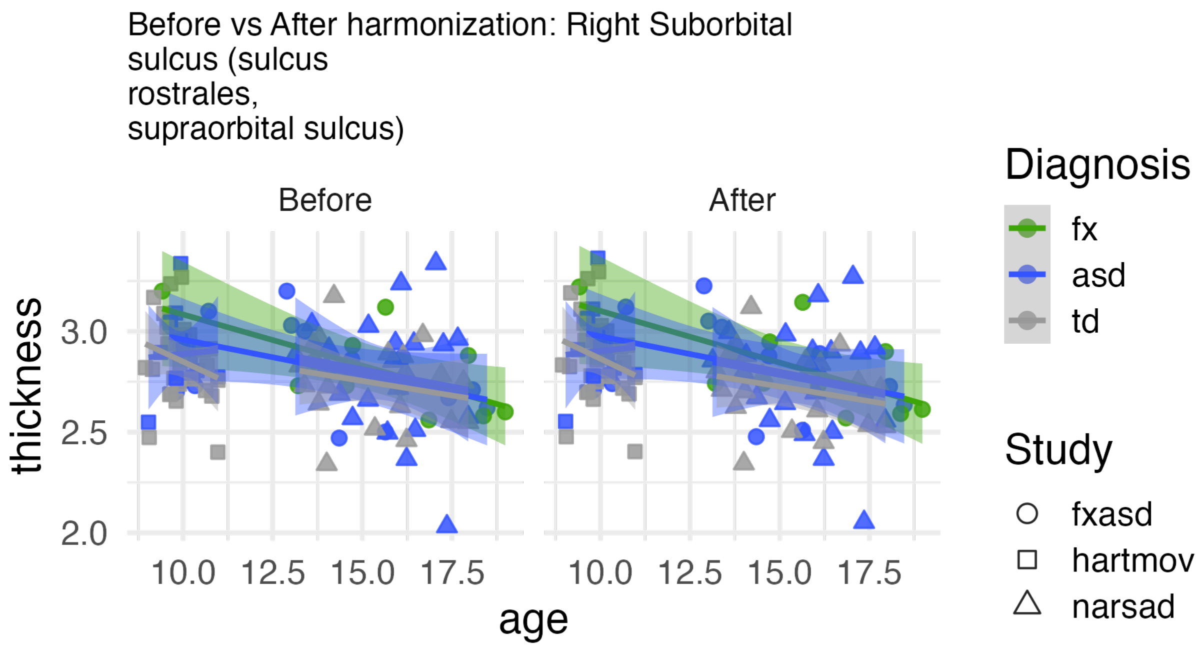
Before vs After harmonization: Right Suborbital
sulcus (sulcus
rostrales,
supraorbital sulcus)
Before
After
thickness
3.0
2.5
2.0
10.0
12.5
15.0
17.5
age
Diagnosis
fx
asd
td
Study
fxasd
hartmov
narsad

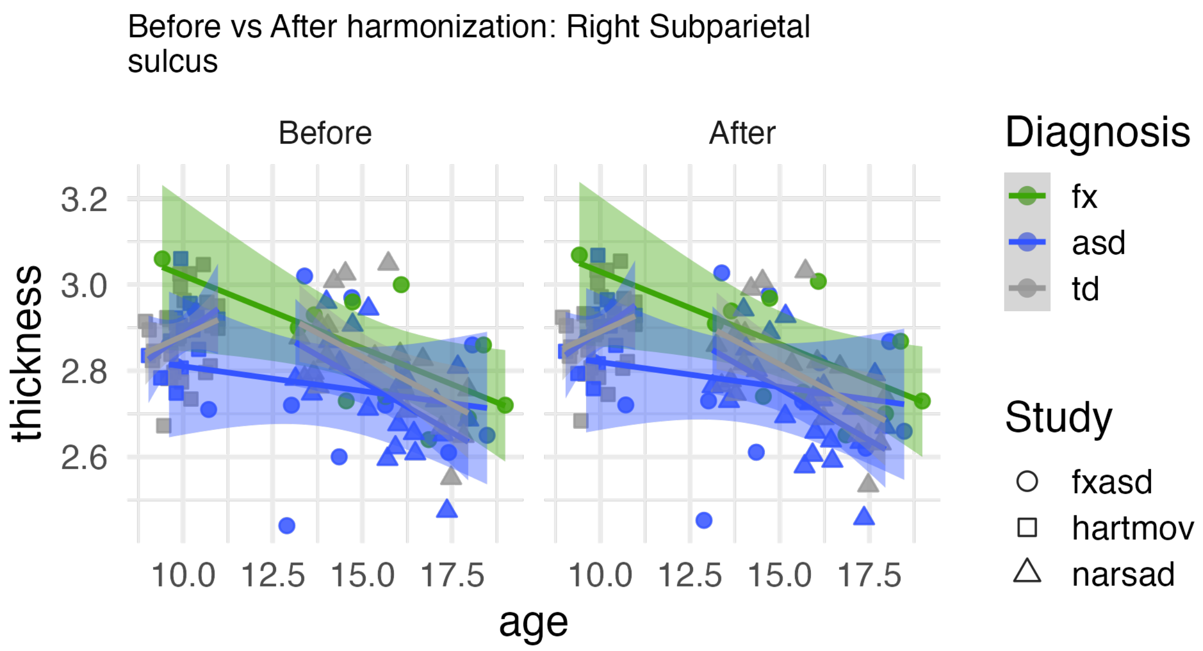
Before vs After harmonization: Right Subparietal sulcus
Before
After
thickness
3.2
3.0
2.8
2.6
10.0
12.5
15.0
17.5
age
Diagnosis
fx
asd
td
Study
fxasd
hartmov
narsad

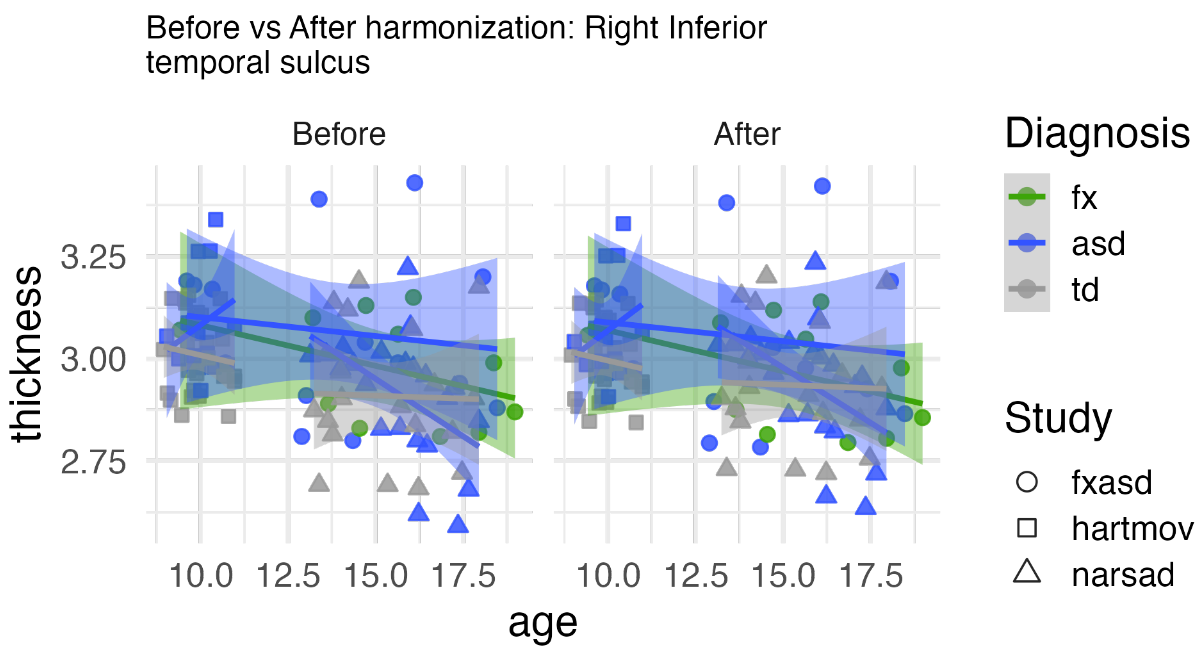
Before vs After harmonization: Right Inferior temporal sulcus
Before
After
thickness
3.25
3.00
2.75
10.0
12.5
15.0
17.5
age
Diagnosis
fx
asd
td
Study
fxasd
hartmov
narsad

Before vs After harmonization: Right Superior temporal
sulcus (parallel
sulcus)

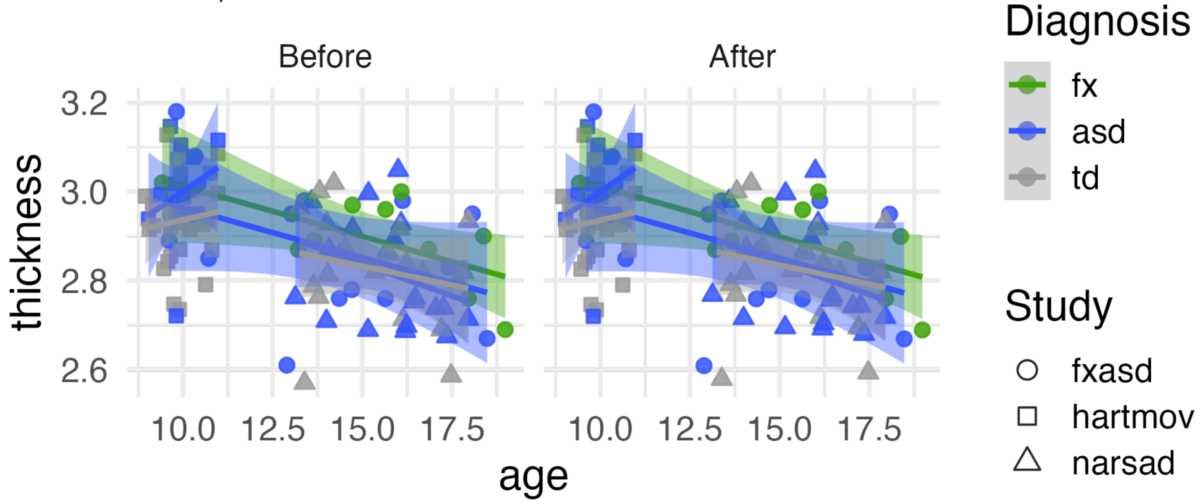

Before vs After harmonization: Right Transverse
temporal sulcus

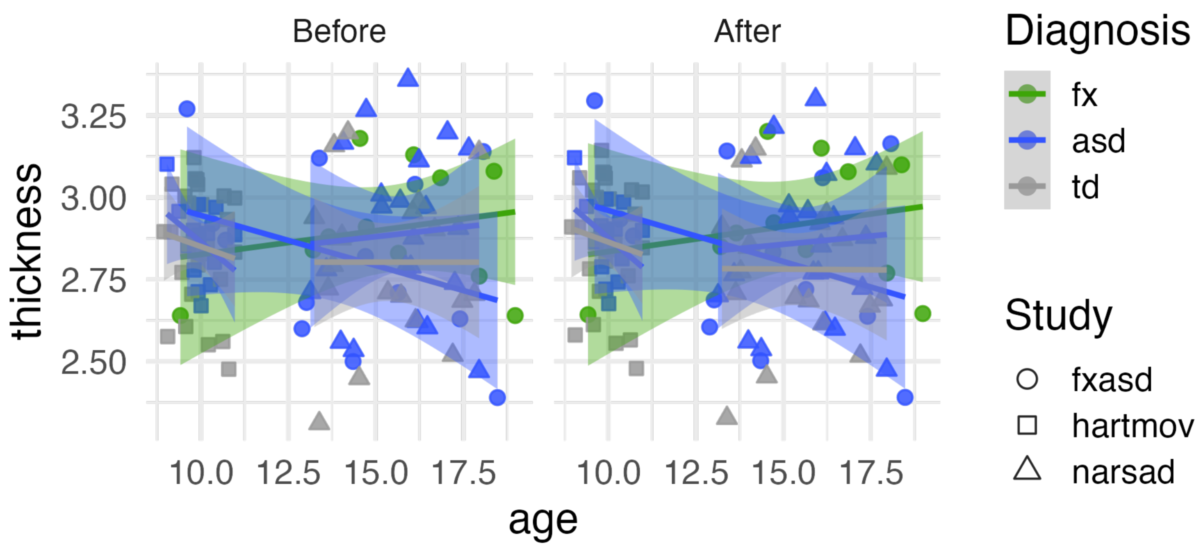

Supplement

1B. qR1 Data Harmonization
Average qR1 measures were extracted from 74 cortical regions per hemisphere of the Destrieux atlas. Data were harmonized across studies using ComBat (Fortin et al., 2017; Richter et al., 2022) with age and diagnosis (ASD, FXS, TD) as covariates and imaging protocol as batch effect. Below, imaging data before and after harmonization are plotted by group, study, and age.

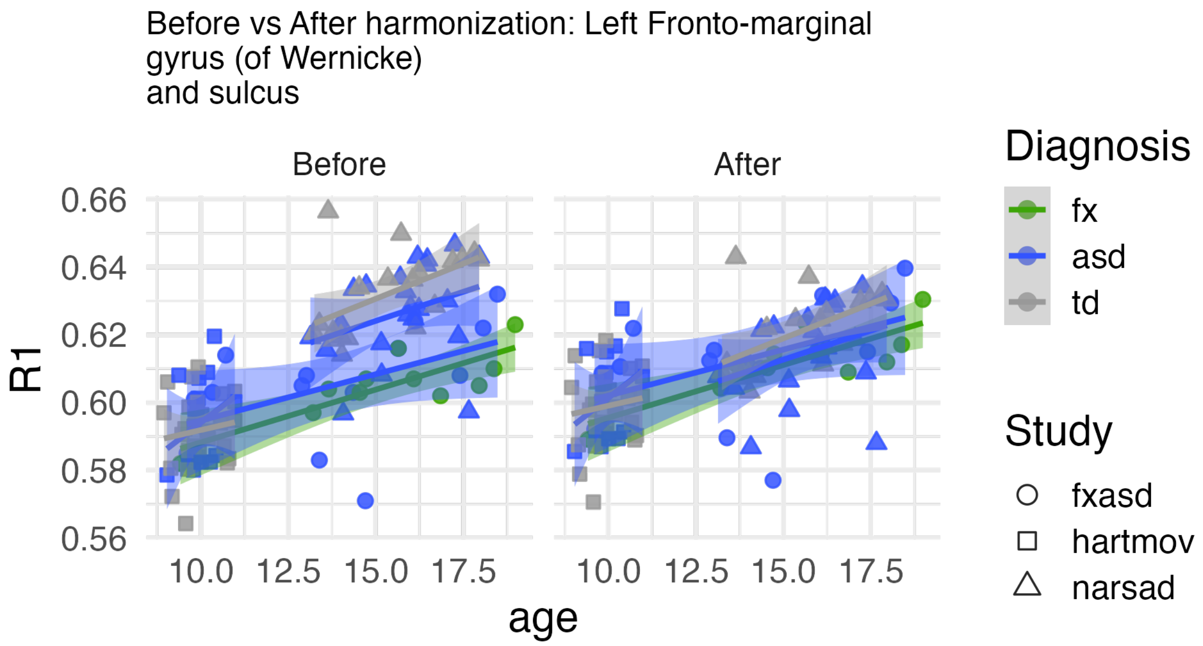

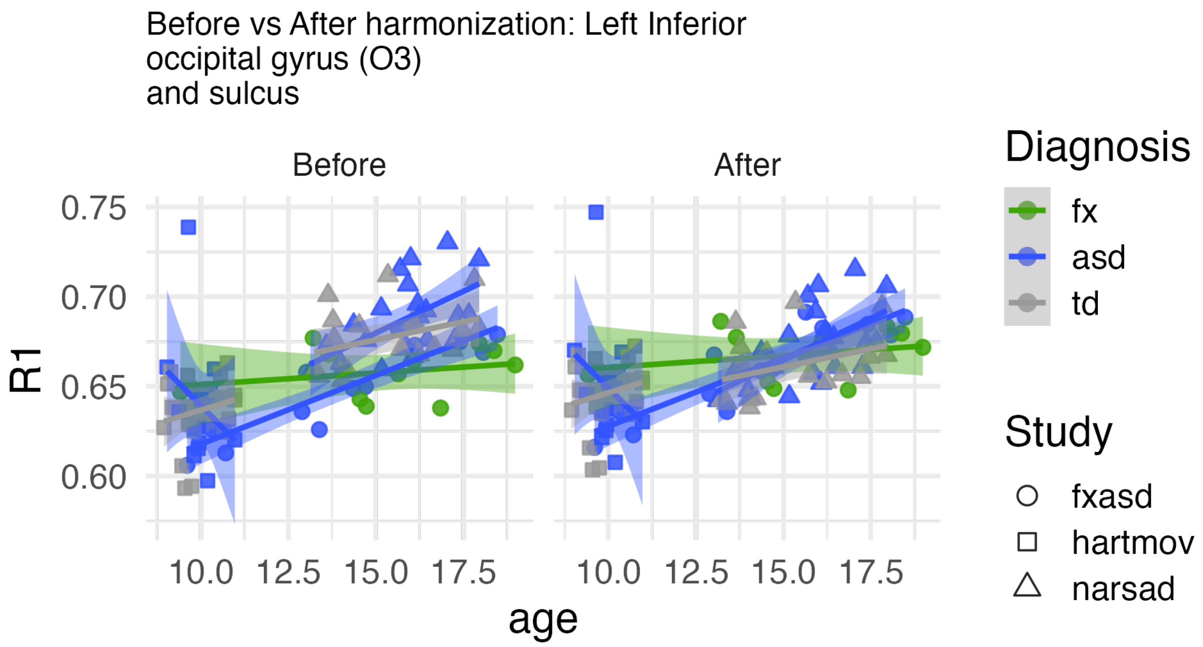

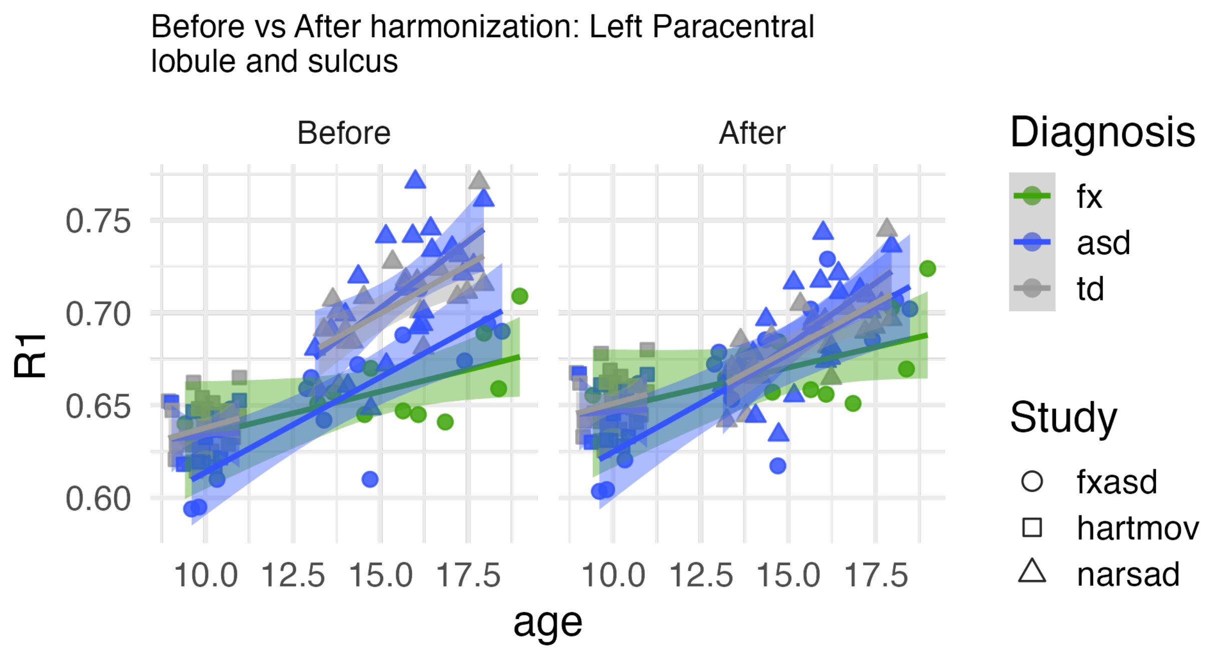
Before vs After harmonization: Left Paracentral
lobule and sulcus
Before
After
R1
0.75
0.70
0.65
0.60
10.0 12.5 15.0 17.5
10.0 12.5 15.0 17.5
age
Diagnosis
fx
asd
td
Study
fxasd
hartmov
narsad

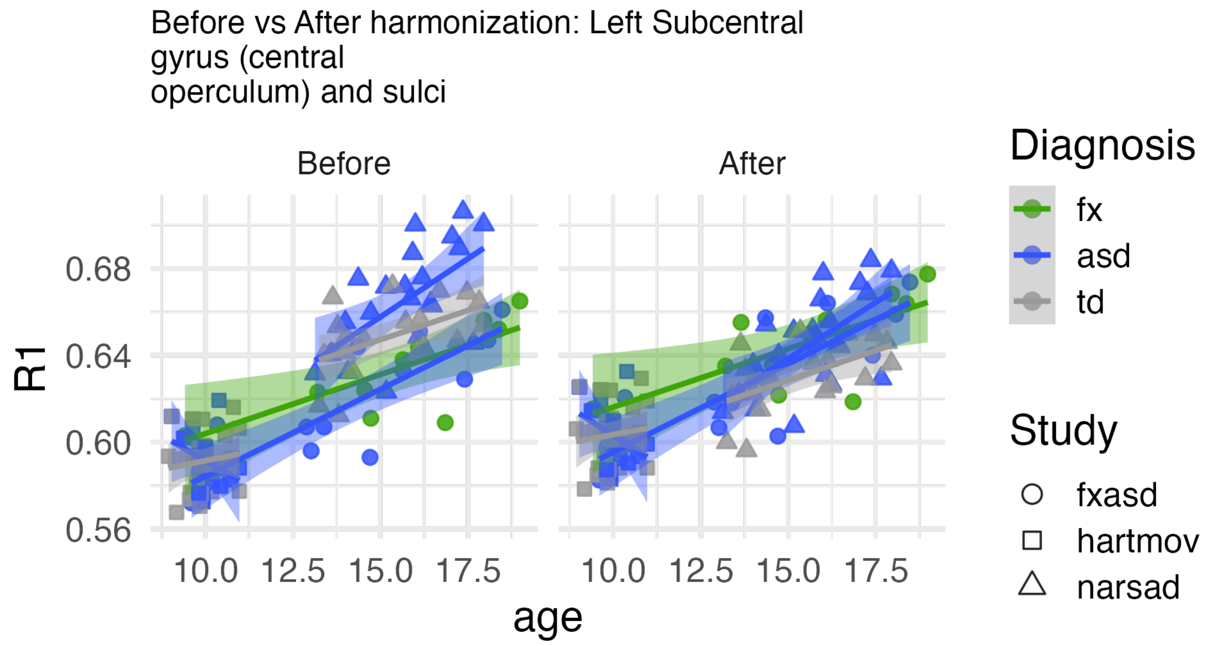
Before vs After harmonization: Left Subcentral
gyrus (central
operculum) and sulci
Before
After
R1
0.68
0.64
0.60
0.56
10.0 12.5 15.0 17.5
10.0 12.5 15.0 17.5
age
Diagnosis
fx
asd
td
Study
fxasd
hartmov
narsad

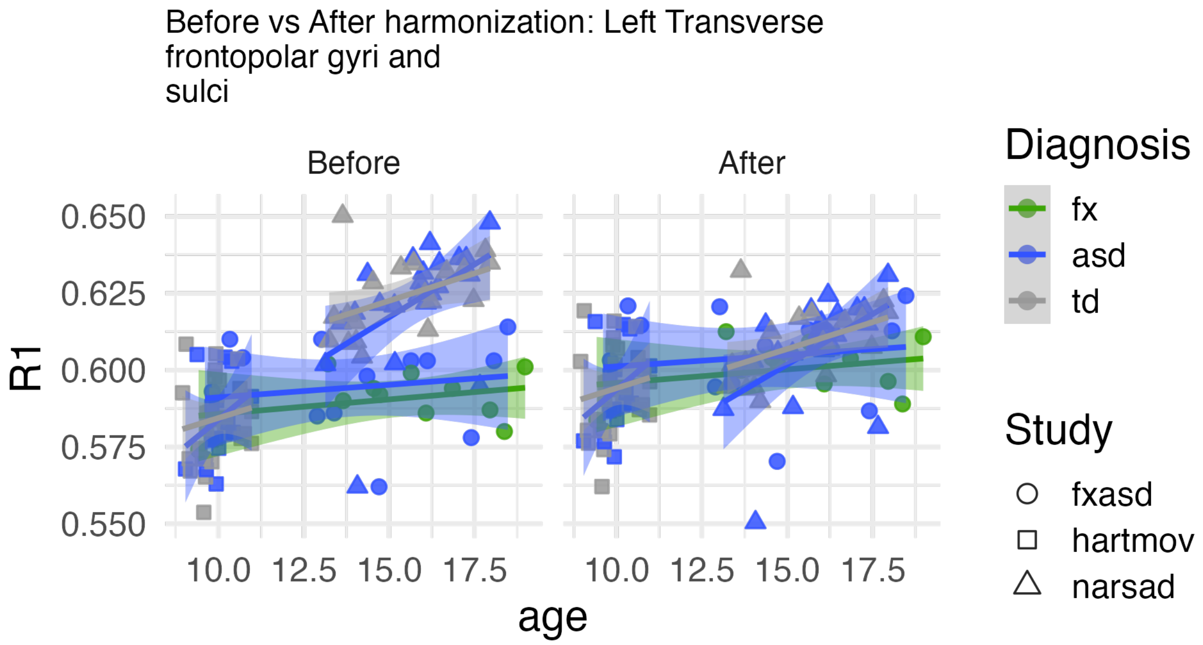
Before vs After harmonization: Left Transverse frontopolar gyri and sulci
Before
After
R1
0.650
0.625
0.600
0.575
0.550
10.0 12.5 15.0 17.5
10.0 12.5 15.0 17.5
age
Diagnosis
fx
asd
td
Study
fxasd
hartmov
narsad

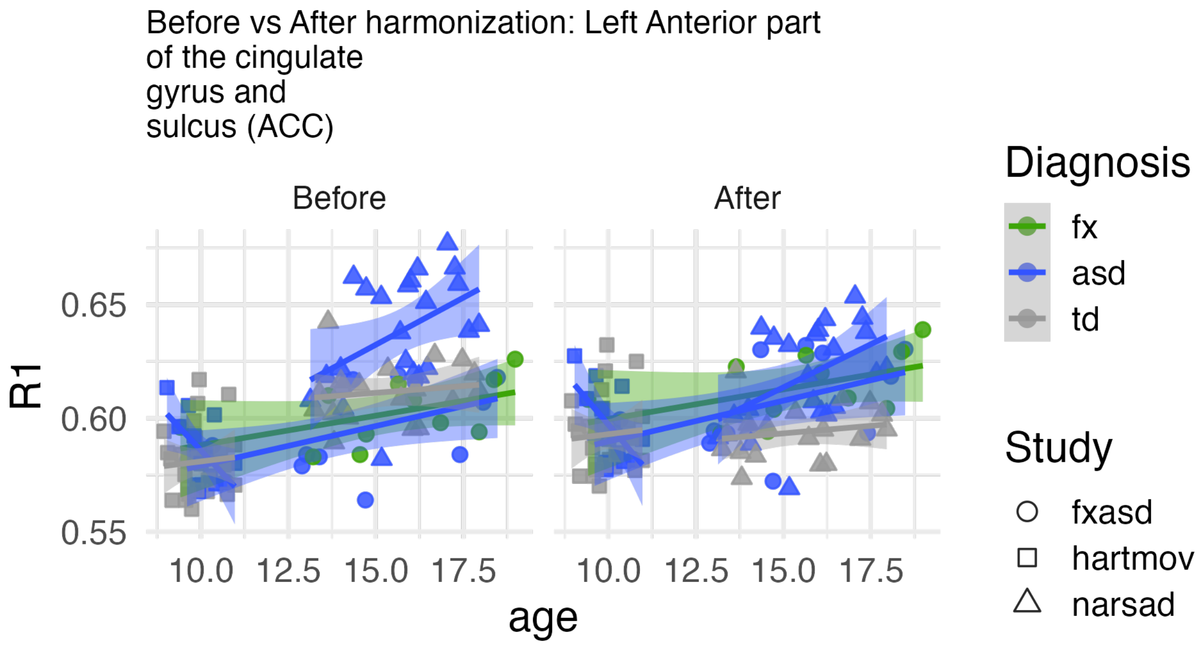
Before vs After harmonization: Left Anterior part of the cingulate gyrus and sulcus (ACC)
Before
After
R1
0.65
0.60
0.55
10.0 12.5 15.0 17.5
10.0 12.5 15.0 17.5
age
Diagnosis
fx
asd
td
Study
fxasd
hartmov
narsad

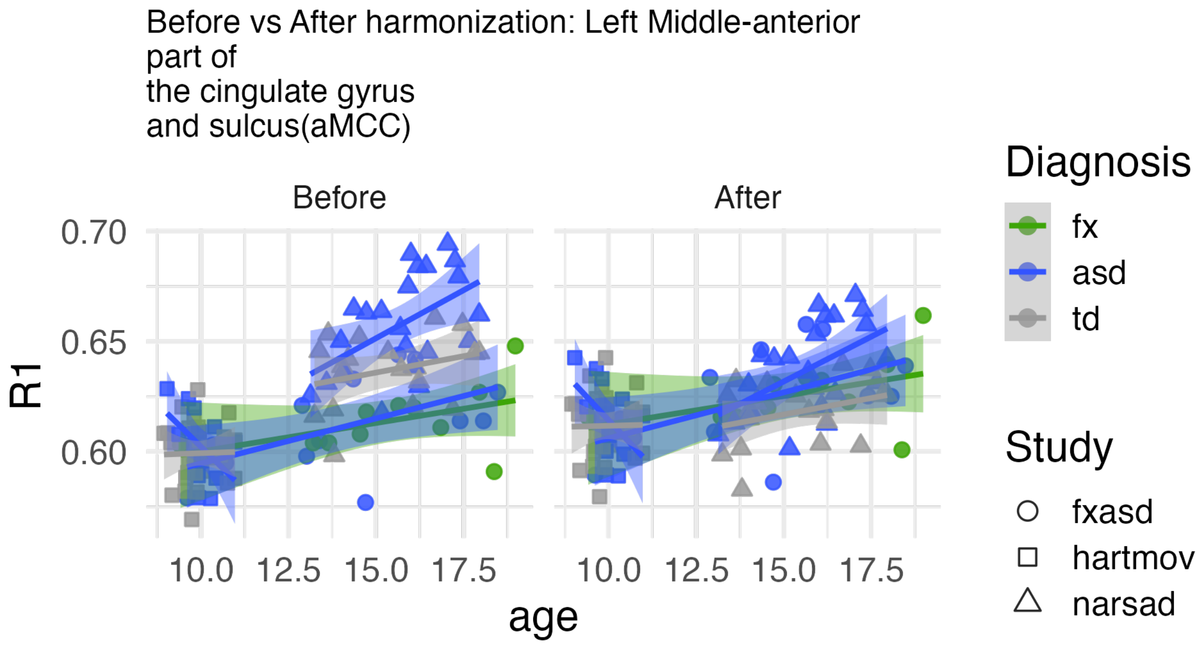
Before vs After harmonization: Left Middle-anterior part of the cingulate gyrus and sulcus(aMCC)
Before
After
R1
0.70
0.65
0.60
10.0 12.5 15.0 17.5
10.0 12.5 15.0 17.5
age
Diagnosis
fx
asd
td
Study
fxasd
hartmov
narsad

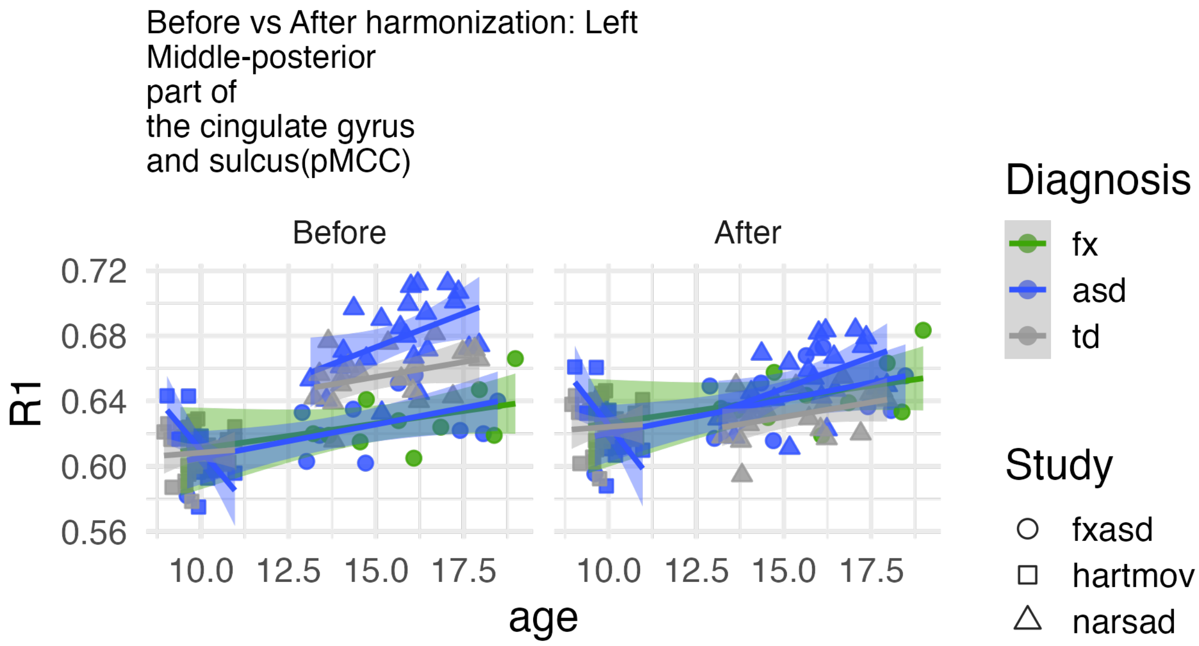
Before vs After harmonization: Left Middle-posterior part of the cingulate gyrus and sulcus(pMCC)
Before
After
R1
0.72
0.68
0.64
0.60
0.56
10.0 12.5 15.0 17.5
10.0 12.5 15.0 17.5
age
Diagnosis
fx
asd
td
Study
fxasd
hartmov
narsad

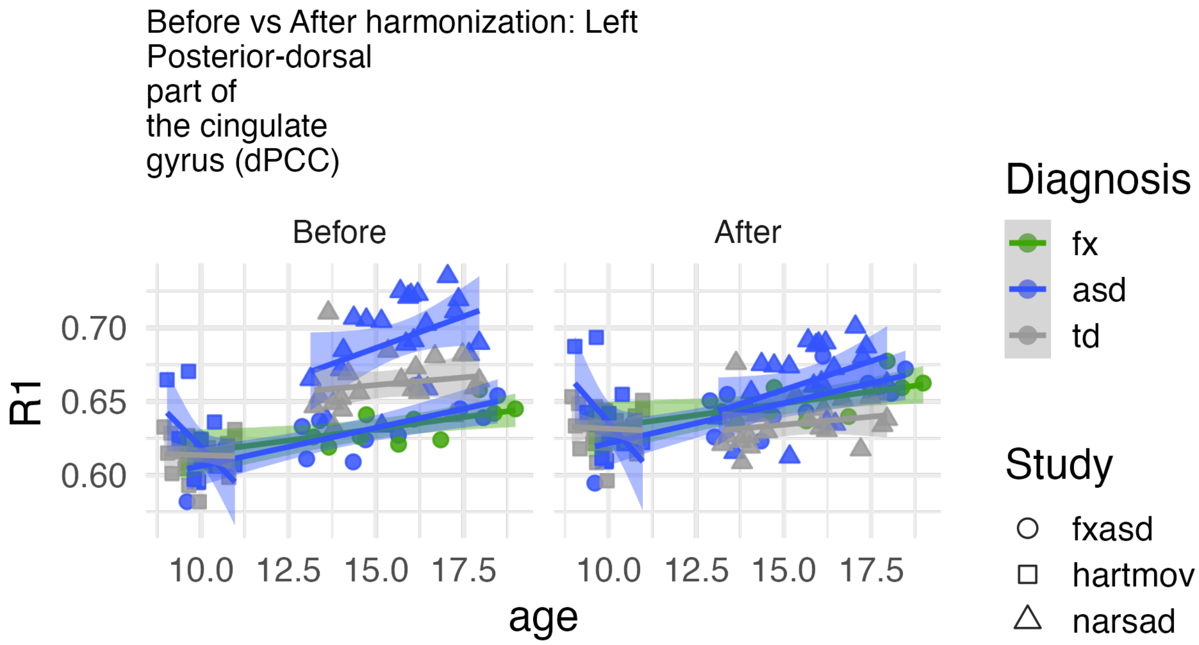
Before vs After harmonization: Left
Posterior-dorsal
part of
the cingulate
gyrus (dPCC)
Before
After
R1
0.70
0.65
0.60
10.0
12.5
15.0
17.5
age
Diagnosis
fx
asd
td
Study
fxasd
hartmov
narsad

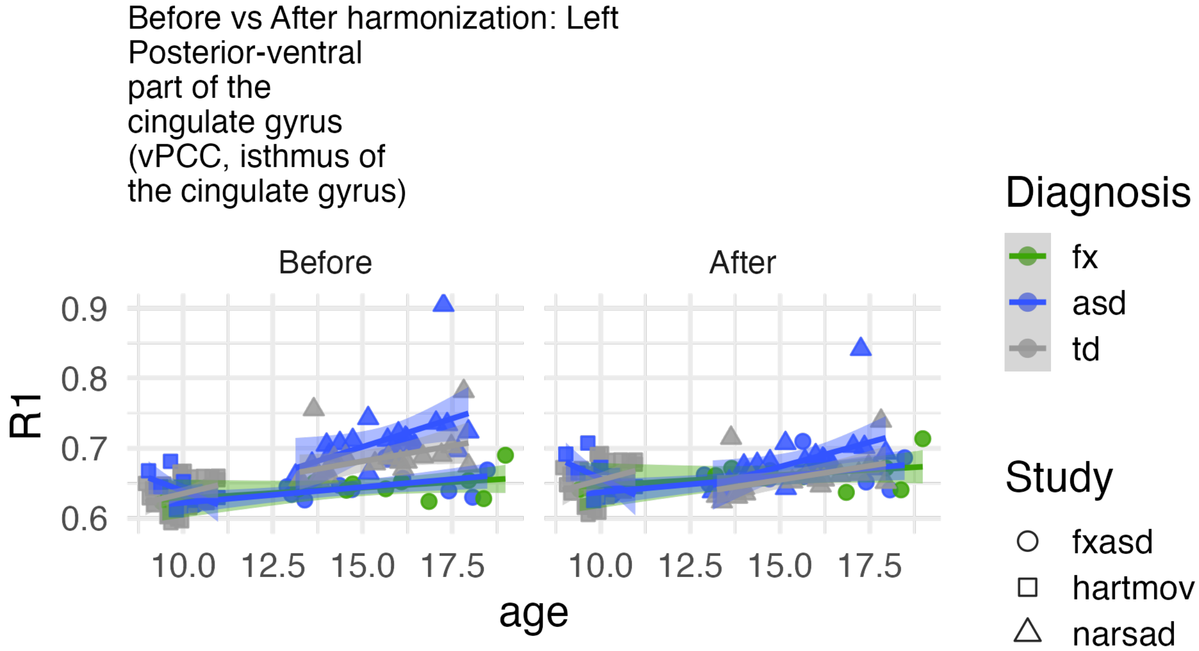
Before vs After harmonization: Left
Posterior-ventral
part of the
cingulate gyrus
(vPCC, isthmus of
the cingulate gyrus)
Before
After
R1
0.9
0.8
0.7
0.6
10.0
12.5
15.0
17.5
age
Diagnosis
fx
asd
td
Study
fxasd
hartmov
narsad

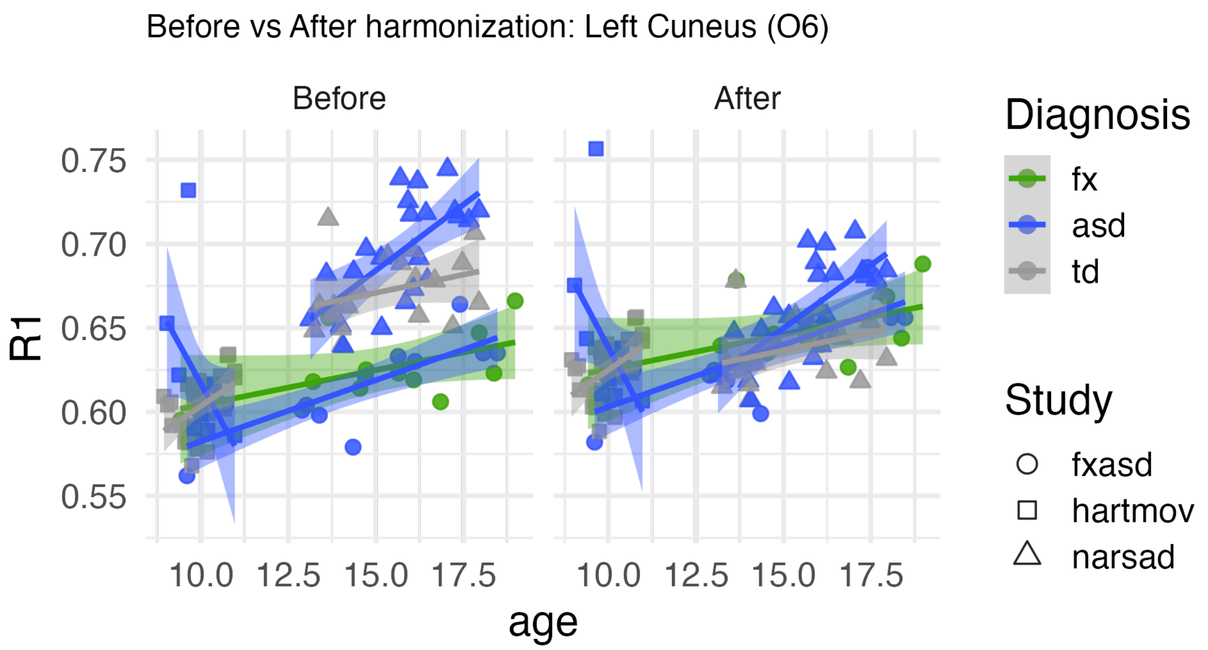
Before vs After harmonization: Left Cuneus (O6)
Before
After
R1
0.75
0.70
0.65
0.60
0.55
10.0
12.5
15.0
17.5
age
Diagnosis
fx
asd
td
Study
fxasd
hartmov
narsad

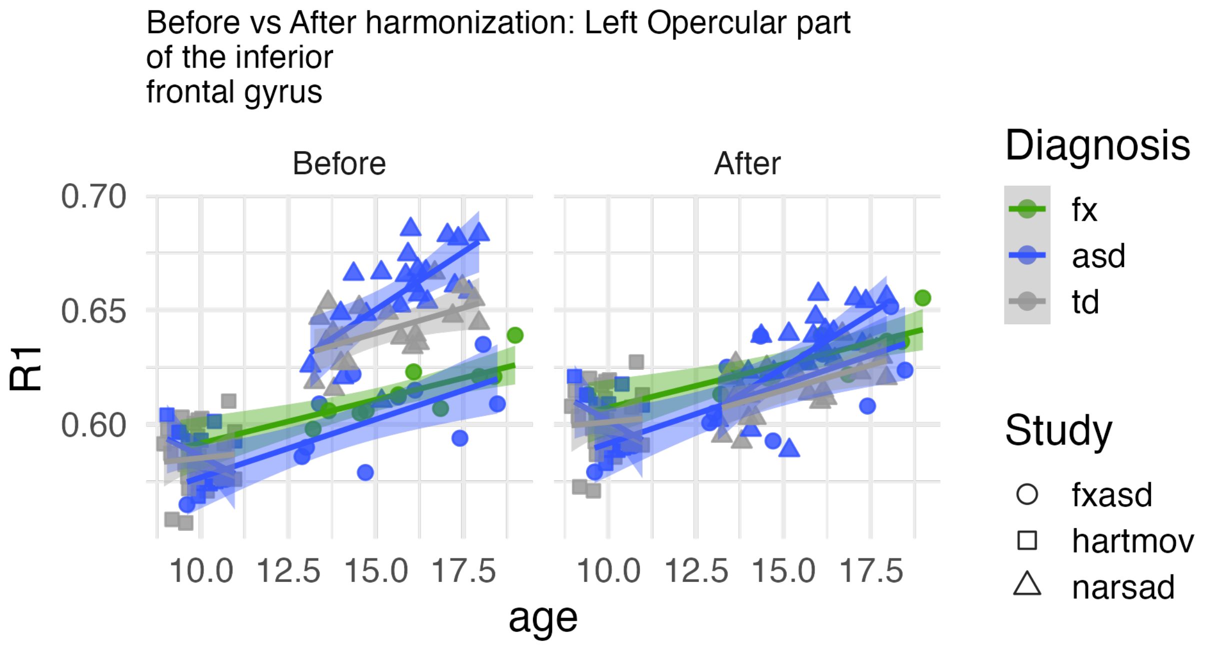
Before vs After harmonization: Left Opercular part
of the inferior
frontal gyrus
Before
After
R1
0.70
0.65
0.60
10.0
12.5
15.0
17.5
age
Diagnosis
fx
asd
td
Study
fxasd
hartmov
narsad

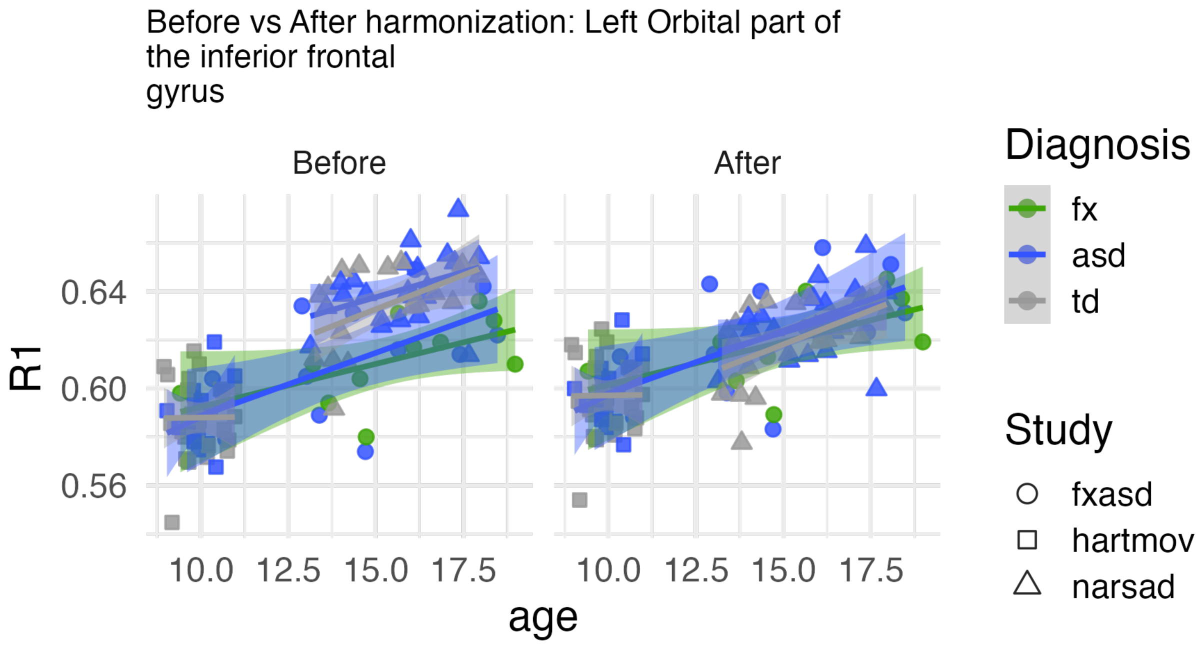
Before vs After harmonization: Left Orbital part of the inferior frontal gyrus
Before
After
R1
0.64
0.60
0.56
10.0
12.5
15.0
17.5
age
Diagnosis
fx
asd
td
Study
fxasd
hartmov
narsad

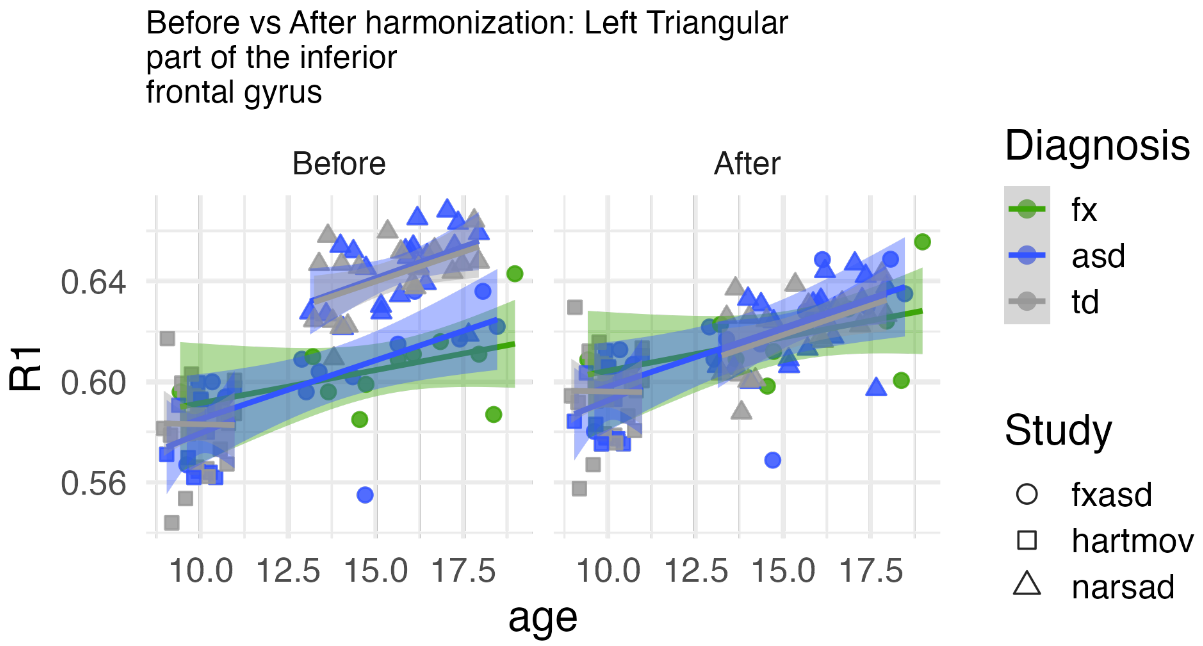
Before vs After harmonization: Left Triangular part of the inferior frontal gyrus
Before
After
R1
0.64
0.60
0.56
10.0
12.5
15.0
17.5
age
Diagnosis
fx
asd
td
Study
fxasd
hartmov
narsad

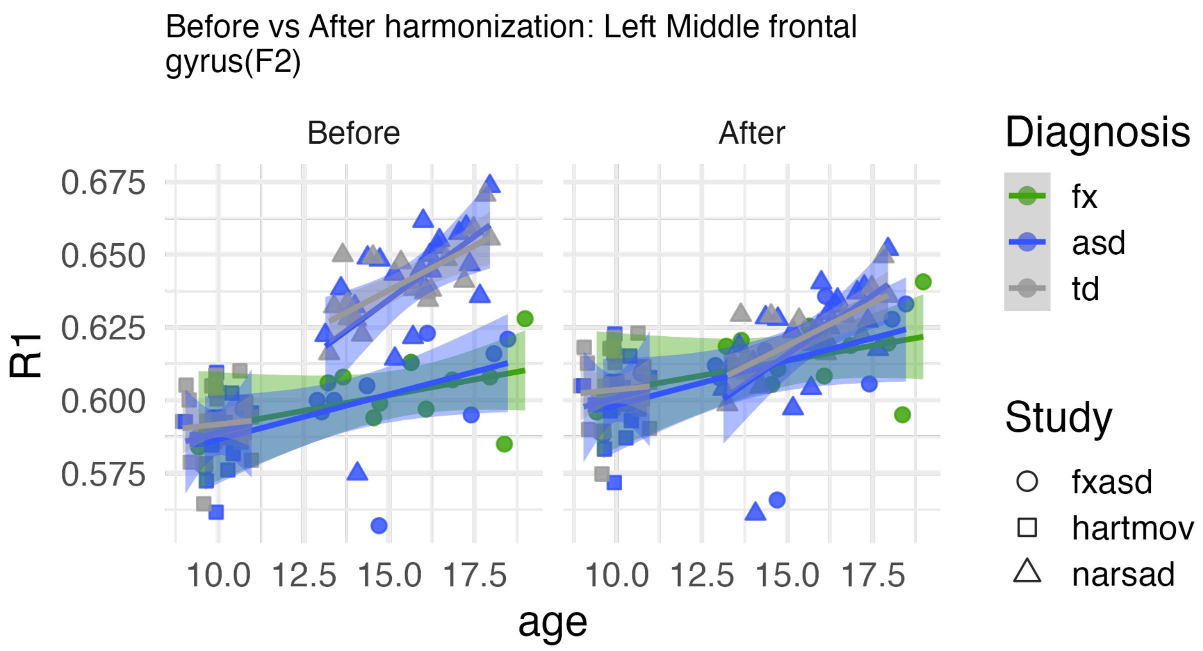
Before vs After harmonization: Left Middle frontal gyrus(F2)
Before
After
0.675
0.650
0.625
0.600
0.575
R1
10.0
12.5
15.0
17.5
age
Diagnosis
fx
asd
td
Study
fxasd
hartmov
narsad

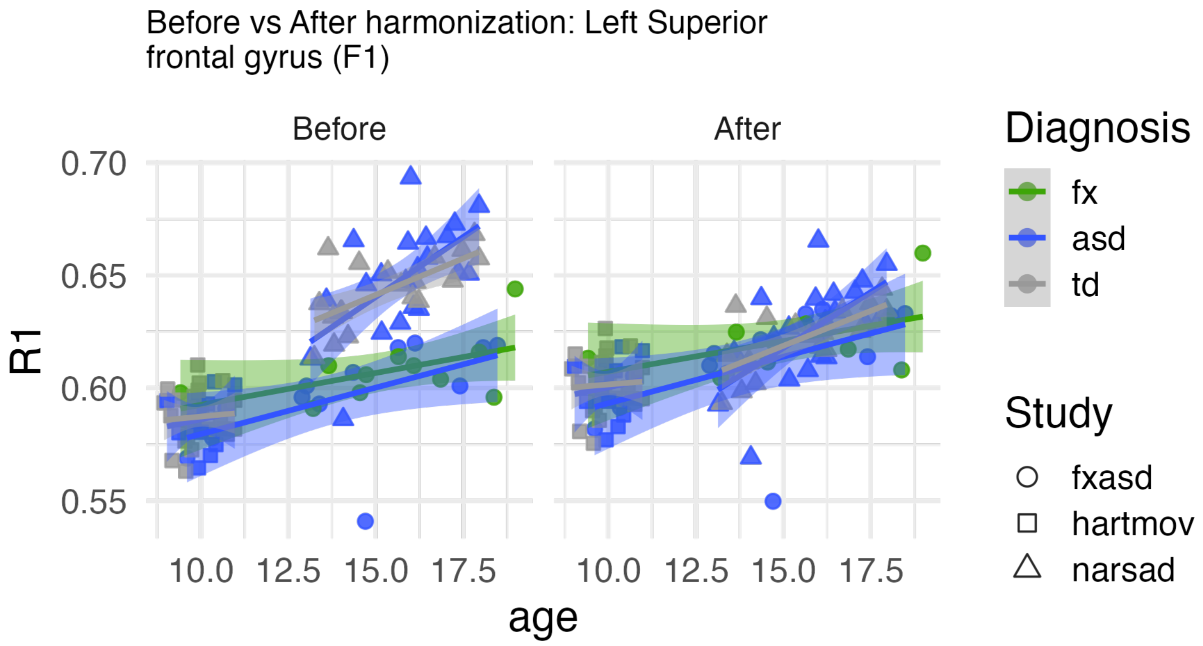
Before vs After harmonization: Left Superior frontal gyrus (F1)
Before
After
0.70
0.65
0.60
0.55
R1
10.0
12.5
15.0
17.5
age
Diagnosis
fx
asd
td
Study
fxasd
hartmov
narsad

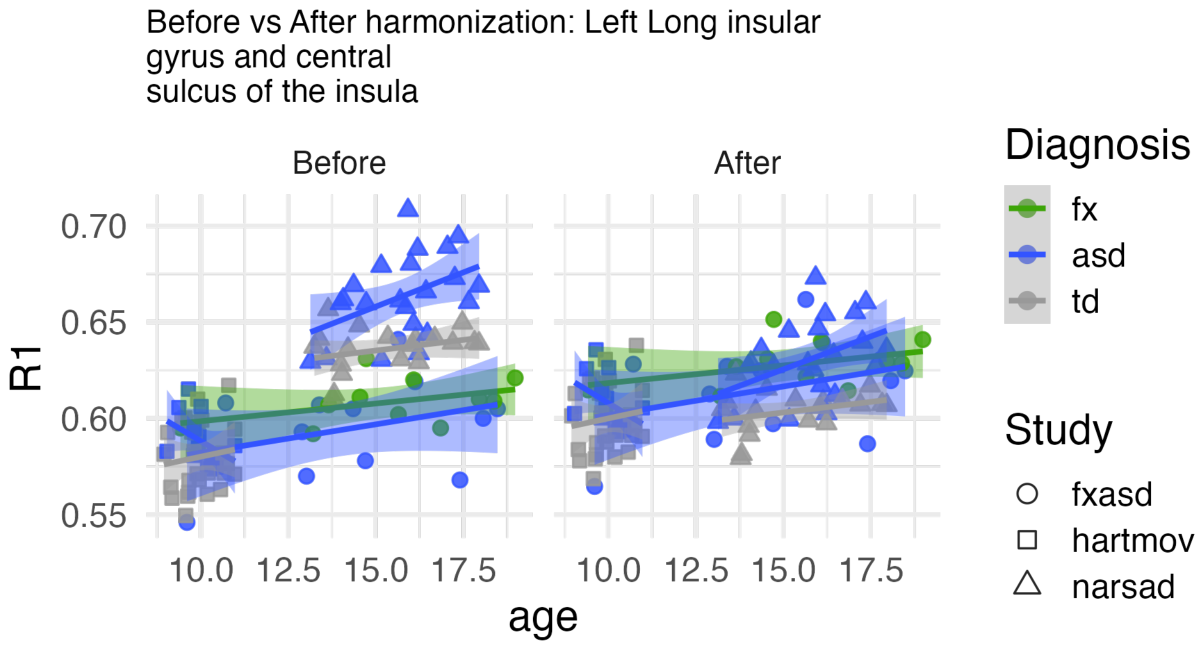
Before vs After harmonization: Left Long insular
gyrus and central
sulcus of the insula
Before
After
R1
0.70
0.65
0.60
0.55
10.0
12.5
15.0
17.5
age
Diagnosis
fx
asd
td
Study
fxasd
hartmov
narsad

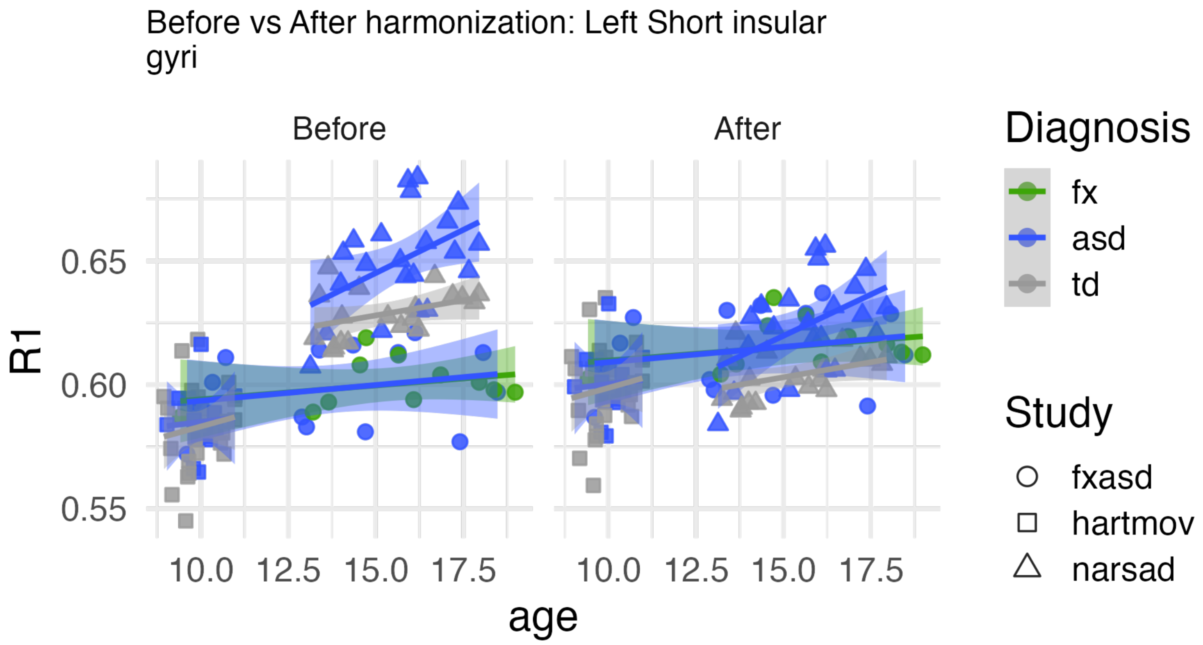
Before vs After harmonization: Left Short insular
gyri
Before
After
R1
0.65
0.60
0.55
10.0
12.5
15.0
17.5
age
Diagnosis
fx
asd
td
Study
fxasd
hartmov
narsad

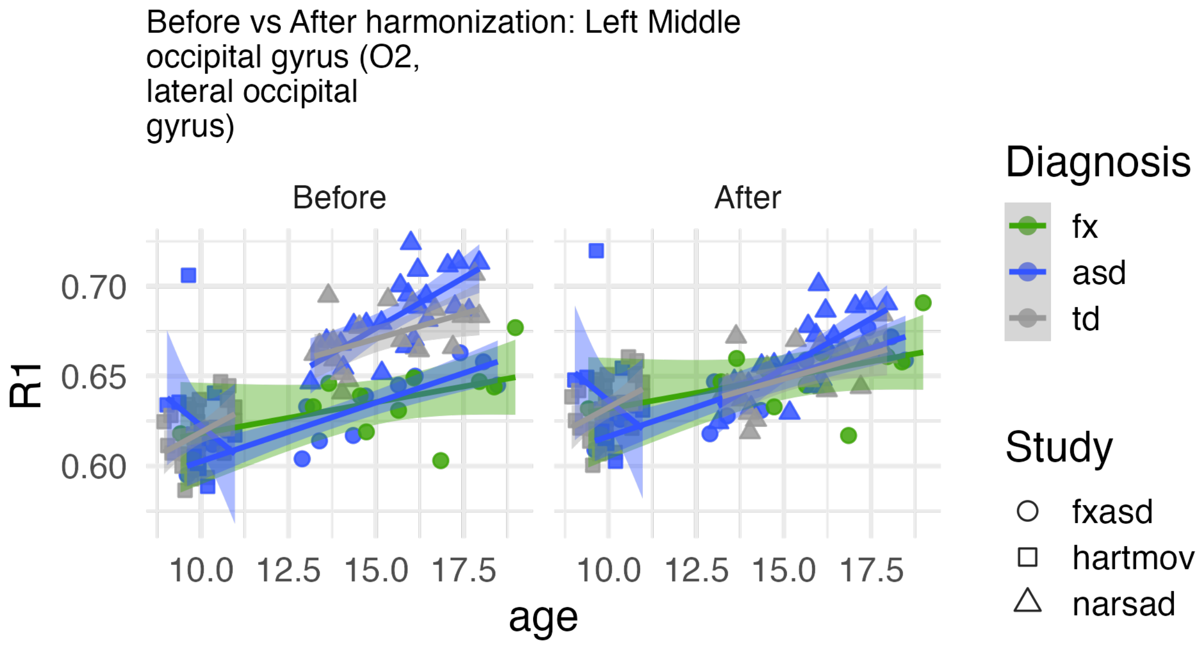
Before vs After harmonization: Left Middle occipital gyrus (O2, lateral occipital gyrus)
Before
After
R1
0.70
0.65
0.60
10.0
12.5
15.0
17.5
age
Diagnosis
fx
asd
td
Study
fxasd
hartmov
narsad

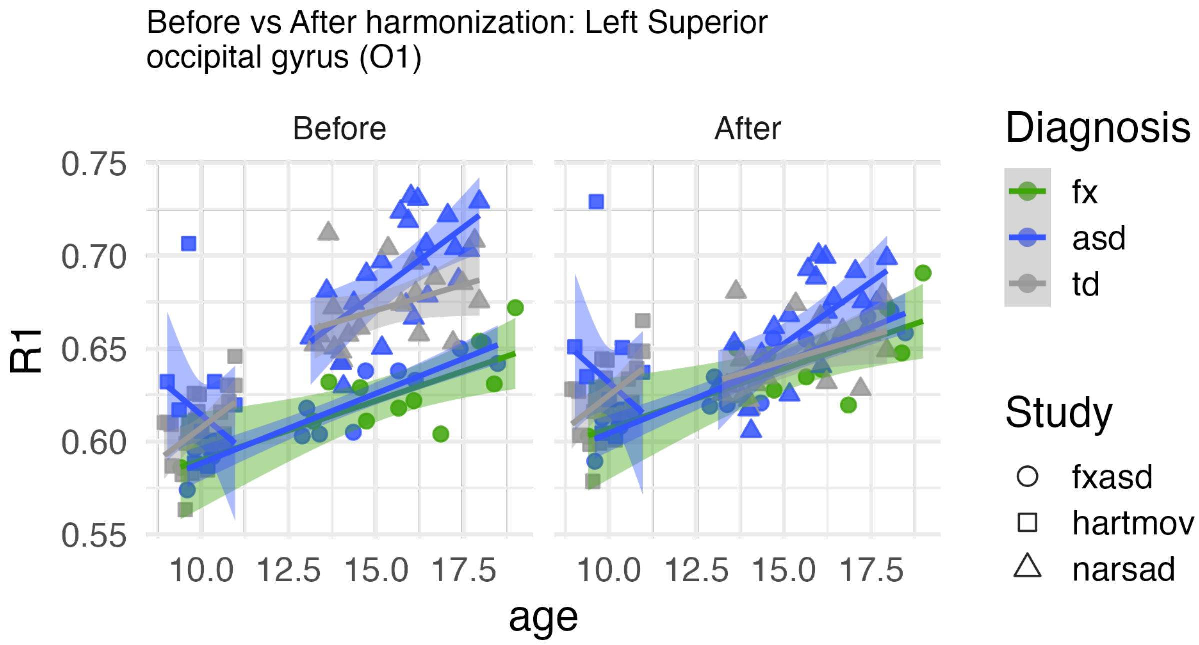
Before vs After harmonization: Left Superior occipital gyrus (O1)
Before
After
R1
0.75
0.70
0.65
0.60
0.55
10.0
12.5
15.0
17.5
age
Diagnosis
fx
asd
td
Study
fxasd
hartmov
narsad

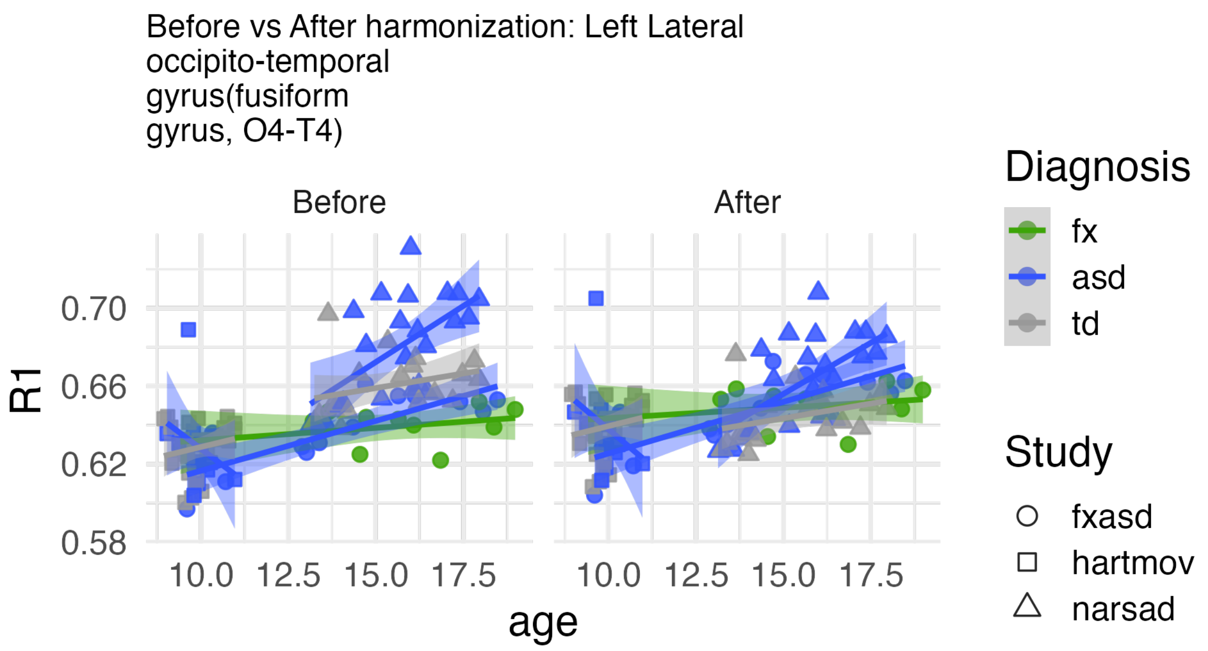

Before vs After harmonization: Left Lateral
occipito-temporal
gyrus(fusiform
gyrus, O4-T4)
Before
After
R1
0.70
0.66
0.62
0.58
10.0
12.5
15.0
17.5
age
Diagnosis
fx
asd
td
Study
fxasd
hartmov
narsad

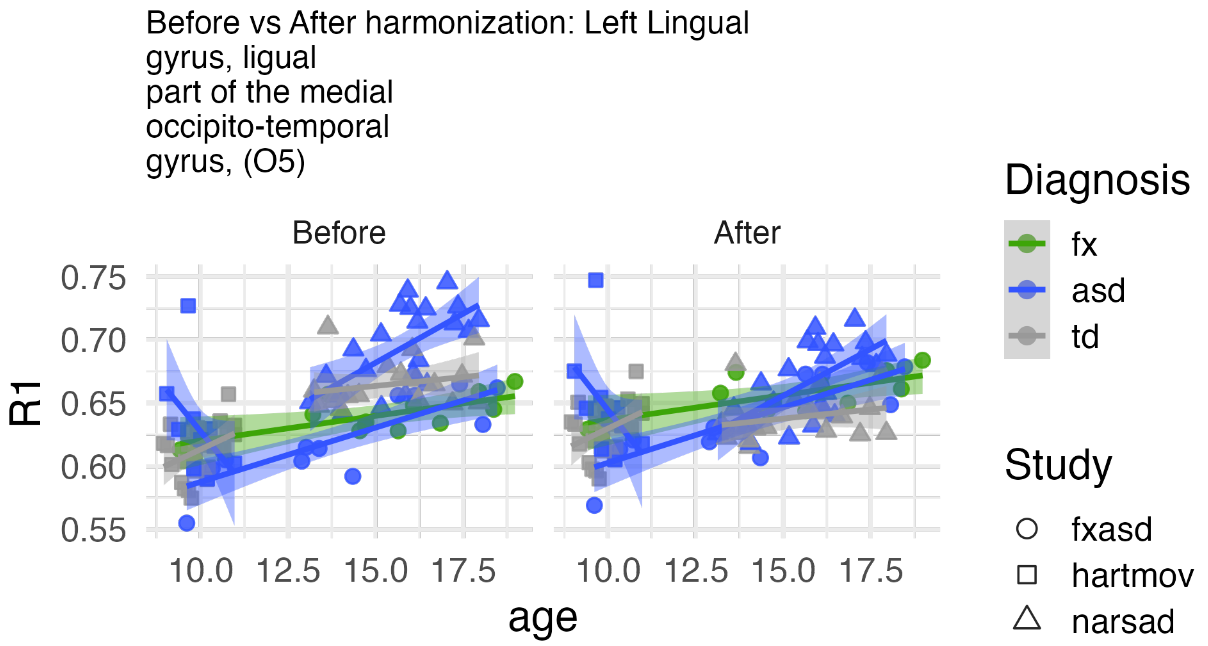

Before vs After harmonization: Left Lingual
gyrus, ligual
part of the medial
occipito-temporal
gyrus, (O5)
Before
After
R1
0.75
0.70
0.65
0.60
0.55
10.0
12.5
15.0
17.5
age
Diagnosis
fx
asd
td
Study
fxasd
hartmov
narsad

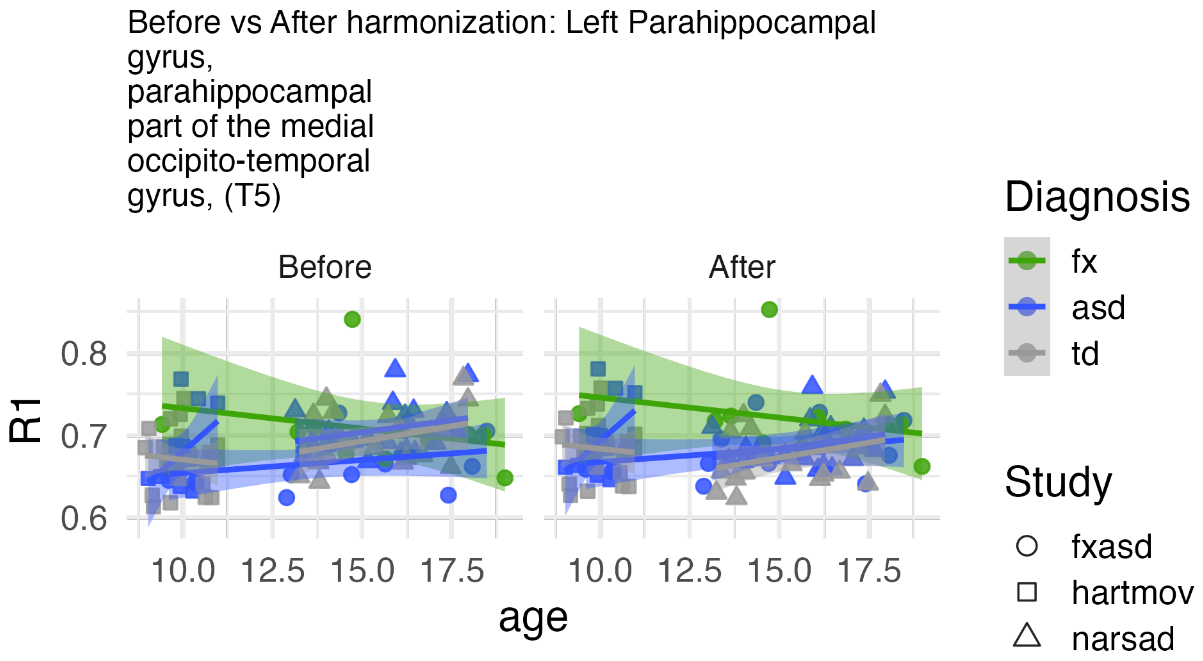
Before vs After harmonization: Left Parahippocampal gyrus,
parahippocampal part of the medial occipito-temporal gyrus, (T5)
Before
After
R1
0.6
0.7
0.8
10.0 12.5 15.0 17.5
age
Diagnosis
fx
asd
td
Study
fxasd
hartmov
narsad

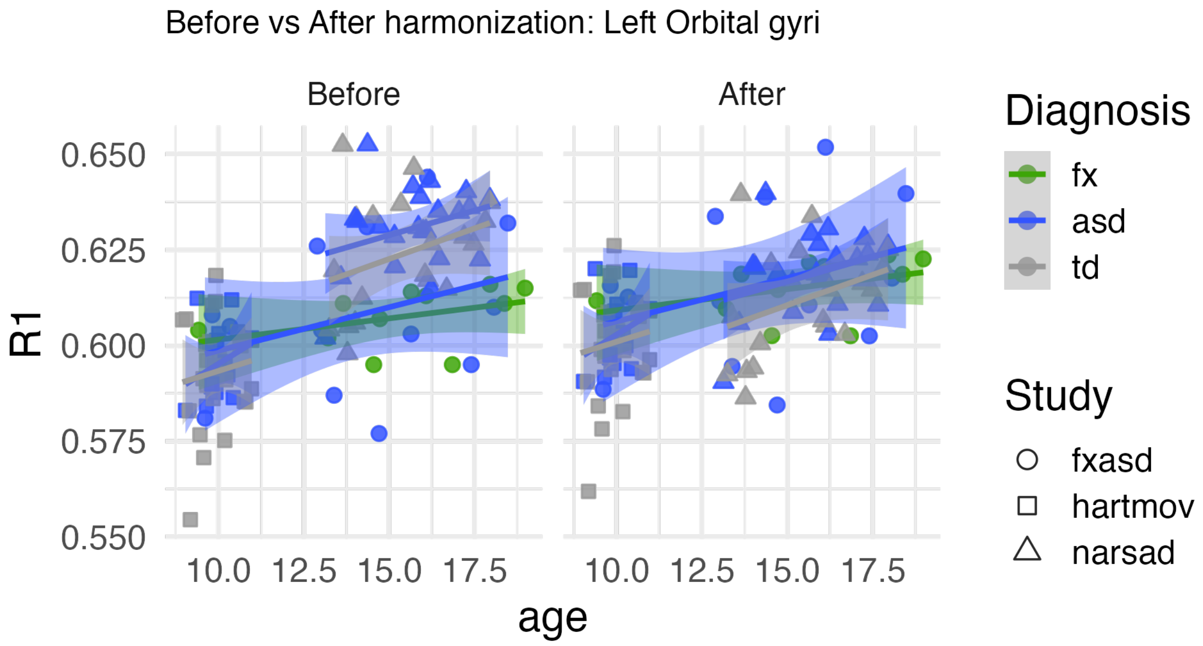
Before vs After harmonization: Left Orbital gyri
Before
After
R1
0.550
0.575
0.600
0.625
0.650
10.0 12.5 15.0 17.5
age
Diagnosis
fx
asd
td
Study
fxasd
hartmov
narsad

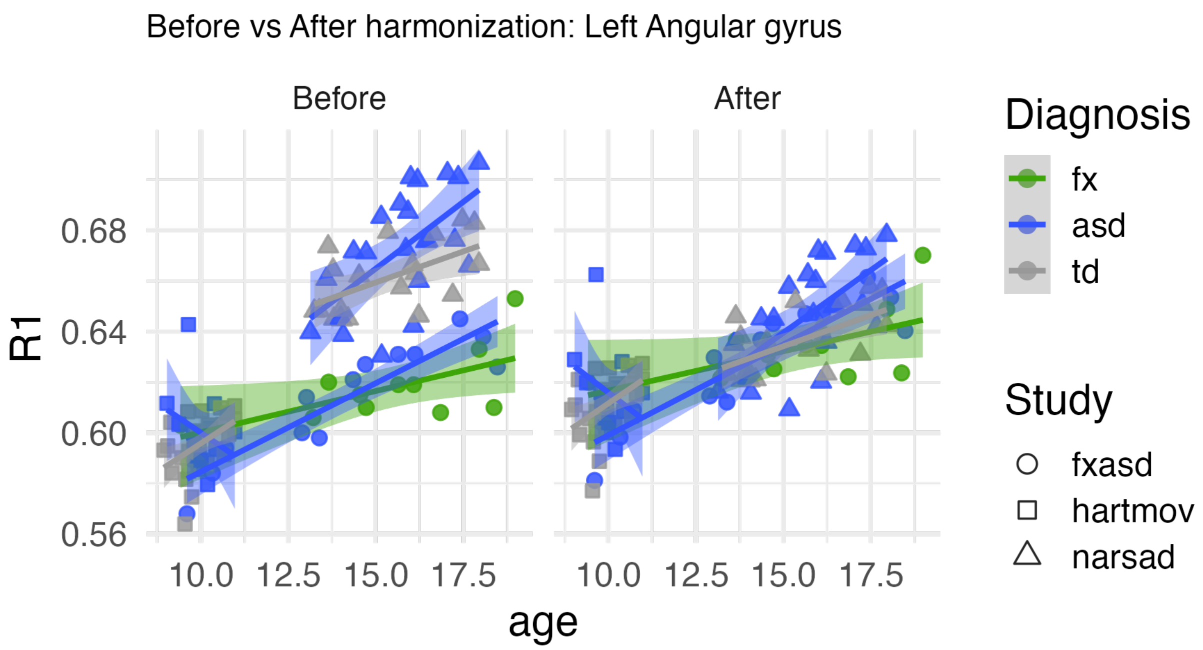
Before vs After harmonization: Left Angular gyrus
Before
After
R1
0.68
0.64
0.60
0.56
10.0
12.5
15.0
17.5
age
Diagnosis
fx
asd
td
Study
fxasd
hartmov
narsad

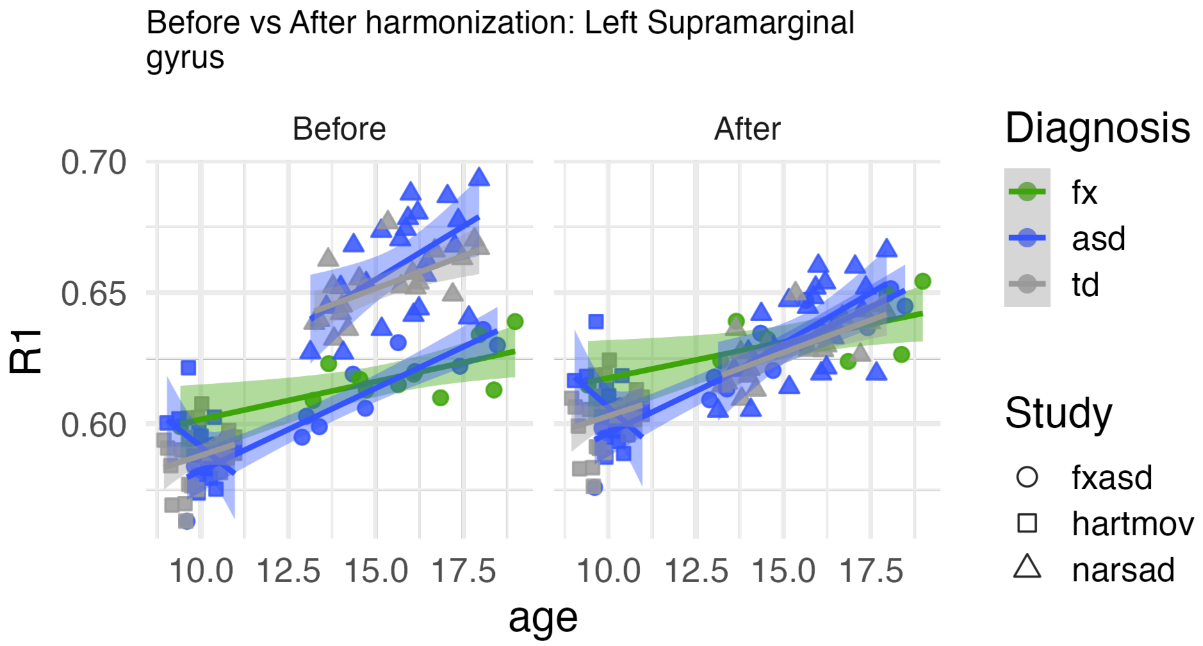
Before vs After harmonization: Left Supramarginal gyrus
Before
After
R1
0.70
0.65
0.60
10.0
12.5
15.0
17.5
age
Diagnosis
fx
asd
td
Study
fxasd
hartmov
narsad

Before vs After harmonization: Left Superior parietal lobule (lateral part of P1)

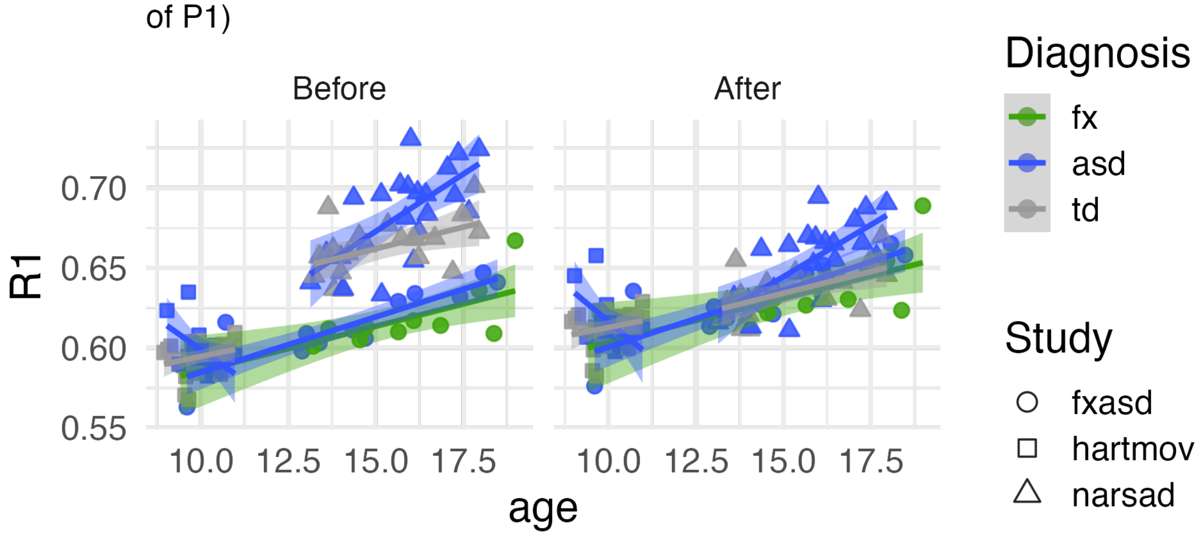

Before vs After harmonization: Left Postcentral gyrus

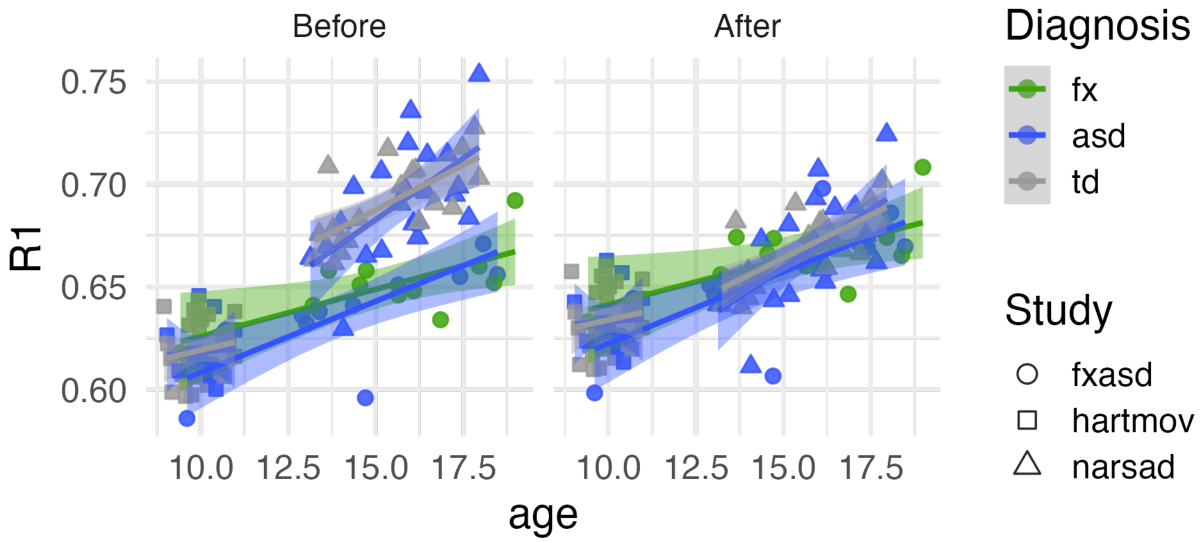

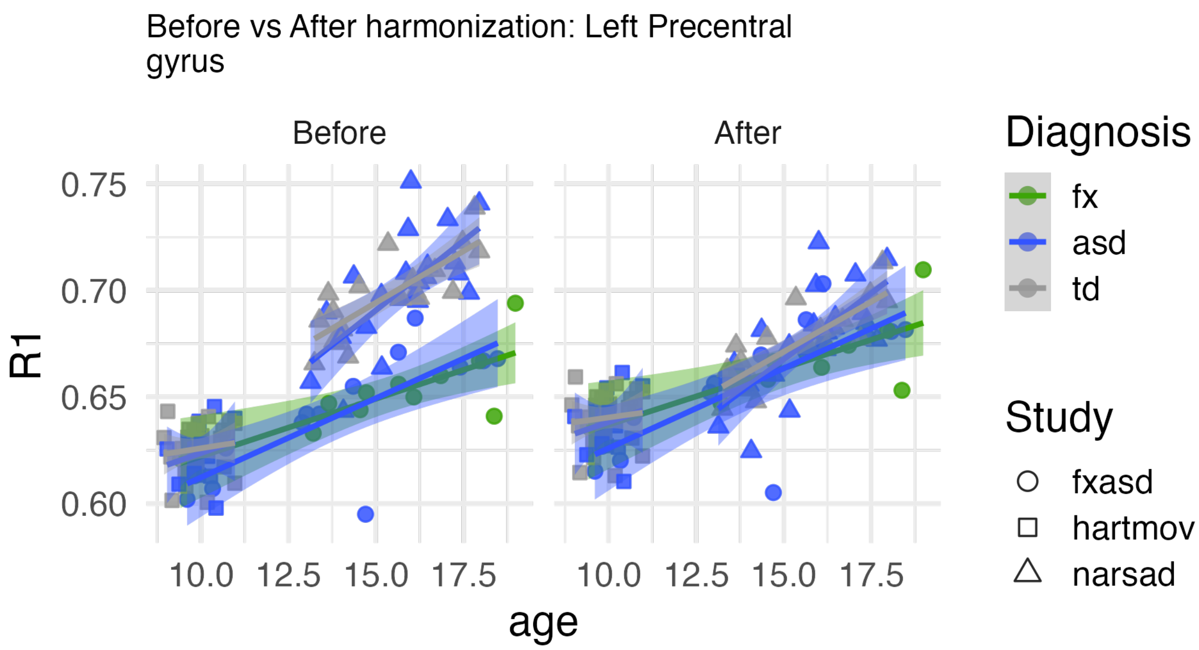
Before vs After harmonization: Left Precentral gyrus
Before
After
R1
0.75
0.70
0.65
0.60
10.0
12.5
15.0
17.5
age
Diagnosis
fx
asd
td
Study
fxasd
hartmov
narsad

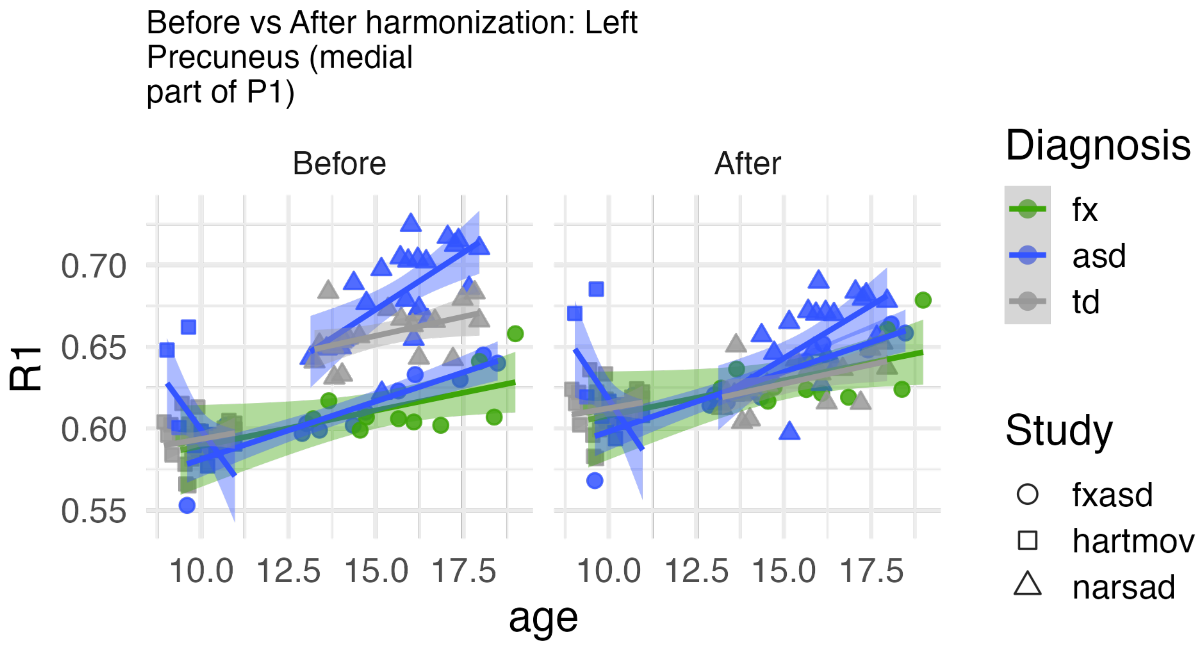
Before vs After harmonization: Left Precuneus (medial part of P1)
Before
After
R1
0.70
0.65
0.60
0.55
10.0
12.5
15.0
17.5
age
Diagnosis
fx
asd
td
Study
fxasd
hartmov
narsad

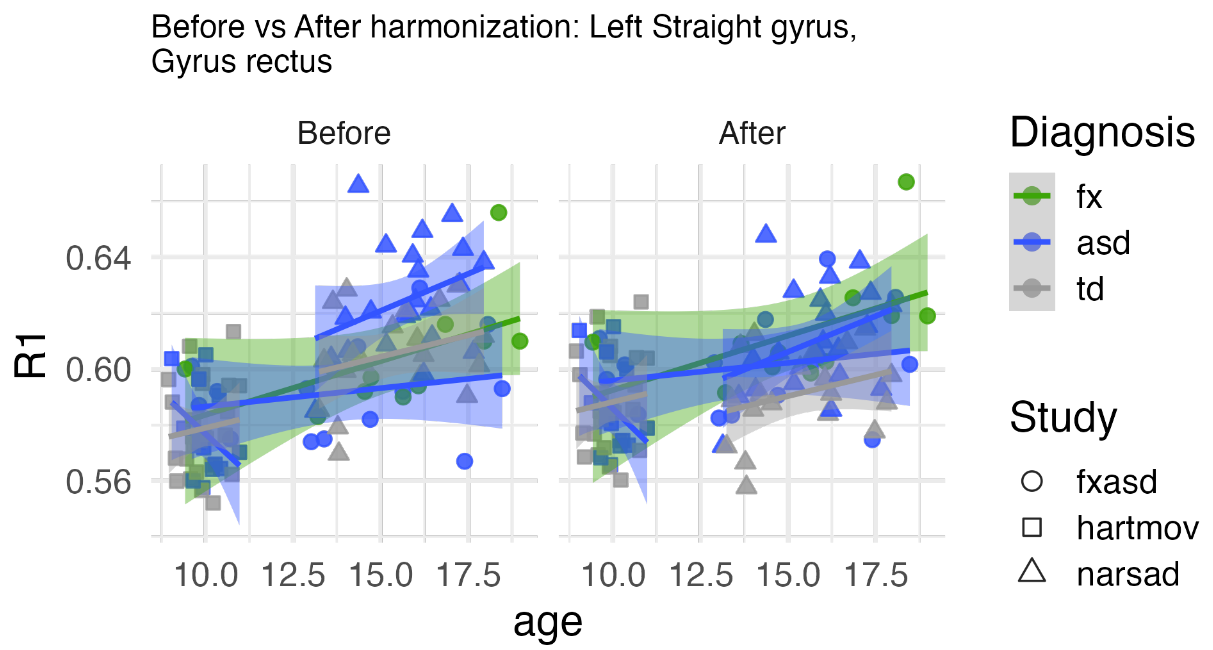

Before vs After harmonization: Left Straight gyrus, Gyrus rectus
Before
After
R1
0.64
0.60
0.56
10.0
12.5
15.0
17.5
age
Diagnosis
fx
asd
td
Study
fxasd
hartmov
narsad

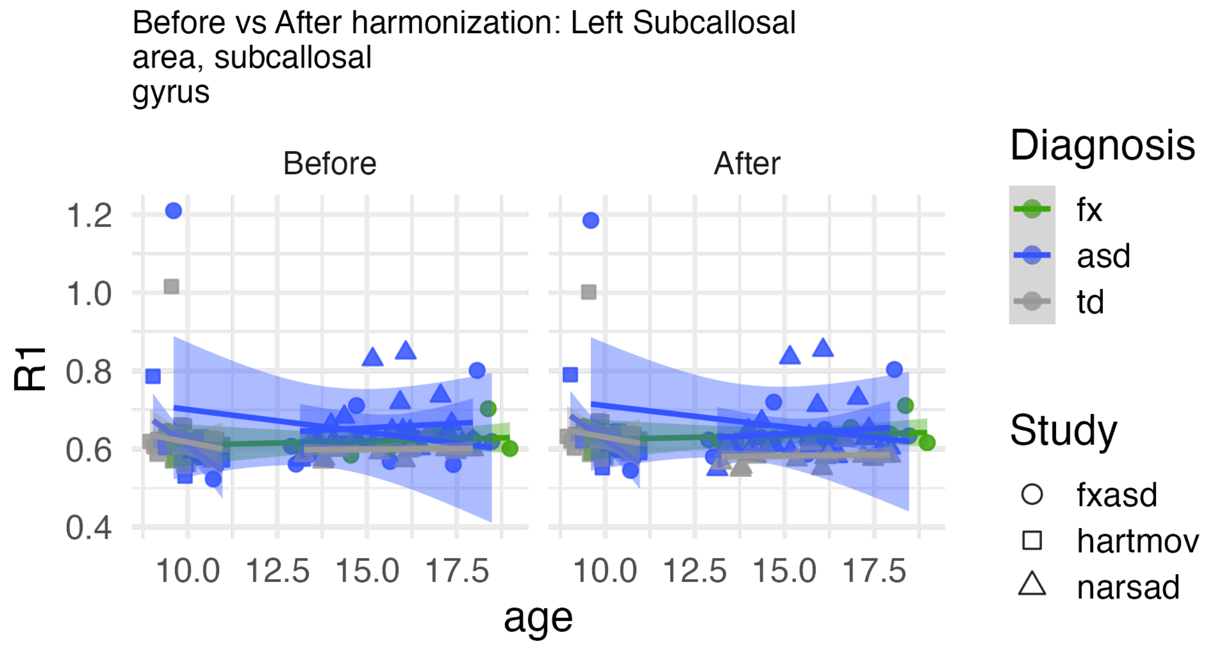

Before vs After harmonization: Left Subcallosal area, subcallosal gyrus
Before
After
R1
1.2
1.0
0.8
0.6
0.4
10.0
12.5
15.0
17.5
age
Diagnosis
fx
asd
td
Study
fxasd
hartmov
narsad

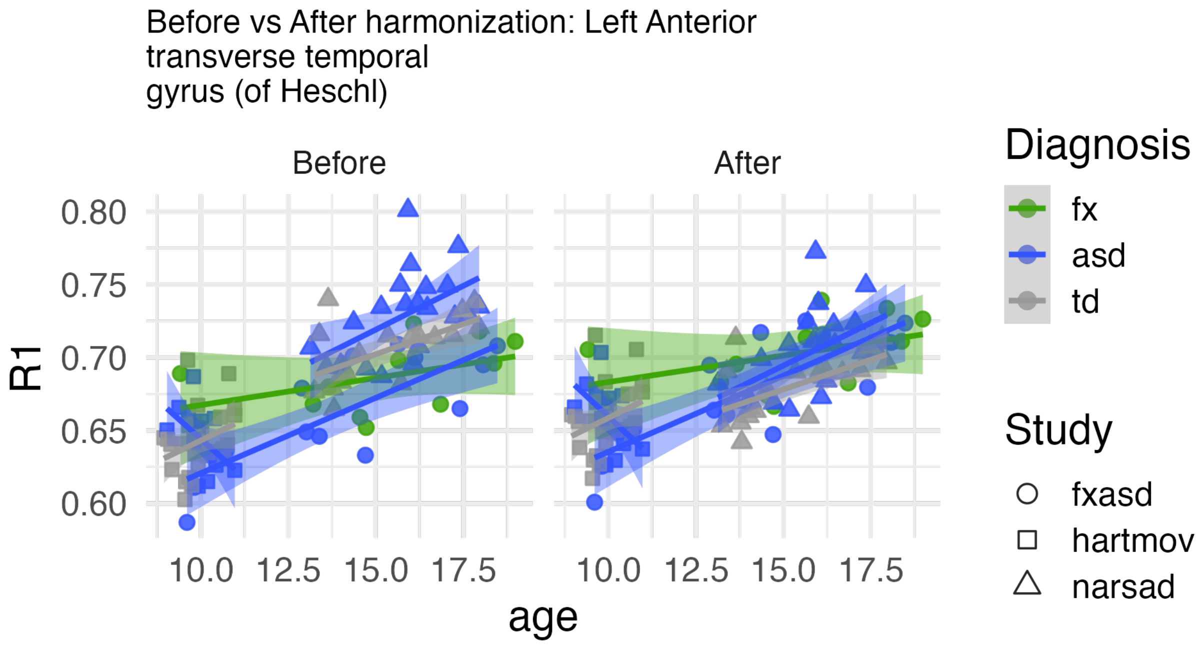
Before vs After harmonization: Left Anterior
transverse temporal
gyrus (of Heschl)
Before
After
0.80
0.75
0.70
0.65
0.60
R1
10.0
12.5
15.0
17.5
age
Diagnosis
fx
asd
td
Study
fxasd
hartmov
narsad

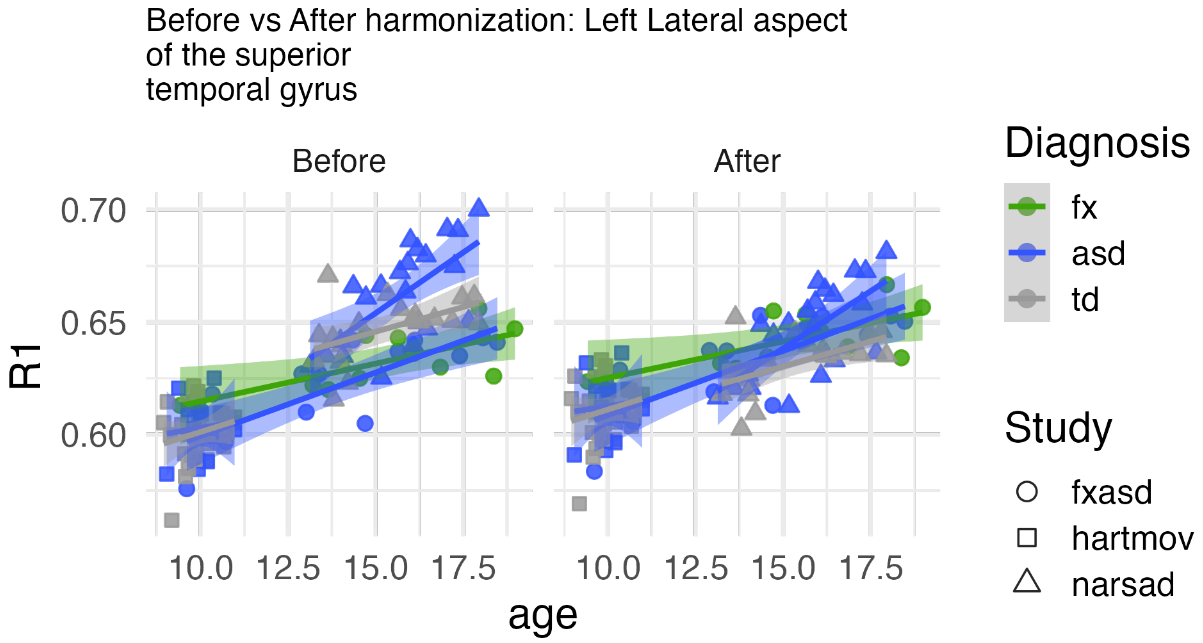
Before vs After harmonization: Left Lateral aspect
of the superior
temporal gyrus
Before
After
0.70
0.65
0.60
R1
10.0
12.5
15.0
17.5
age
Diagnosis
fx
asd
td
Study
fxasd
hartmov
narsad

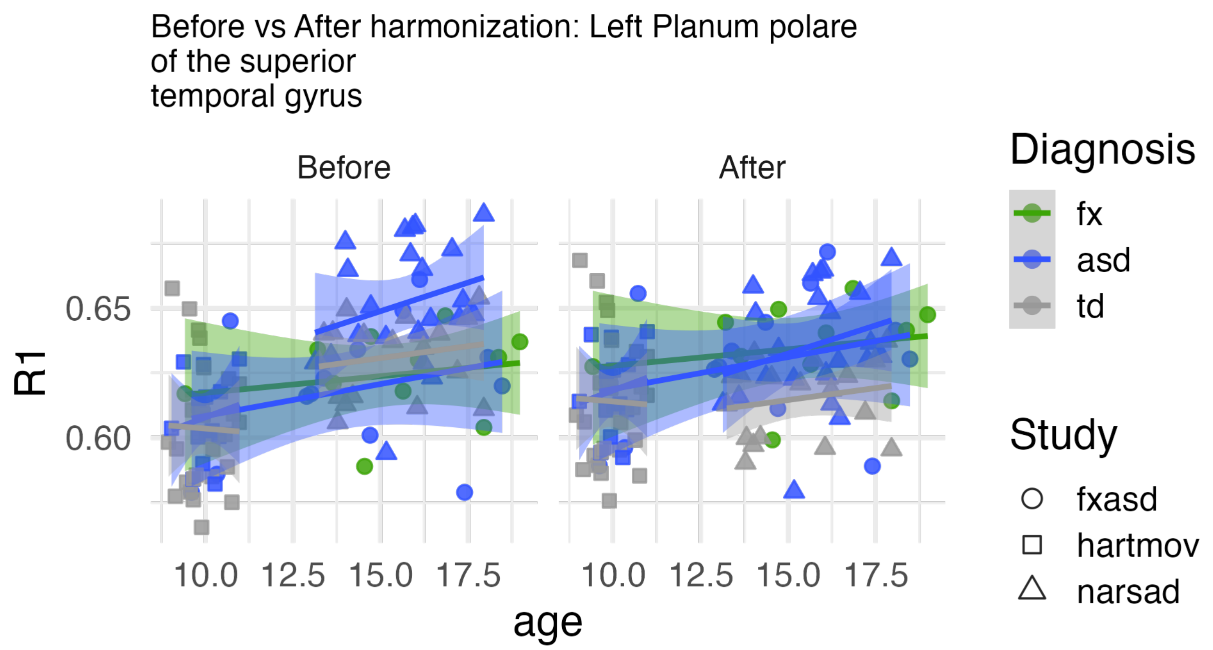

Before vs After harmonization: Left Planum polare
of the superior
temporal gyrus
Before
After
R1
0.65
0.60
10.0
12.5
15.0
17.5
age
Diagnosis
fx
asd
td
Study
fxasd
hartmov
narsad

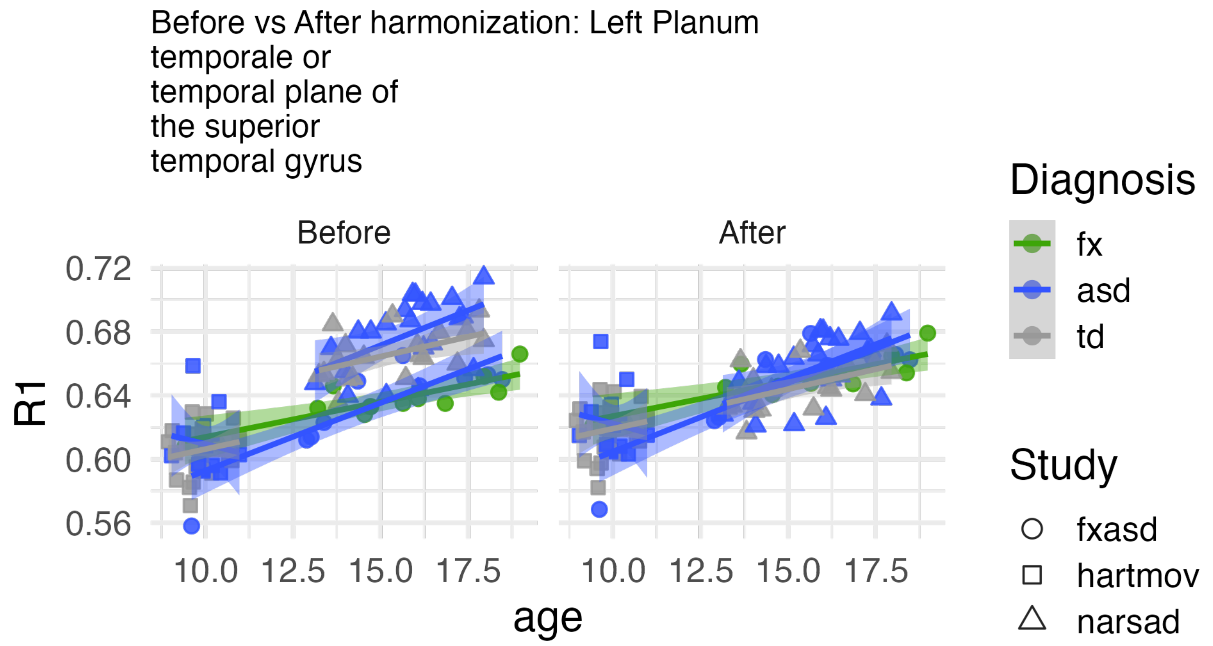

Before vs After harmonization: Left Planum
temporale or
temporal plane of
the superior
temporal gyrus
Before
After
R1
0.72
0.68
0.64
0.60
0.56
10.0
12.5
15.0
17.5
age
Diagnosis
fx
asd
td
Study
fxasd
hartmov
narsad

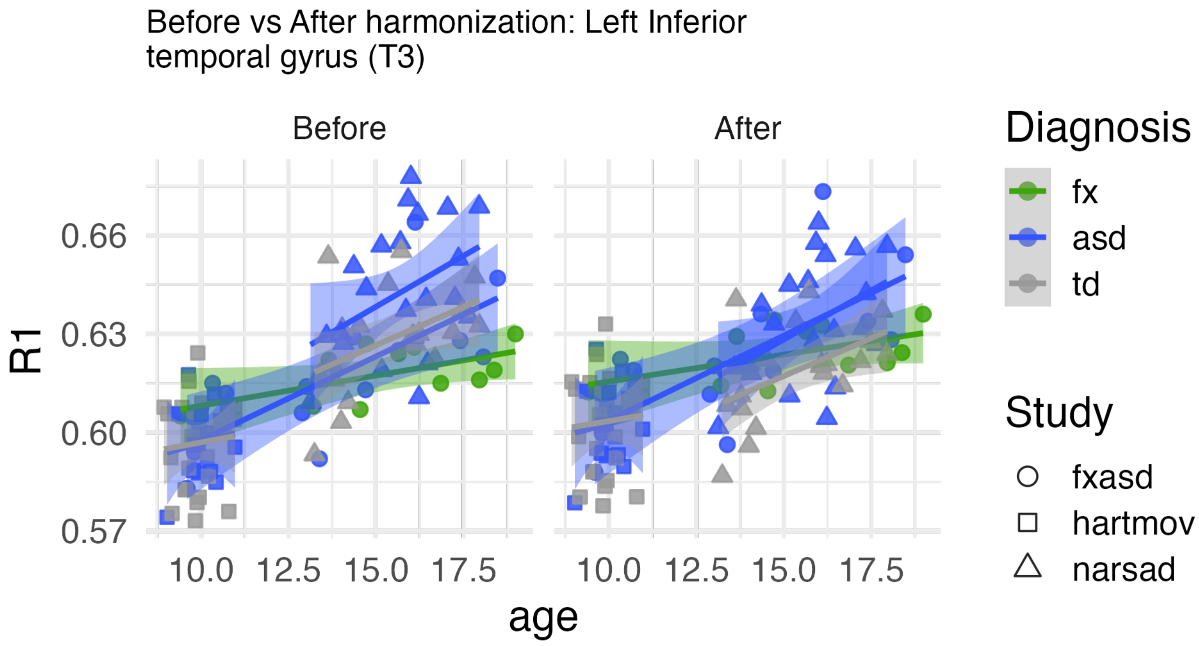
Before vs After harmonization: Left Inferior temporal gyrus (T3)
Before
After
R1
0.66
0.63
0.60
0.57
10.0
12.5
15.0
17.5
age
Diagnosis
fx
asd
td
Study
fxasd
hartmov
narsad

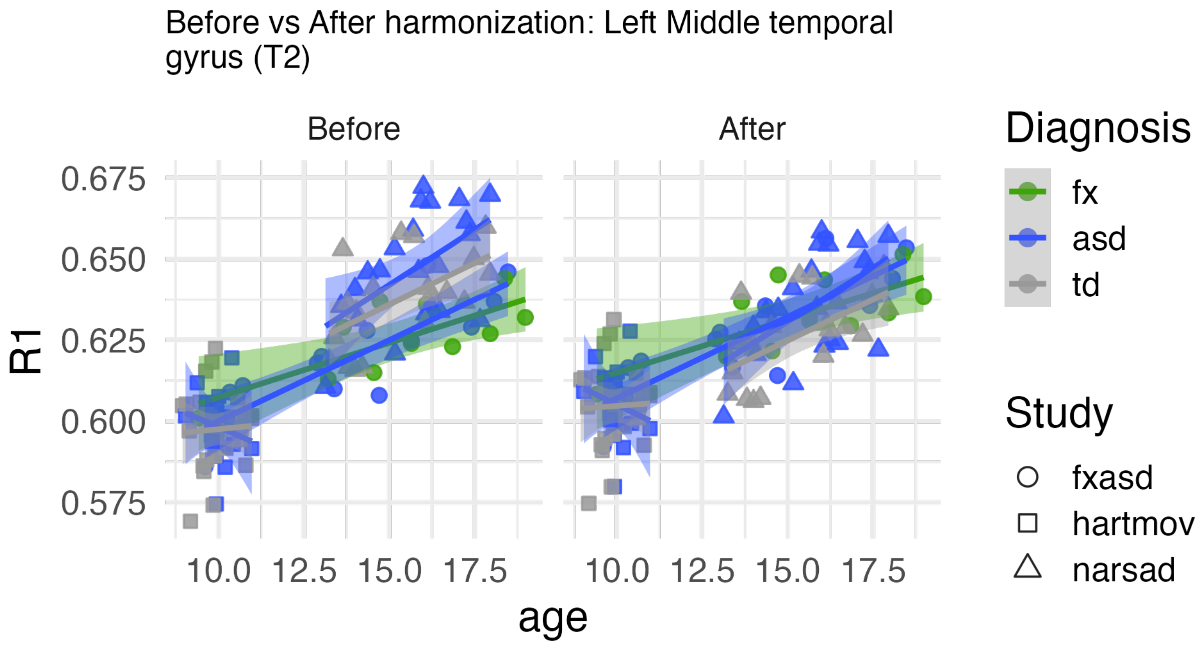
Before vs After harmonization: Left Middle temporal gyrus (T2)
Before
After
R1
0.675
0.650
0.625
0.600
0.575
10.0
12.5
15.0
17.5
age
Diagnosis
fx
asd
td
Study
fxasd
hartmov
narsad

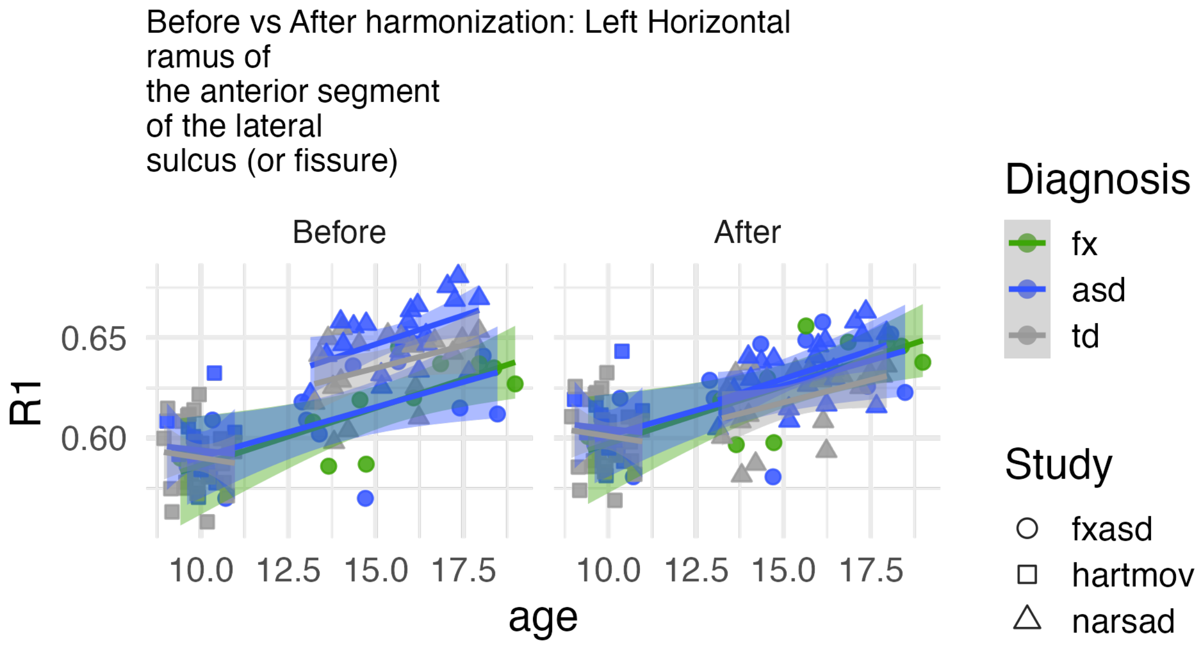
Before vs After harmonization: Left Horizontal
ramus of
the anterior segment
of the lateral
sulcus (or fissure)
Before
After
R1
0.65
0.60
10.0
12.5
15.0
17.5
age
Diagnosis
fx
asd
td
Study
fxasd
hartmov
narsad

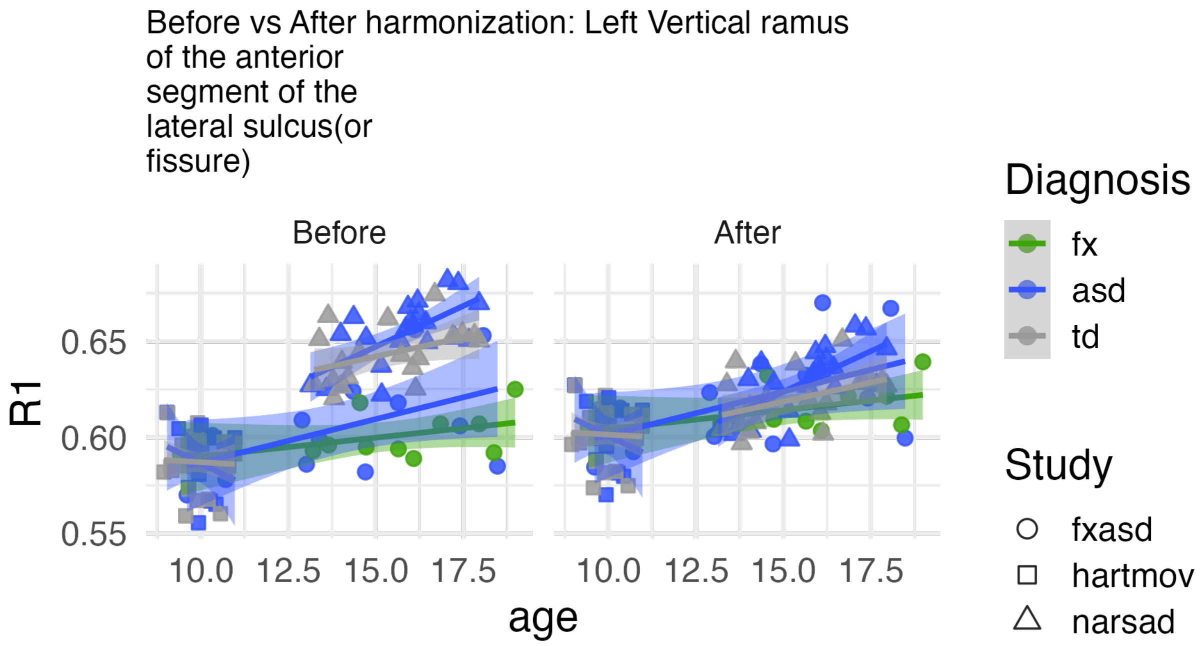
Before vs After harmonization: Left Vertical ramus
of the anterior
segment of the
lateral sulcus(or
fissure)
Before
After
R1
0.65
0.60
0.55
10.0
12.5
15.0
17.5
age
Diagnosis
fx
asd
td
Study
fxasd
hartmov
narsad

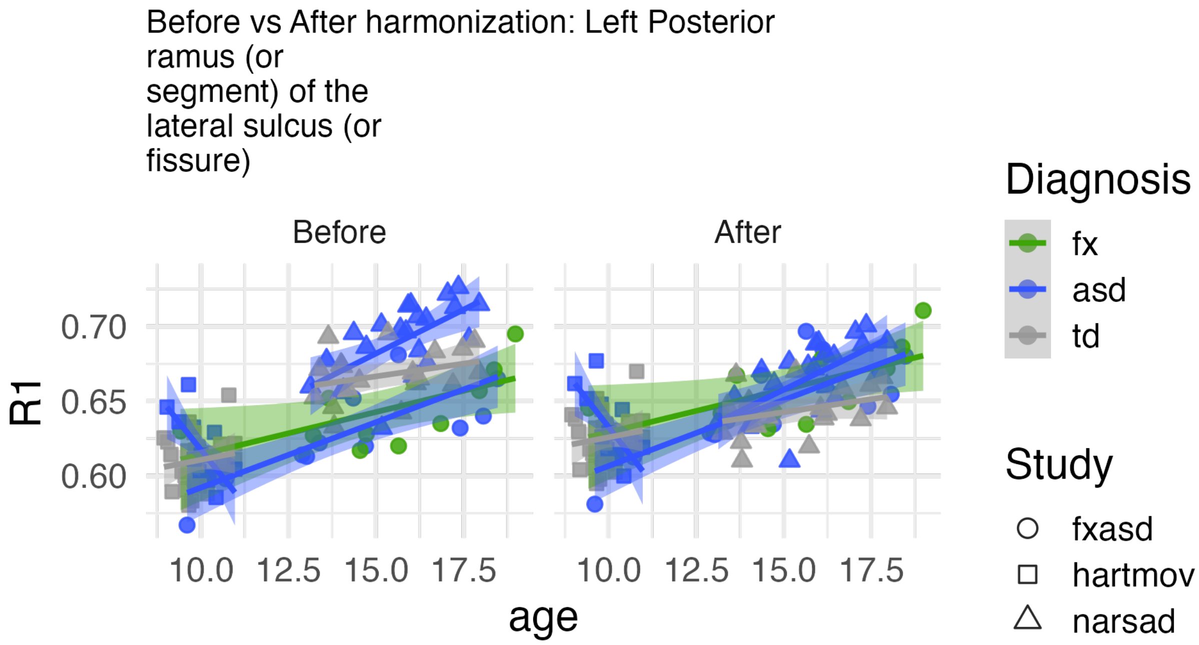
Before vs After harmonization: Left Posterior ramus (or segment) of the lateral sulcus (or fissure)
Before
After
R1
0.70
0.65
0.60
10.0
12.5
15.0
17.5
age
Diagnosis
fx
asd
td
Study
fxasd
hartmov
narsad

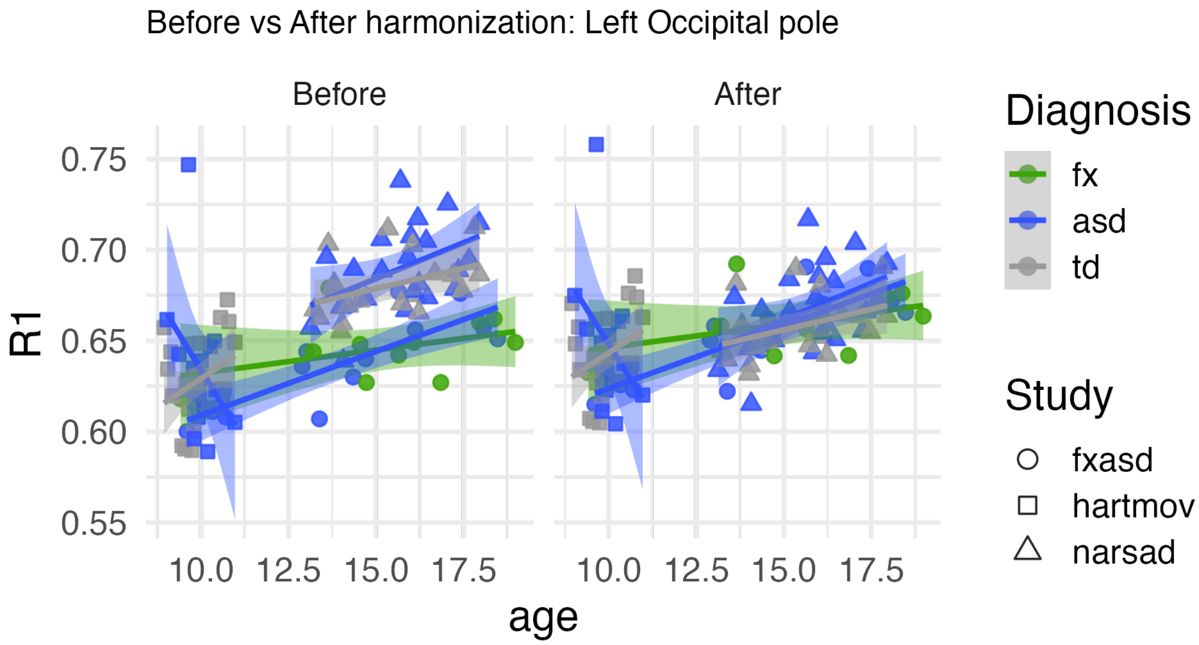
Before vs After harmonization: Left Occipital pole
Before
After
R1
0.75
0.70
0.65
0.60
0.55
10.0
12.5
15.0
17.5
age
Diagnosis
fx
asd
td
Study
fxasd
hartmov
narsad

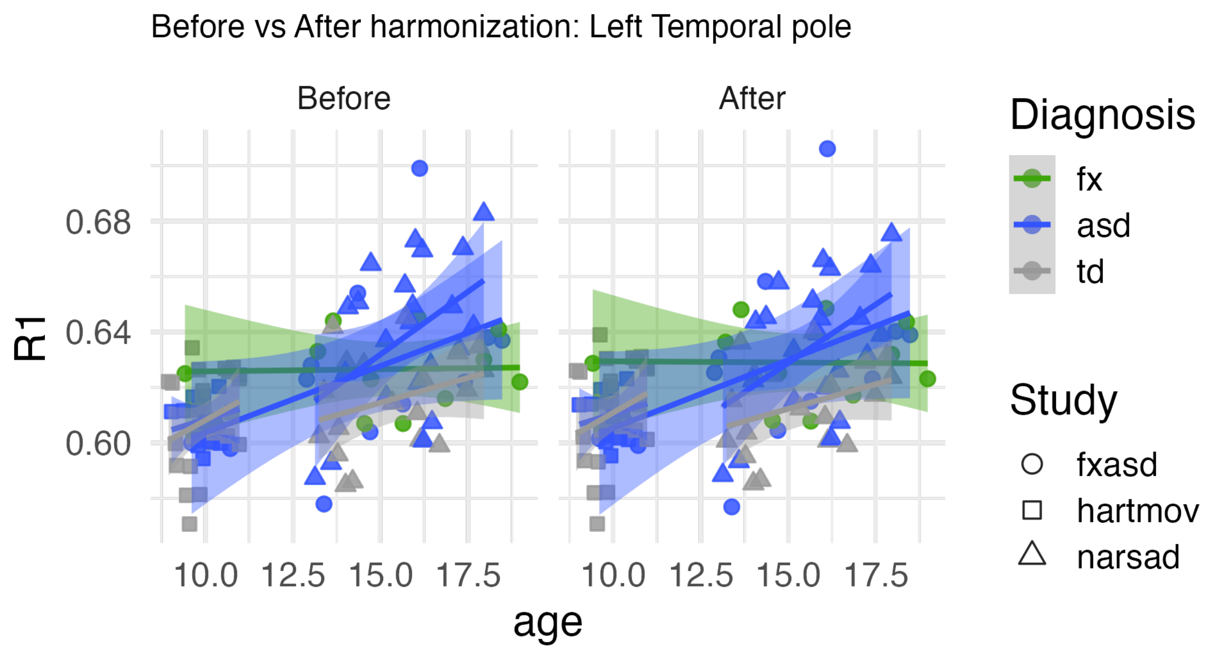
Before vs After harmonization: Left Temporal pole
Before
After
0.68
0.64
0.60
R1
10.0
12.5
15.0
17.5
age
Diagnosis
fx
asd
td
Study
fxasd
hartmov
narsad

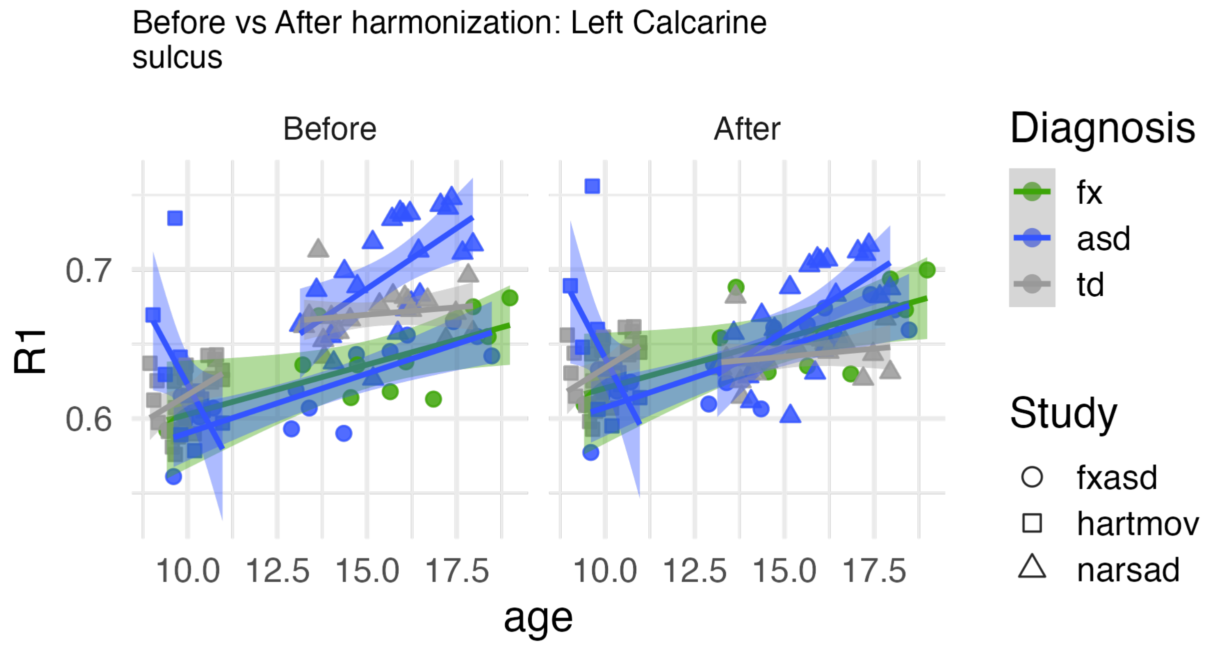
Before vs After harmonization: Left Calcarine sulcus
Before
After
0.7
0.6
R1
10.0
12.5
15.0
17.5
age
Diagnosis
fx
asd
td
Study
fxasd
hartmov
narsad

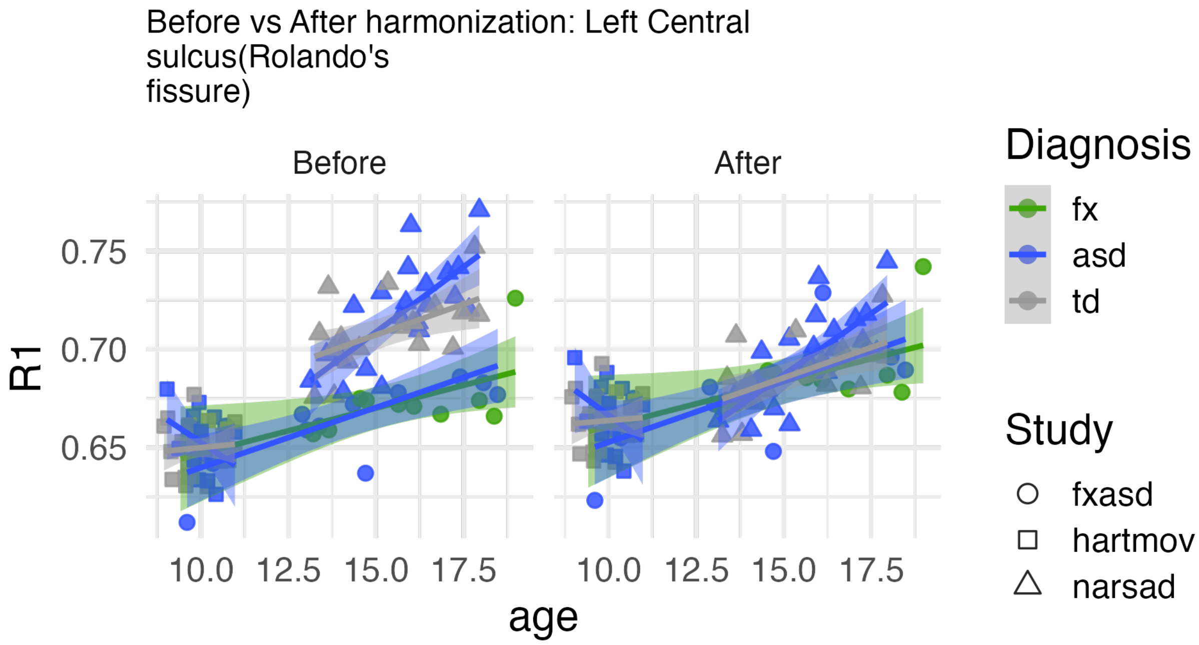
Before vs After harmonization: Left Central
sulcus(Rolando's
fissure)
Before
After
R1
0.75
0.70
0.65
10.0
12.5
15.0
17.5
age
Diagnosis
fx
asd
td
Study
fxasd
hartmov
narsad

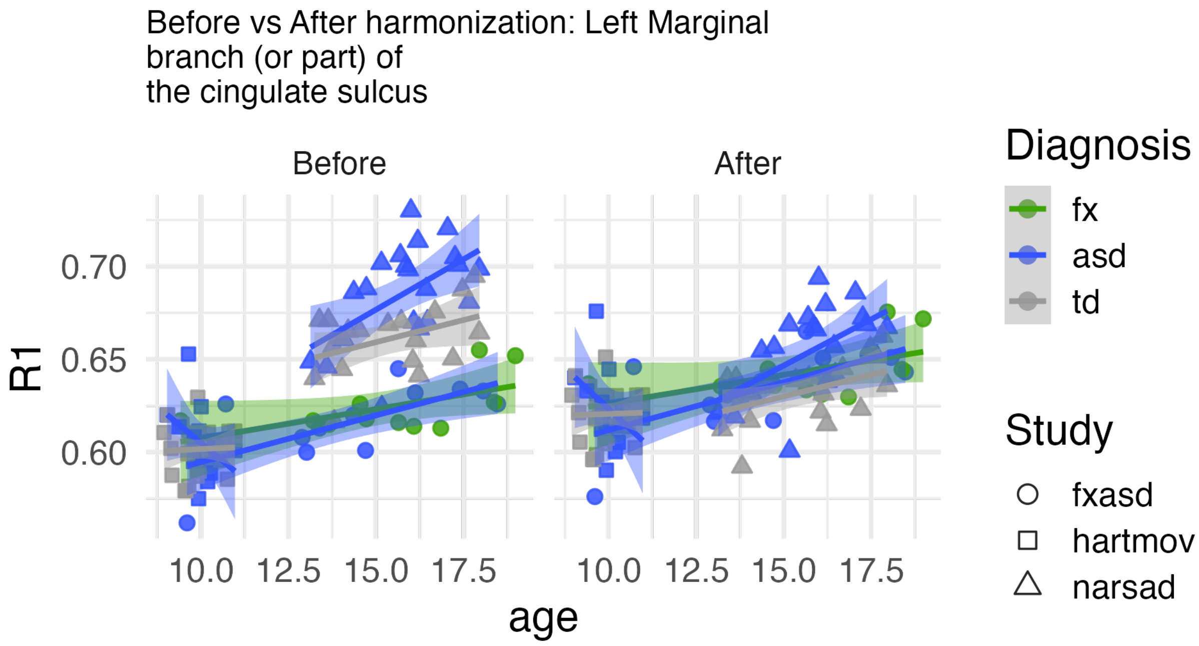
Before vs After harmonization: Left Marginal
branch (or part) of
the cingulate sulcus
Before
After
R1
0.70
0.65
0.60
10.0
12.5
15.0
17.5
age
Diagnosis
fx
asd
td
Study
fxasd
hartmov
narsad

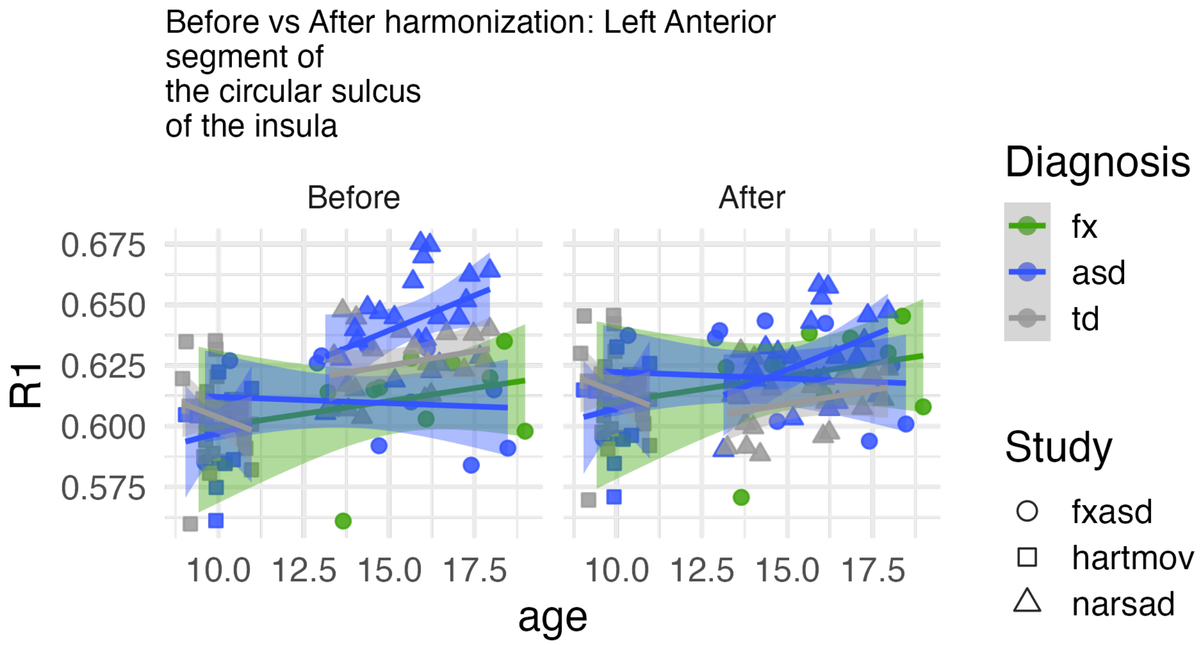

Before vs After harmonization: Left Anterior
segment of
the circular sulcus
of the insula
Before
After
R1
0.675
0.650
0.625
0.600
0.575
10.0
12.5
15.0
17.5
age
Diagnosis
fx
asd
td
Study
fxasd
hartmov
narsad

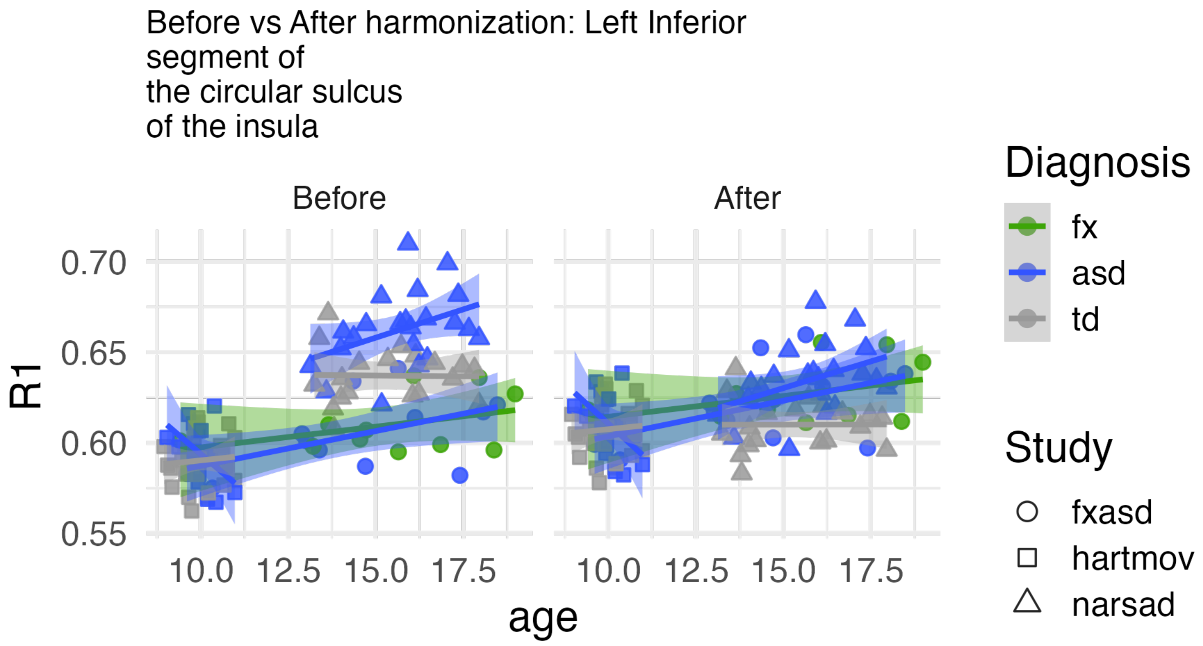

Before vs After harmonization: Left Inferior
segment of
the circular sulcus
of the insula
Before
After
R1
0.70
0.65
0.60
0.55
10.0
12.5
15.0
17.5
age
Diagnosis
fx
asd
td
Study
fxasd
hartmov
narsad

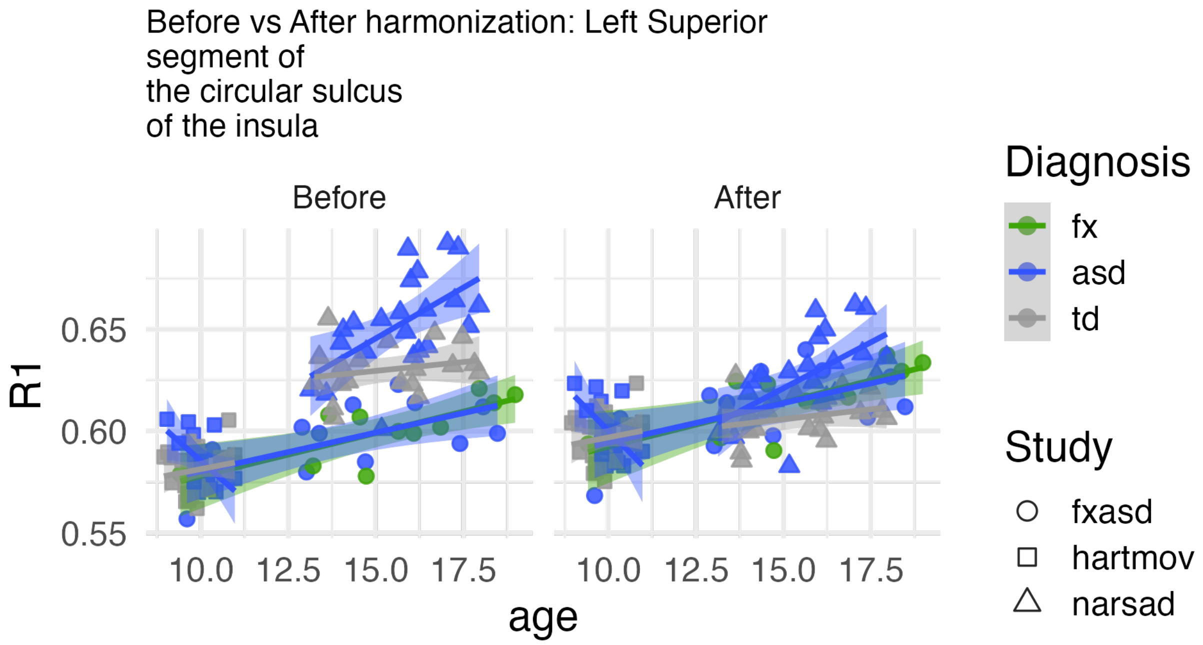

Before vs After harmonization: Left Superior
segment of
the circular sulcus
of the insula
Before
After
R1
0.65
0.60
0.55
10.0
12.5
15.0
17.5
age
Diagnosis
fx
asd
td
Study
fxasd
hartmov
narsad

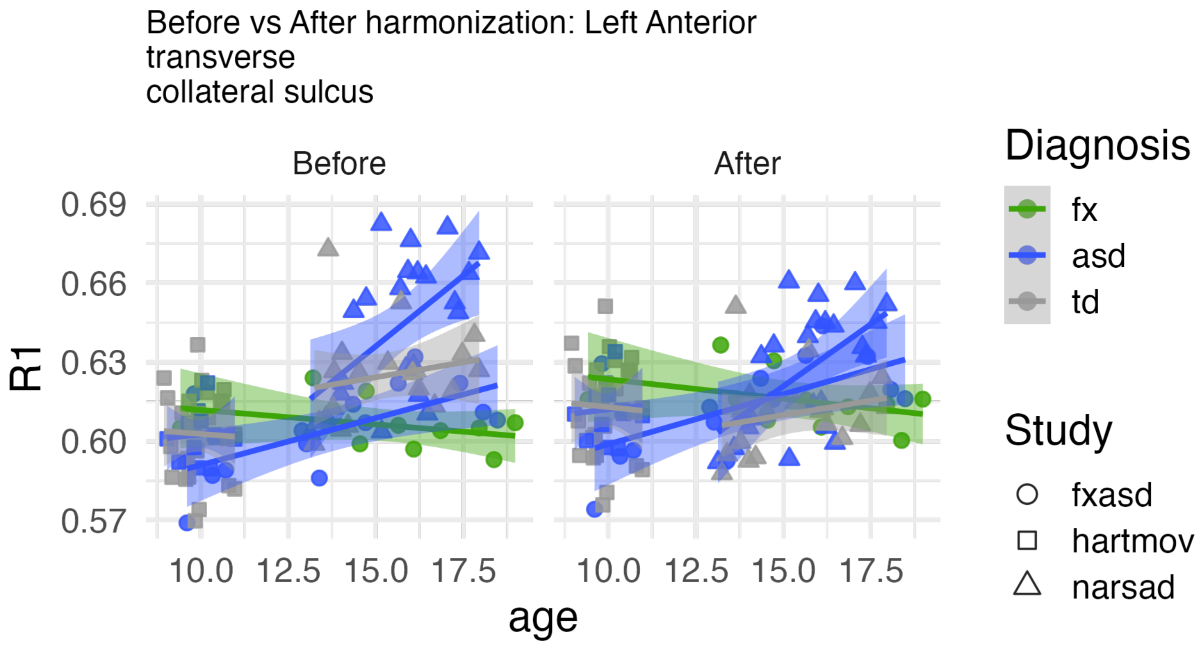

Before vs After harmonization: Left Anterior
transverse
collateral sulcus
Before
After
R1
0.69
0.66
0.63
0.60
0.57
10.0
12.5
15.0
17.5
age
Diagnosis
fx
asd
td
Study
fxasd
hartmov
narsad

Before vs After harmonization: Left Posterior
transverse
collateral sulcus

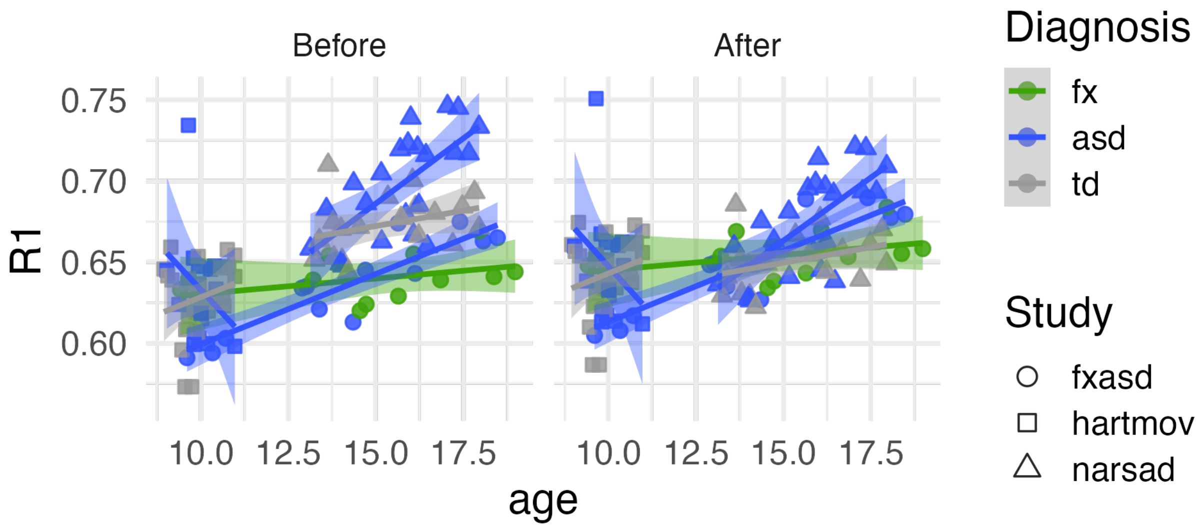

Before vs After harmonization: Left Inferior
frontal sulcus

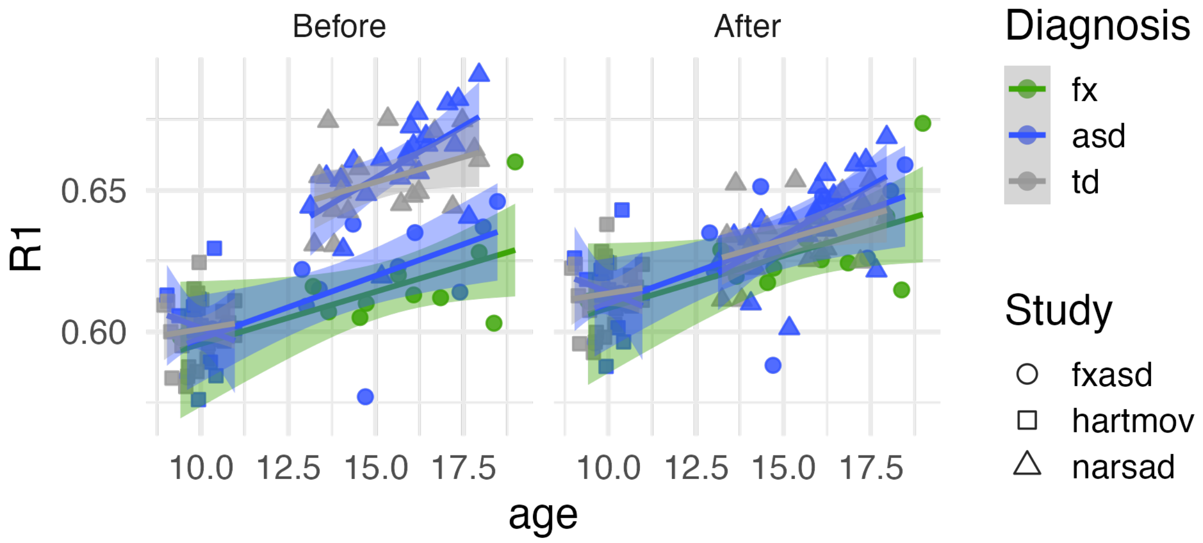

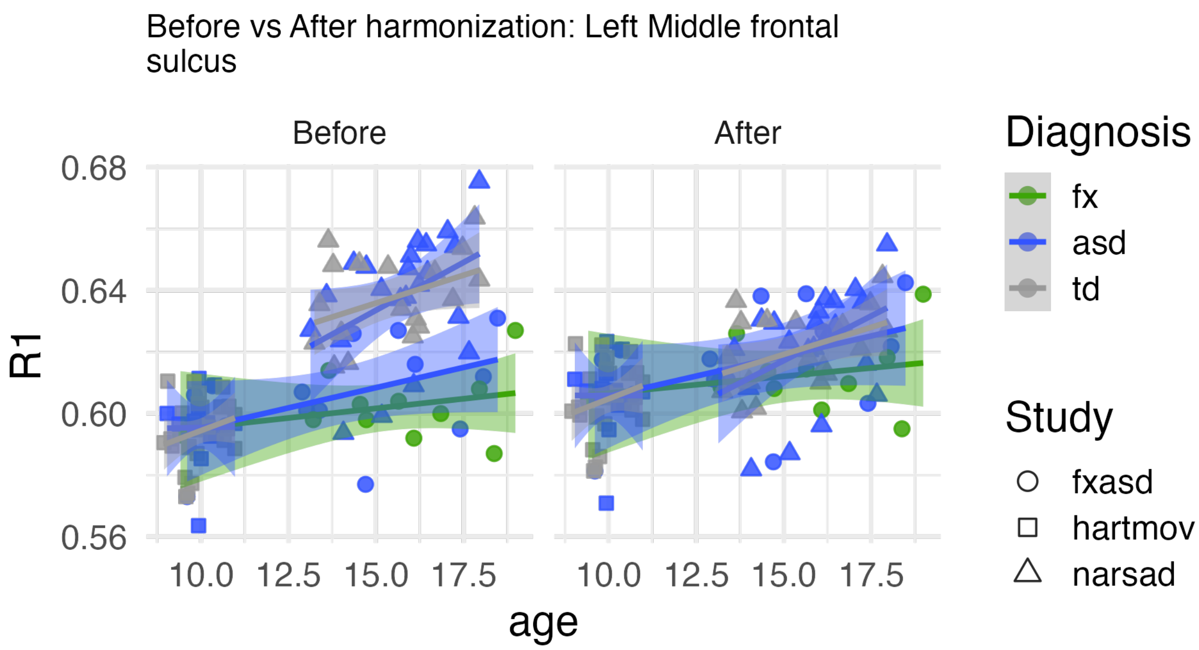
Before vs After harmonization: Left Middle frontal sulcus
Before
After
0.68
0.64
0.60
0.56
R1
10.0 12.5 15.0 17.5
10.0 12.5 15.0 17.5
age
Diagnosis
fx
asd
td
Study
fxasd
hartmov
narsad

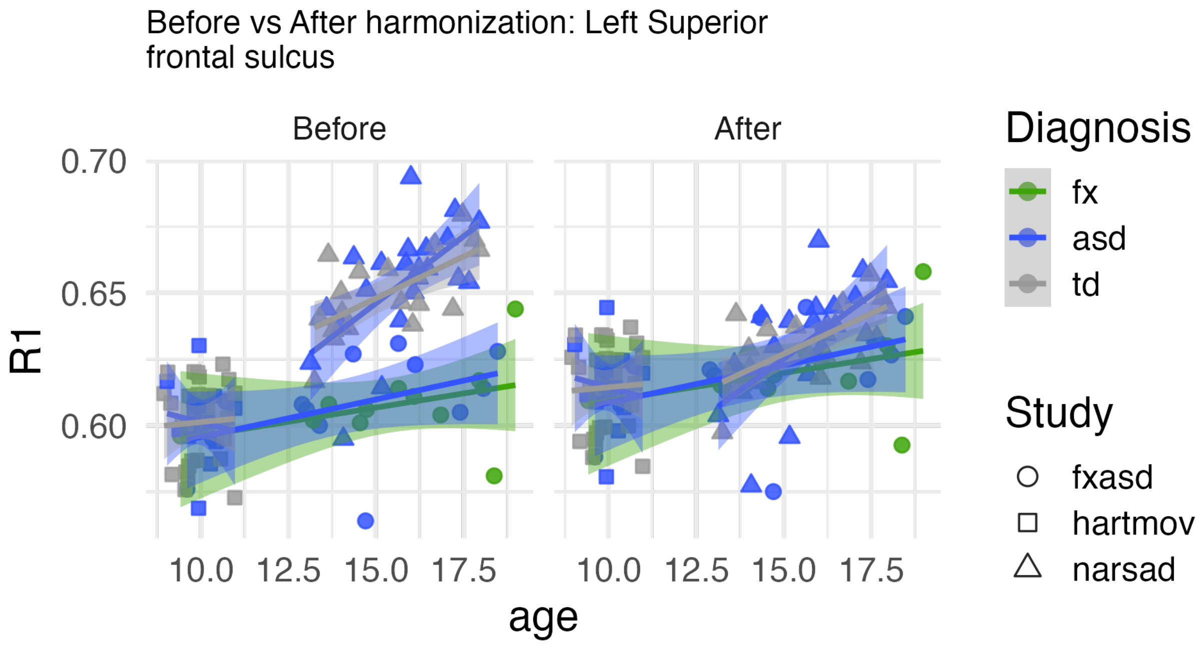
Before vs After harmonization: Left Superior frontal sulcus
Before
After
0.70
0.65
0.60
R1
10.0 12.5 15.0 17.5
10.0 12.5 15.0 17.5
age
Diagnosis
fx
asd
td
Study
fxasd
hartmov
narsad

Before vs After harmonization: Left Sulcus
intermedius primus
(of Jensen)

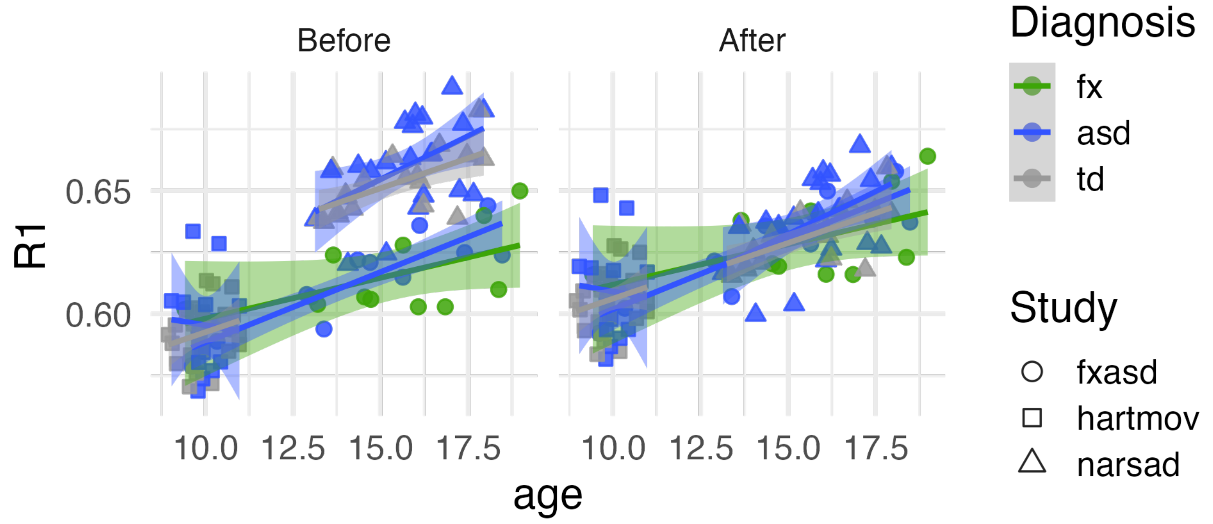

Before vs After harmonization: Left Intraparietal
sulcus(interparietal
sulcus) and
transverse parietal
sulci

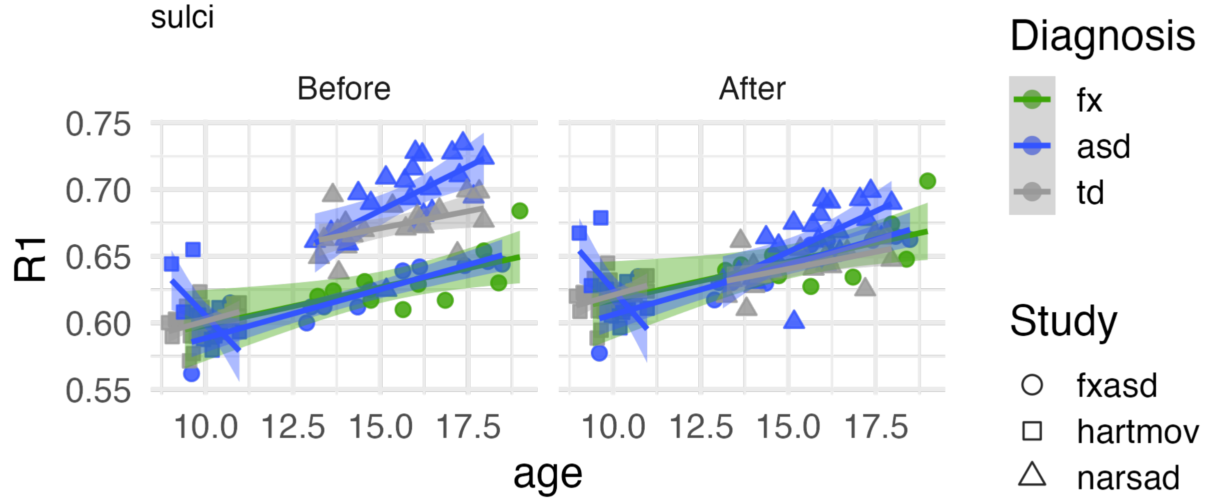

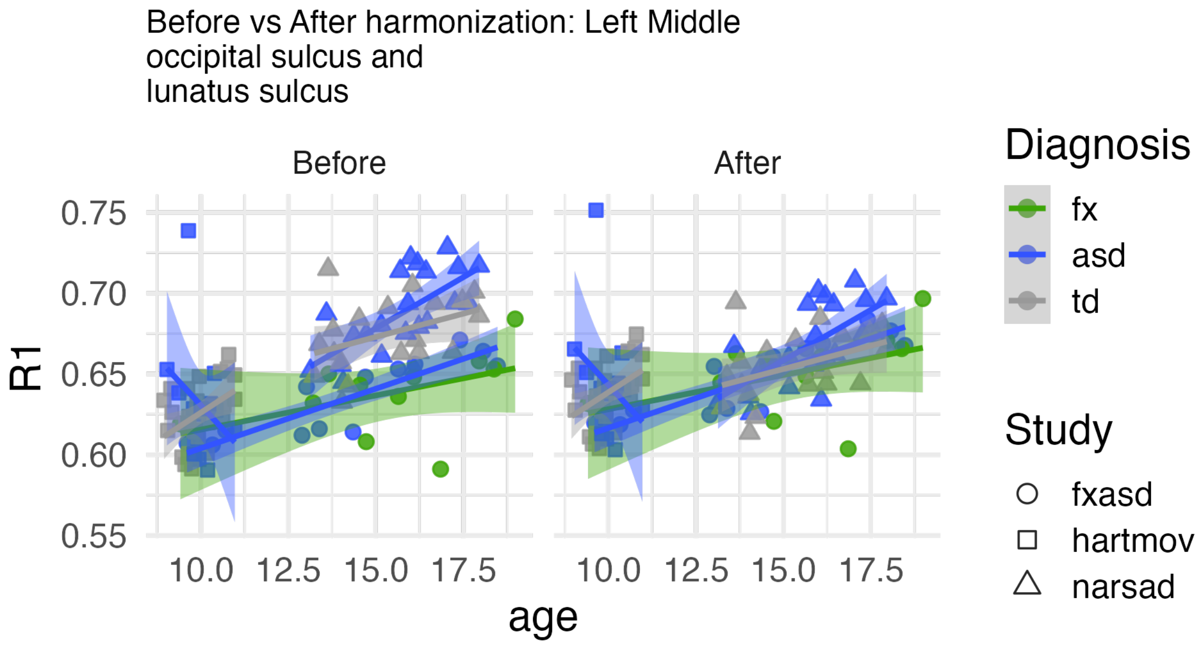

Before vs After harmonization: Left Middle occipital sulcus and lunatus sulcus
Before
After
R1
0.75
0.70
0.65
0.60
0.55
10.0
12.5
15.0
17.5
age
Diagnosis
fx
asd
td
Study
fxasd
hartmov
narsad

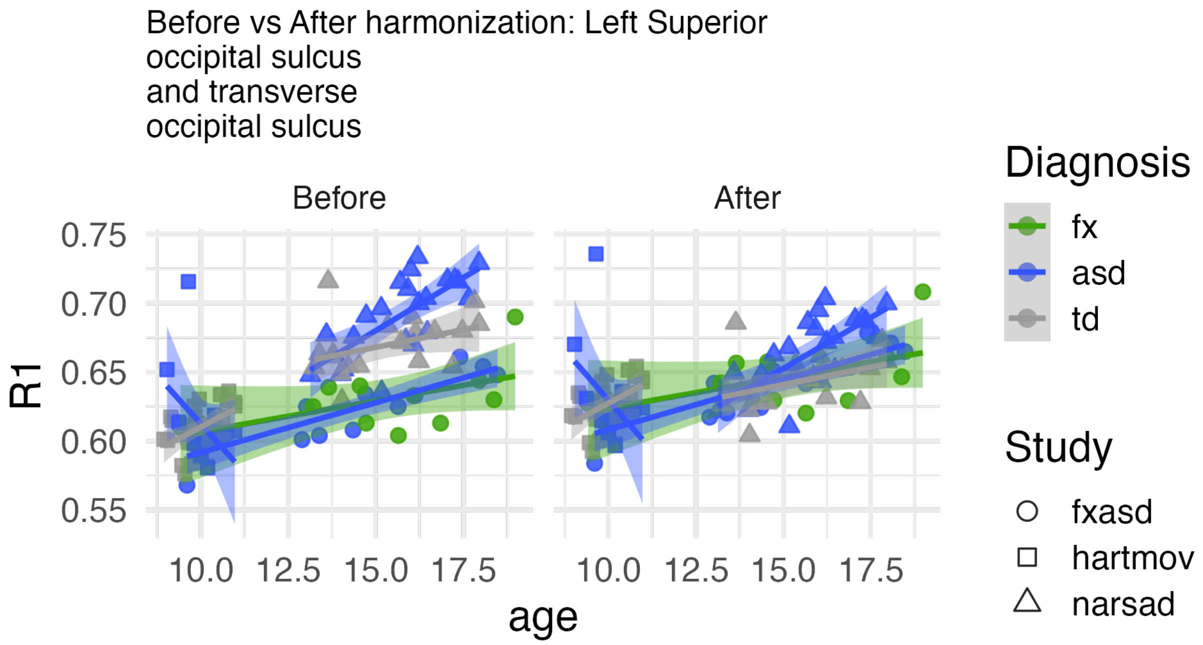

Before vs After harmonization: Left Superior occipital sulcus and transverse occipital sulcus
Before
After
R1
0.75
0.70
0.65
0.60
0.55
10.0
12.5
15.0
17.5
age
Diagnosis
fx
asd
td
Study
fxasd
hartmov
narsad

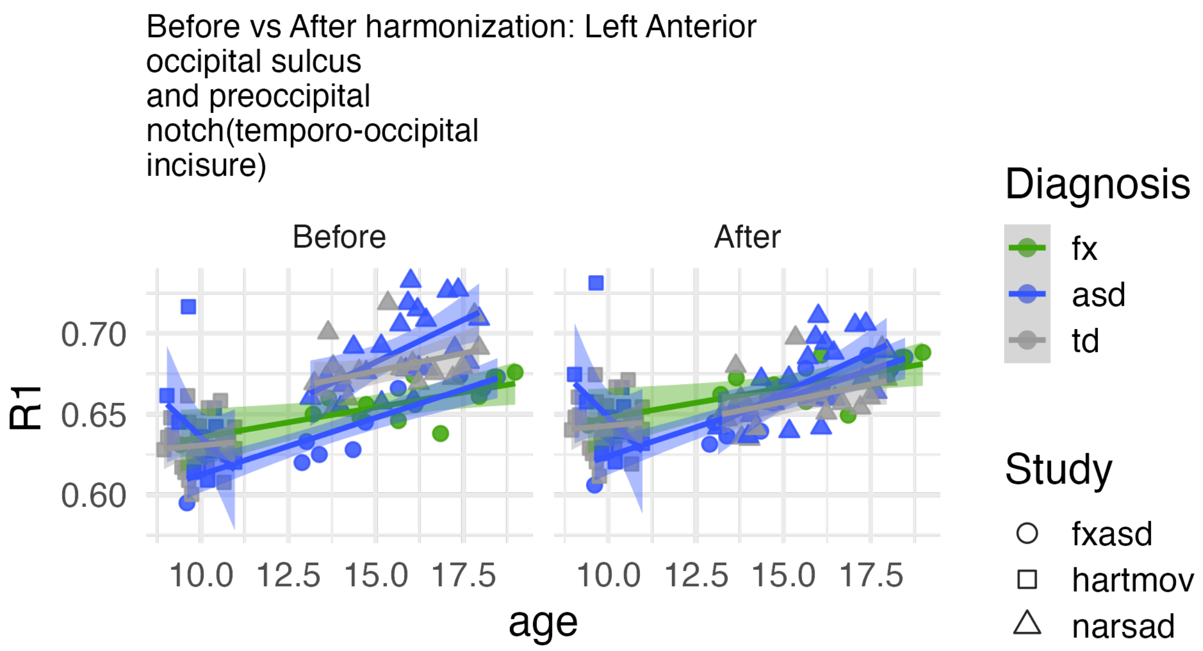

Before vs After harmonization: Left Anterior
occipital sulcus
and preoccipital
notch(temporo-occipital
incisure)
Before
After
R1
0.70
0.65
0.60
10.0
12.5
15.0
17.5
age
Diagnosis
fx
asd
td
Study
fxasd
hartmov
narsad

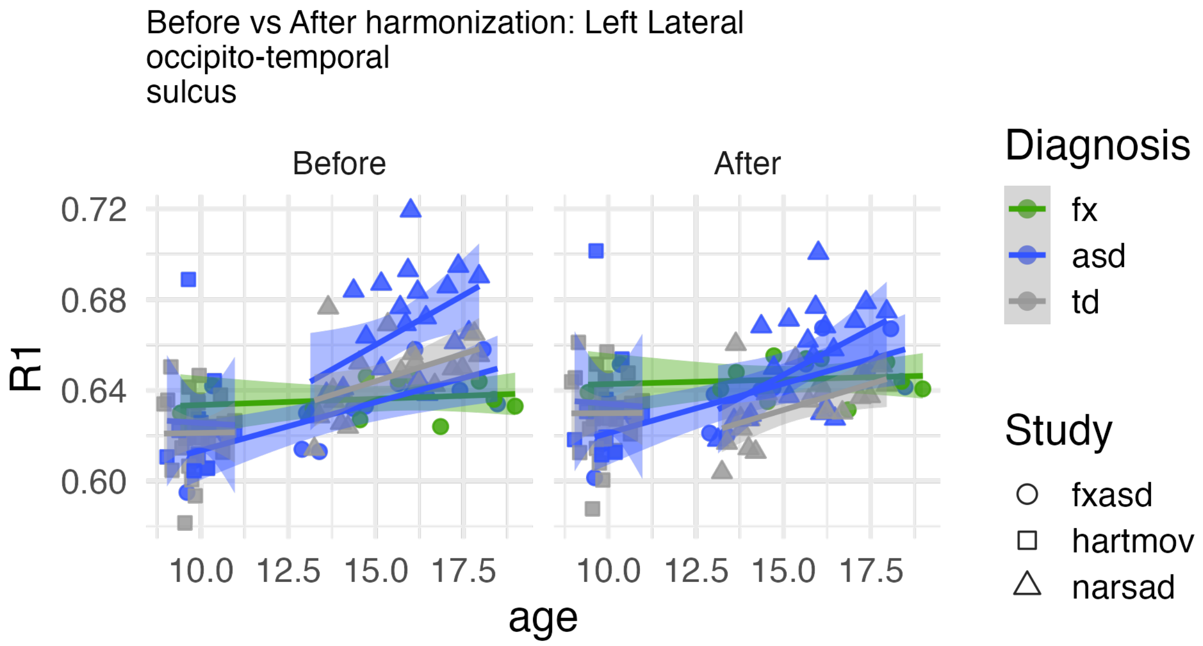

Before vs After harmonization: Left Lateral
occipito-temporal
sulcus
Before
After
R1
0.72
0.68
0.64
0.60
10.0
12.5
15.0
17.5
age
Diagnosis
fx
asd
td
Study
fxasd
hartmov
narsad

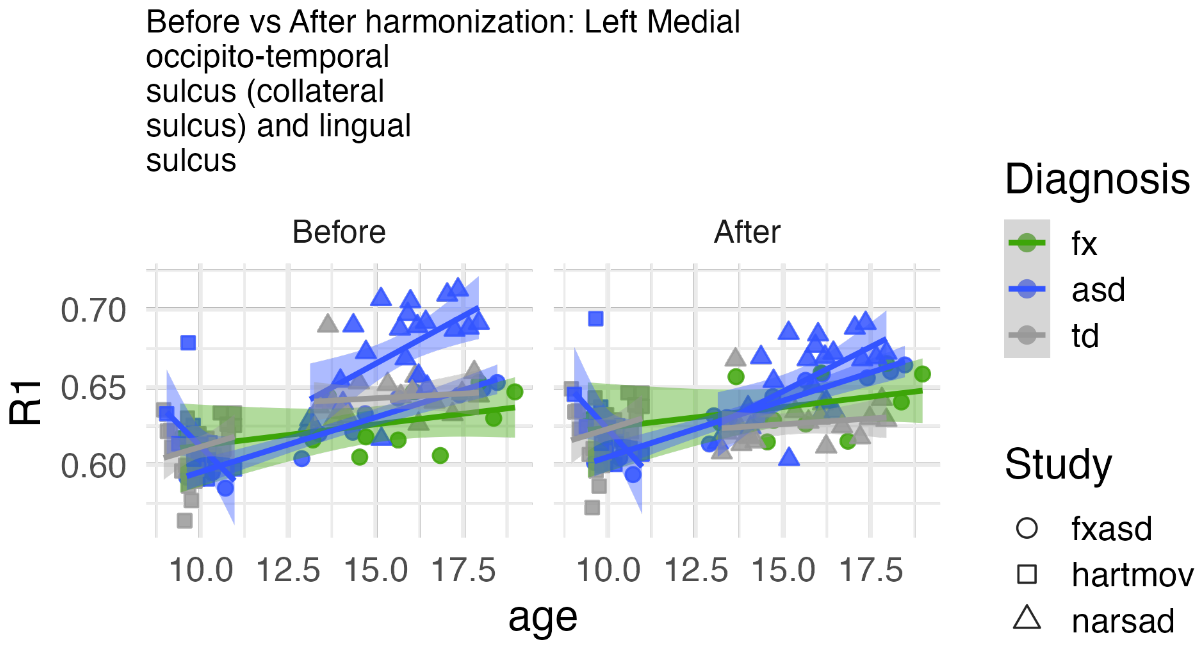
Before vs After harmonization: Left Medial occipito-temporal sulcus (collateral sulcus) and lingual sulcus
Before
After
R1
0.70
0.65
0.60
10.0
12.5
15.0
17.5
age
Diagnosis
fx
asd
td
Study
fxasd
hartmov
narsad

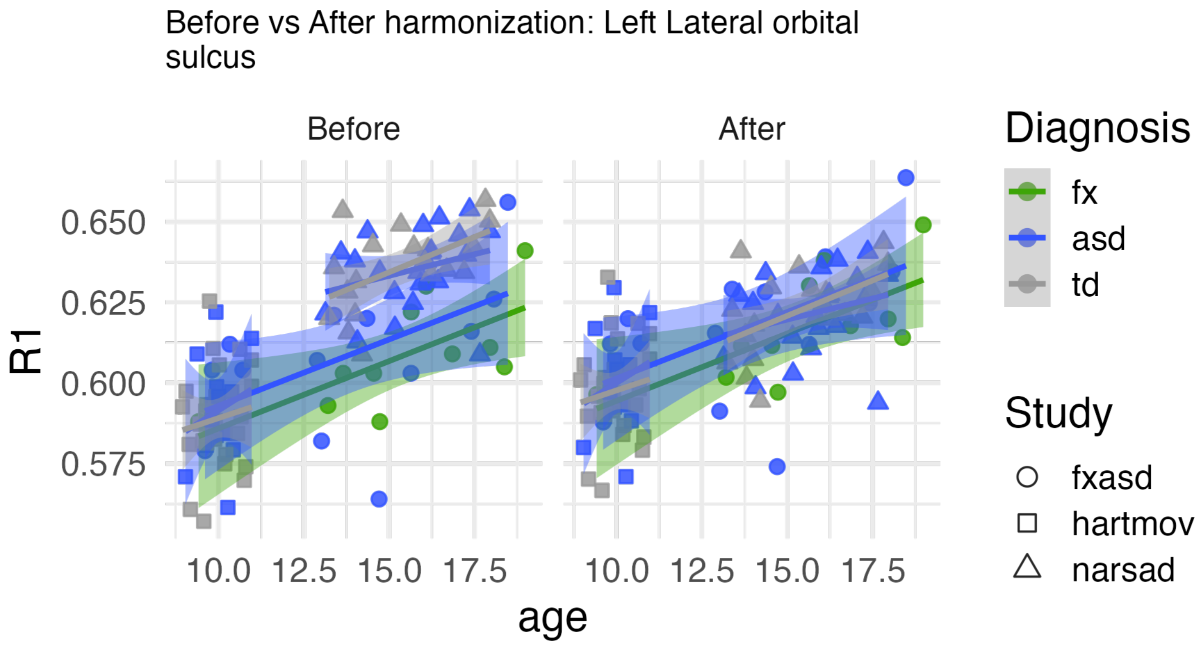
Before vs After harmonization: Left Lateral orbital sulcus
Before
After
R1
0.650
0.625
0.600
0.575
10.0
12.5
15.0
17.5
age
Diagnosis
fx
asd
td
Study
fxasd
hartmov
narsad

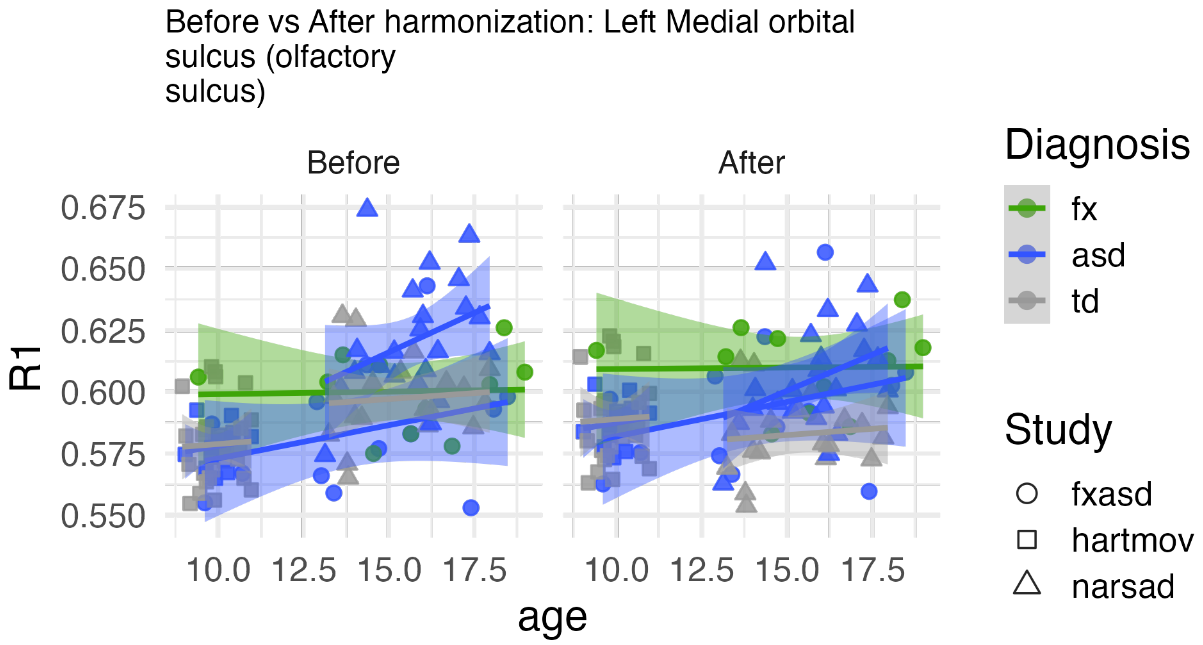
Before vs After harmonization: Left Medial orbital
sulcus (olfactory
sulcus)
Before
After
0.675
0.650
0.625
0.600
0.575
0.550
R1
10.0 12.5 15.0 17.5
10.0 12.5 15.0 17.5
age
Diagnosis
fx
asd
td
Study
fxasd
hartmov
narsad

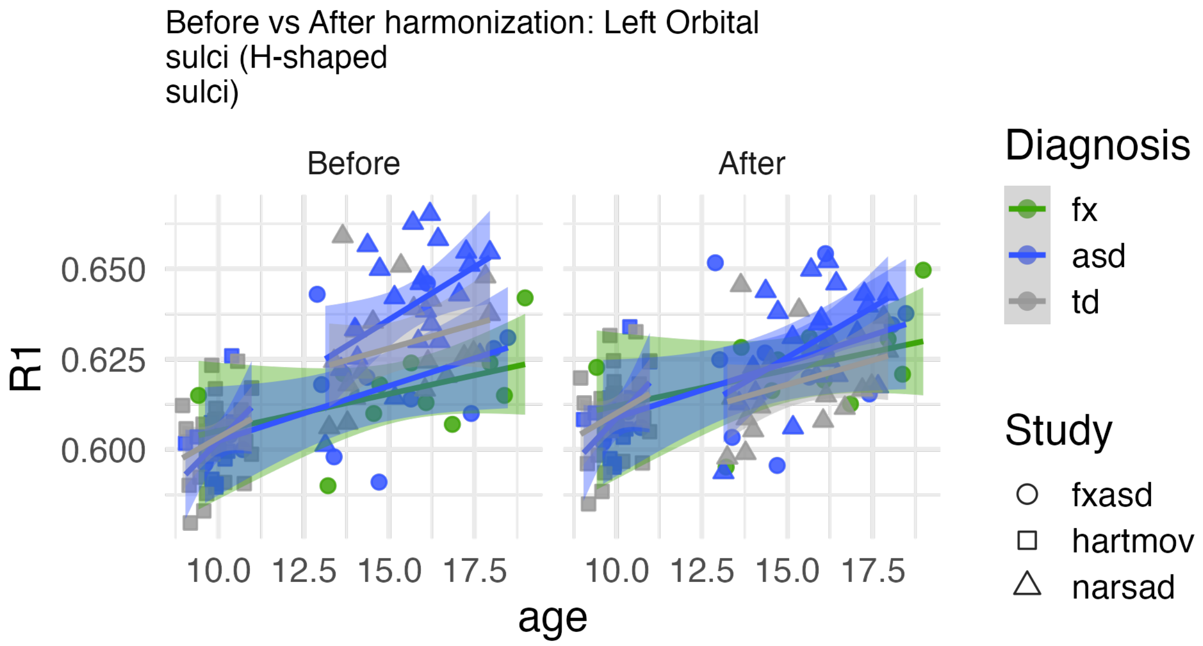
Before vs After harmonization: Left Orbital
sulci (H-shaped
sulci)
Before
After
0.650
0.625
0.600
R1
10.0 12.5 15.0 17.5
10.0 12.5 15.0 17.5
age
Diagnosis
fx
asd
td
Study
fxasd
hartmov
narsad

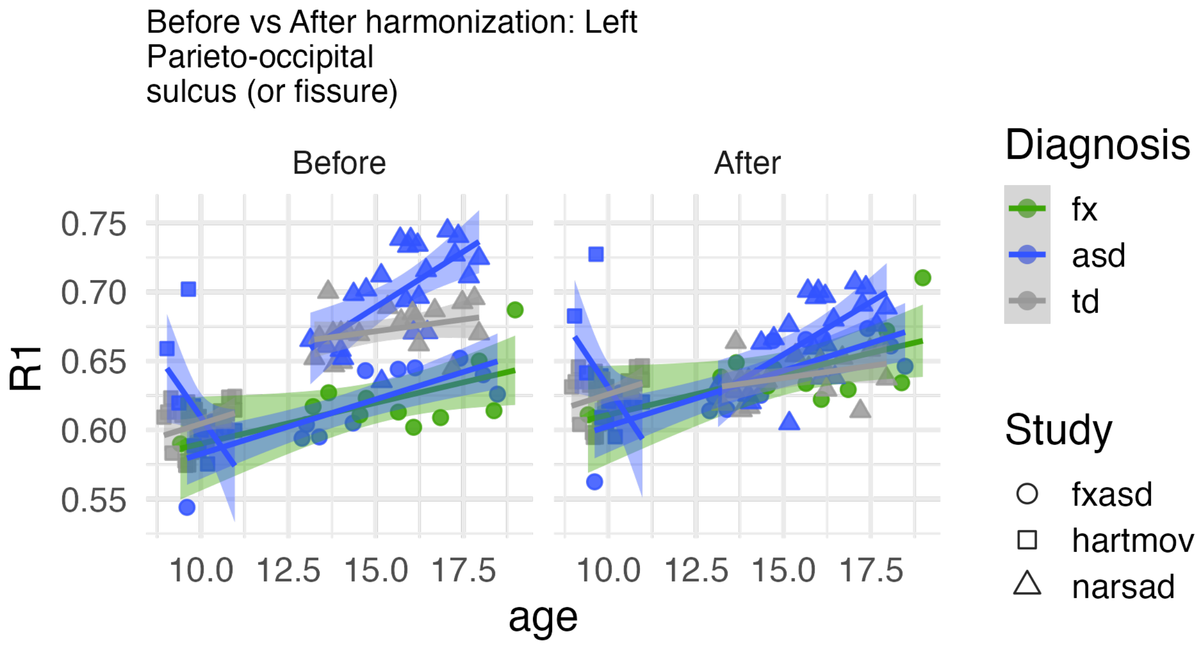

Before vs After harmonization: Left
Parieto-occipital
sulcus (or fissure)
Before
After
R1
0.75
0.70
0.65
0.60
0.55
10.0
12.5
15.0
17.5
age
Diagnosis
fx
asd
td
Study
fxasd
hartmov
narsad

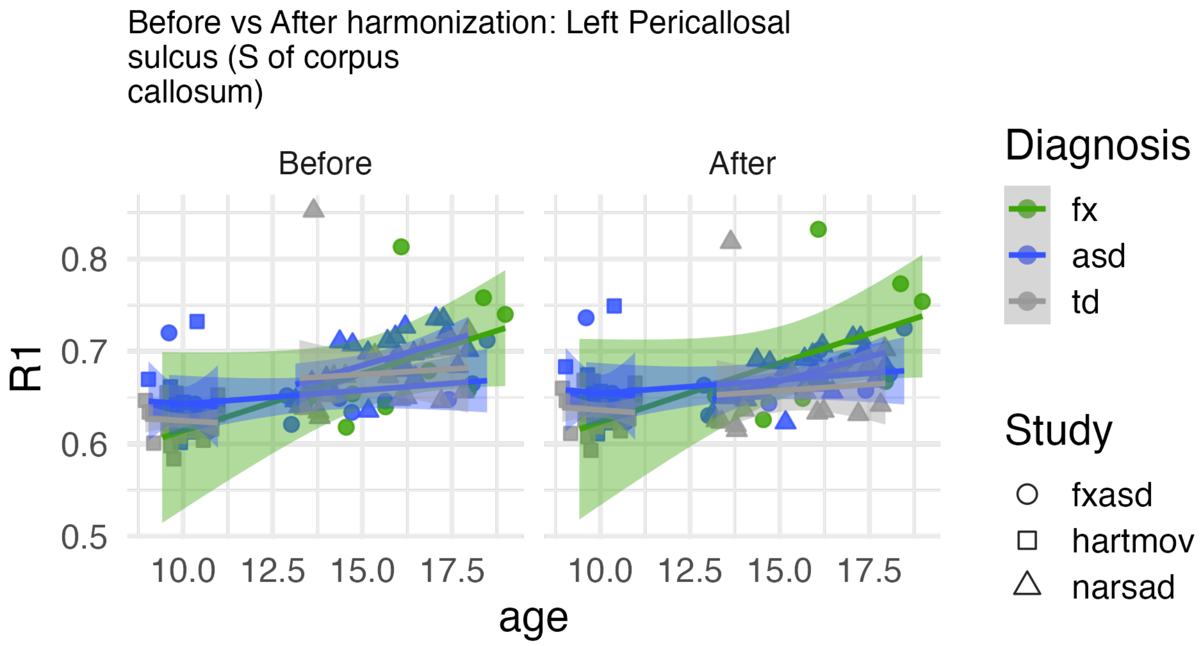

Before vs After harmonization: Left Pericallosal
sulcus (S of corpus
callosum)
Before
After
R1
0.8
0.7
0.6
0.5
10.0
12.5
15.0
17.5
age
Diagnosis
fx
asd
td
Study
fxasd
hartmov
narsad

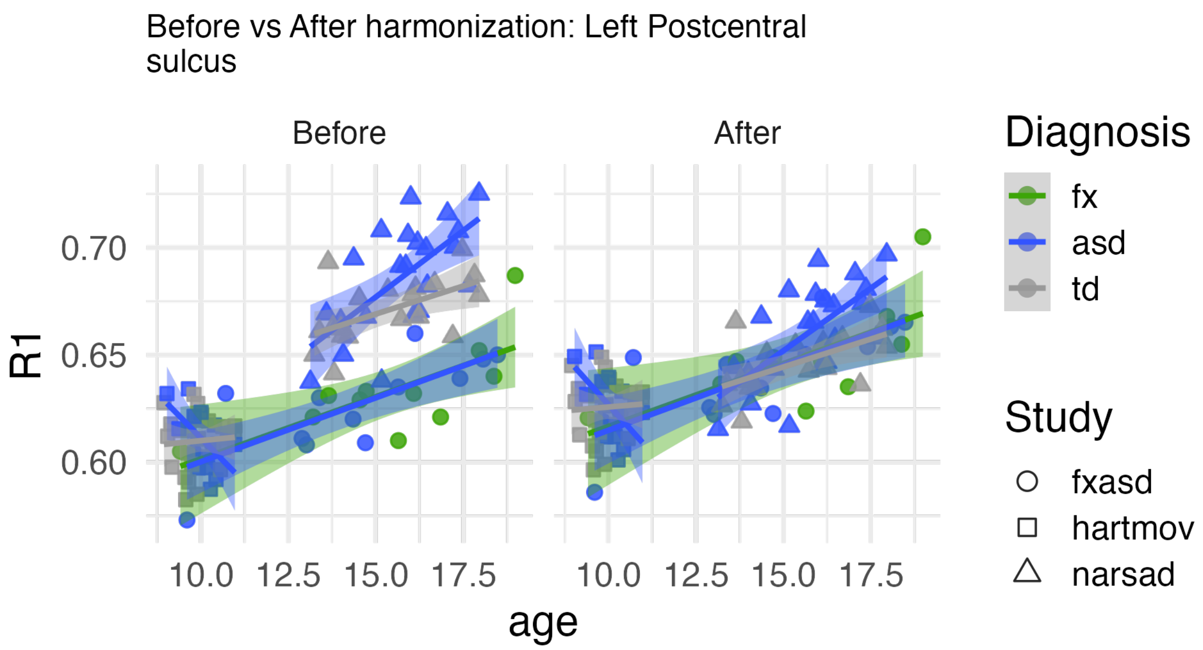
Before vs After harmonization: Left Postcentral
sulcus
Before
After
R1
0.70
0.65
0.60
10.0 12.5 15.0 17.5
10.0 12.5 15.0 17.5
age
Diagnosis
fx
asd
td
Study
fxasd
hartmov
narsad

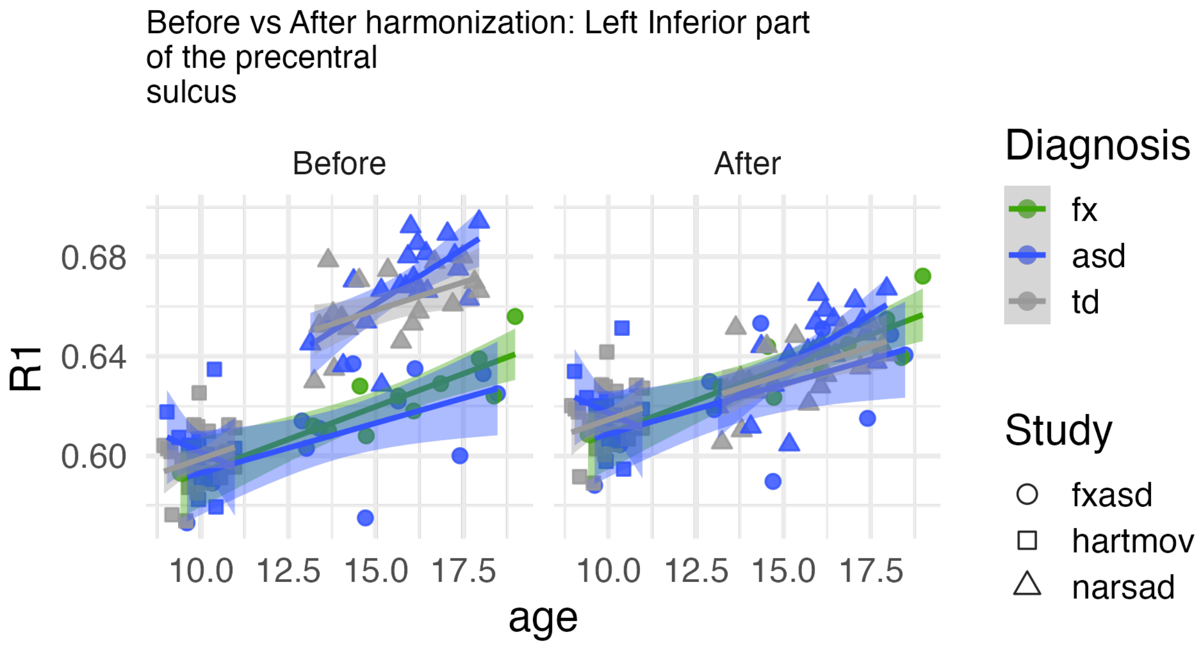
Before vs After harmonization: Left Inferior part
of the precentral
sulcus
Before
After
R1
0.68
0.64
0.60
10.0 12.5 15.0 17.5
10.0 12.5 15.0 17.5
age
Diagnosis
fx
asd
td
Study
fxasd
hartmov
narsad

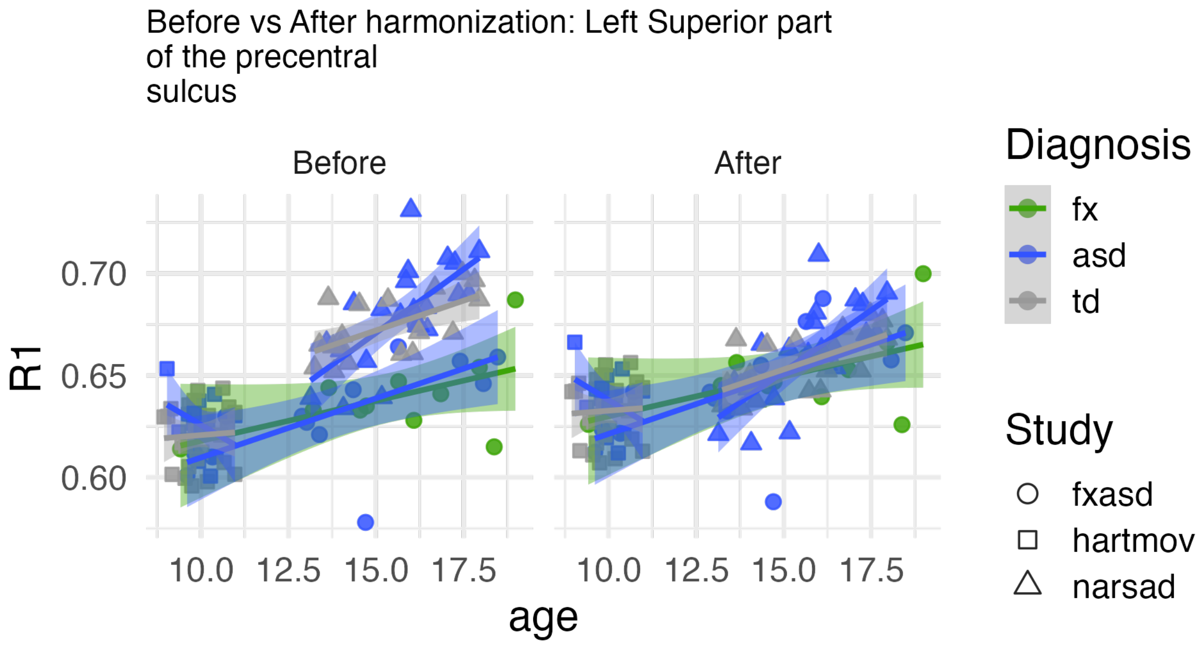
Before vs After harmonization: Left Superior part
of the precentral
sulcus
Before
After
R1
0.70
0.65
0.60
10.0 12.5 15.0 17.5
10.0 12.5 15.0 17.5
age
Diagnosis
fx
asd
td
Study
fxasd
hartmov
narsad

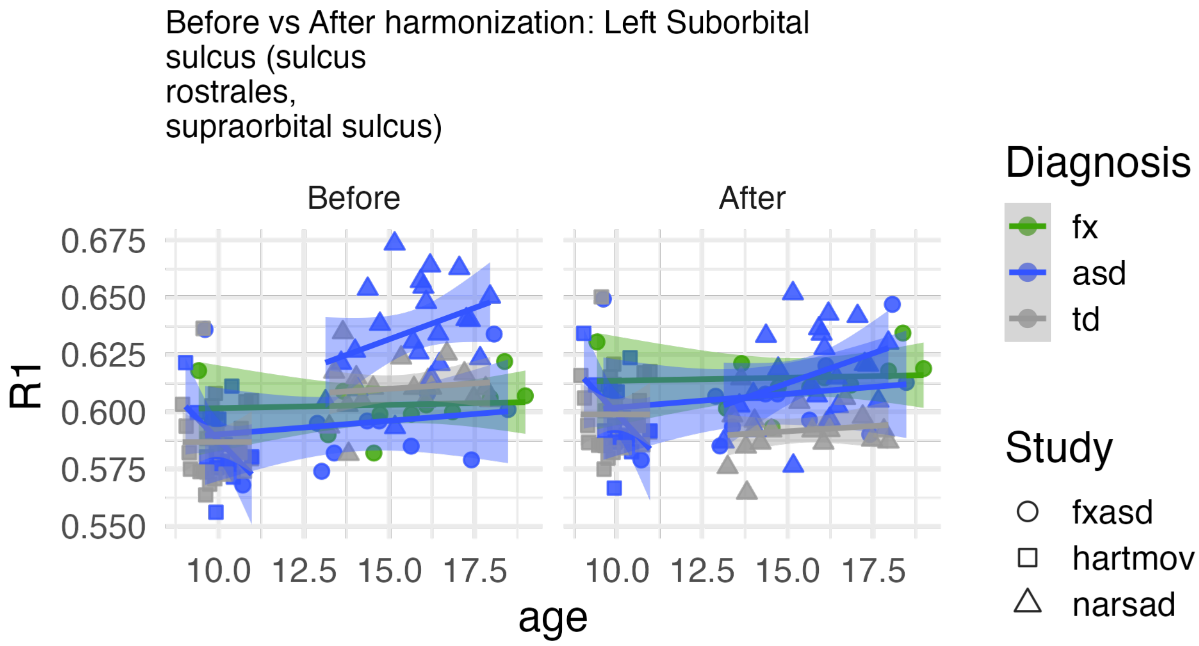
Before vs After harmonization: Left Suborbital
sulcus (sulcus
rostrales,
supraorbital sulcus)
Before
After
R1
0.675
0.650
0.625
0.600
0.575
0.550
10.0 12.5 15.0 17.5
10.0 12.5 15.0 17.5
age
Diagnosis
fx
asd
td
Study
fxasd
hartmov
narsad

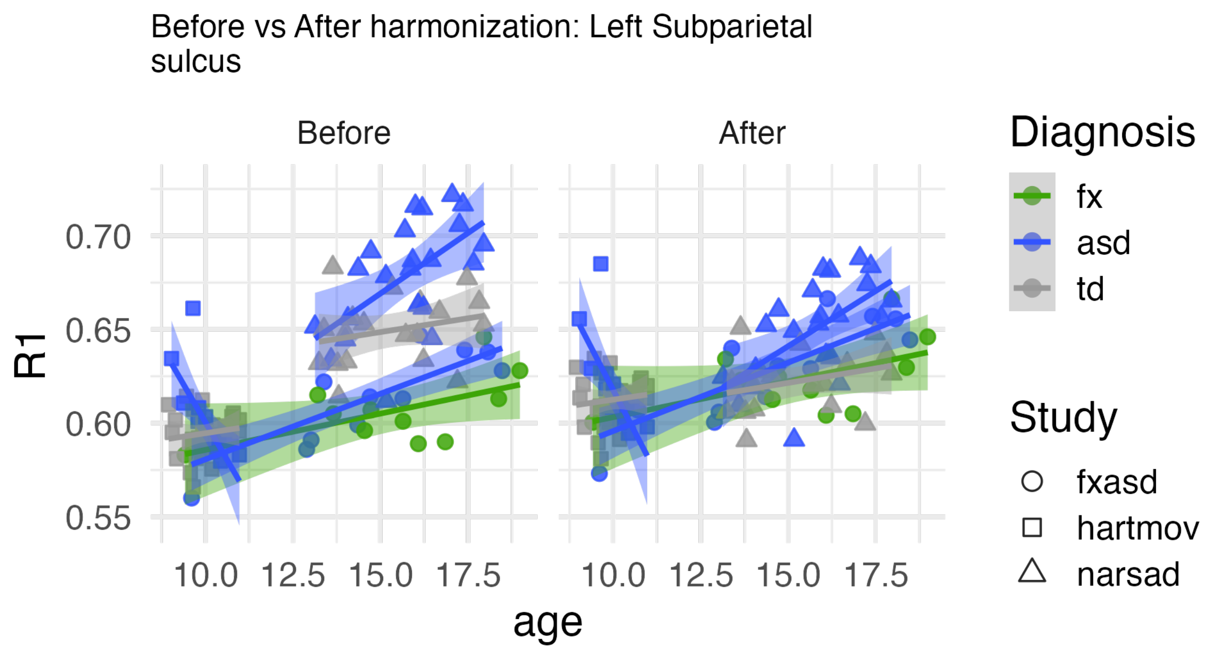
Before vs After harmonization: Left Subparietal sulcus
Before
After
R1
0.70
0.65
0.60
0.55
10.0
12.5
15.0
17.5
age
Diagnosis
fx
asd
td
Study
fxasd
hartmov
narsad

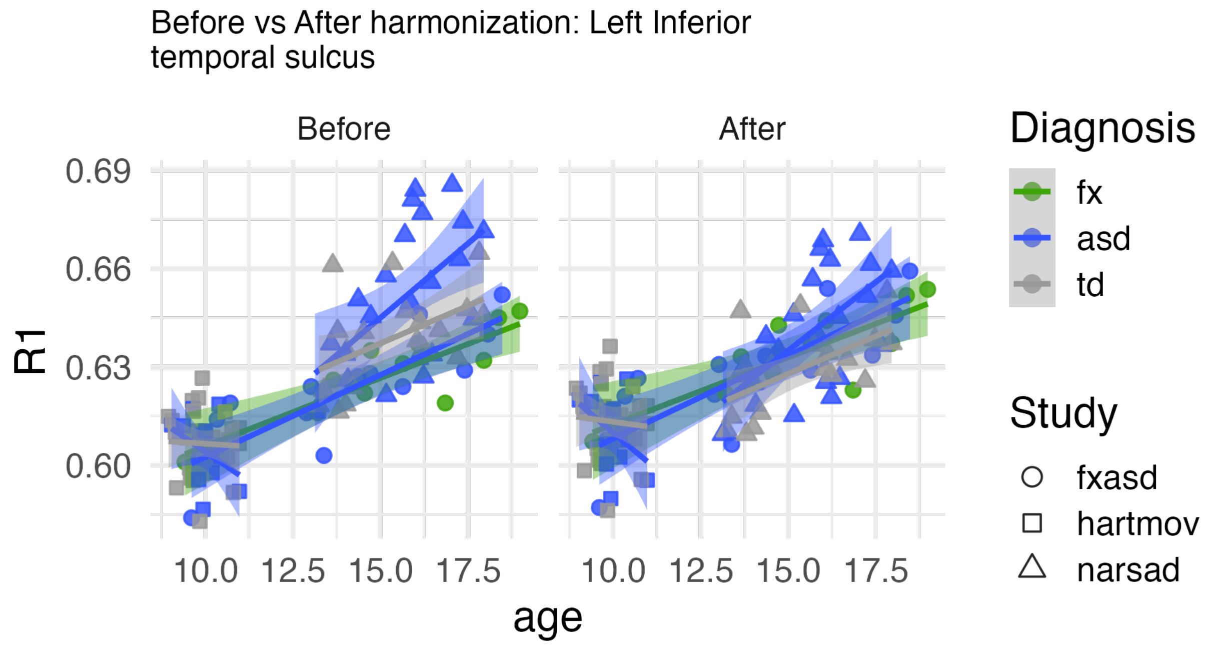
Before vs After harmonization: Left Inferior temporal sulcus
Before
After
R1
0.69
0.66
0.63
0.60
10.0
12.5
15.0
17.5
age
Diagnosis
fx
asd
td
Study
fxasd
hartmov
narsad

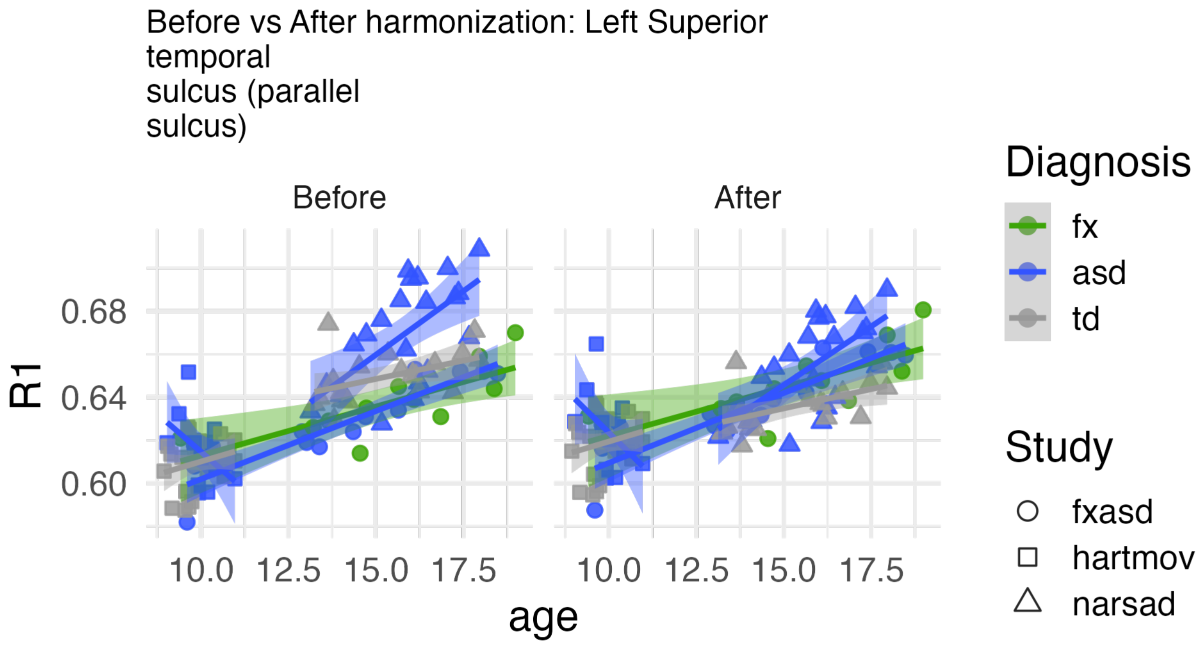
Before vs After harmonization: Left Superior
temporal
sulcus (parallel
sulcus)
Before
After
R1
0.68
0.64
0.60
10.0
12.5
15.0
17.5
age
Diagnosis
fx
asd
td
Study
fxasd
hartmov
narsad

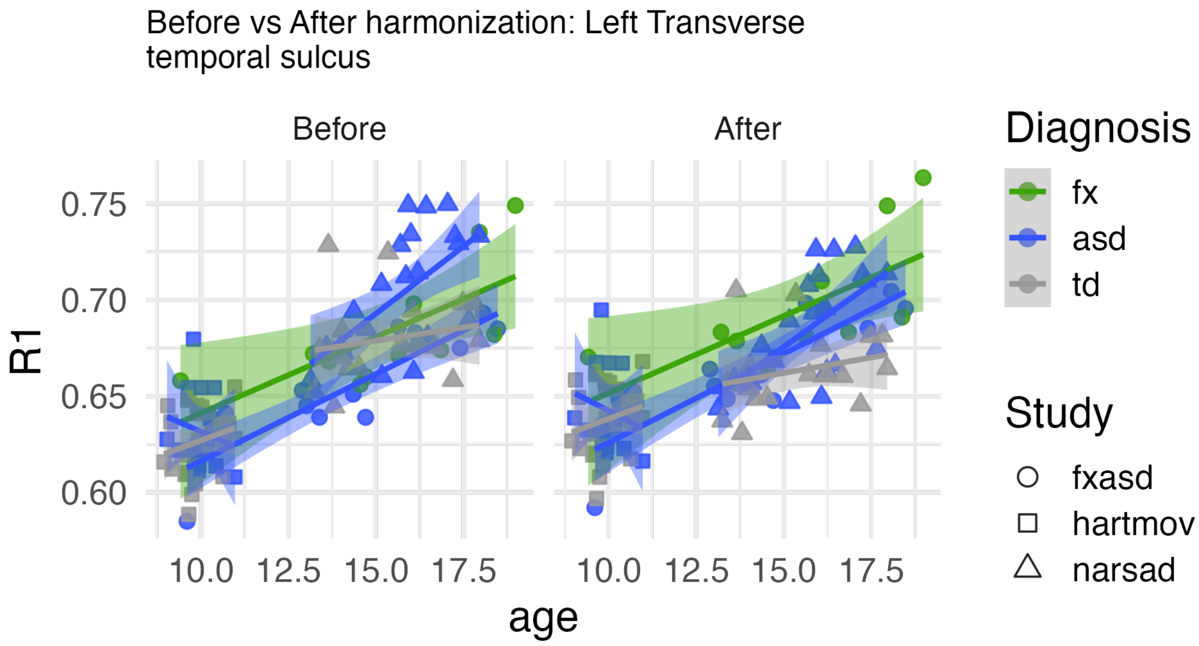
Before vs After harmonization: Left Transverse
temporal sulcus
Before
After
R1
0.75
0.70
0.65
0.60
10.0
12.5
15.0
17.5
age
Diagnosis
fx
asd
td
Study
fxasd
hartmov
narsad

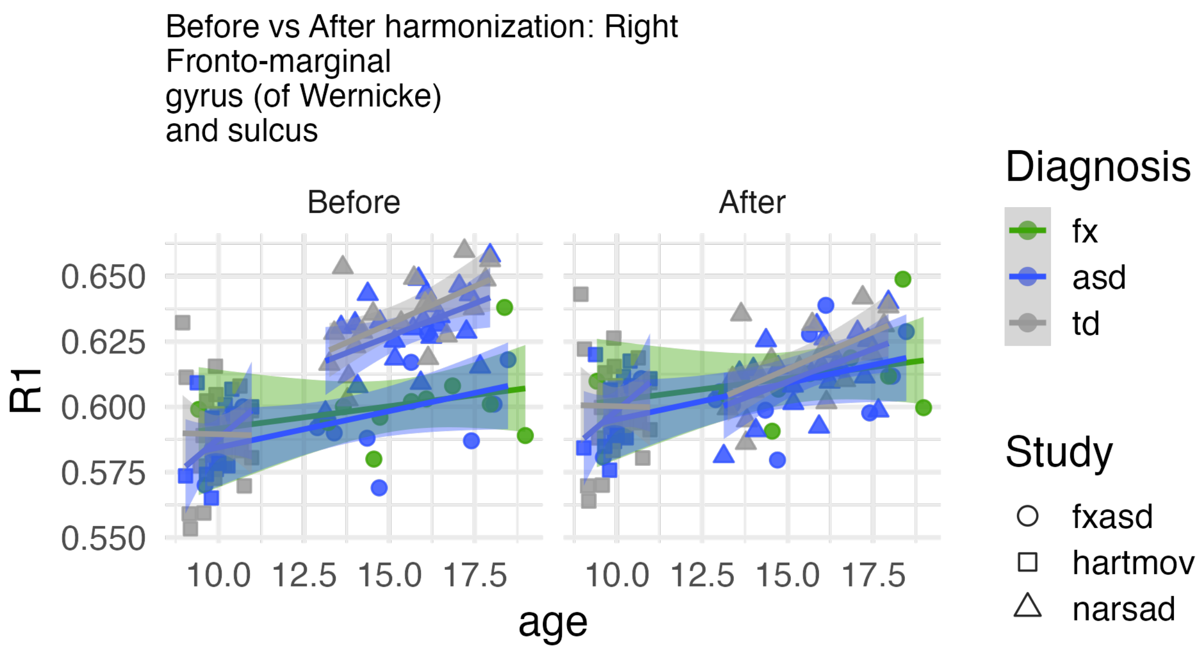

Before vs After harmonization: Right
Fronto-marginal
gyrus (of Wernicke)
and sulcus
Before
After
R1
0.650
0.625
0.600
0.575
0.550
10.0 12.5 15.0 17.5
age
Diagnosis
fx
asd
td
Study
fxasd
hartmov
narsad

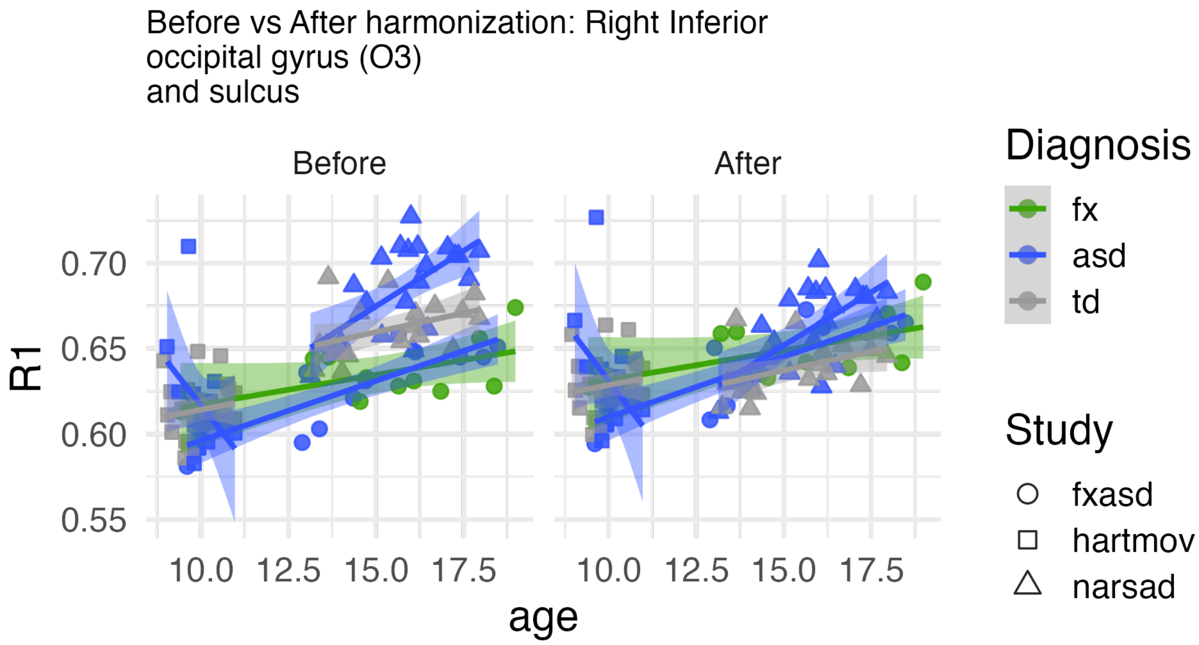

Before vs After harmonization: Right Inferior
occipital gyrus (O3)
and sulcus
Before
After
R1
0.70
0.65
0.60
0.55
10.0 12.5 15.0 17.5
age
Diagnosis
fx
asd
td
Study
fxasd
hartmov
narsad

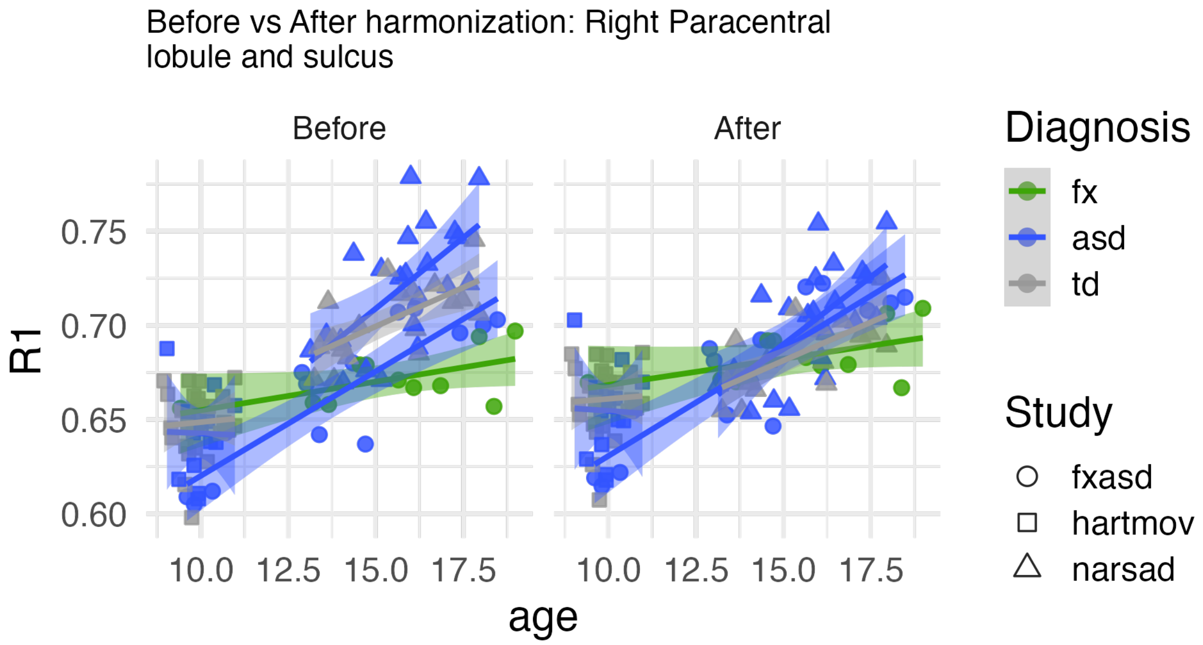
Before vs After harmonization: Right Paracentral
lobule and sulcus
Before
After
R1
0.60
0.65
0.70
0.75
10.0
12.5
15.0
17.5
age
Diagnosis
fx
asd
td
Study
fxasd
hartmov
narsad

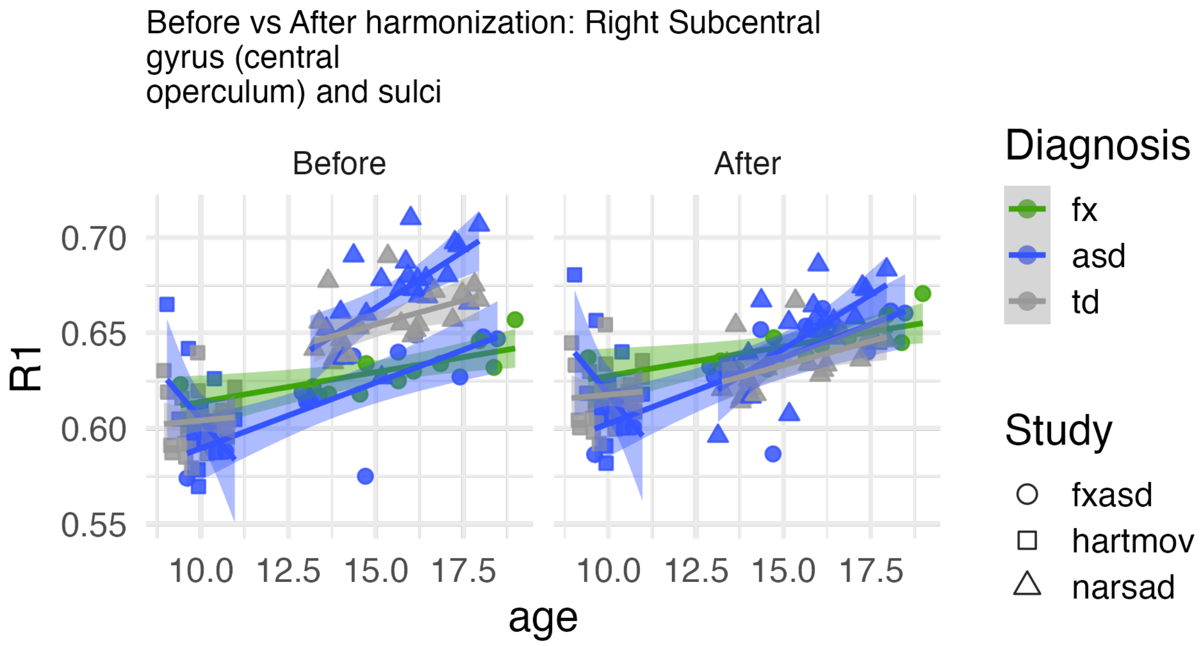
Before vs After harmonization: Right Subcentral
gyrus (central
operculum) and sulci
Before
After
R1
0.55
0.60
0.65
0.70
10.0
12.5
15.0
17.5
age
Diagnosis
fx
asd
td
Study
fxasd
hartmov
narsad

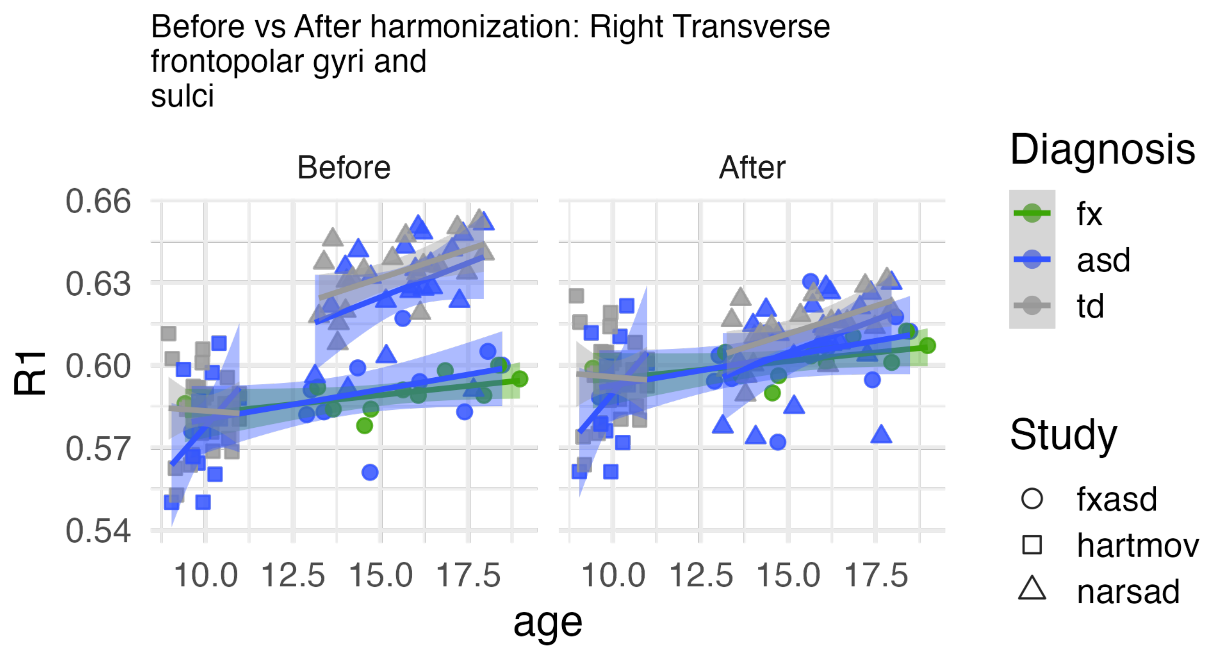
Before vs After harmonization: Right Transverse
frontopolar gyri and
sulci
Before
After
0.66
0.63
0.60
0.57
0.54
R1
10.0
12.5
15.0
17.5
age
Diagnosis
fx
asd
td
Study
fxasd
hartmov
narsad

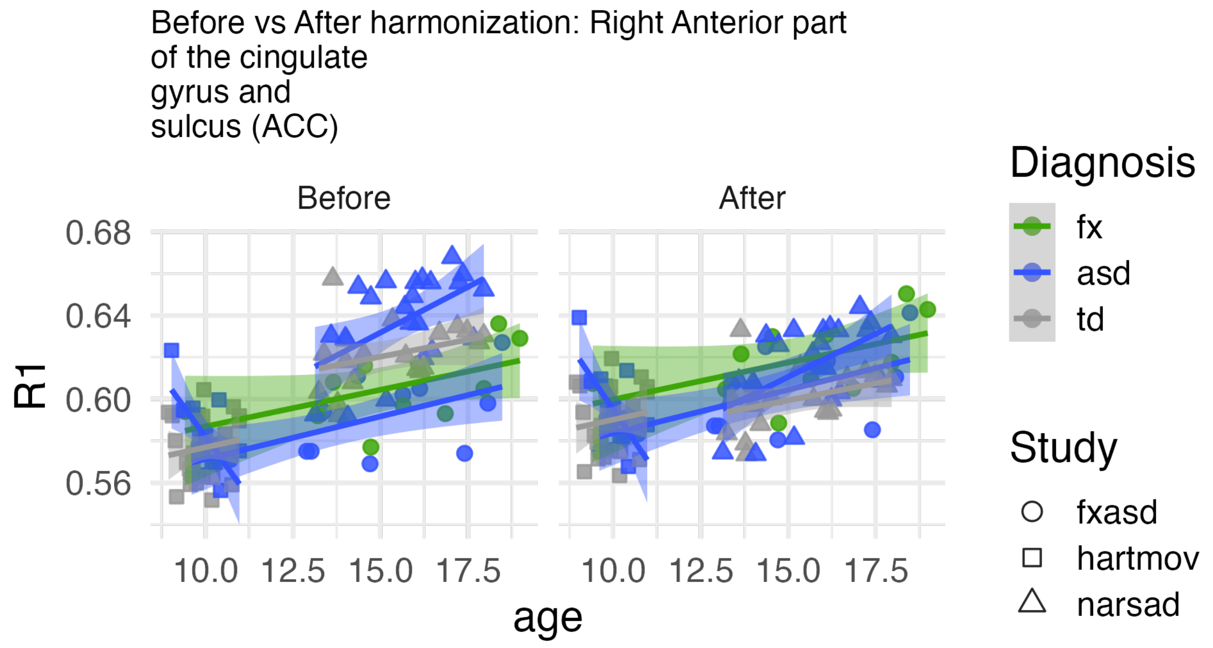
Before vs After harmonization: Right Anterior part
of the cingulate
gyrus and
sulcus (ACC)
Before
After
0.68
0.64
0.60
0.56
R1
10.0
12.5
15.0
17.5
age
Diagnosis
fx
asd
td
Study
fxasd
hartmov
narsad

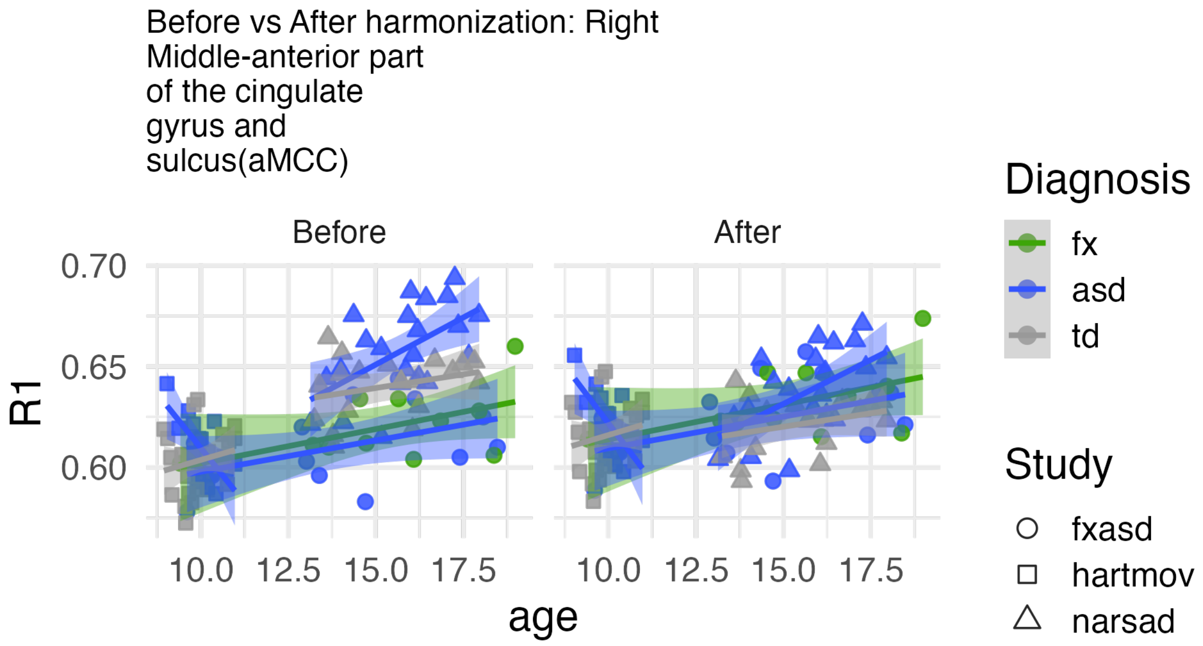
Before vs After harmonization: Right
Middle-anterior part
of the cingulate
gyrus and
sulcus(aMCC)
Before
After
0.70
0.65
0.60
R1
10.0
12.5
15.0
17.5
age
Diagnosis
fx
asd
td
Study
fxasd
hartmov
narsad

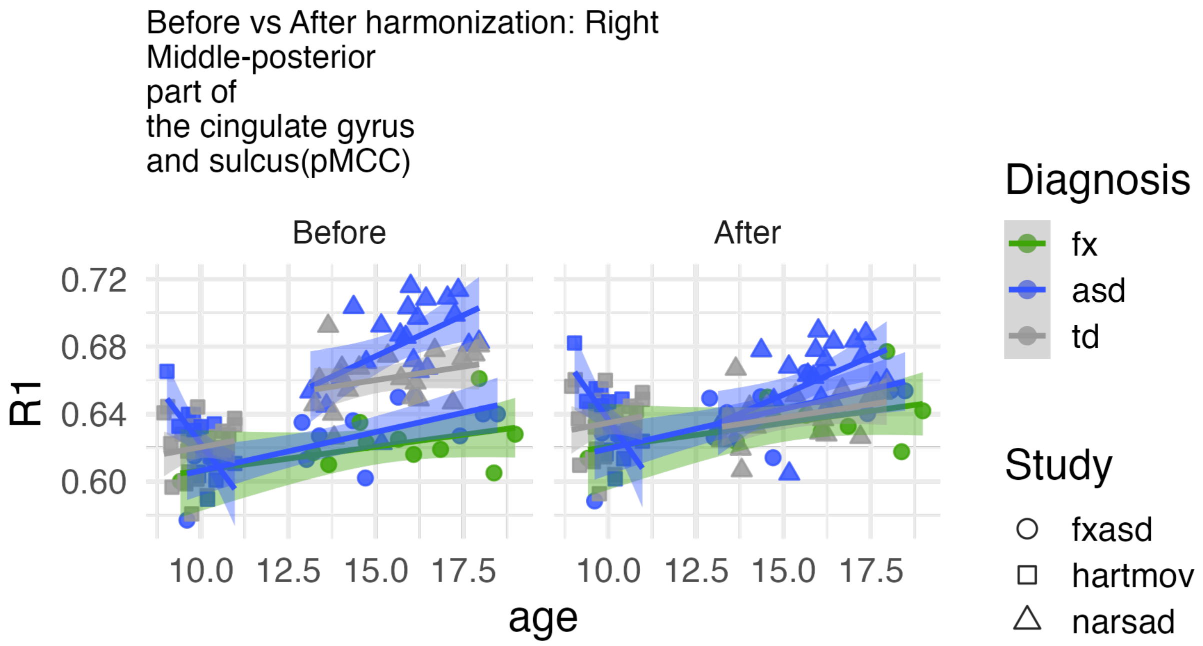
Before vs After harmonization: Right
Middle-posterior
part of
the cingulate gyrus
and sulcus(pMCC)
Before
After
0.72
0.68
0.64
0.60
R1
10.0
12.5
15.0
17.5
age
Diagnosis
fx
asd
td
Study
fxasd
hartmov
narsad

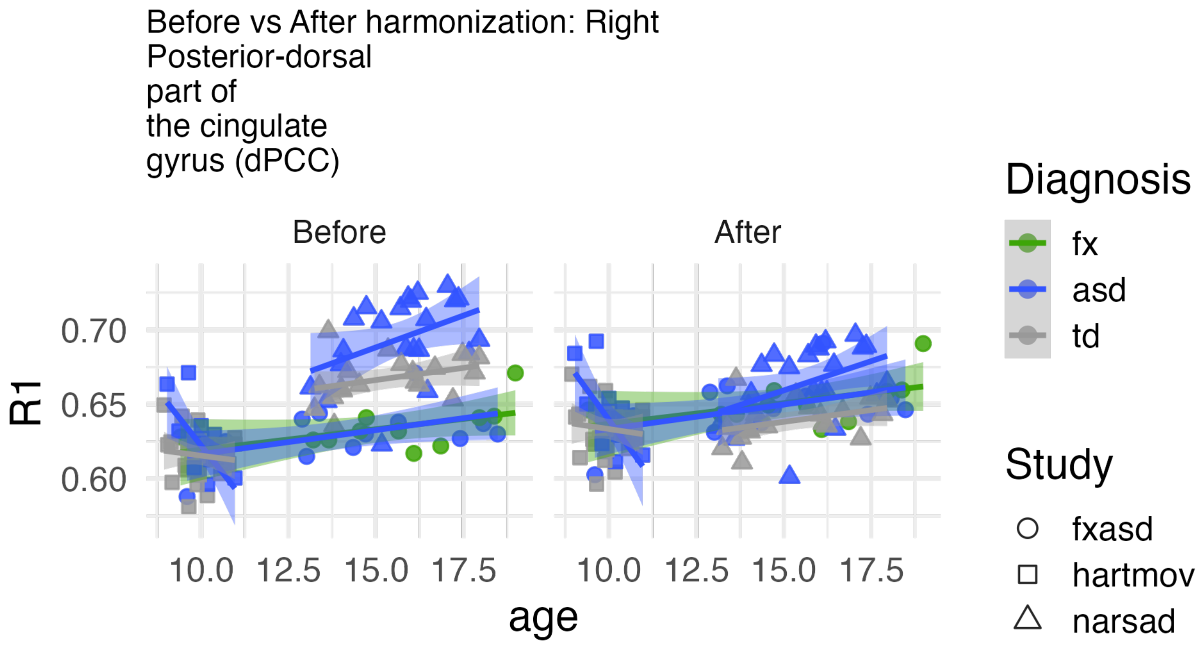
Before vs After harmonization: Right
Posterior-dorsal
part of
the cingulate
gyrus (dPCC)
Before
After
R1
0.70
0.65
0.60
10.0
12.5
15.0
17.5
age
Diagnosis
fx
asd
td
Study
fxasd
hartmov
narsad

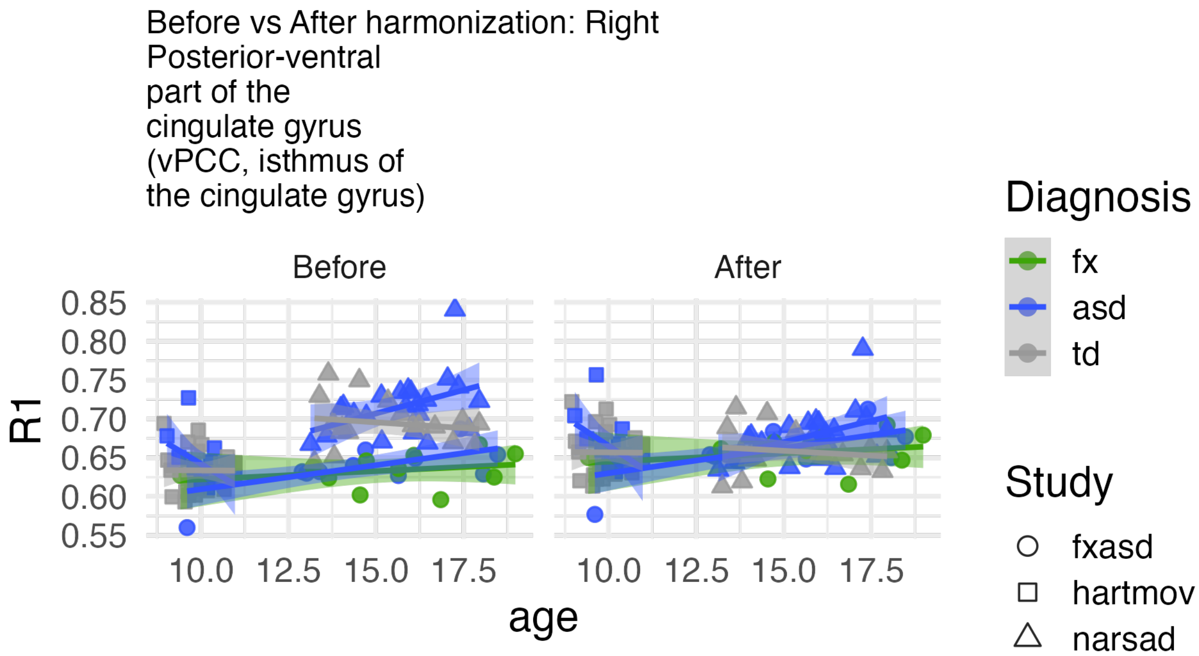
Before vs After harmonization: Right
Posterior-ventral
part of the
cingulate gyrus
(vPCC, isthmus of
the cingulate gyrus)
Before
After
R1
0.85
0.80
0.75
0.70
0.65
0.60
0.55
10.0
12.5
15.0
17.5
age
Diagnosis
fx
asd
td
Study
fxasd
hartmov
narsad

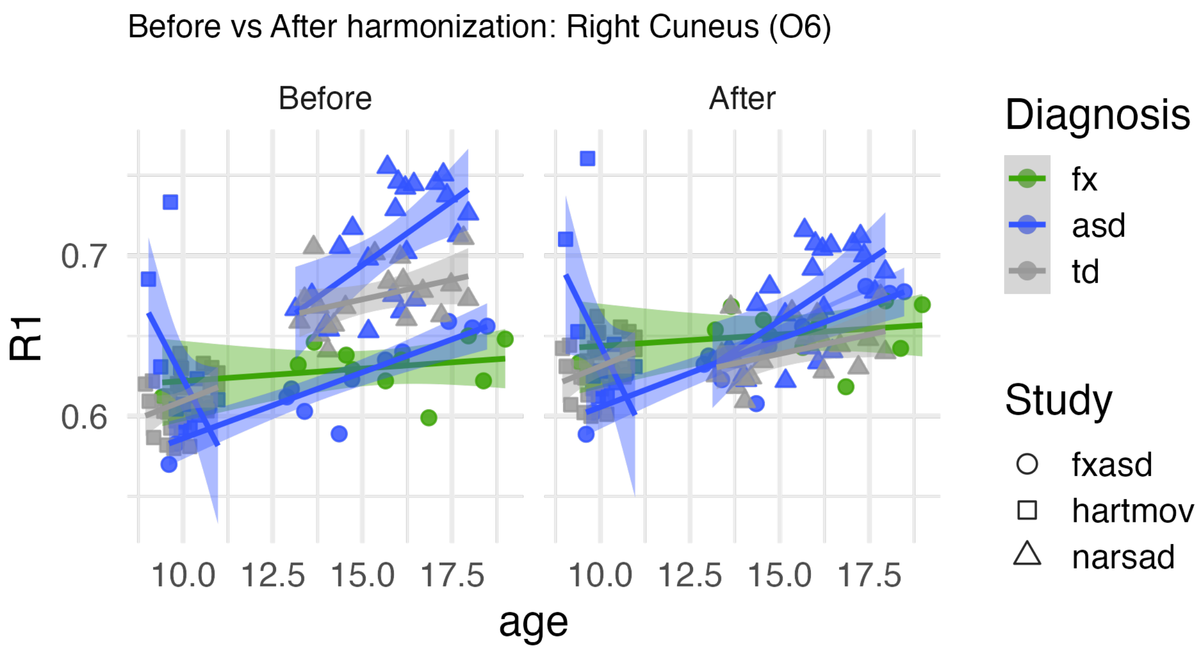
Before vs After harmonization: Right Cuneus (O6)
Before
After
0.7
0.6
R1
10.0
12.5
15.0
17.5
age
Diagnosis
fx
asd
td
Study
fxasd
hartmov
narsad

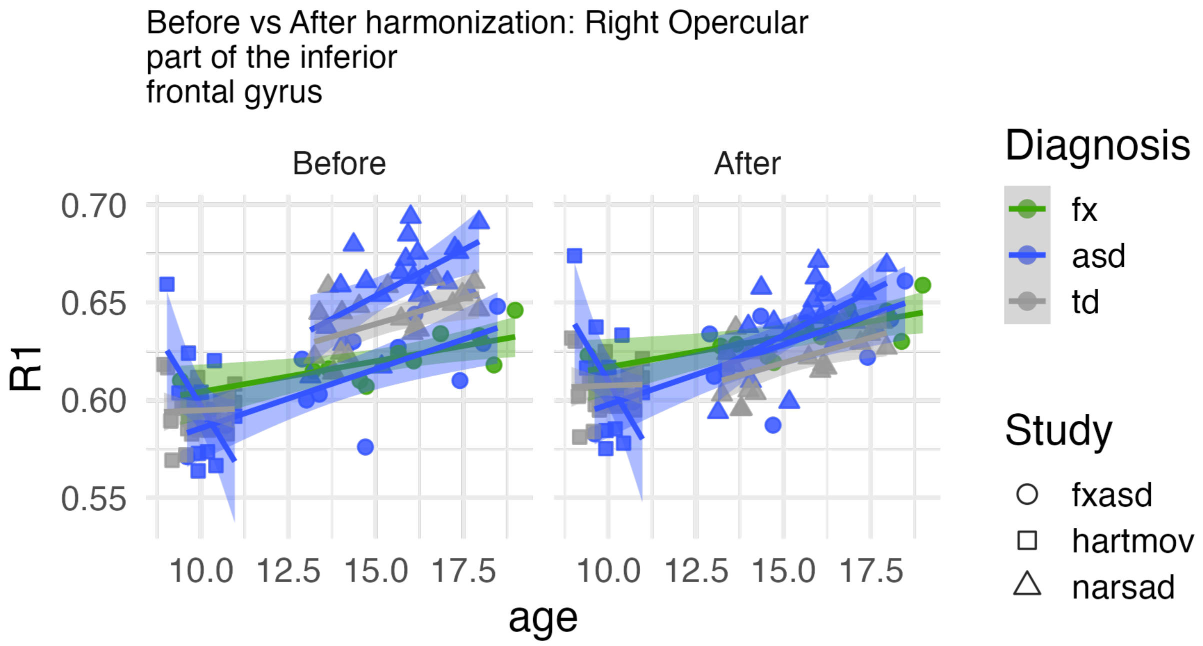
Before vs After harmonization: Right Opercular
part of the inferior
frontal gyrus
Before
After
0.70
0.65
0.60
0.55
R1
10.0
12.5
15.0
17.5
age
Diagnosis
fx
asd
td
Study
fxasd
hartmov
narsad

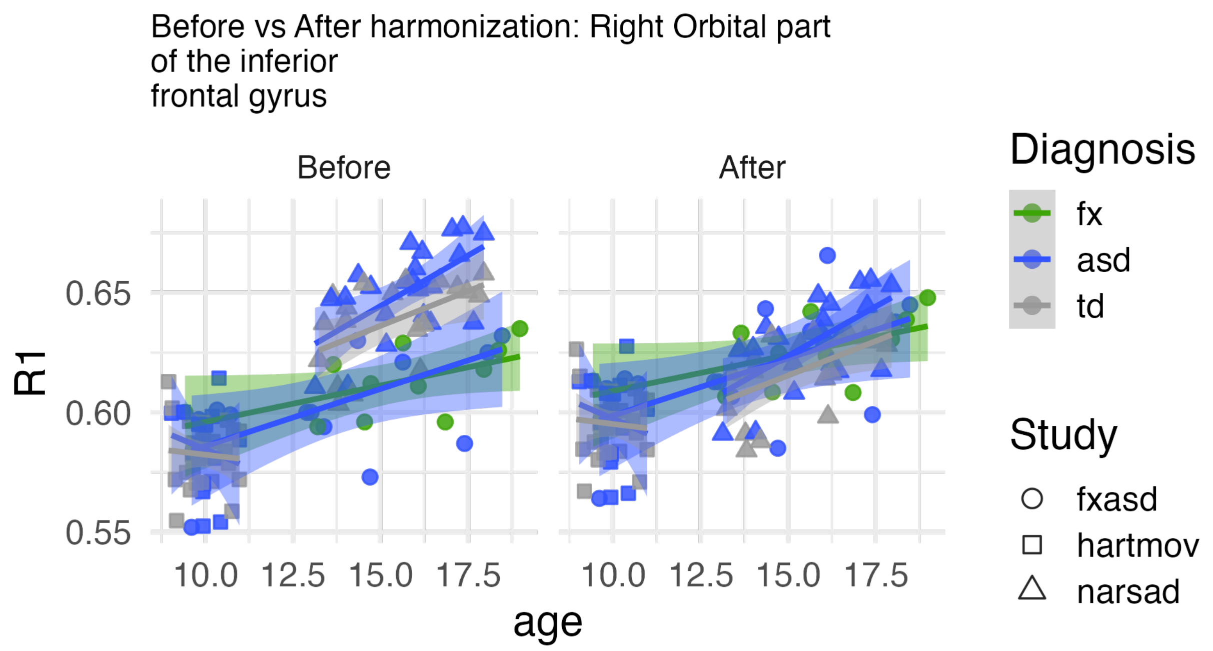
Before vs After harmonization: Right Orbital part
of the inferior
frontal gyrus
Before
After
R1
0.55
0.60
0.65
10.0
12.5
15.0
17.5
age
Diagnosis
fx
asd
td
Study
fxasd
hartmov
narsad

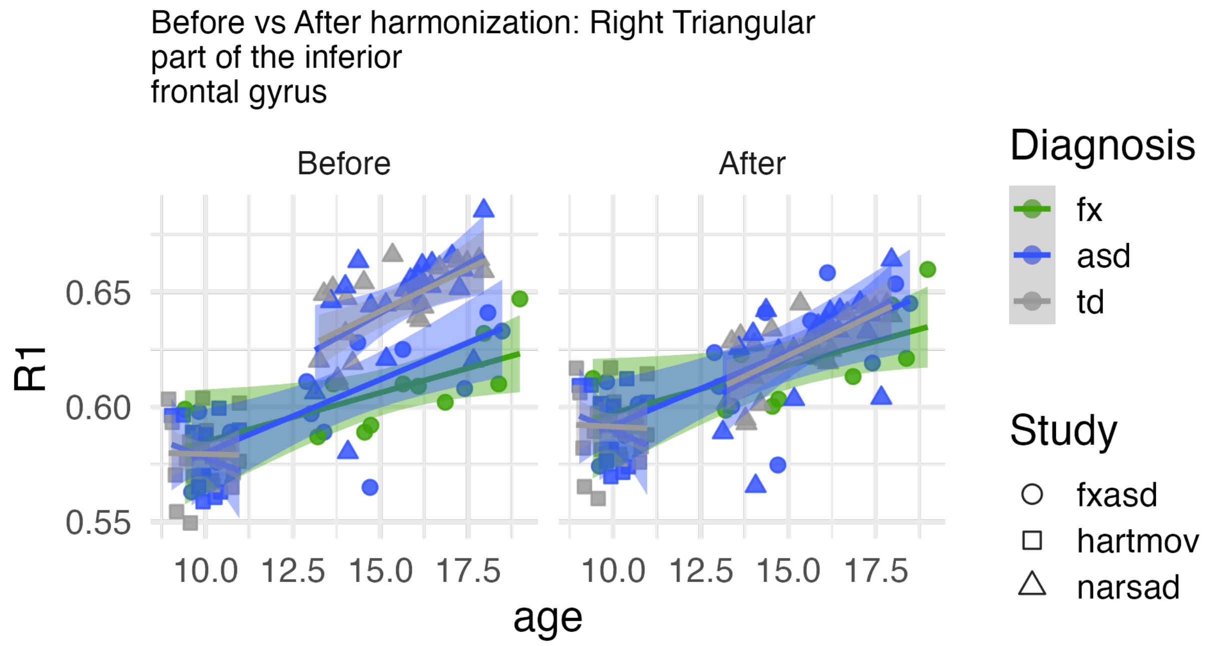
Before vs After harmonization: Right Triangular
part of the inferior
frontal gyrus
Before
After
R1
0.55
0.60
0.65
10.0
12.5
15.0
17.5
age
Diagnosis
fx
asd
td
Study
fxasd
hartmov
narsad

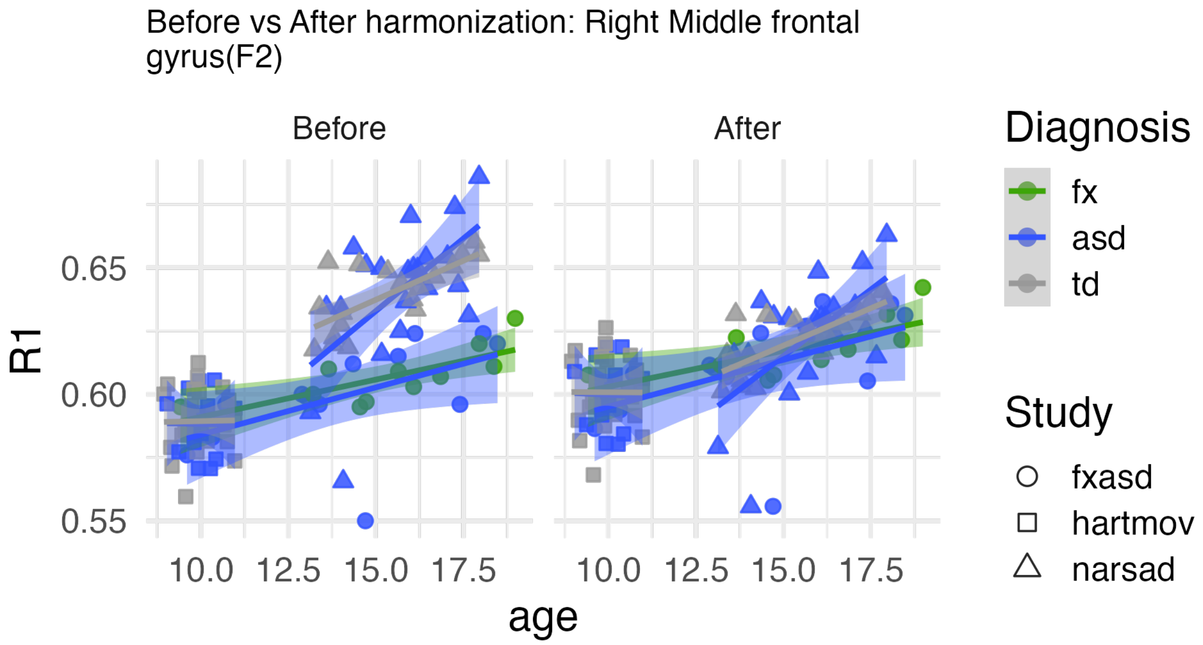

Before vs After harmonization: Right Middle frontal gyrus(F2)
Before
After
R1
0.65
0.60
0.55
10.0
12.5
15.0
17.5
age
Diagnosis
fx
asd
td
Study
fxasd
hartmov
narsad

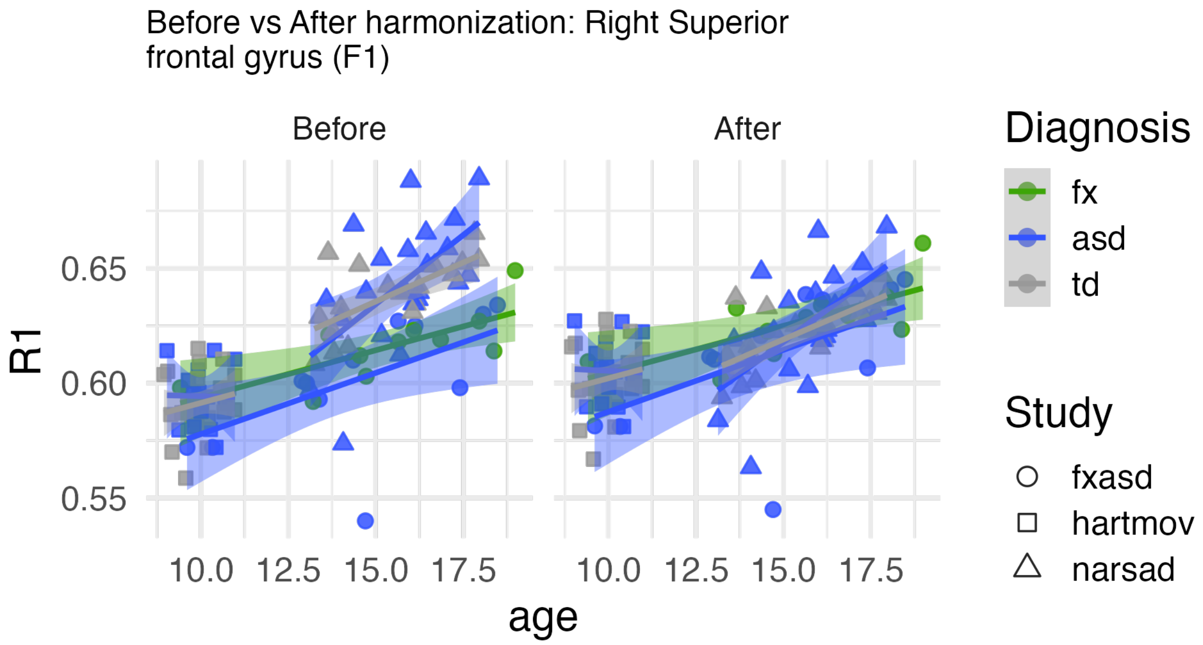

Before vs After harmonization: Right Superior frontal gyrus (F1)
Before
After
R1
0.65
0.60
0.55
10.0
12.5
15.0
17.5
age
Diagnosis
fx
asd
td
Study
fxasd
hartmov
narsad

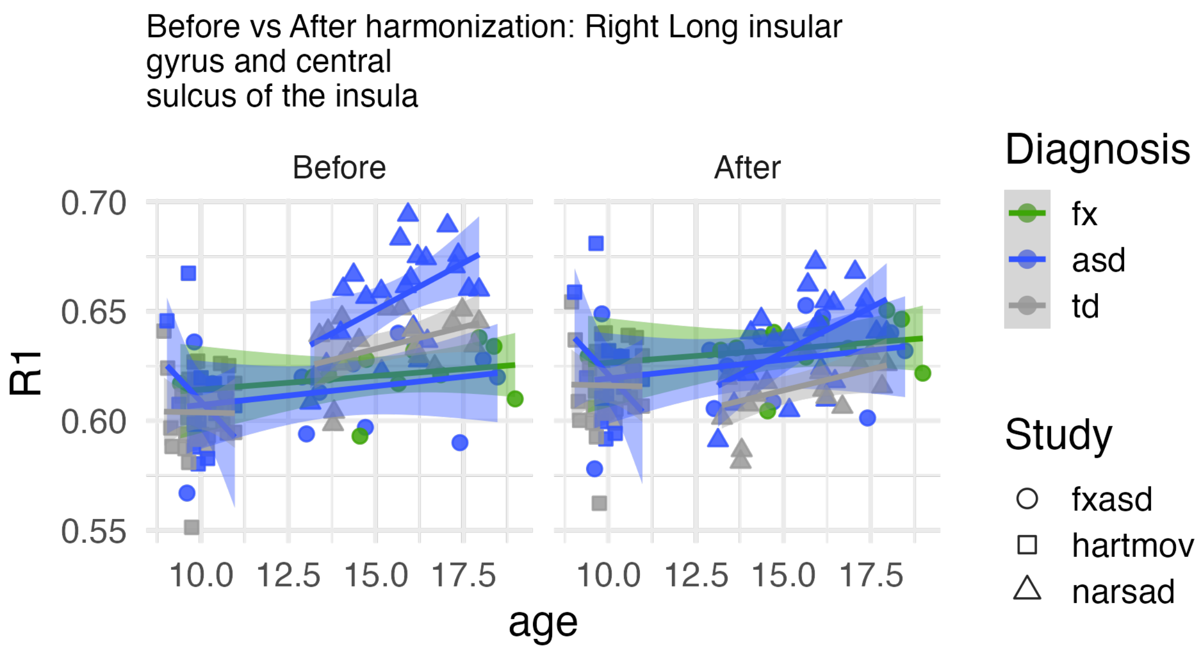

Before vs After harmonization: Right Long insular gyrus and central sulcus of the insula
Before
After
R1
0.70
0.65
0.60
0.55
10.0
12.5
15.0
17.5
age
Diagnosis
fx
asd
td
Study
fxasd
hartmov
narsad

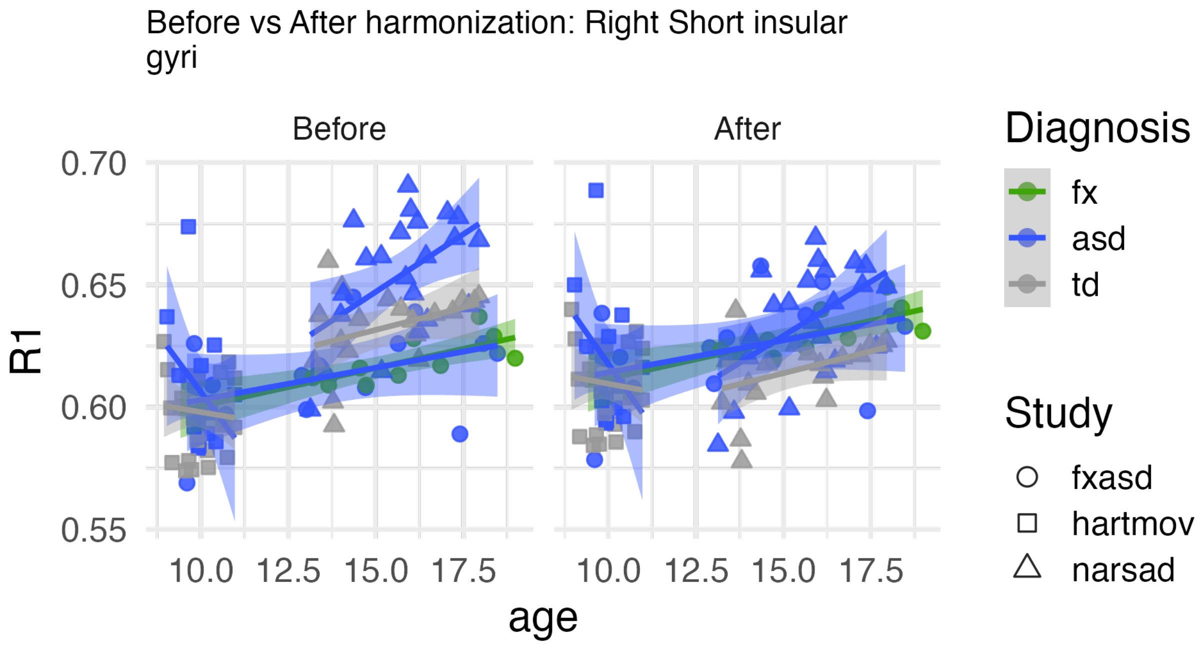

Before vs After harmonization: Right Short insular gyri
Before
After
R1
0.70
0.65
0.60
0.55
10.0
12.5
15.0
17.5
age
Diagnosis
fx
asd
td
Study
fxasd
hartmov
narsad

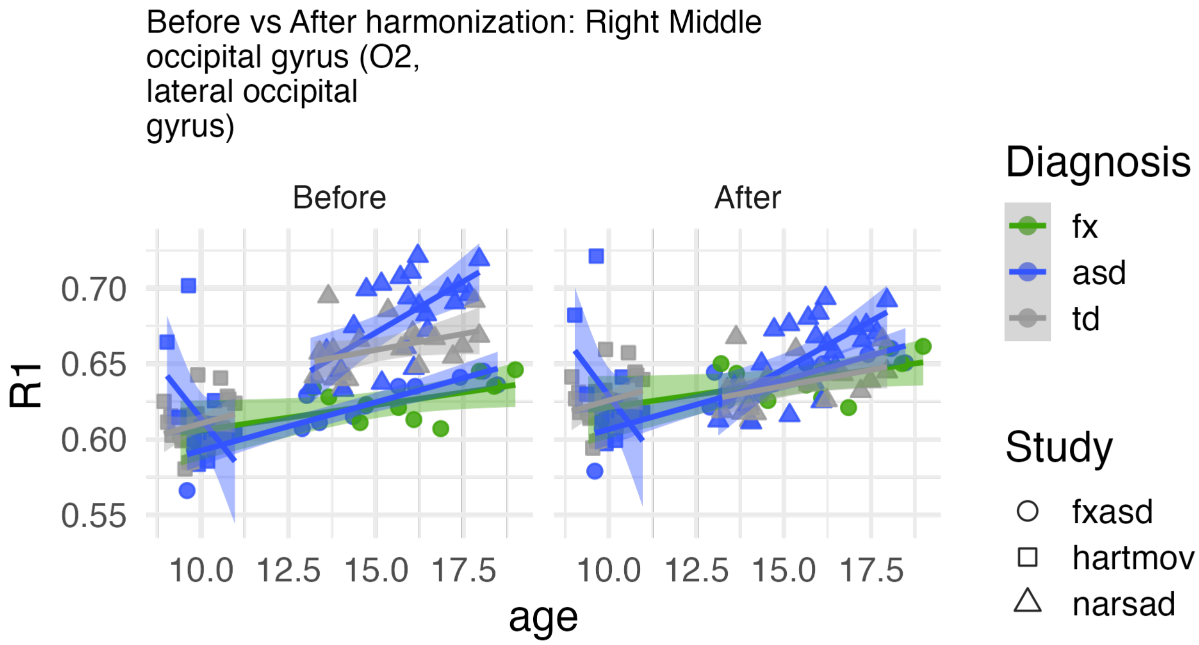
Before vs After harmonization: Right Middle occipital gyrus (O2, lateral occipital gyrus)
Before
After
R1
0.70
0.65
0.60
0.55
10.0
12.5
15.0
17.5
age
Diagnosis
fx
asd
td
Study
fxasd
hartmov
narsad

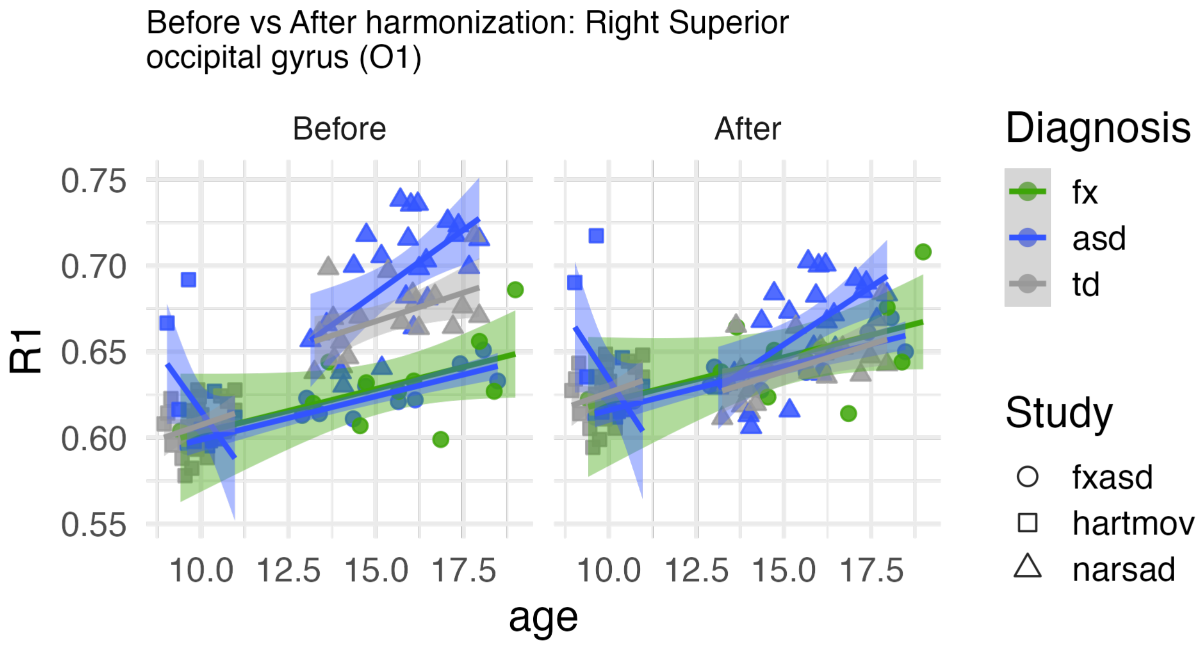
Before vs After harmonization: Right Superior occipital gyrus (O1)
Before
After
R1
0.75
0.70
0.65
0.60
0.55
10.0
12.5
15.0
17.5
age
Diagnosis
fx
asd
td
Study
fxasd
hartmov
narsad

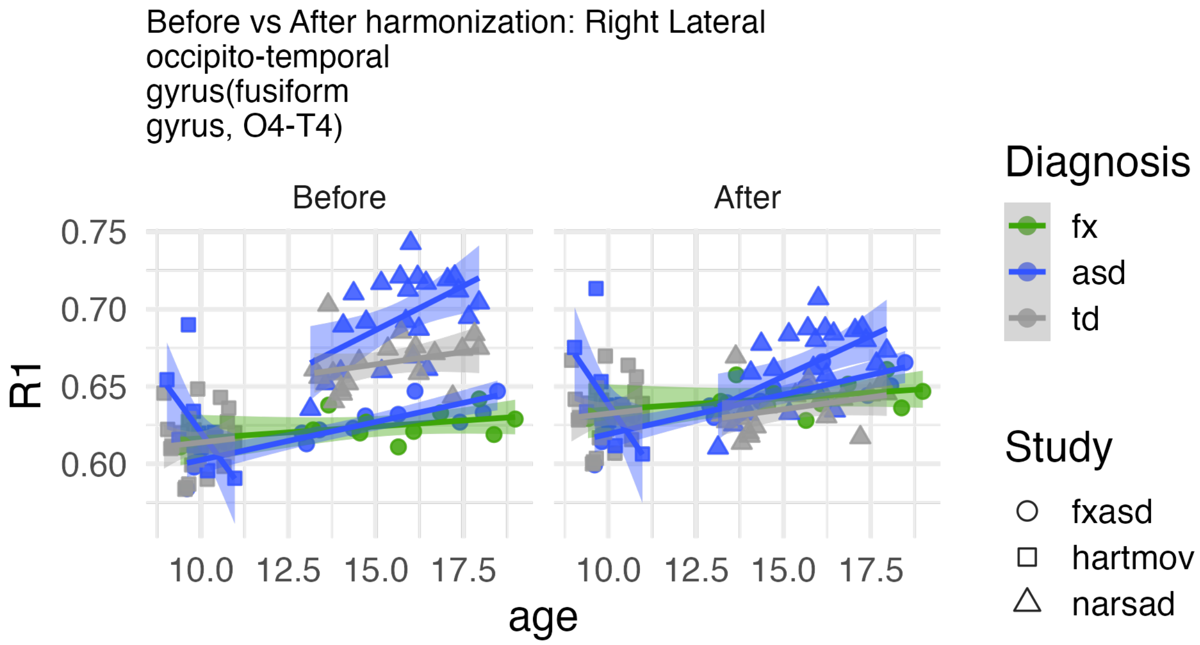
Before vs After harmonization: Right Lateral
occipito-temporal
gyrus(fusiform
gyrus, O4-T4)
Before
After
R1
0.75
0.70
0.65
0.60
10.0
12.5
15.0
17.5
age
Diagnosis
fx
asd
td
Study
fxasd
hartmov
narsad

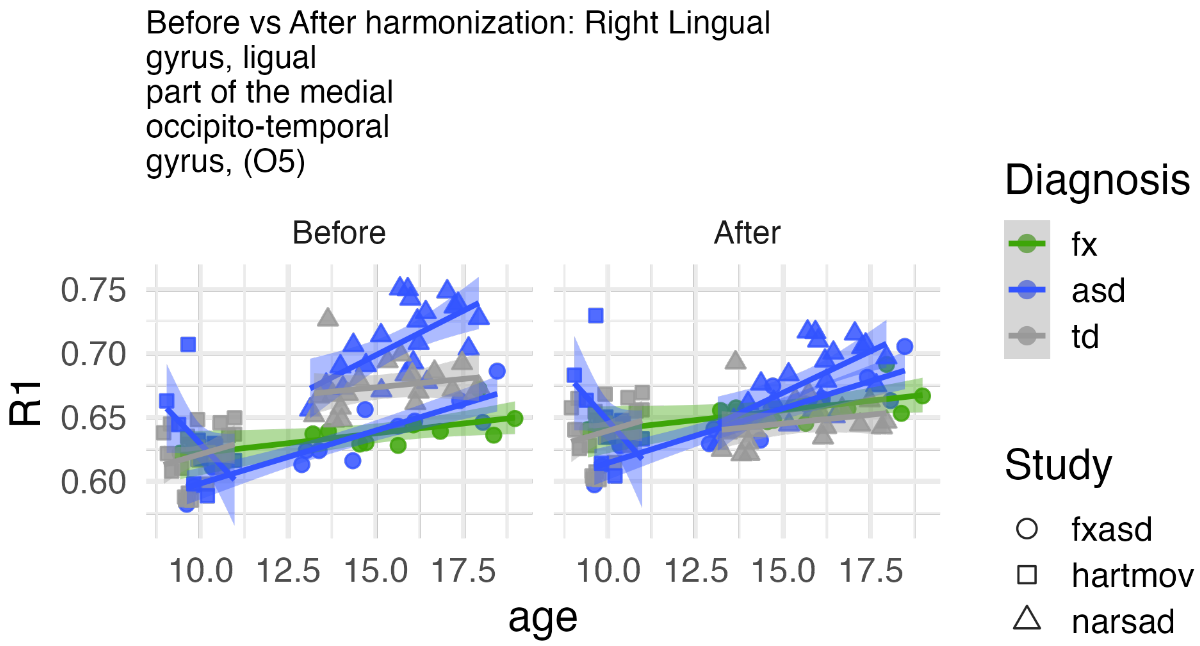
Before vs After harmonization: Right Lingual
gyrus, ligual
part of the medial
occipito-temporal
gyrus, (O5)
Before
After
R1
0.75
0.70
0.65
0.60
10.0
12.5
15.0
17.5
age
Diagnosis
fx
asd
td
Study
fxasd
hartmov
narsad

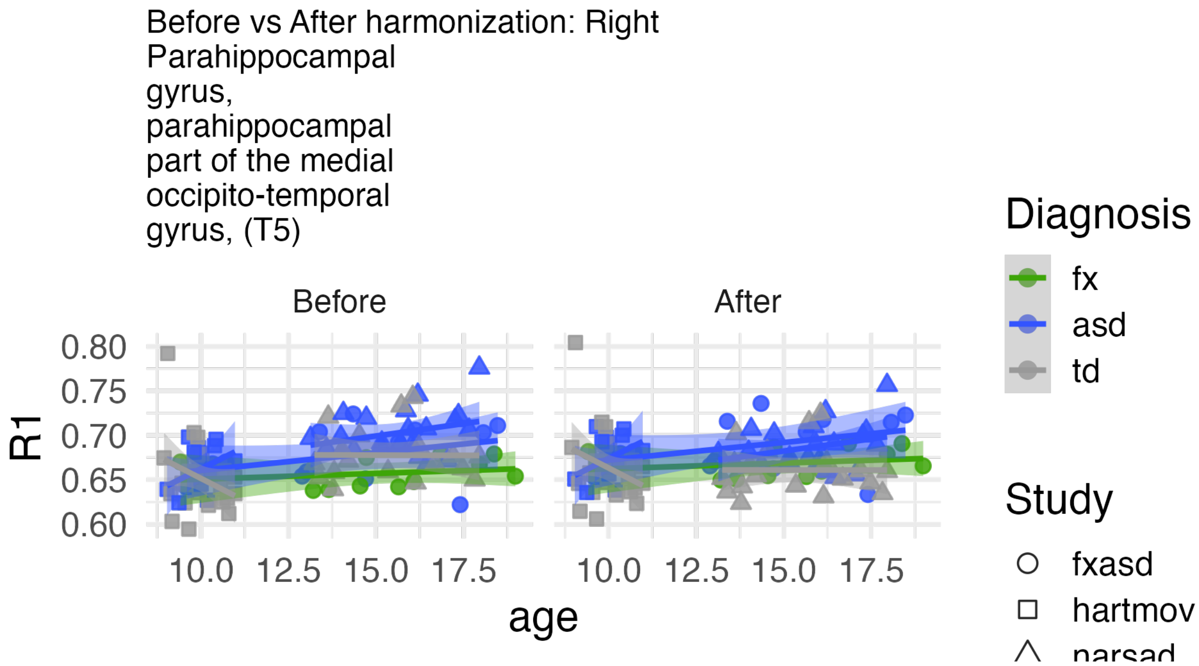

Before vs After harmonization: Right Parahippocampal gyrus, parahippocampal part of the medial occipito-temporal gyrus, (T5)
Before
After
R1
0.80
0.75
0.70
0.65
0.60
10.0
12.5
15.0
17.5
age
Diagnosis
fx
asd
td
Study
fxasd
hartmov

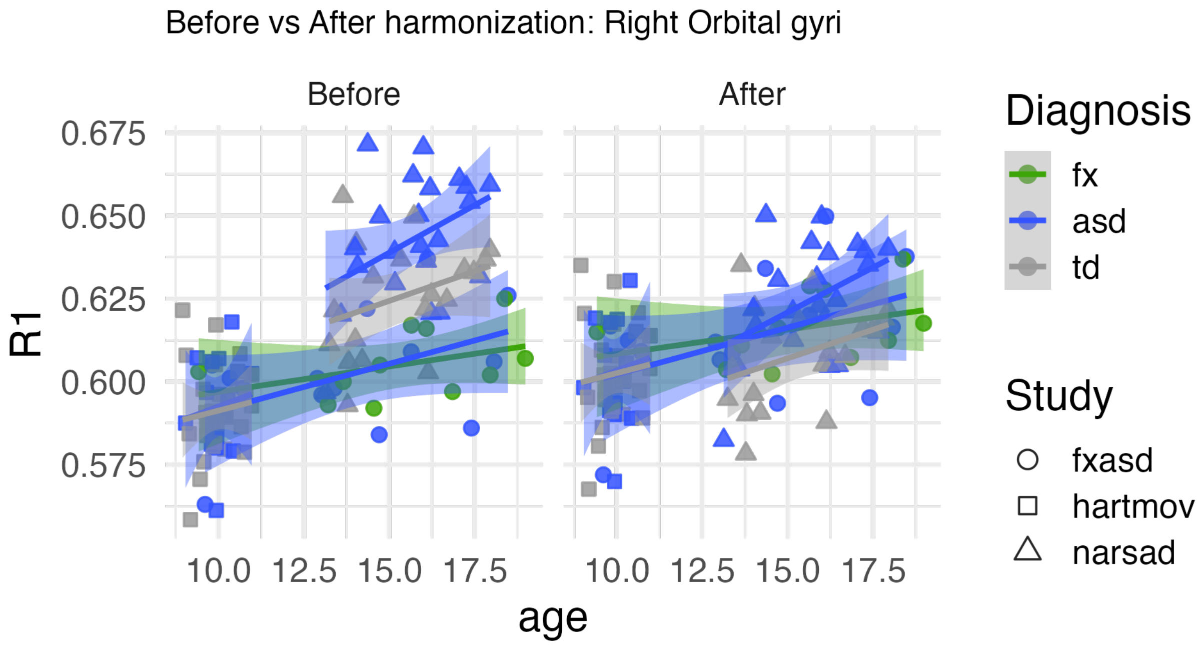

Before vs After harmonization: Right Orbital gyri
Before
After
R1
0.675
0.650
0.625
0.600
0.575
10.0
12.5
15.0
17.5
age
Diagnosis
fx
asd
td
Study
fxasd
hartmov
narsad

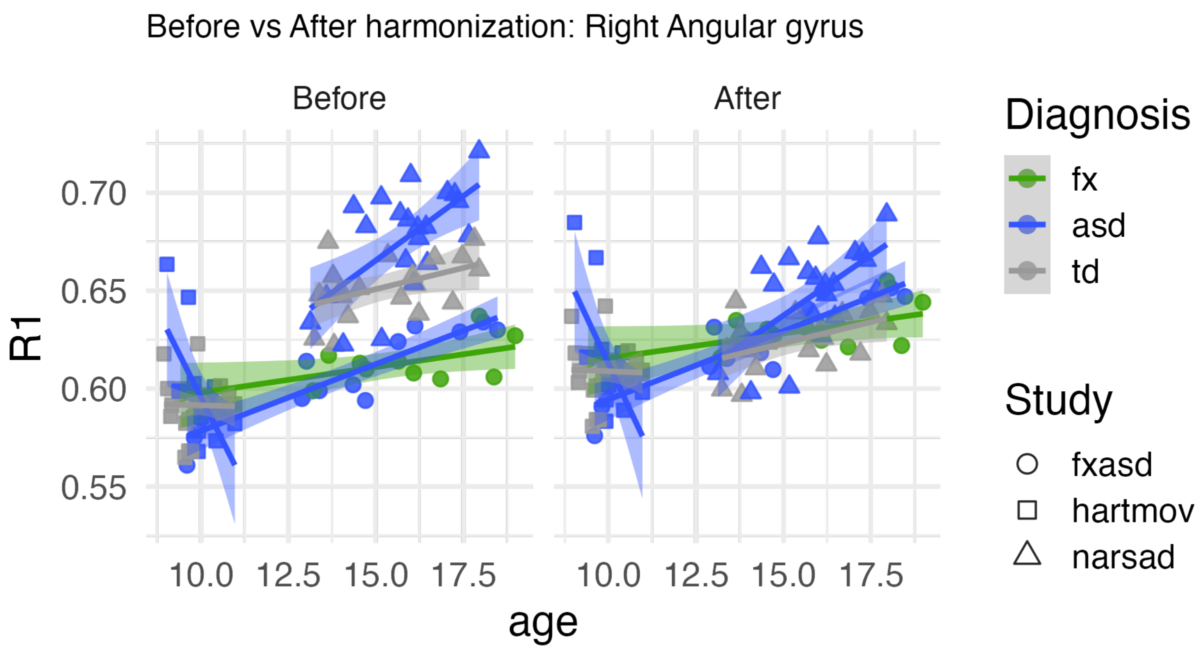

Before vs After harmonization: Right Angular gyrus
Before
After
R1
0.70
0.65
0.60
0.55
10.0
12.5
15.0
17.5
age
Diagnosis
fx
asd
td
Study
fxasd
hartmov
narsad

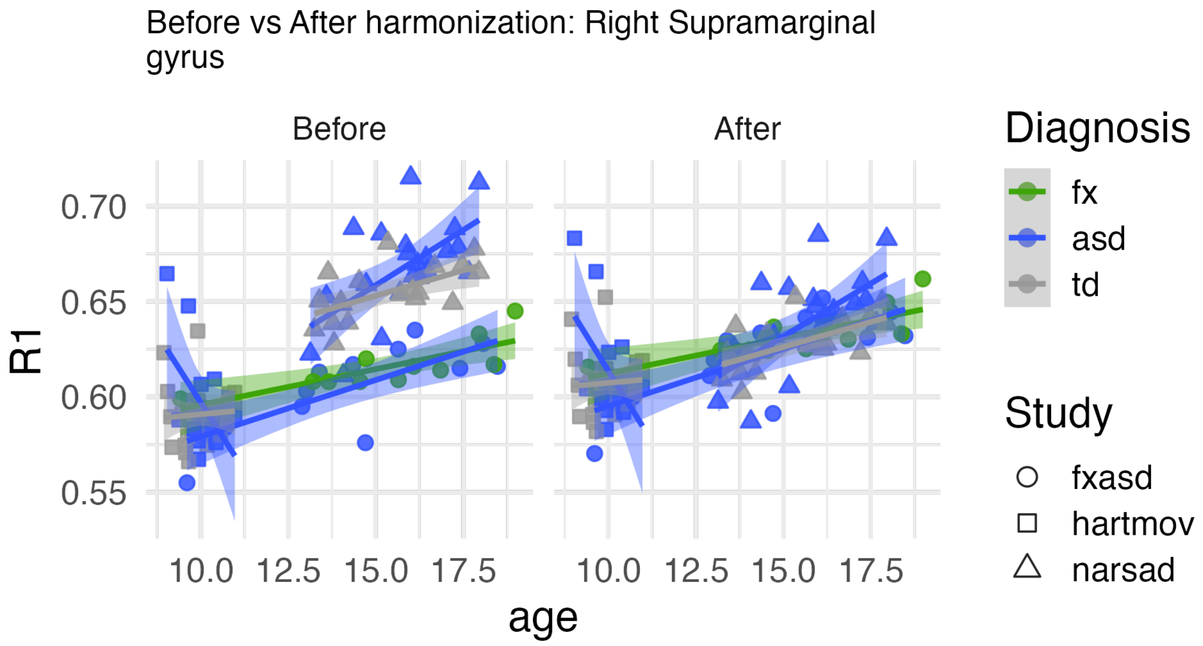

Before vs After harmonization: Right Supramarginal gyrus
Before
After
R1
0.70
0.65
0.60
0.55
10.0
12.5
15.0
17.5
age
Diagnosis
fx
asd
td
Study
fxasd
hartmov
narsad

Before vs After harmonization: Right Superior parietal lobule (lateral part of P1)

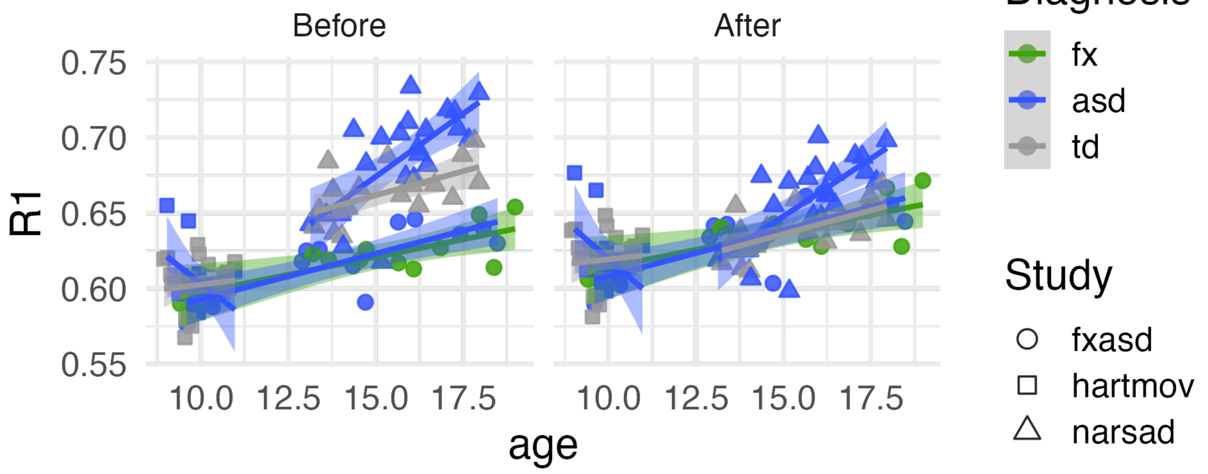

Before vs After harmonization: Right Postcentral gyrus

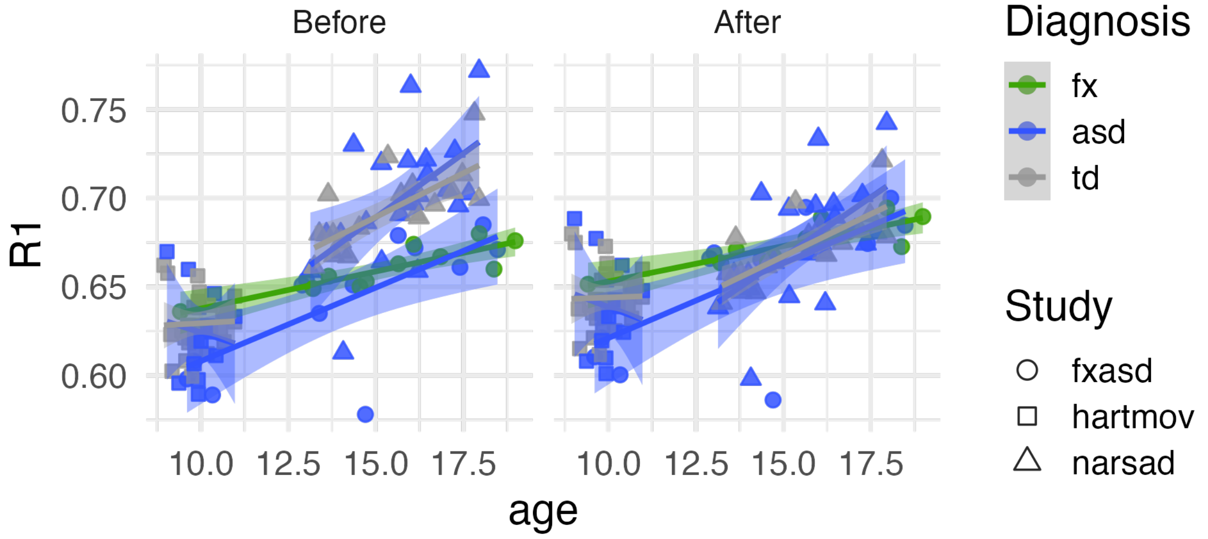

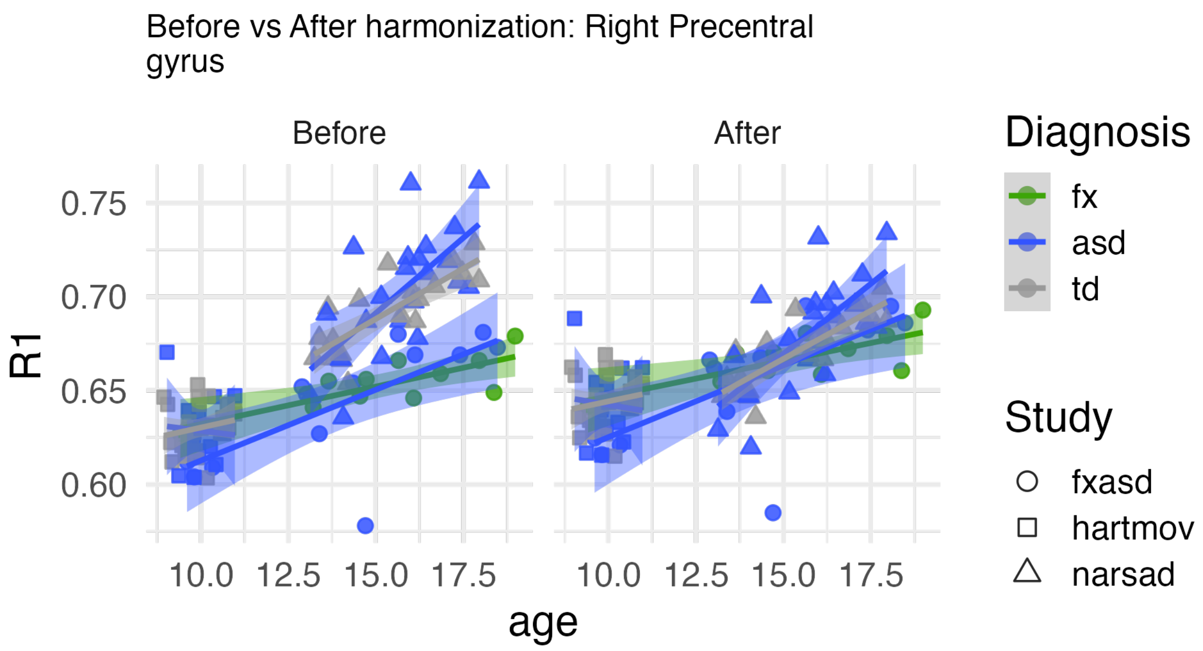
Before vs After harmonization: Right Precentral
gyrus
Before
After
R1
0.75
0.70
0.65
0.60
10.0 12.5 15.0 17.5
10.0 12.5 15.0 17.5
age
Diagnosis
fx
asd
td
Study
fxasd
hartmov
narsad

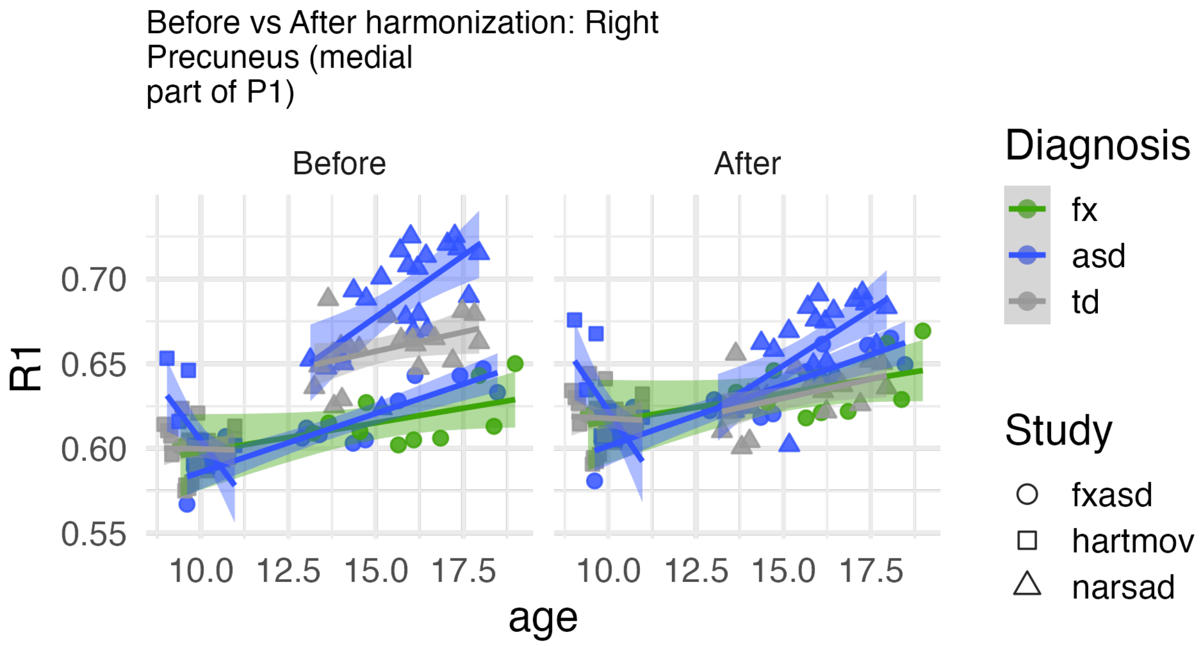
Before vs After harmonization: Right
Precuneus (medial
part of P1)
Before
After
R1
0.70
0.65
0.60
0.55
10.0 12.5 15.0 17.5
10.0 12.5 15.0 17.5
age
Diagnosis
fx
asd
td
Study
fxasd
hartmov
narsad

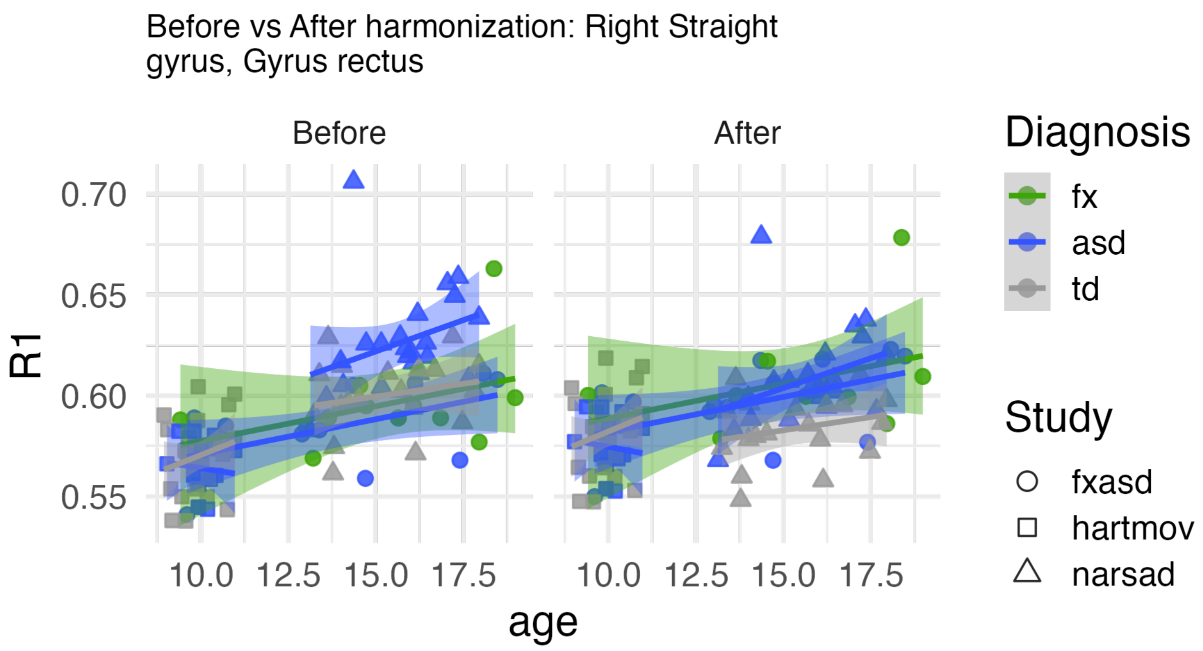

Before vs After harmonization: Right Straight gyrus, Gyrus rectus
Before
After
R1
0.70
0.65
0.60
0.55
10.0
12.5
15.0
17.5
age
Diagnosis
fx
asd
td
Study
fxasd
hartmov
narsad

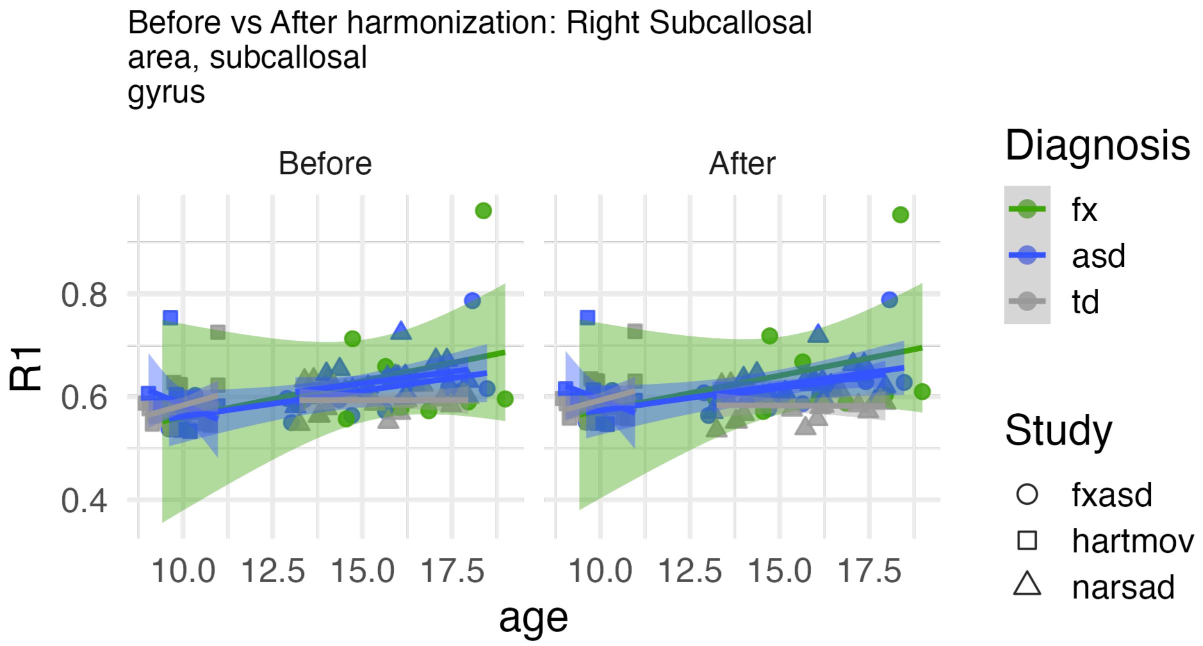

Before vs After harmonization: Right Subcallosal area, subcallosal gyrus
Before
After
R1
0.8
0.6
0.4
10.0
12.5
15.0
17.5
age
Diagnosis
fx
asd
td
Study
fxasd
hartmov
narsad

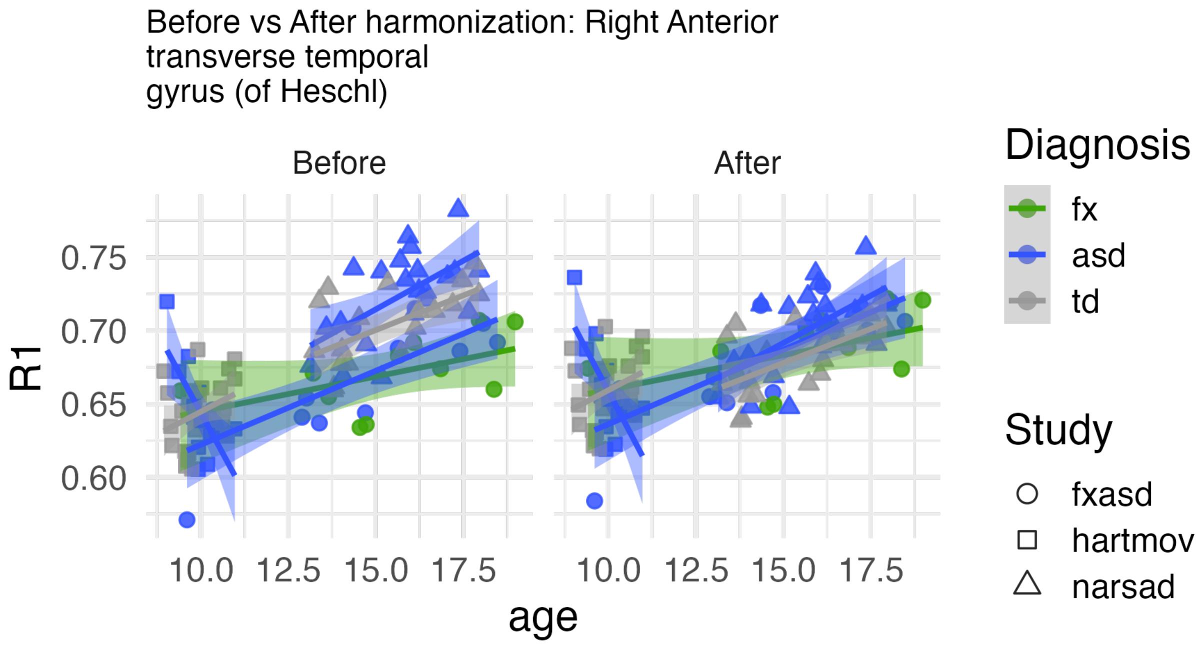
Before vs After harmonization: Right Anterior
transverse temporal
gyrus (of Heschl)
Before
After
R1
0.75
0.70
0.65
0.60
10.0
12.5
15.0
17.5
age
Diagnosis
fx
asd
td
Study
fxasd
hartmov
narsad

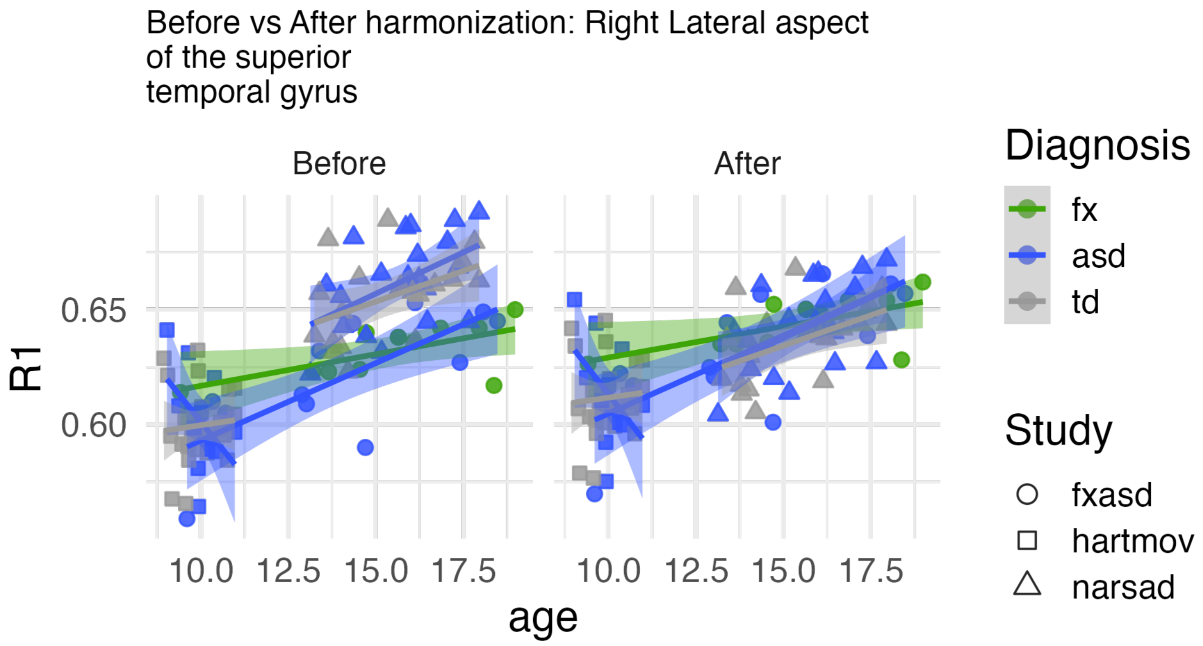
Before vs After harmonization: Right Lateral aspect
of the superior
temporal gyrus
Before
After
R1
0.65
0.60
10.0
12.5
15.0
17.5
age
Diagnosis
fx
asd
td
Study
fxasd
hartmov
narsad

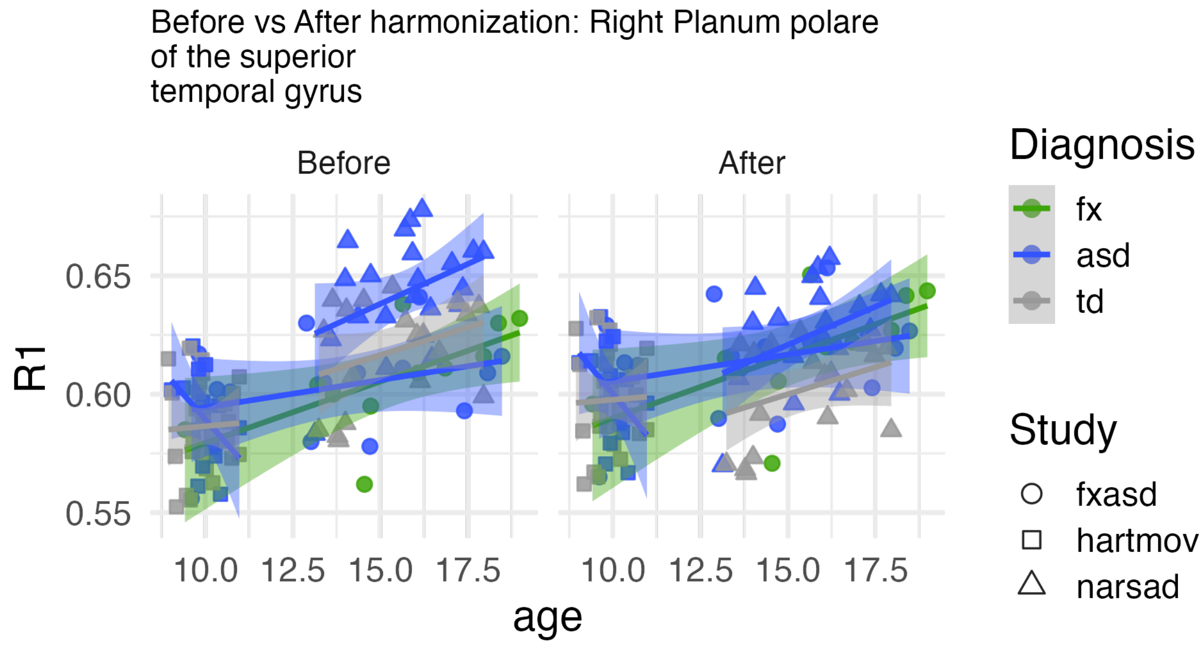
Before vs After harmonization: Right Planum polare
of the superior
temporal gyrus
Before
After
R1
0.65
0.60
0.55
10.0 12.5 15.0 17.5
10.0 12.5 15.0 17.5
age
Diagnosis
fx
asd
td
Study
fxasd
hartmov
narsad

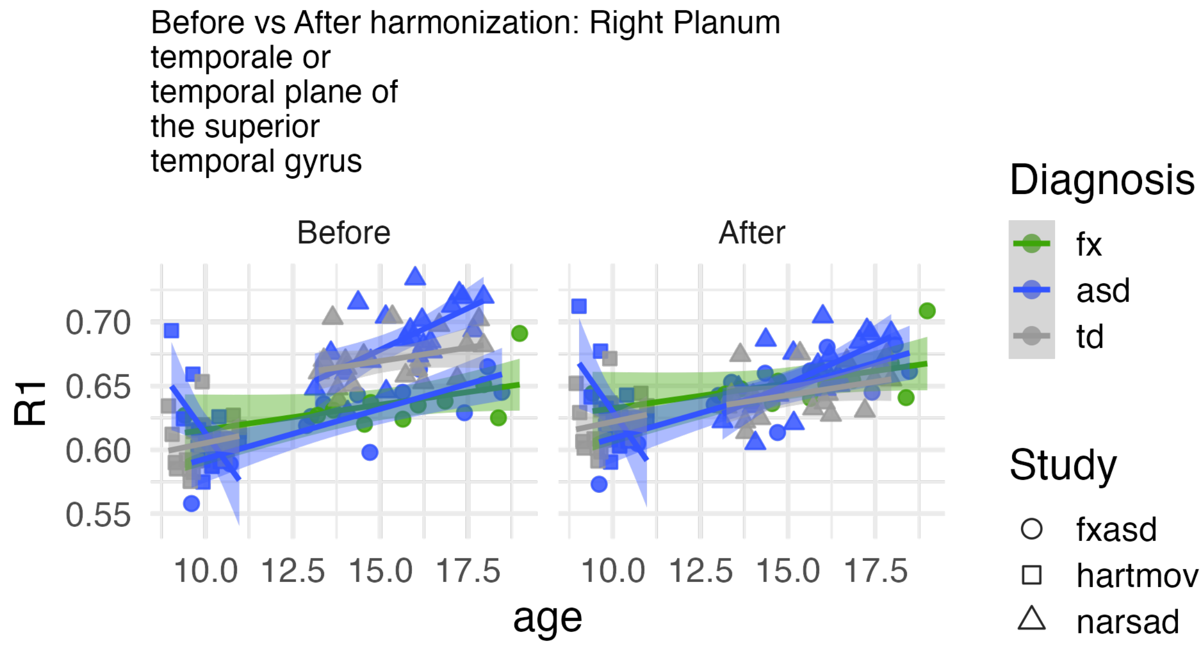
Before vs After harmonization: Right Planum
temporale or
temporal plane of
the superior
temporal gyrus
Before
After
R1
0.70
0.65
0.60
0.55
10.0 12.5 15.0 17.5
10.0 12.5 15.0 17.5
age
Diagnosis
fx
asd
td
Study
fxasd
hartmov
narsad

Before vs After harmonization: Right Inferior temporal gyrus (T3)

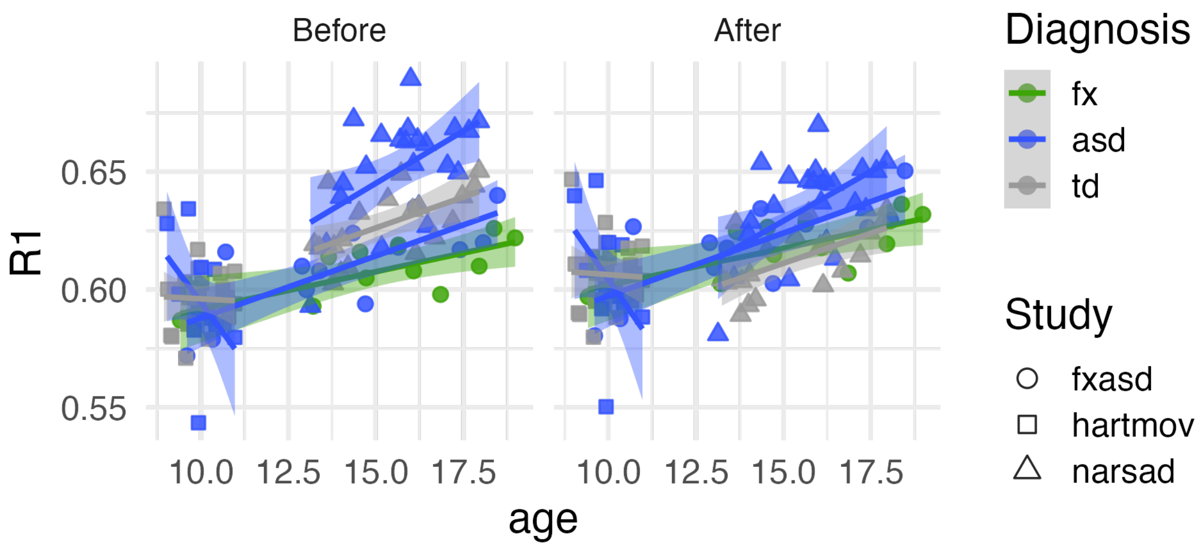

Before vs After harmonization: Right Middle temporal gyrus (T2)

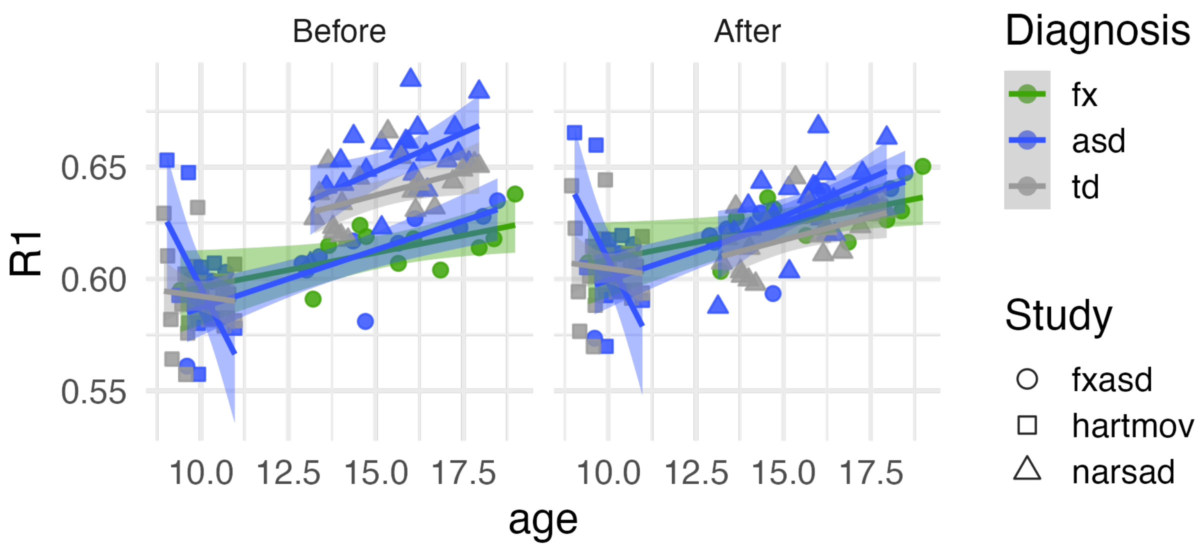

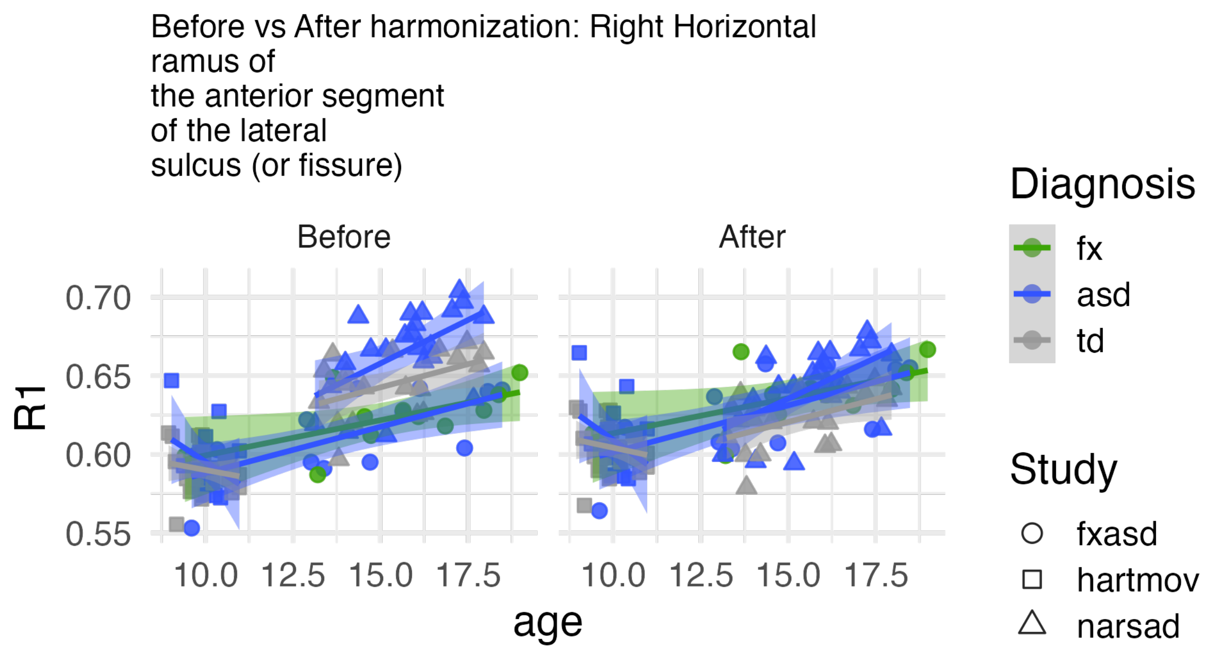
Before vs After harmonization: Right Horizontal
ramus of
the anterior segment
of the lateral
sulcus (or fissure)
Before
After
0.70
0.65
0.60
0.55
R1
10.0
12.5
15.0
17.5
age
Diagnosis
fx
asd
td
Study
fxasd
hartmov
narsad

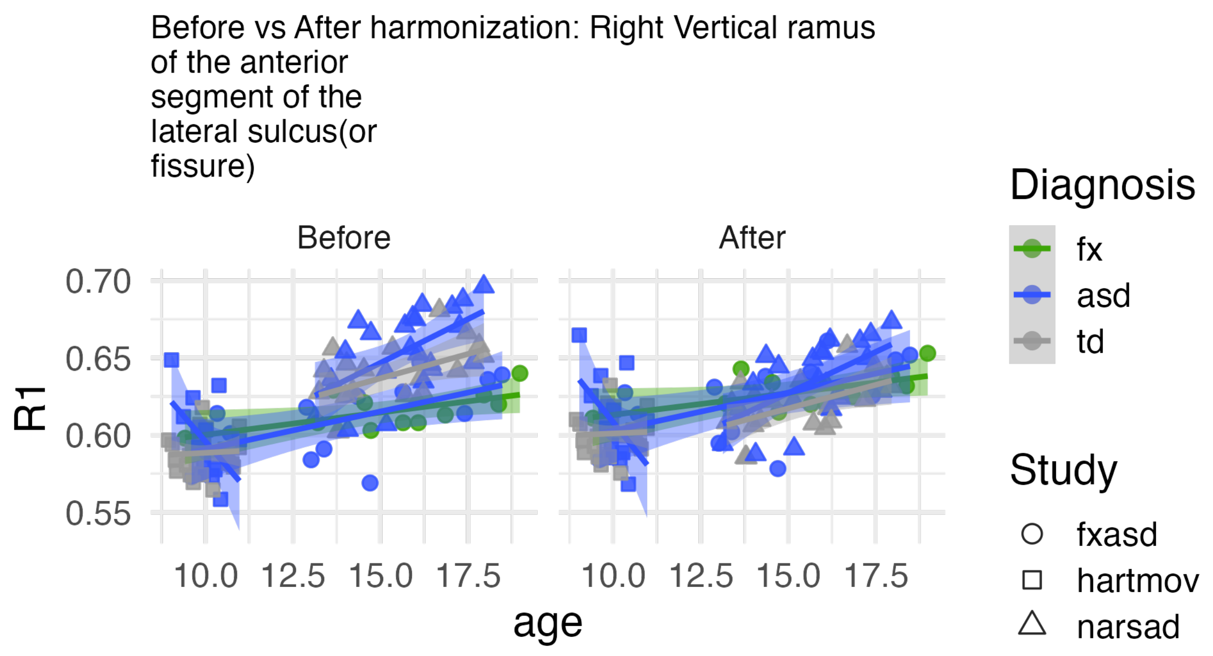
Before vs After harmonization: Right Vertical ramus
of the anterior
segment of the
lateral sulcus(or
fissure)
Before
After
0.70
0.65
0.60
0.55
R1
10.0
12.5
15.0
17.5
age
Diagnosis
fx
asd
td
Study
fxasd
hartmov
narsad

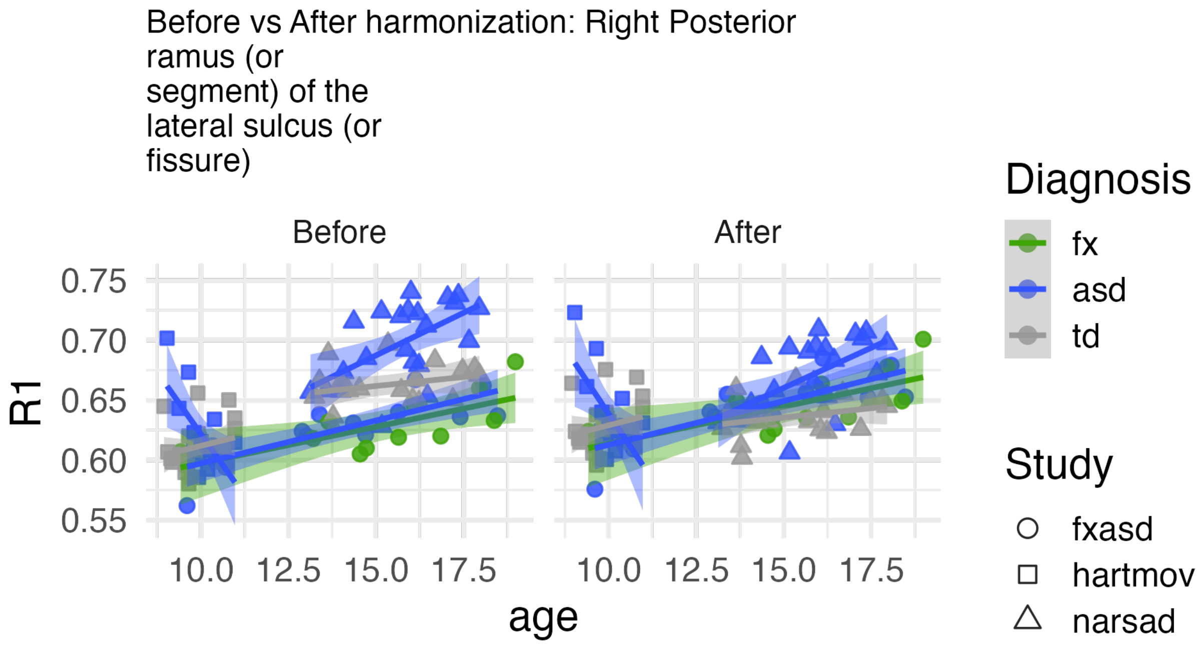

Before vs After harmonization: Right Posterior
ramus (or
segment) of the
lateral sulcus (or
fissure)
Before
After
R1
0.75
0.70
0.65
0.60
0.55
10.0
12.5
15.0
17.5
age
Diagnosis
fx
asd
td
Study
fxasd
hartmov
narsad

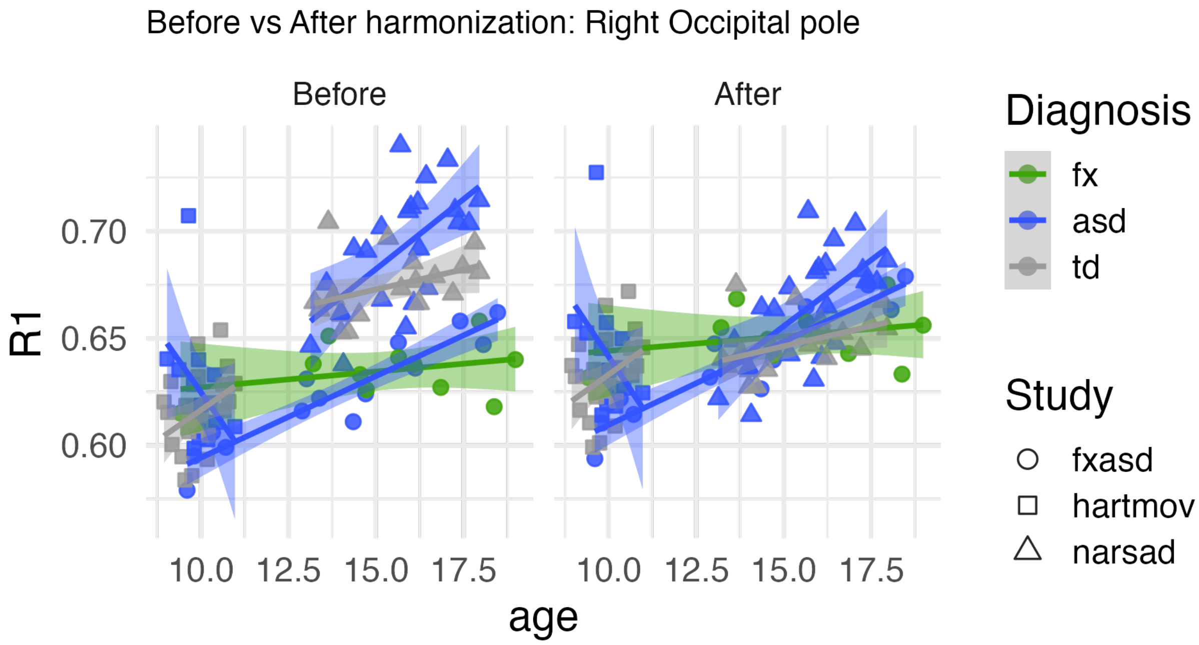

Before vs After harmonization: Right Occipital pole
Before
After
R1
0.70
0.65
0.60
10.0
12.5
15.0
17.5
age
Diagnosis
fx
asd
td
Study
fxasd
hartmov
narsad

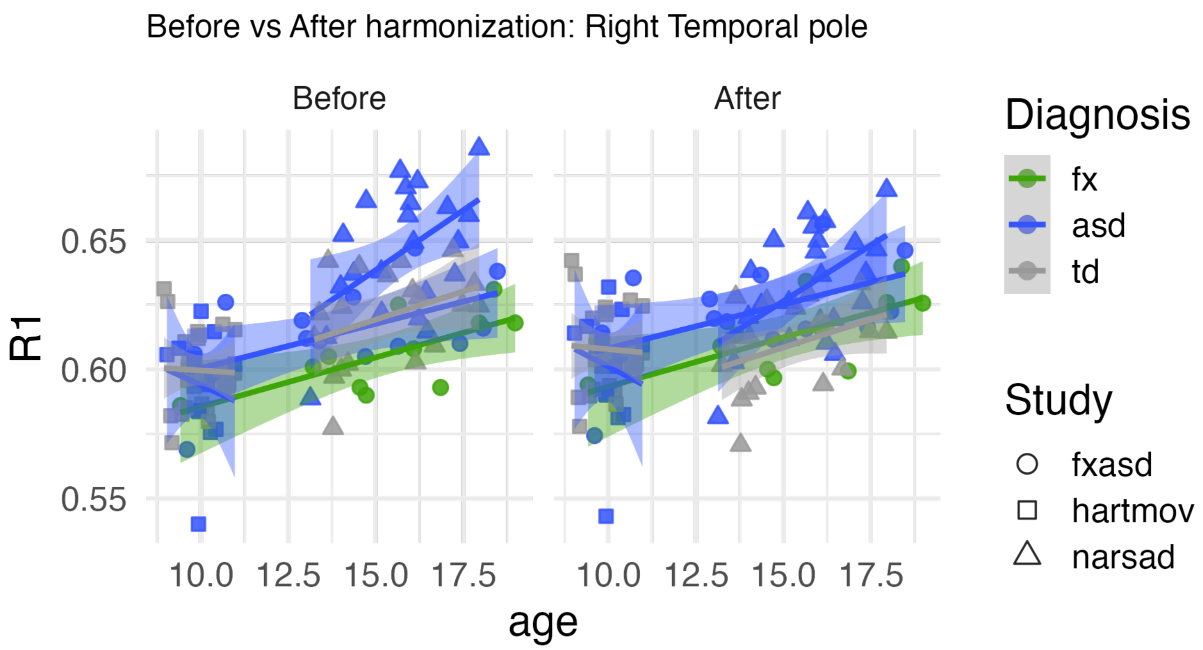

Before vs After harmonization: Right Temporal pole
Before
After
R1
0.65
0.60
0.55
10.0 12.5 15.0 17.5
10.0 12.5 15.0 17.5
age
Diagnosis
fx
asd
td
Study
fxasd
hartmov
narsad

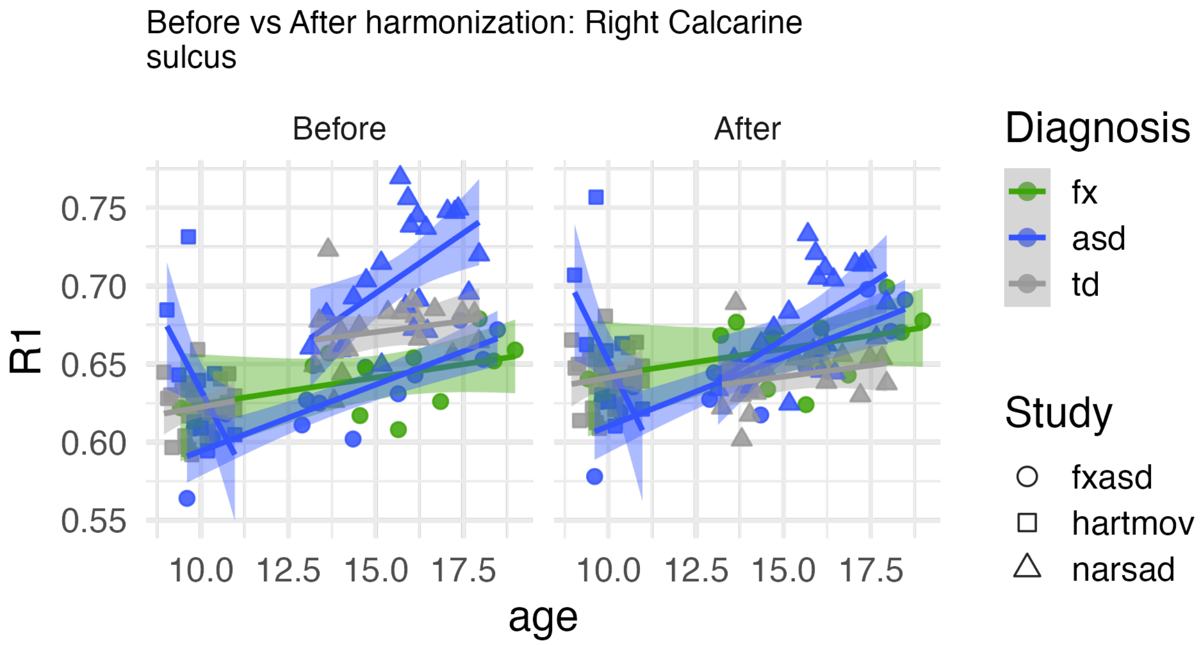

Before vs After harmonization: Right Calcarine sulcus
Before
After
R1
0.75
0.70
0.65
0.60
0.55
10.0 12.5 15.0 17.5
10.0 12.5 15.0 17.5
age
Diagnosis
fx
asd
td
Study
fxasd
hartmov
narsad

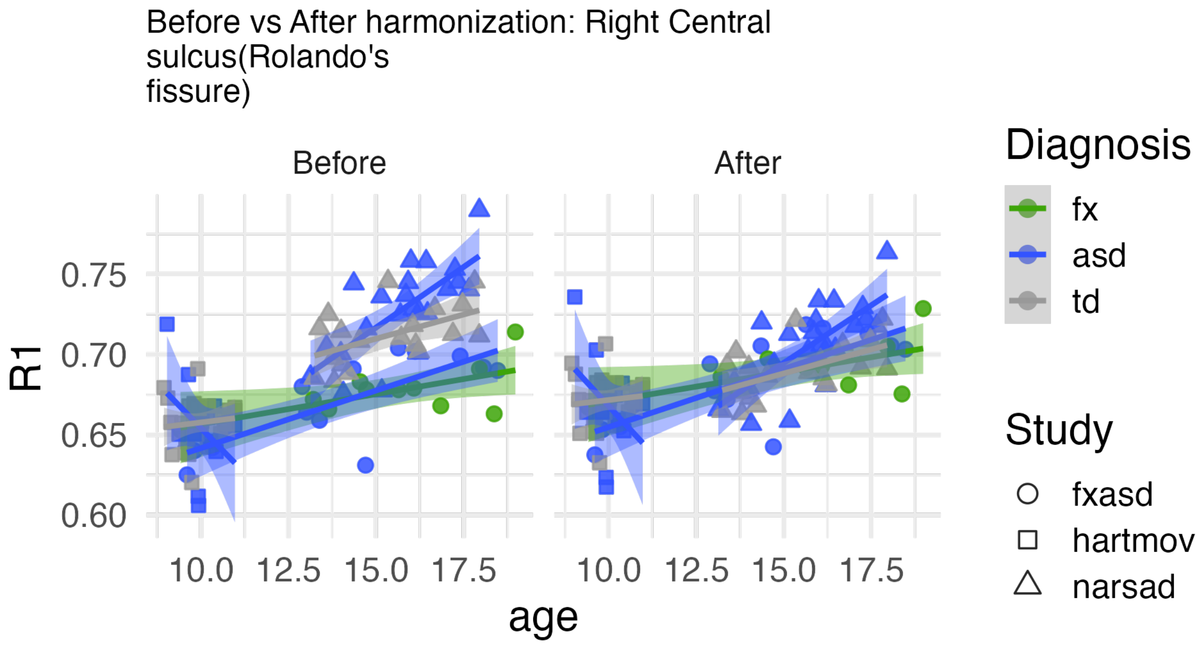
Before vs After harmonization: Right Central
sulcus(Rolando's
fissure)
Before
After
R1
0.75
0.70
0.65
0.60
10.0
12.5
15.0
17.5
age
Diagnosis
fx
asd
td
Study
fxasd
hartmov
narsad

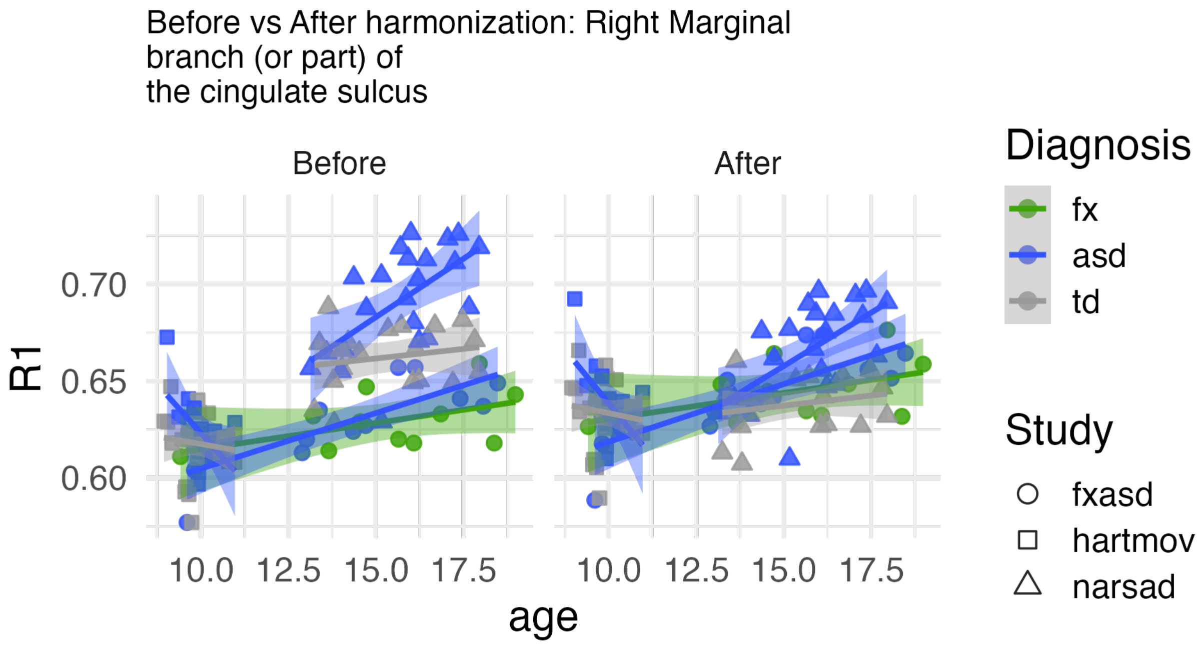
Before vs After harmonization: Right Marginal
branch (or part) of
the cingulate sulcus
Before
After
R1
0.70
0.65
0.60
10.0
12.5
15.0
17.5
age
Diagnosis
fx
asd
td
Study
fxasd
hartmov
narsad

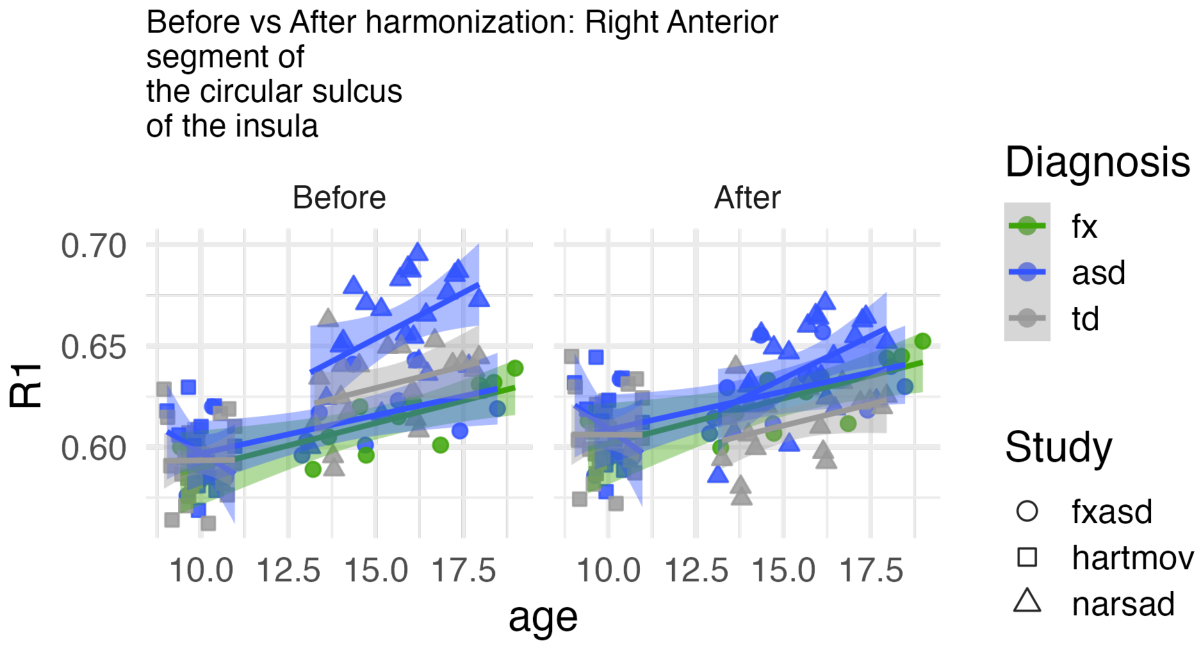
Before vs After harmonization: Right Anterior
segment of
the circular sulcus
of the insula
Before
After
0.70
0.65
0.60
R1
10.0 12.5 15.0 17.5
10.0 12.5 15.0 17.5
age
Diagnosis
fx
asd
td
Study
fxasd
hartmov
narsad

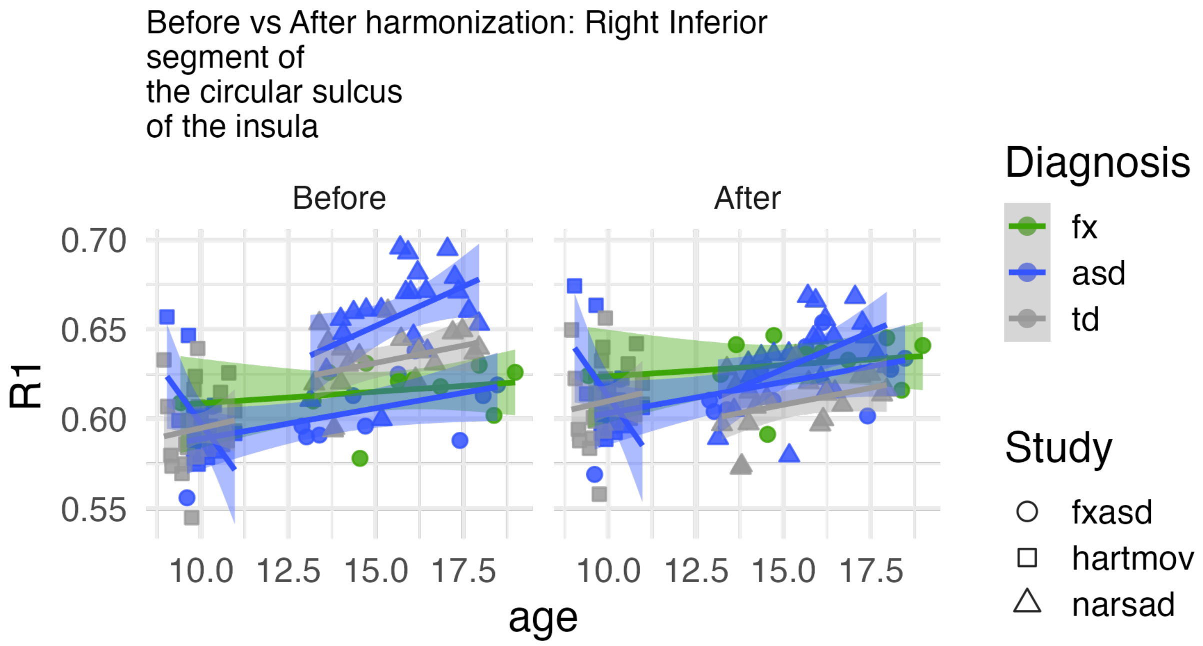
Before vs After harmonization: Right Inferior
segment of
the circular sulcus
of the insula
Before
After
0.70
0.65
0.60
0.55
R1
10.0 12.5 15.0 17.5
10.0 12.5 15.0 17.5
age
Diagnosis
fx
asd
td
Study
fxasd
hartmov
narsad

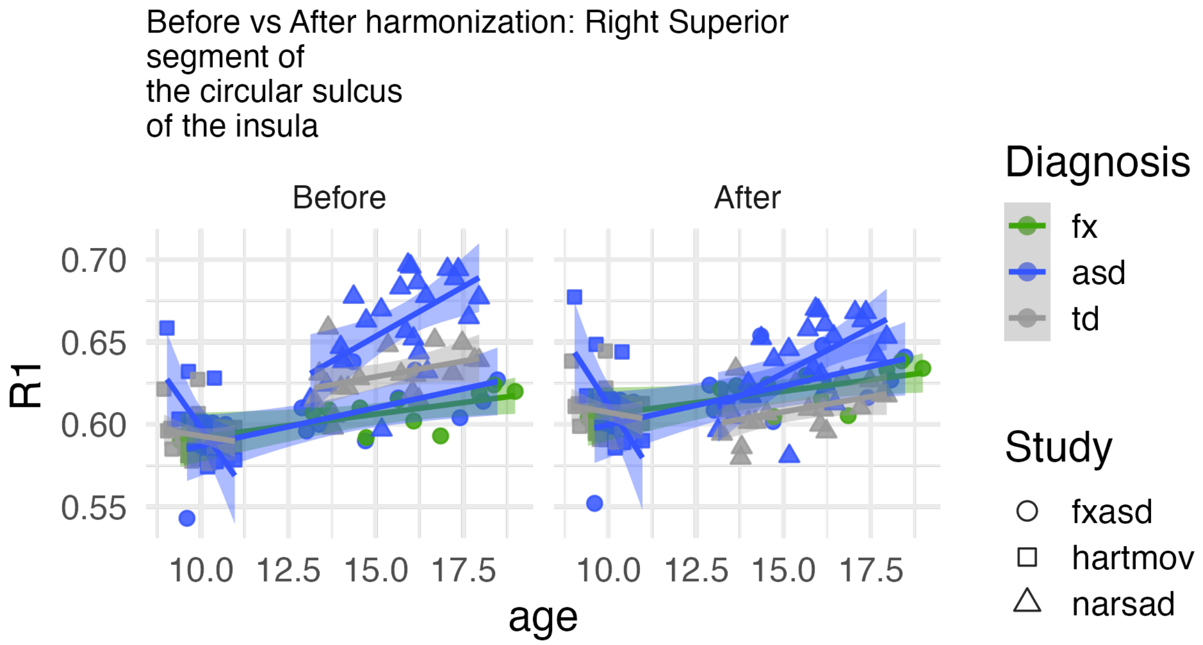
Before vs After harmonization: Right Superior
segment of
the circular sulcus
of the insula
Before
After
R1
0.70
0.65
0.60
0.55
10.0
12.5
15.0
17.5
age
Diagnosis
fx
asd
td
Study
fxasd
hartmov
narsad

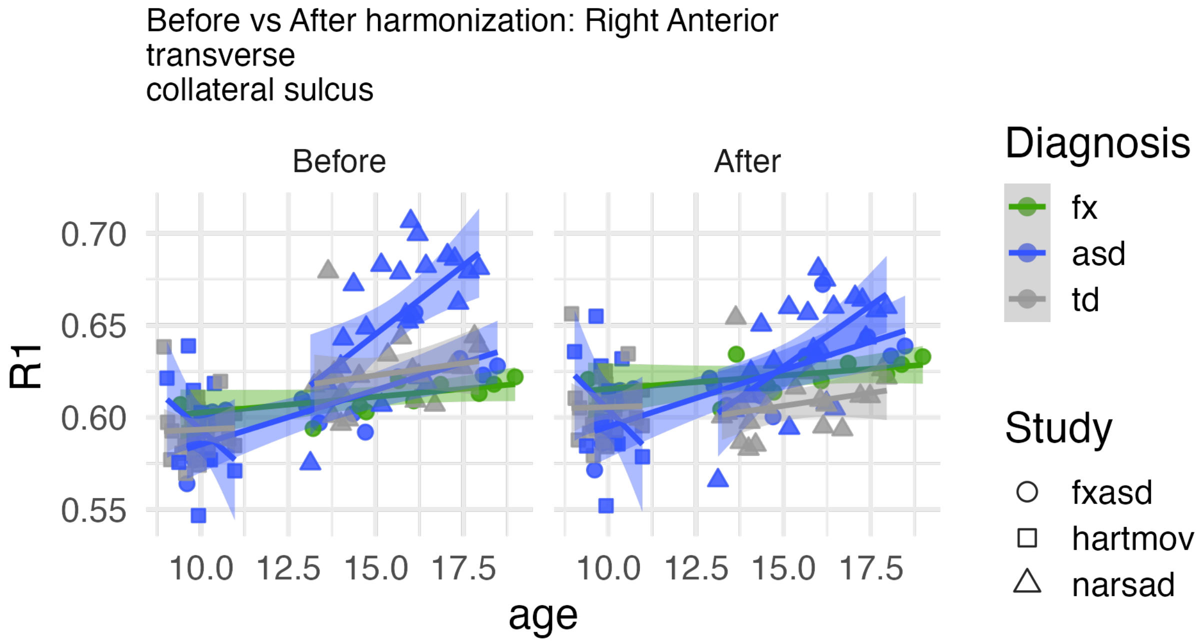
Before vs After harmonization: Right Anterior
transverse
collateral sulcus
Before
After
R1
0.70
0.65
0.60
0.55
10.0
12.5
15.0
17.5
age
Diagnosis
fx
asd
td
Study
fxasd
hartmov
narsad

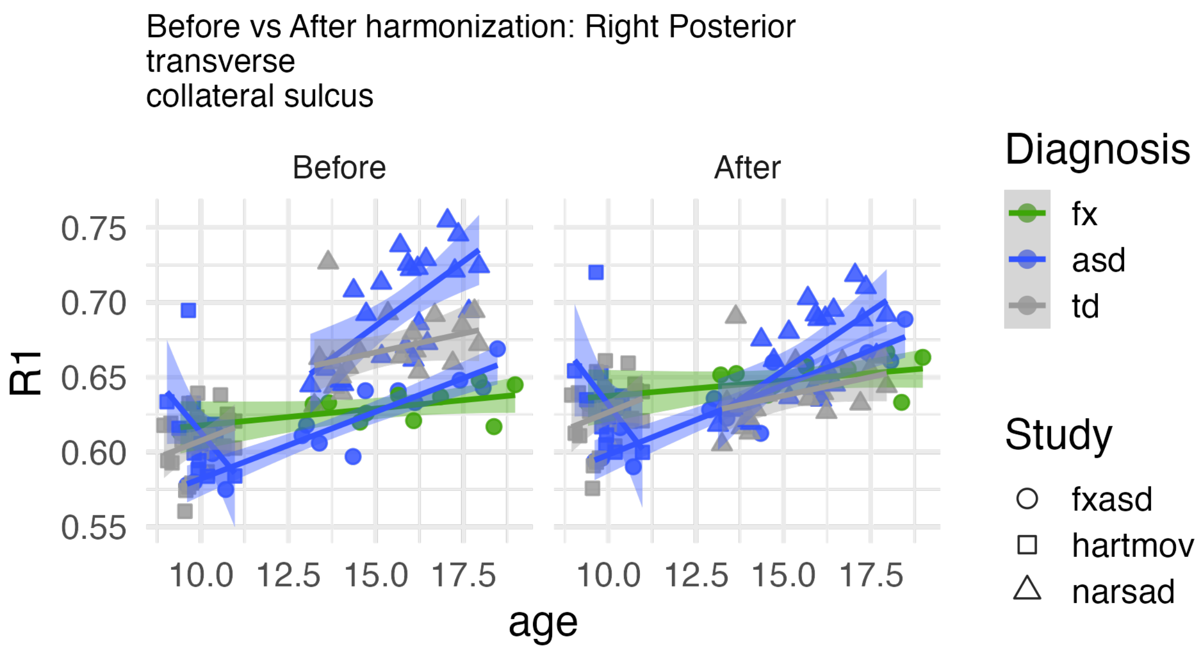
Before vs After harmonization: Right Posterior transverse
collateral sulcus
Before
After
R1
0.75
0.70
0.65
0.60
0.55
10.0 12.5 15.0 17.5
10.0 12.5 15.0 17.5
age
Diagnosis
fx
asd
td
Study
fxasd
hartmov
narsad

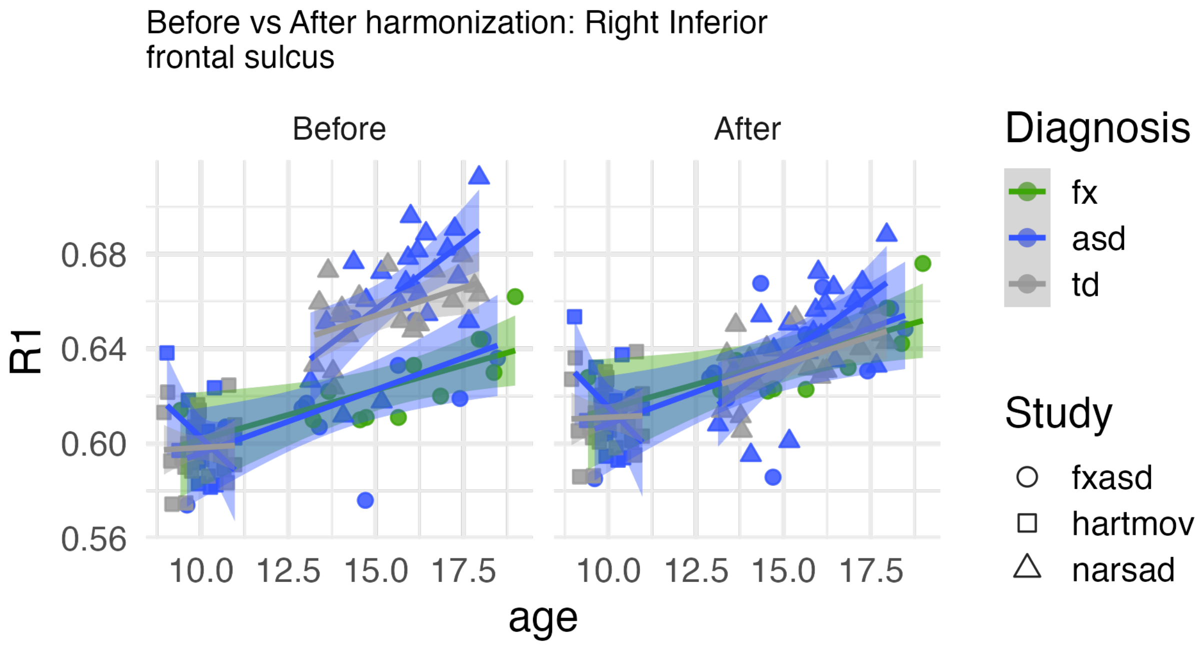
Before vs After harmonization: Right Inferior
frontal sulcus
Before
After
R1
0.68
0.64
0.60
0.56
10.0 12.5 15.0 17.5
10.0 12.5 15.0 17.5
age
Diagnosis
fx
asd
td
Study
fxasd
hartmov
narsad

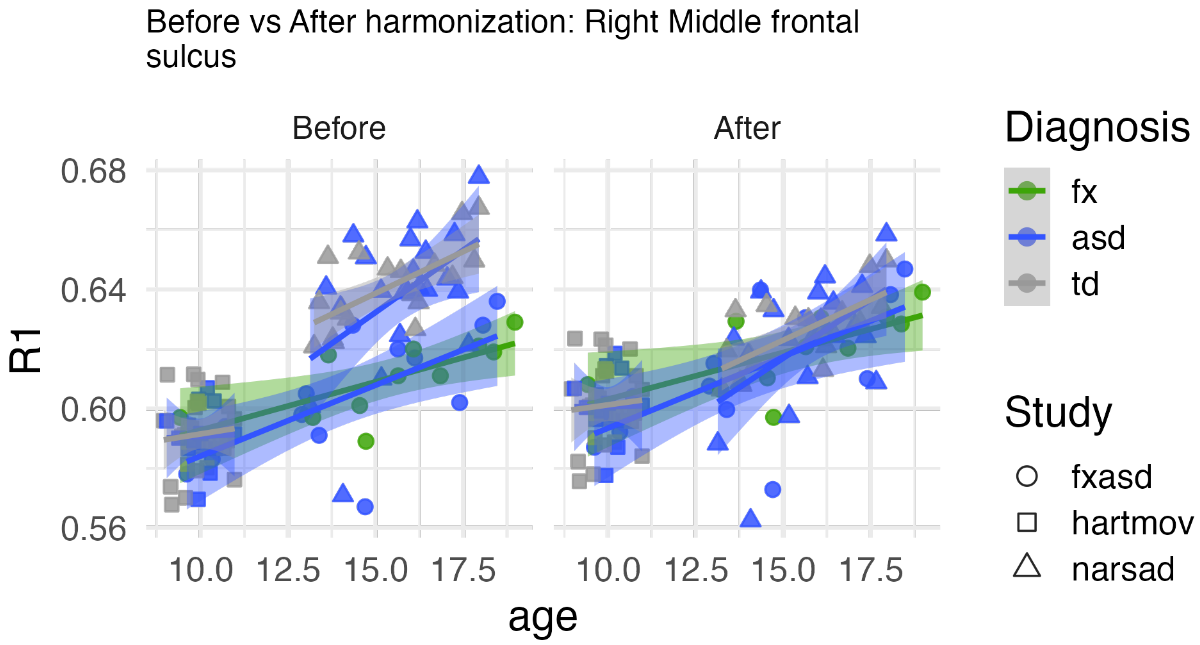
Before vs After harmonization: Right Middle frontal sulcus
Before
After
R1
0.68
0.64
0.60
0.56
10.0
12.5
15.0
17.5
age
Diagnosis
fx
asd
td
Study
fxasd
hartmov
narsad

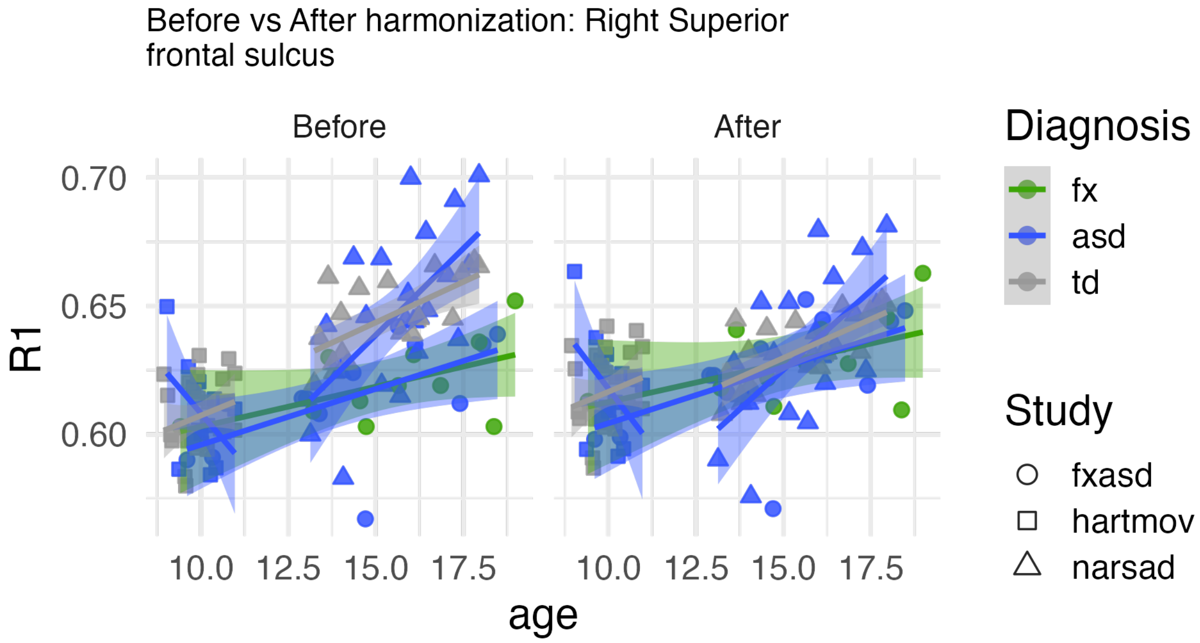
Before vs After harmonization: Right Superior frontal sulcus
Before
After
R1
0.70
0.65
0.60
10.0
12.5
15.0
17.5
age
Diagnosis
fx
asd
td
Study
fxasd
hartmov
narsad

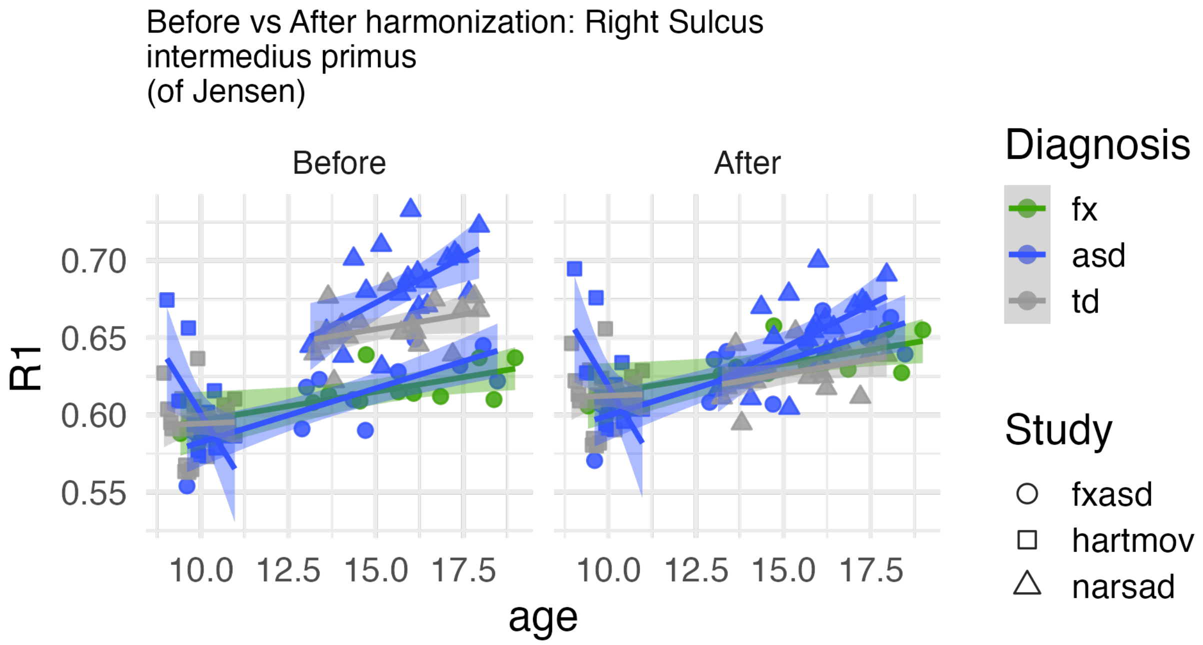
Before vs After harmonization: Right Sulcus
intermedius primus
(of Jensen)
Before
After
0.70
0.65
0.60
0.55
R1
10.0
12.5
15.0
17.5
age
Diagnosis
fx
asd
td
Study
fxasd
hartmov
narsad

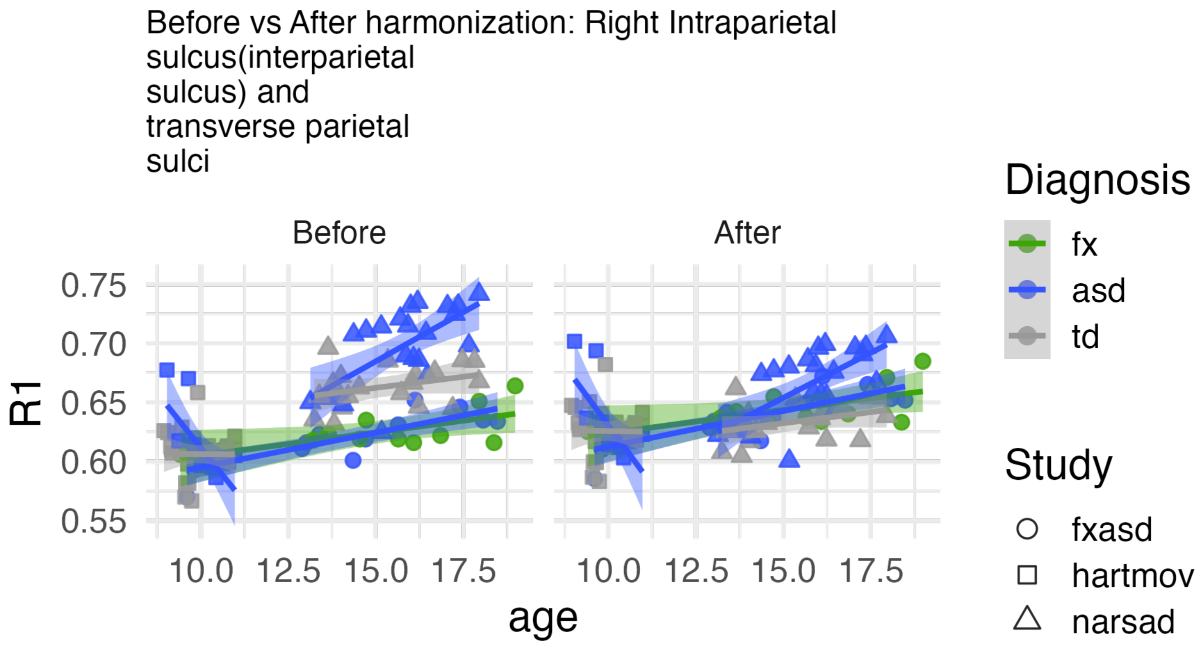
Before vs After harmonization: Right Intraparietal
sulcus(interparietal
sulcus) and
transverse parietal
sulci
Before
After
0.75
0.70
0.65
0.60
0.55
R1
10.0
12.5
15.0
17.5
age
Diagnosis
fx
asd
td
Study
fxasd
hartmov
narsad

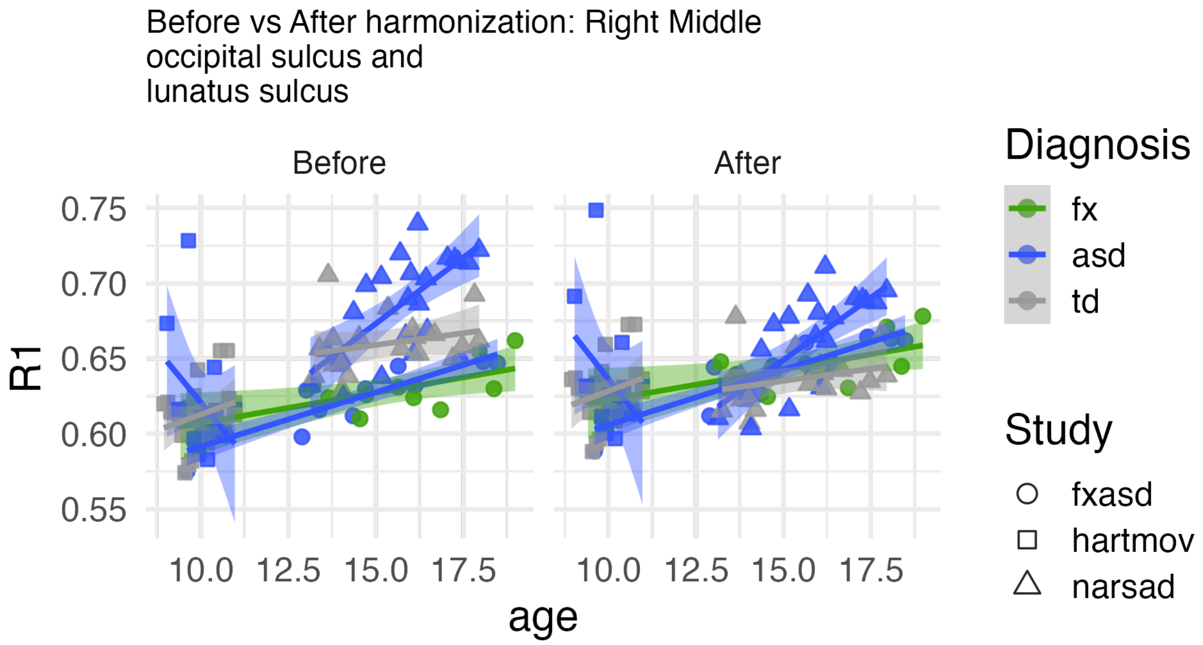

Before vs After harmonization: Right Middle
occipital sulcus and
lunatus sulcus
Before
After
R1
0.75
0.70
0.65
0.60
0.55
10.0
12.5
15.0
17.5
age
Diagnosis
fx
asd
td
Study
fxasd
hartmov
narsad

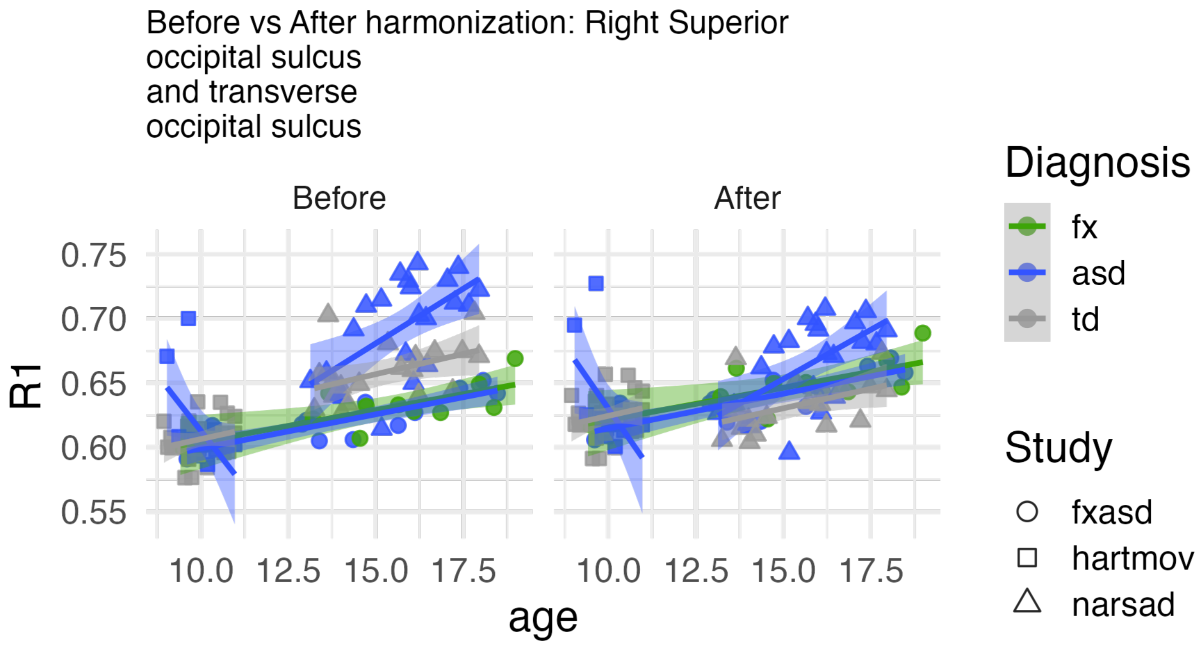

Before vs After harmonization: Right Superior
occipital sulcus
and transverse
occipital sulcus
Before
After
R1
0.75
0.70
0.65
0.60
0.55
10.0
12.5
15.0
17.5
age
Diagnosis
fx
asd
td
Study
fxasd
hartmov
narsad

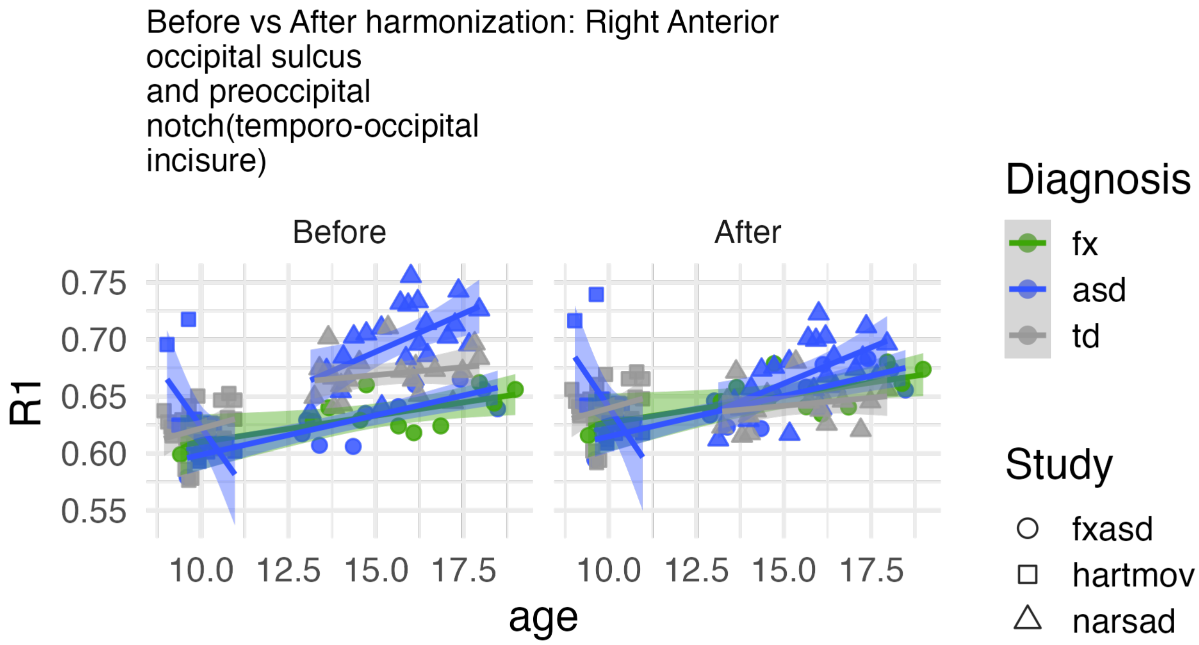
Before vs After harmonization: Right Anterior
occipital sulcus
and preoccipital
notch(temporo-occipital
incisure)
Before
After
R1
0.75
0.70
0.65
0.60
0.55
10.0
12.5
15.0
17.5
age
Diagnosis
fx
asd
td
Study
fxasd
hartmov
narsad

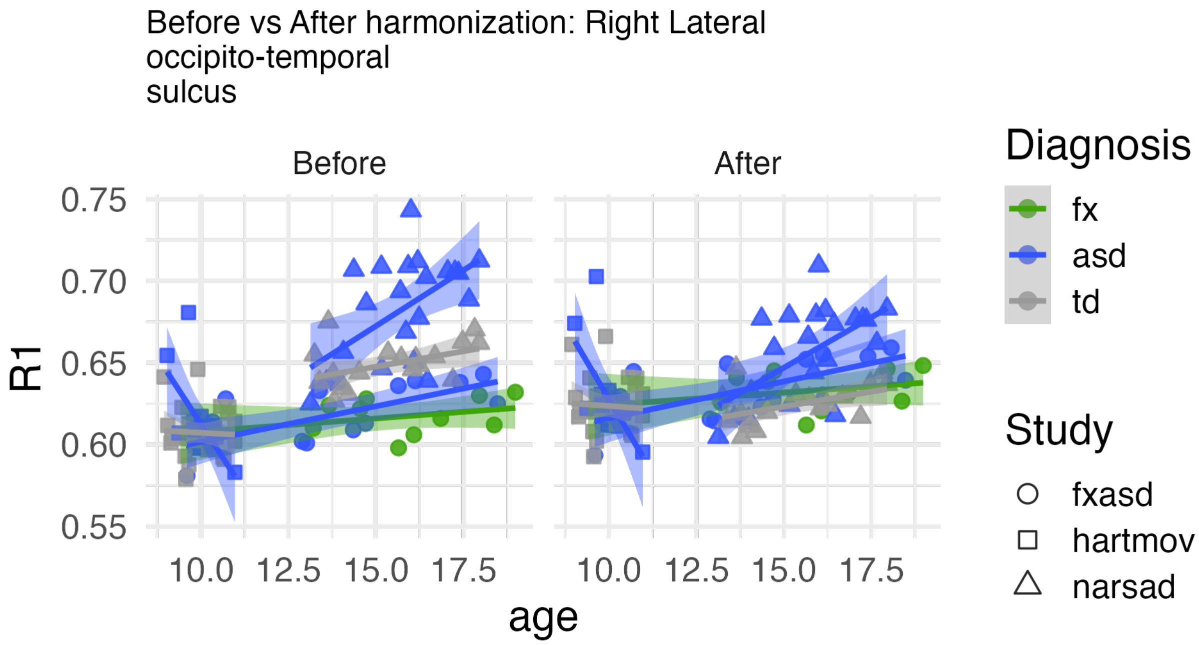
Before vs After harmonization: Right Lateral
occipito-temporal
sulcus
Before
After
R1
0.75
0.70
0.65
0.60
0.55
10.0
12.5
15.0
17.5
age
Diagnosis
fx
asd
td
Study
fxasd
hartmov
narsad

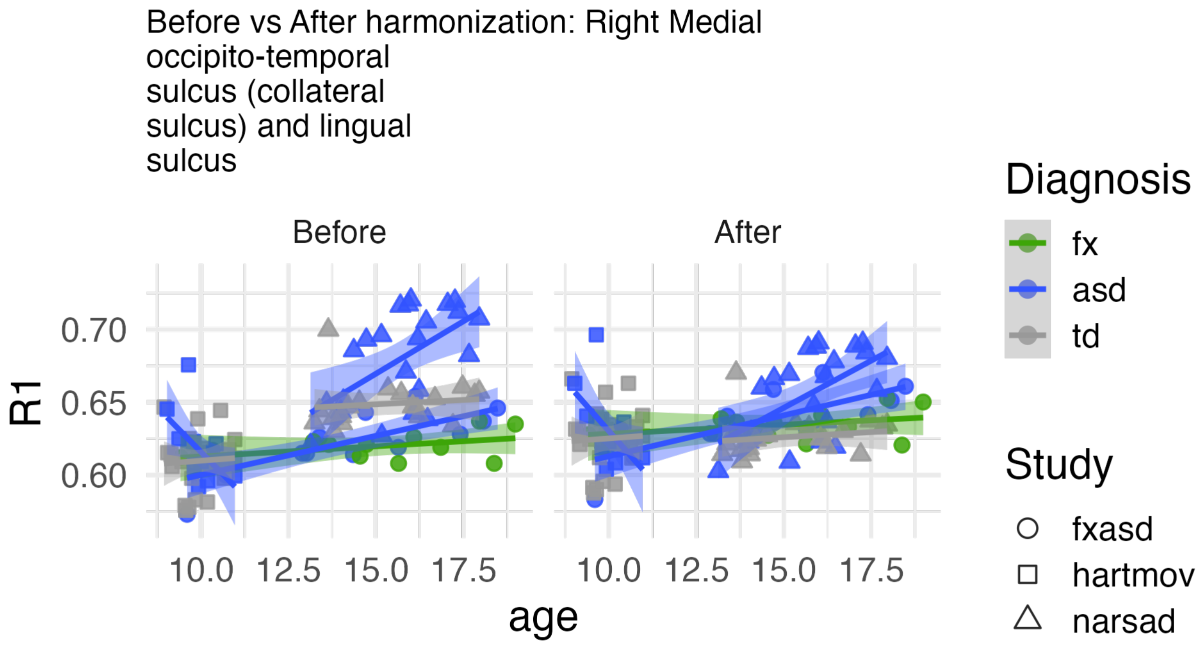
Before vs After harmonization: Right Medial
occipito-temporal
sulcus (collateral
sulcus) and lingual
sulcus
Before
After
R1
0.70
0.65
0.60
10.0
12.5
15.0
17.5
age
Diagnosis
fx
asd
td
Study
fxasd
hartmov
narsad

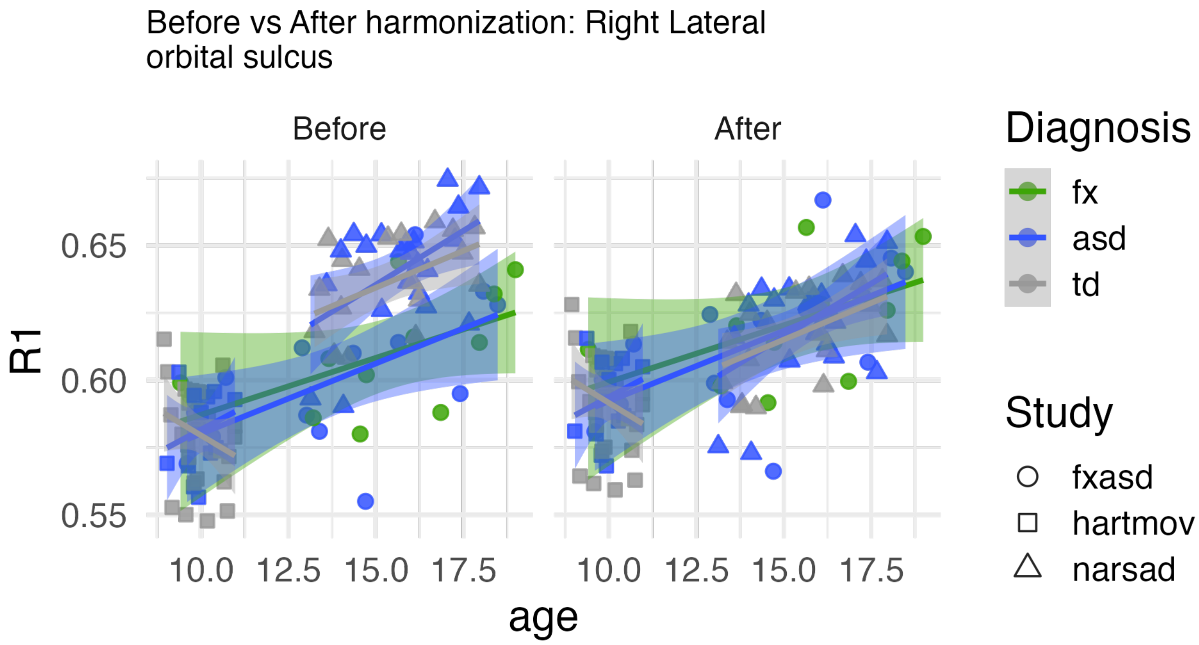
Before vs After harmonization: Right Lateral
orbital sulcus
Before
After
R1
0.65
0.60
0.55
10.0
12.5
15.0
17.5
age
Diagnosis
fx
asd
td
Study
fxasd
hartmov
narsad

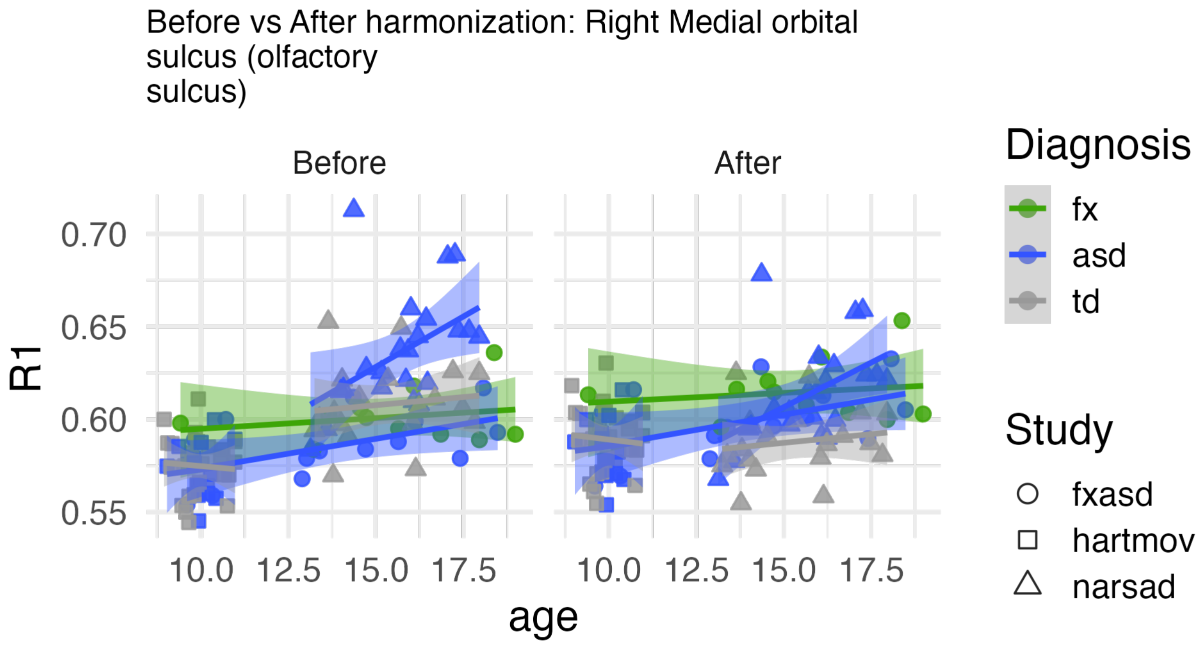

Before vs After harmonization: Right Medial orbital
sulcus (olfactory
sulcus)
Before
After
R1
0.70
0.65
0.60
0.55
10.0
12.5
15.0
17.5
age
Diagnosis
fx
asd
td
Study
fxasd
hartmov
narsad

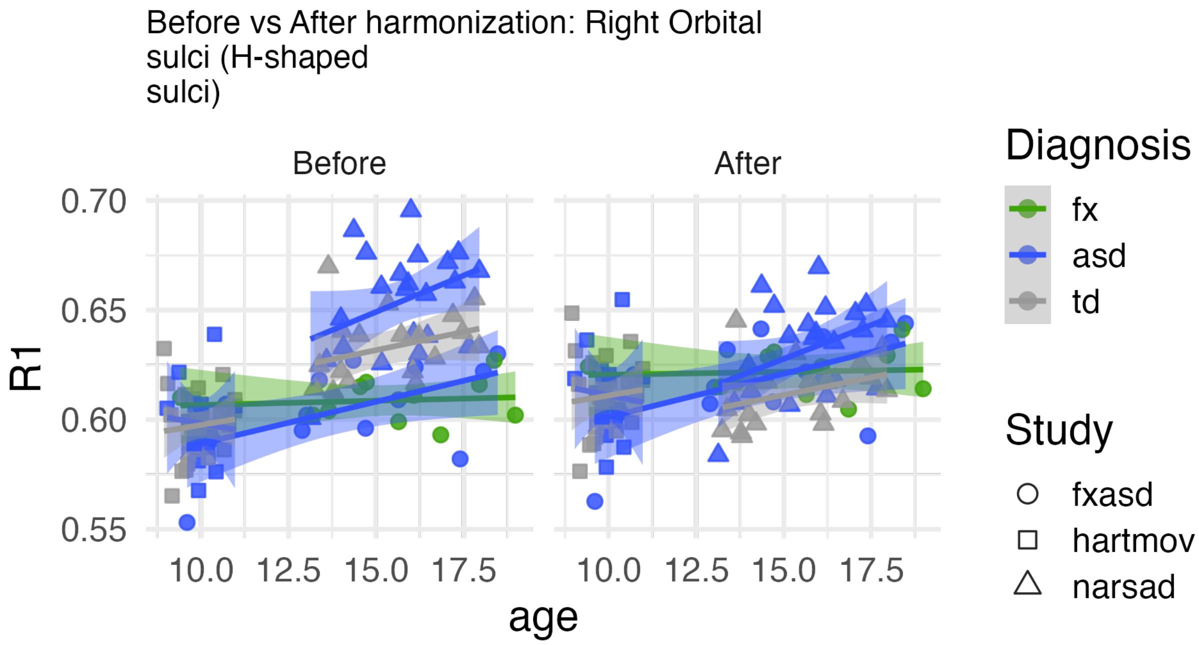

Before vs After harmonization: Right Orbital
sulci (H-shaped
sulci)
Before
After
R1
0.70
0.65
0.60
0.55
10.0
12.5
15.0
17.5
age
Diagnosis
fx
asd
td
Study
fxasd
hartmov
narsad

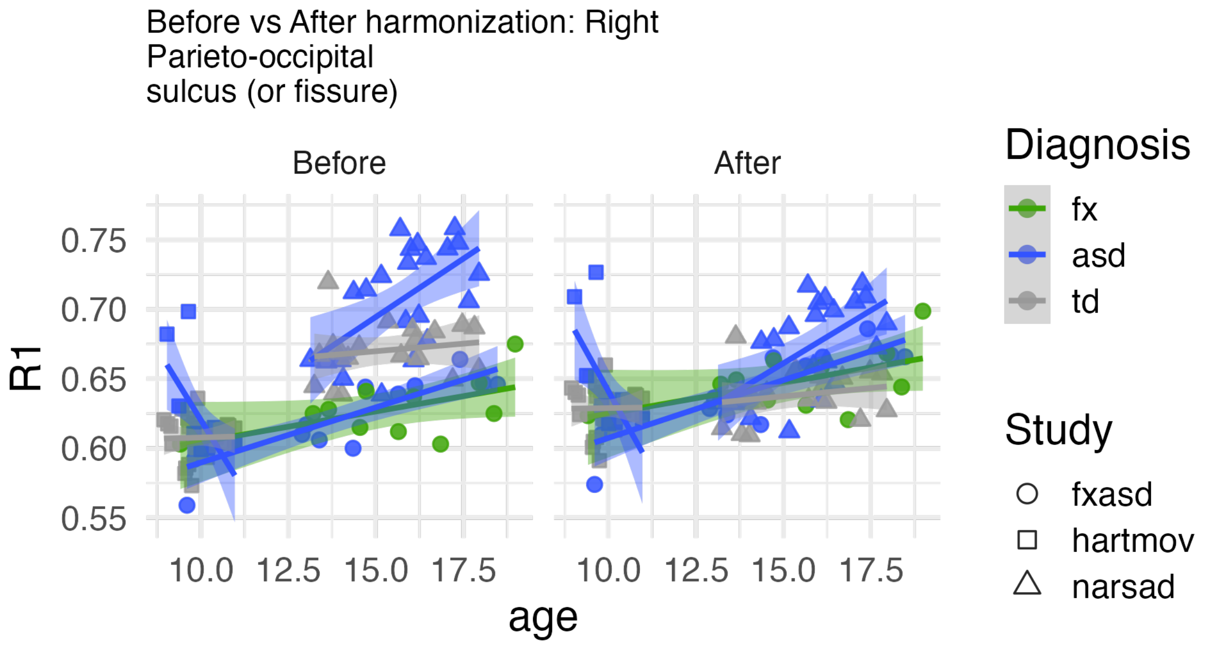
Before vs After harmonization: Right
Parieto-occipital
sulcus (or fissure)
Before
After
R1
0.75
0.70
0.65
0.60
0.55
10.0
12.5
15.0
17.5
age
Diagnosis
fx
asd
td
Study
fxasd
hartmov
narsad

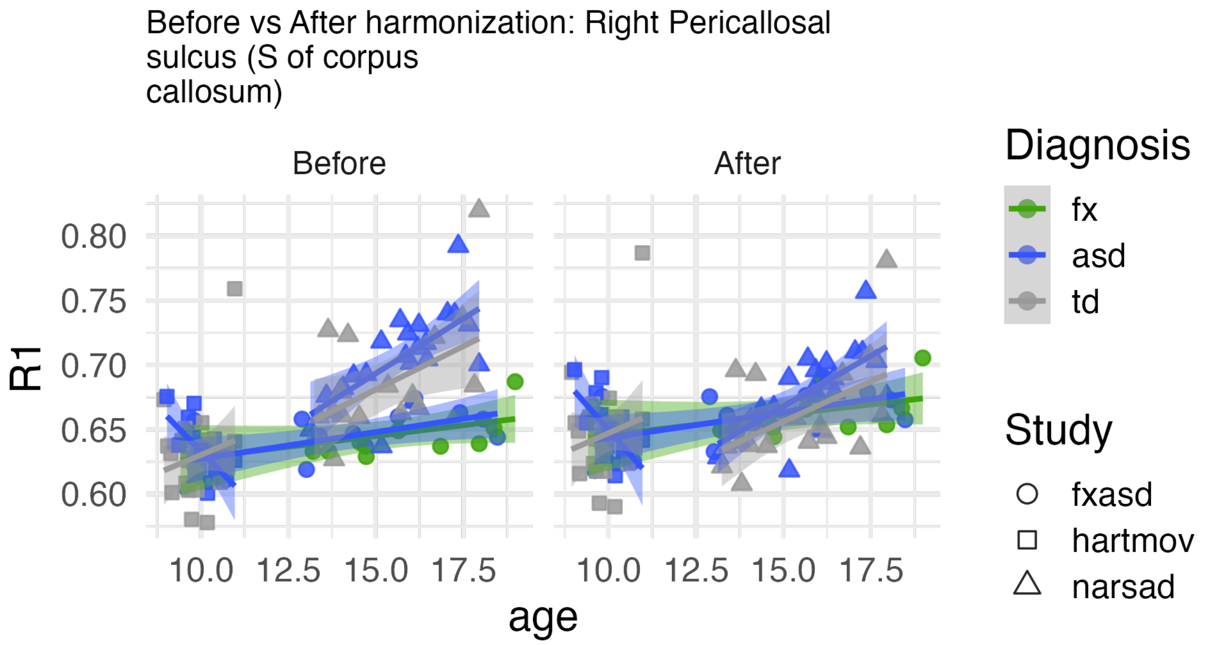
Before vs After harmonization: Right Pericallosal
sulcus (S of corpus
callosum)
Before
After
R1
0.80
0.75
0.70
0.65
0.60
10.0
12.5
15.0
17.5
age
Diagnosis
fx
asd
td
Study
fxasd
hartmov
narsad

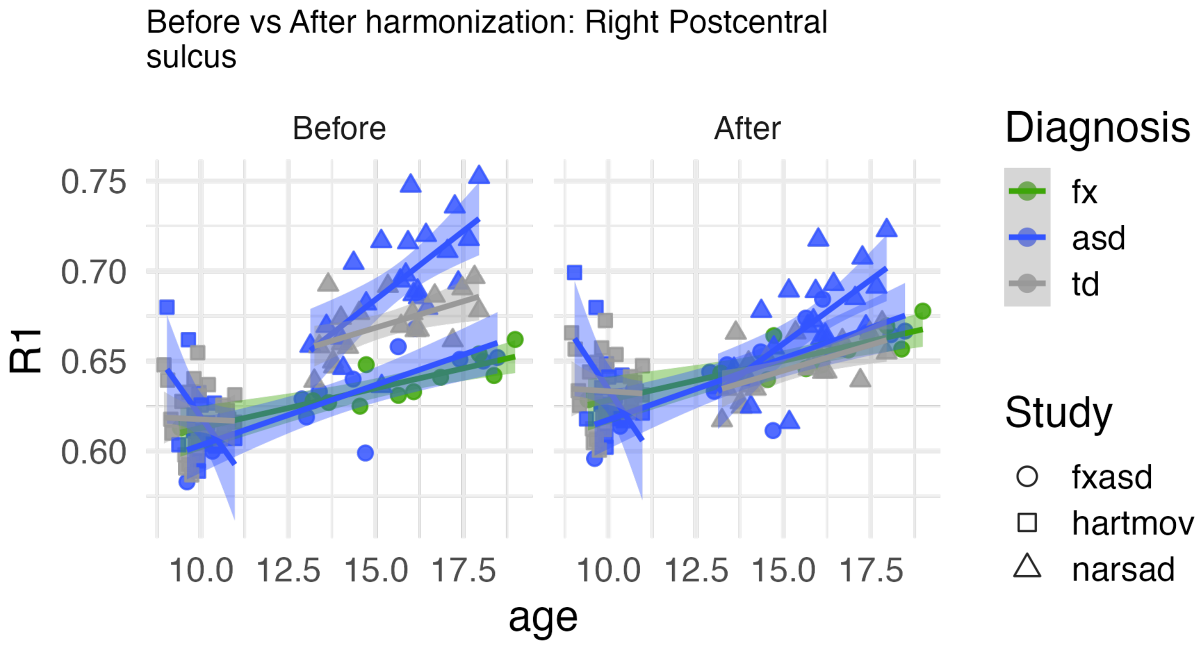
Before vs After harmonization: Right Postcentral sulcus
Before
After
R1
0.75
0.70
0.65
0.60
10.0
12.5
15.0
17.5
age
Diagnosis
fx
asd
td
Study
fxasd
hartmov
narsad

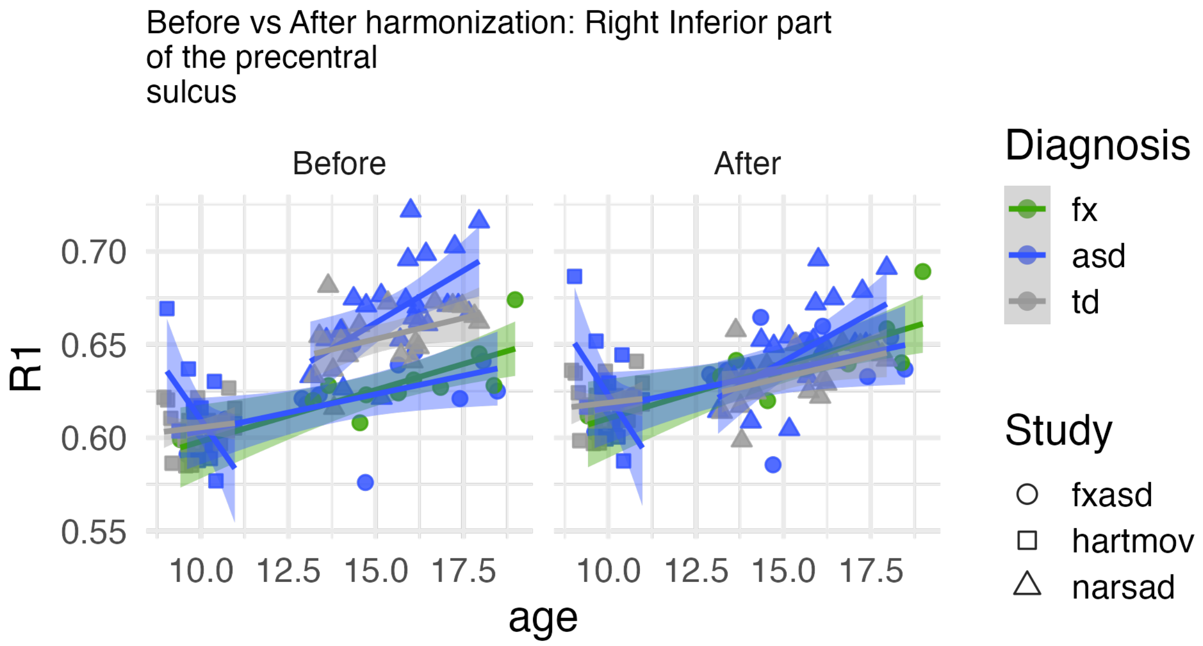
Before vs After harmonization: Right Inferior part of the precentral sulcus
Before
After
R1
0.70
0.65
0.60
0.55
10.0
12.5
15.0
17.5
age
Diagnosis
fx
asd
td
Study
fxasd
hartmov
narsad

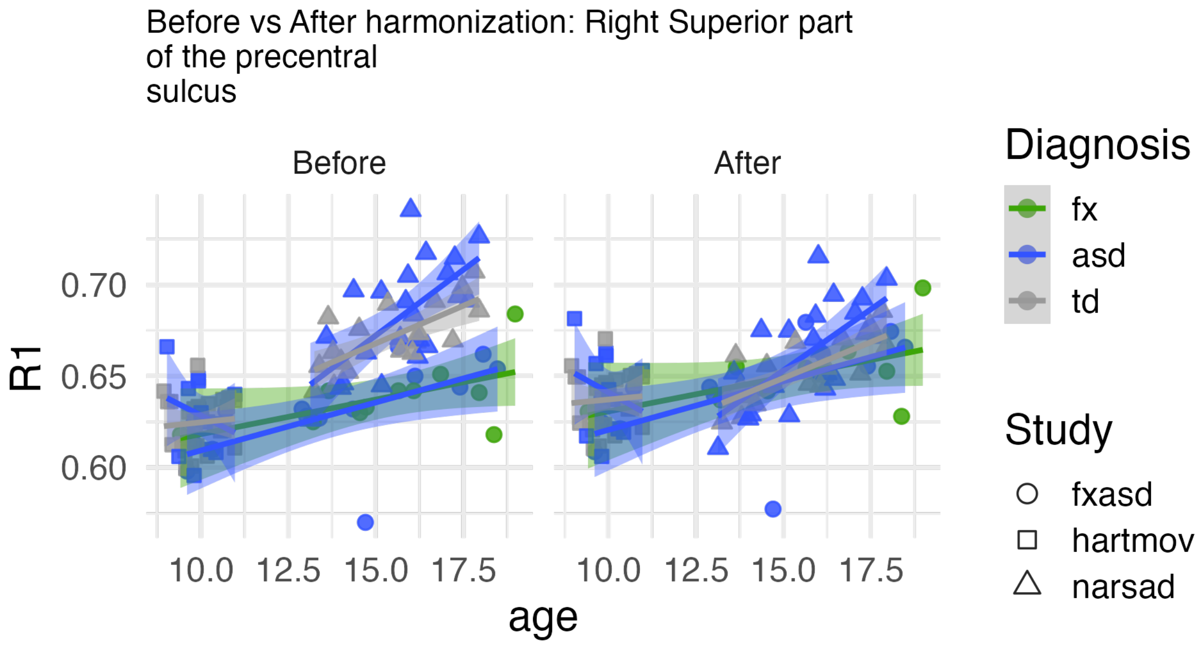

Before vs After harmonization: Right Superior part
of the precentral
sulcus
Before
After
R1
0.70
0.65
0.60
10.0
12.5
15.0
17.5
age
Diagnosis
fx
asd
td
Study
fxasd
hartmov
narsad

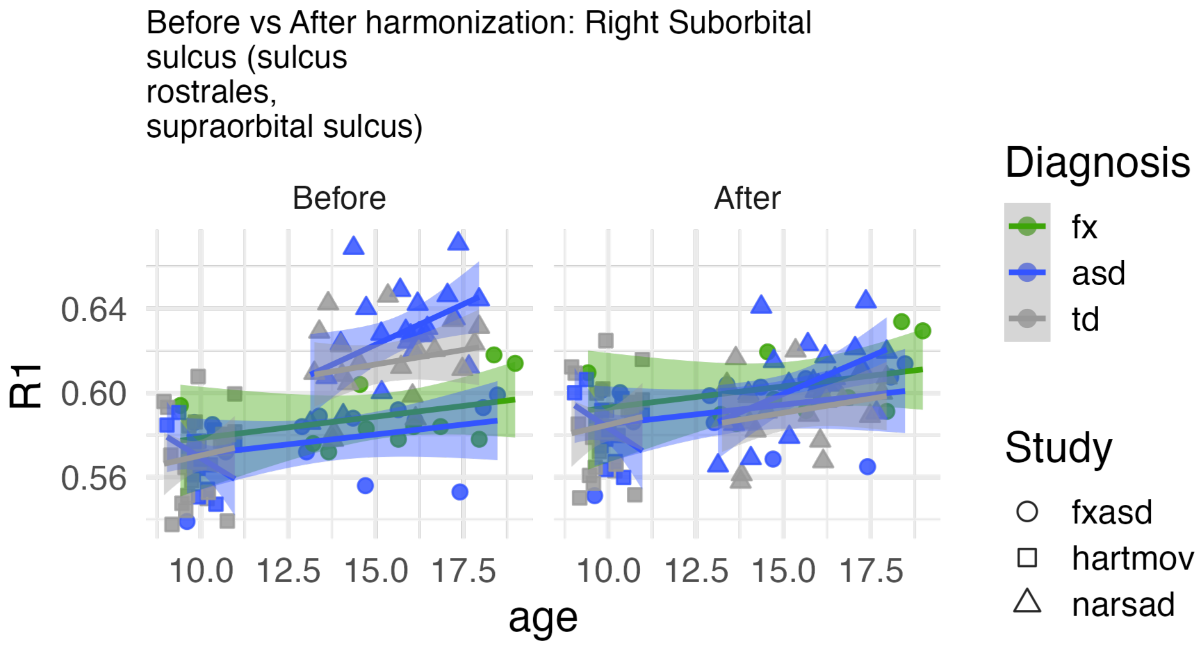

Before vs After harmonization: Right Suborbital
sulcus (sulcus
rostrales,
supraorbital sulcus)
Before
After
R1
0.64
0.60
0.56
10.0
12.5
15.0
17.5
age
Diagnosis
fx
asd
td
Study
fxasd
hartmov
narsad

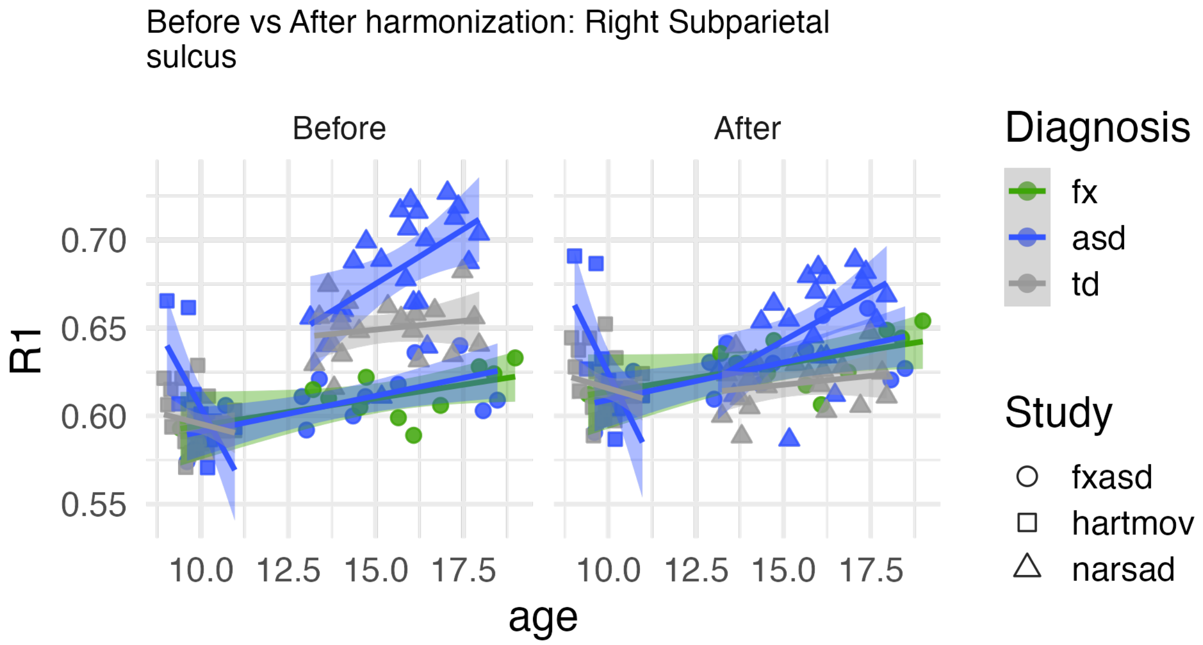
Before vs After harmonization: Right Subparietal sulcus
Before
After
R1
0.70
0.65
0.60
0.55
10.0
12.5
15.0
17.5
age
Diagnosis
fx
asd
td
Study
fxasd
hartmov
narsad

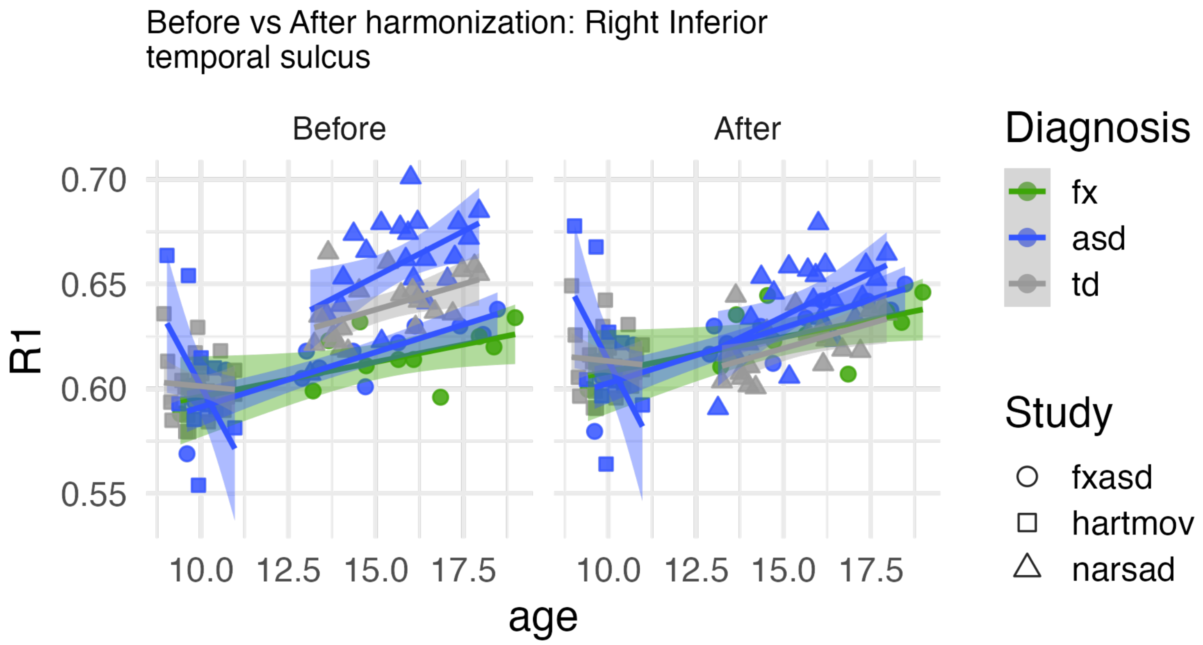
Before vs After harmonization: Right Inferior temporal sulcus
Before
After
R1
0.70
0.65
0.60
0.55
10.0
12.5
15.0
17.5
age
Diagnosis
fx
asd
td
Study
fxasd
hartmov
narsad

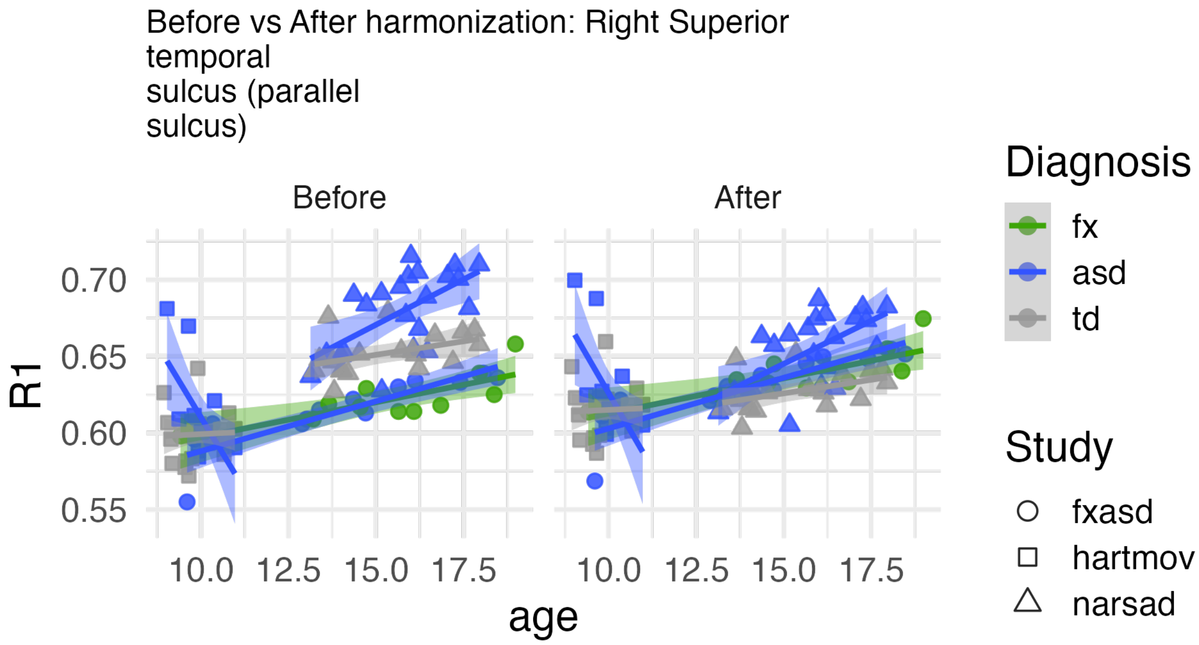
Before vs After harmonization: Right Superior
temporal
sulcus (parallel
sulcus)
Before
After
R1
0.70
0.65
0.60
0.55
10.0
12.5
15.0
17.5
age
Diagnosis
fx
asd
td
Study
fxasd
hartmov
narsad

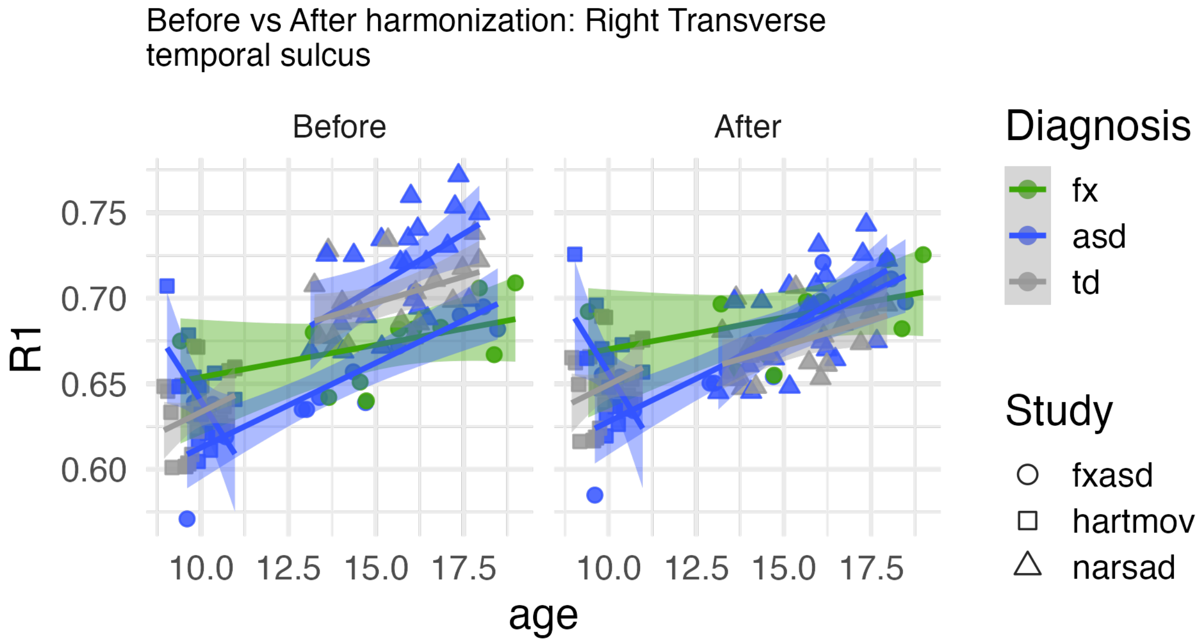
Before vs After harmonization: Right Transverse
temporal sulcus
Before
After
R1
0.75
0.70
0.65
0.60
10.0
12.5
15.0
17.5
age
Diagnosis
fx
asd
td
Study
fxasd
hartmov
narsad

Supplement

Section 2A. Significant group differences in cortical thickness for FXS and ASD

Cortical thickness was modeled linearly with age, group, and age-by-group terms. Table 1 below shows results from comparing FXS to ASD data from the original and added groups combined. False discovery rate (FDR) was used to control the number of false positives due to multiple comparisons.

Table 1: **Significant statistical results from group comparisons in thickness of cortical regions.**

		Right				Left			
**Label**	unit	**effect**	**cohens**	**Uncorr. p**	**FDR Corr. p**	**effect**	**cohens**	**Uncorr. p**	**FDR Corr. p**
G_and_S_frontomargin	1	1	0.462	**0.012823**	0.065442	0	-0.166	0.318259	0.480637
G_and_S_occipital_inf	2	1	-1.161	**0.000000**	**0.000010**	1	-0.923	**0.000009**	**0.000277**
G_and_S_subcentral	4	1	-0.609	**0.004421**	**0.031161**	0	-0.336	0.098663	0.235517
G_and_S_cingul.Mid.Ant	7	0	0.149	0.437332	0.610484	1	0.508	**0.013968**	0.068910
G_cuneus	11	1	-1.085	**0.000000**	**0.000017**	1	-0.752	**0.000164**	**0.001731**
G_front_inf.Opercular	12	1	-0.500	**0.020400**	0.094350	1	-0.454	**0.028247**	0.108633
G_front_inf.Triangul	14	0	1.061	0.085114	0.213505	1	-0.441	**0.029997**	0.110989
G_occipital_middle	19	1	-0.769	**0.000033**	**0.000701**	1	-0.928	**0.000002**	**0.000065**
G_occipital_sup	20	1	-0.919	**0.000001**	**0.000065**	1	-0.749	**0.000131**	**0.001488**
G_oc.temp_lat.fusifor	21	0	-0.368	0.066927	0.190485	1	-0.655	**0.000686**	**0.005638**
G_oc.temp_med.Lingual	22	0	-0.086	0.682492	0.789131	1	-0.443	**0.033582**	0.115583
G_oc.temp_med.Parahip	23	0	-0.069	0.751165	0.848644	1	0.661	**0.002471**	**0.019248**
G_orbital	24	0	-0.282	0.134532	0.304271	1	-0.390	**0.038145**	0.125454
G_pariet_inf.Angular	25	1	-0.653	**0.000183**	**0.001803**	1	-0.494	**0.009024**	**0.049466**
G_pariet_inf.Supramar	26	0	-0.273	0.151807	0.320963	1	-0.428	**0.028626**	0.108633
G_parietal_sup	27	1	-0.428	**0.004333**	**0.031161**	1	-0.368	**0.022587**	0.100214
G_precentral	29	0	-0.132	0.543275	0.699172	2	-1.353	**0.032843**	0.115583
G_precuneus	30	1	-0.586	**0.000303**	**0.002803**	1	-0.818	**0.000014**	**0.000352**
G_temp_sup.G_T_transv	33	0	-0.356	0.088312	0.217836	1	-0.463	**0.028505**	0.108633
G_temp_sup.Lateral	34	0	-0.027	0.894677	0.926411	1	-0.429	**0.048347**	0.152240
G_temporal_middle	38	0	-0.209	0.310445	0.480578	1	-0.450	**0.023150**	0.100214
Pole_occipital	42	1	-0.917	**0.000048**	**0.000821**	1	-0.927	**0.000057**	**0.000848**
S_calcarine	44	1	-0.506	**0.006895**	**0.042518**	1	-0.501	**0.008358**	**0.047579**
S_central	45	0	-0.168	0.408743	0.581672	2	-1.401	**0.007580**	**0.044872**
S_collat_transv_ant	50	1	0.444	**0.031834**	0.114911	0	0.045	0.815703	0.881198
S_collat_transv_post	51	1	-0.548	**0.005028**	**0.033828**	1	-0.706	**0.000604**	**0.005259**
S_front_middle	53	1	0.455	**0.024376**	0.100214	0	0.100	0.590860	0.728727
S_interm_prim.Jensen	55	1	-0.331	**0.045267**	0.145643	0	-0.376	0.071309	0.191885
S_oc_middle_and_Lunatus	57	1	-0.835	**0.000050**	**0.000821**	1	-0.782	**0.000074**	**0.001001**
S_oc_sup_and_transversal	58	1	-0.462	**0.017939**	0.085644	0	-0.379	0.055965	0.169037
S_oc.temp_lat	60	0	-0.227	0.271484	0.441534	1	-0.430	**0.011072**	0.058526
S_parieto_occipital	65	1	-0.681	**0.000093**	**0.001151**	0	-0.316	0.081236	0.207292
S_subparietal	71	1	-0.493	**0.006722**	**0.042518**	1	-0.440	**0.034808**	0.117083
S_temporal_sup	73	0	-0.239	0.167747	0.341919	1	-0.412	**0.024267**	0.100214

Supplement

Section 2B. Significant group differences in qR1 for FXS and ASD

Same as for cortical thickness, qR1 was modeled linearly with age, group, and age-by-group terms. Table 2 below shows non-FDR corrected significant results from comparing FXS to ASD data from the original and added groups combined. In the pages below, plots are shown for these regions. Also for reference, qR1 measures from typically developing (td) children are plotted alongside the FXS and ASD groups.

Table 2: **Non-FDR corrected significant statistical results from group comparisons in qR1 of cortical regions.**

		Right				Left			
**Label**	unit	**effect**	**cohens**	**Uncorr. p**	**FDR Corr. p**	**effect**	**cohens**	**Uncorr. p**	**FDR Corr. p**
G_and_S_paracentral	3	2	-1.148	**0.047379**	0.466988	0	0.273	0.059899	0.471524
G_and_S_cingul.Mid.Post	8	1	0.482	**0.011252**	0.424113	0	0.154	0.386376	0.680759
G_oc.temp_lat.fusifor	21	1	0.371	**0.049224**	0.466988	0	0.280	0.113253	0.475188
G_oc.temp_med.Parahip	23	1	0.463	**0.026028**	0.466988	1	-0.554	**0.012737**	0.424113
G_parietal_sup	27	0	0.287	0.069684	0.471524	1	0.348	**0.016936**	0.424113
G_precuneus	30	1	0.391	**0.016599**	0.424113	1	0.341	**0.038853**	0.466988
Lat_Fis.ant.Vertical	40	0	0.101	0.576733	0.791191	1	0.347	**0.037830**	0.466988
Pole_temporal	43	1	0.410	**0.017194**	0.424113	0	0.093	0.615966	0.801429
S_cingul.Marginalis	46	1	0.390	**0.027039**	0.466988	0	0.137	0.419462	0.705212
S_collat_transv_ant	50	0	0.212	0.224654	0.593729	2	-1.246	**0.037617**	0.466988
S_oc.temp_lat	60	1	0.412	**0.030390**	0.466988	0	0.135	0.474613	0.732578
S_oc.temp_med_and_Lingual	61	1	0.382	**0.041360**	0.466988	0	0.286	0.086021	0.471524
S_subparietal	71	0	0.310	0.104166	0.475188	1	0.448	**0.010127**	0.424113

Frontal lobe

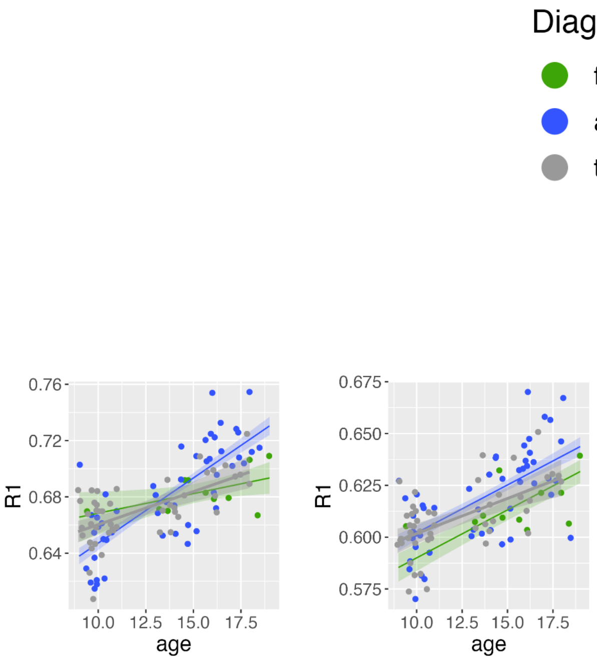

Right · Paracentral lobule and sulcus

Left · Vertical ramus of the anterior segment of the lateral sulcus(or fissure)

Parietal lobe

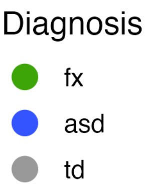

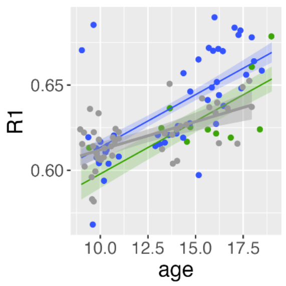

Left · Precuneus (medial part of P1)

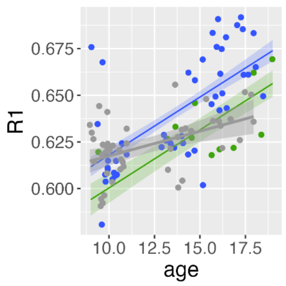

Right · Precuneus (medial part of P1)

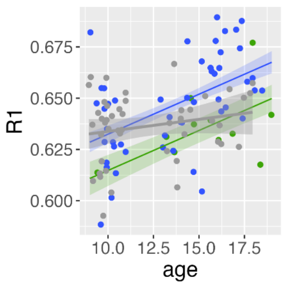

Right · Middle-posterior part of the cingulate gyrus and sulcus(pMCC)

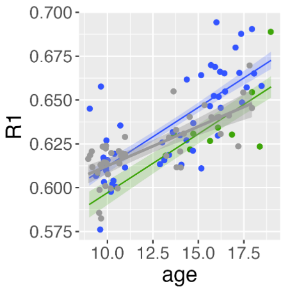

Left · Superior parietal lobule (lateral part of P1)

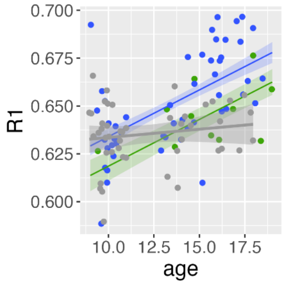

Right · Marginal branch (or part) of the cingulate sulcus

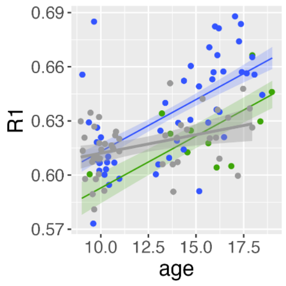

Left · Subparietal sulcus

Temporal lobe

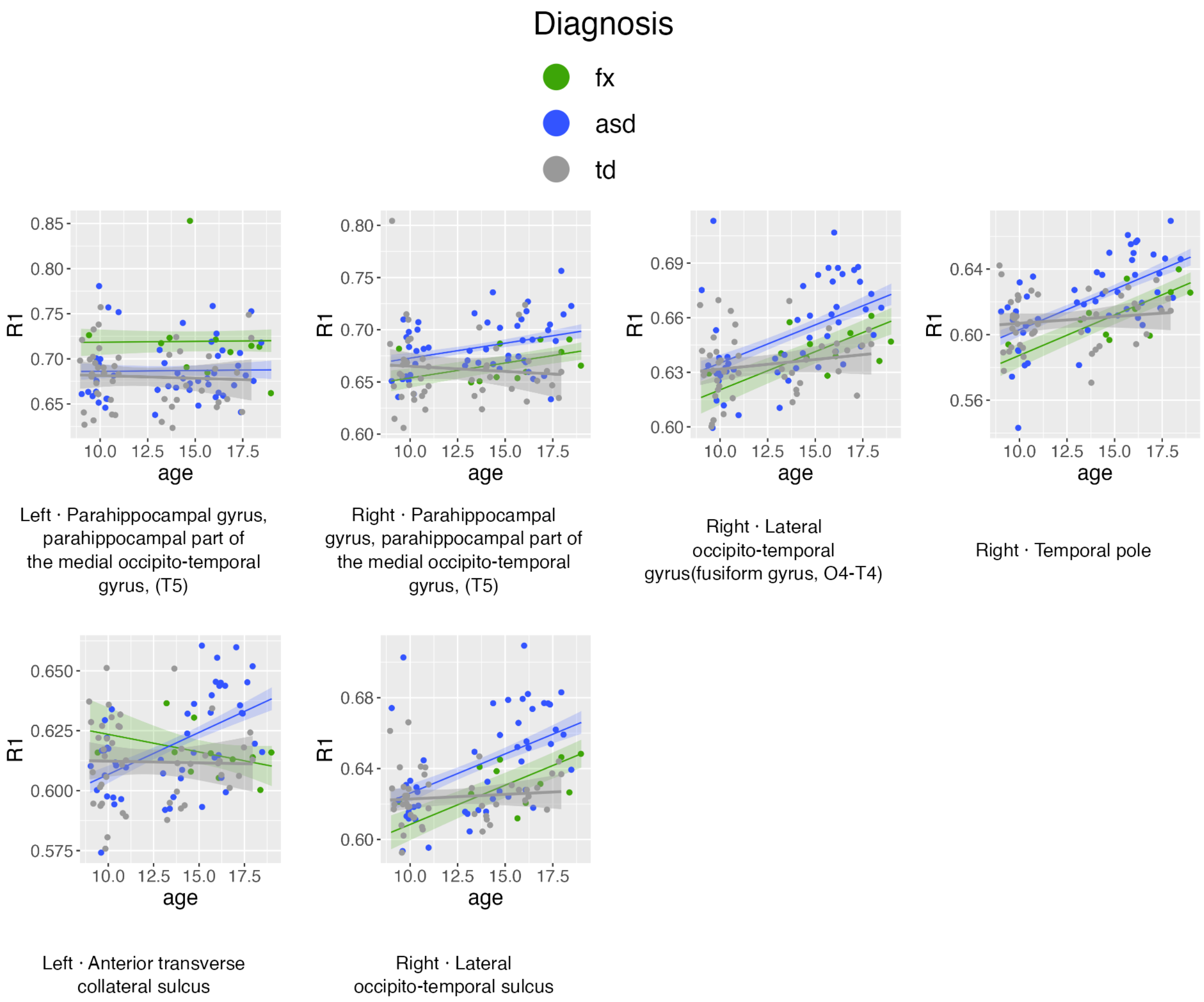

Occipital lobe

Diagnosis

fx

asd

td

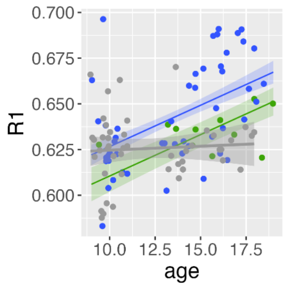

Right · Medial occipito-temporal sulcus (collateral sulcus) and lingual sulcus

Supplement

Section 3A. Exploratory within-FX subgroup analysis

In order to characterize the impact of heterogenous ADOS-2 comparison scores within-FXS on the primary analysis findings, we conducted an exploratory analysis stratifying the FXS cohort into low (ADOS-2 CS <5; n=4) and moderate-to-high (ADOS-2 CS ≥5; n=7). Focusing on the cortical regions that showed significant FXS-ASD differences in the primary analysis, we modeled cortical thickness as a function of FXS severity subgroup and age. Only two regions, the left fusiform gyrus and the right superior occipital gyrus, had significant differences between the two subgroups with lower cortical thickness in the "moderate-to-high" subgroup. However, no effects survived FDR correction, likely related to the limited statistical power of 4 vs 7 comparison. These findings are summarized in the plots below.

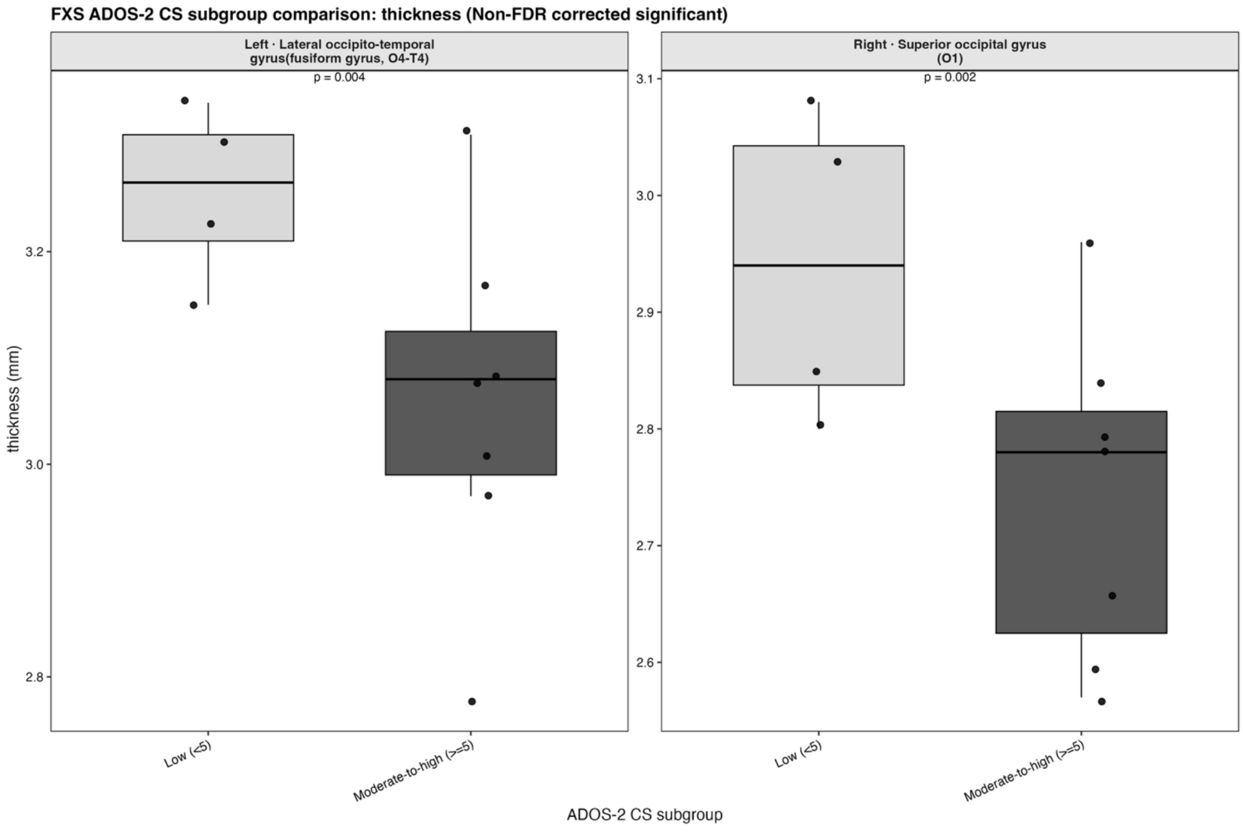

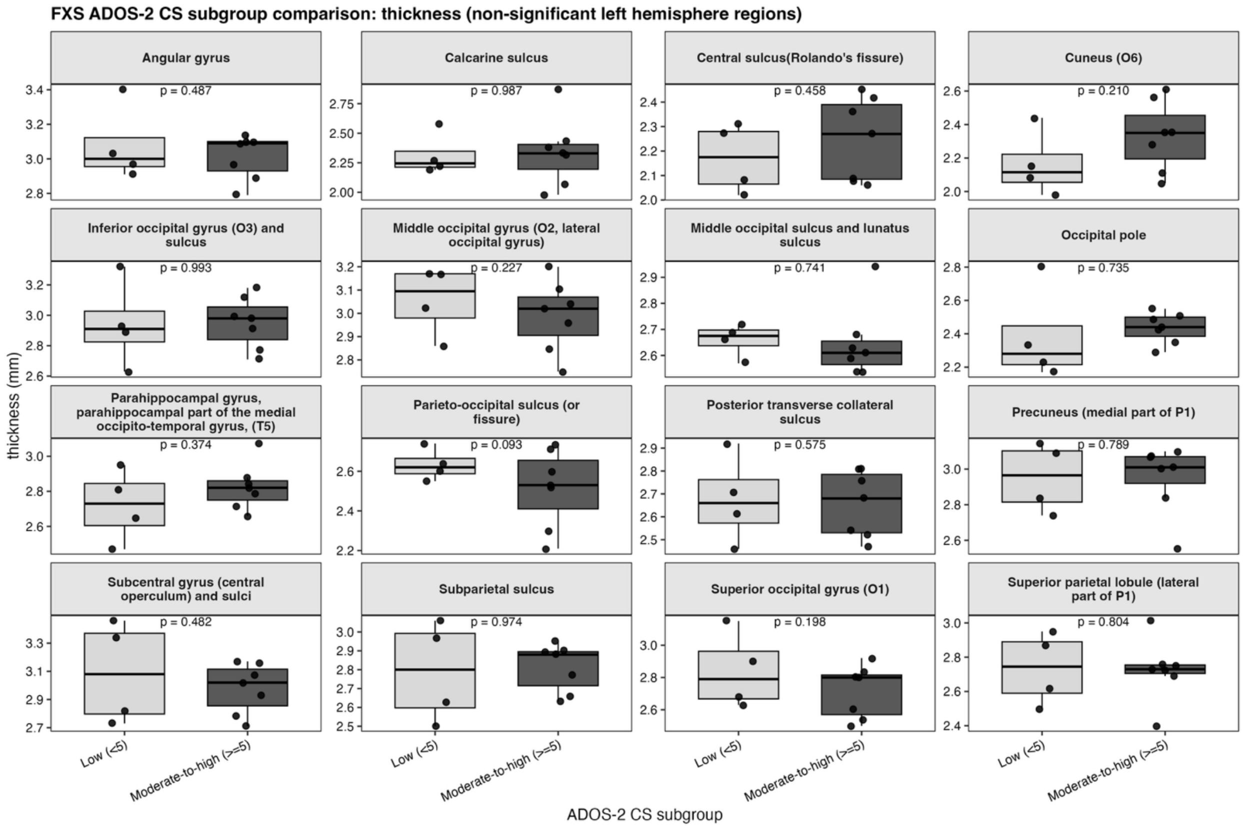

FXS ADOS-2 CS subgroup comparison: thickness (non-significant left hemisphere regions)
Angular gyrus
p = 0.487
Calcarine sulcus
p = 0.987
Central sulcus(Rolando's fissure)
p = 0.458
Cuneus (O6)
p = 0.210
Inferior occipital gyrus (O3) and sulcus
p = 0.993
Middle occipital gyrus (O2, lateral occipital gyrus)
p = 0.227
Middle occipital sulcus and lunatus sulcus
p = 0.741
Occipital pole
p = 0.735
Parahippocampal gyrus, parahippocampal part of the medial occipito-temporal gyrus, (T5)
p = 0.374
Parieto-occipital sulcus (or fissure)
p = 0.093
Posterior transverse collateral sulcus
p = 0.575
Precuneus (medial part of P1)
p = 0.789
Subcentral gyrus (central operculum) and sulci
p = 0.482
Subparietal sulcus
p = 0.974
Superior occipital gyrus (O1)
p = 0.198
Superior parietal lobule (lateral part of P1)
p = 0.804
thickness (mm)
Low (<5)
Moderate-to-high (>=5)
ADOS-2 CS subgroup

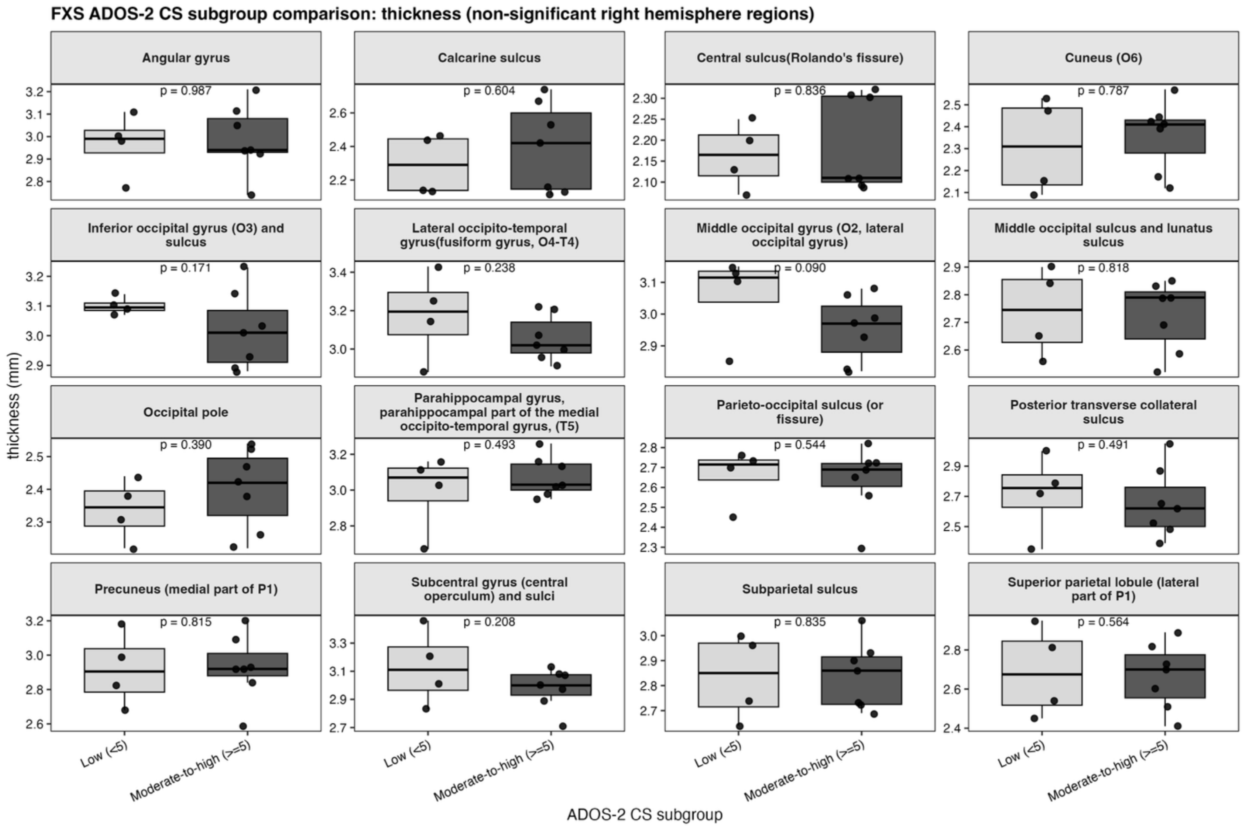

FXS ADOS-2 CS subgroup comparison: thickness (non-significant right hemisphere regions)
Angular gyrus
p = 0.987
Calcarine sulcus
p = 0.604
Central sulcus(Rolando's fissure)
p = 0.836
Cuneus (O6)
p = 0.787
Inferior occipital gyrus (O3) and sulcus
p = 0.171
Lateral occipito-temporal gyrus(fusiform gyrus, O4-T4)
p = 0.238
Middle occipital gyrus (O2, lateral occipital gyrus)
p = 0.090
Middle occipital sulcus and lunatus sulcus
p = 0.818
Occipital pole
p = 0.390
Parahippocampal gyrus, parahippocampal part of the medial occipito-temporal gyrus, (T5)
p = 0.493
Parieto-occipital sulcus (or fissure)
p = 0.544
Posterior transverse collateral sulcus
p = 0.491
Precuneus (medial part of P1)
p = 0.815
Subcentral gyrus (central operculum) and sulci
p = 0.208
Subparietal sulcus
p = 0.835
Superior parietal lobule (lateral part of P1)
p = 0.564
thickness (mm)
Low (<5)
Moderate-to-high (>=5)
ADOS-2 CS subgroup
